# Photochemical three-component assembly of tri-substituted oxazoles through a carbenic phosphorus-nitrile hybrid ylide formation/trapping cascade†

**DOI:** 10.1039/d4sc01355g

**Published:** 2024-04-05

**Authors:** Xingchen Ye, Huaijin Pan, Yong Huang, Jiean Chen, Zhaofeng Wang

**Affiliations:** a State Key Laboratory of Chemo/Biosensing and Chemometrics, College of Chemistry and Chemical Engineering, Hunan University Changsha Hunan 410082 P. R. China zfwangchem@hnu.edu.cn; b Department of Chemistry, The Hong Kong University of Science and Technology Clear Water Bay Kowloon Hong Kong SAR P. R. China yonghuang@ust.hk; c Pingshan Translational Medicine Center, Shenzhen Bay Laboratory Shenzhen 518118 P. R. China chenja@szbl.ac.cn

## Abstract

Construction of complex molecular skeletons with ubiquitous chemical feedstocks in a single transformation is highly appealing in organic synthesis. We report a novel visible-light-induced three-component reaction for the construction of complex 2,4,5-trisubstituted oxazoles, which are valuable in medicinal chemistry, from simple and readily available iodonium-phosphonium hybrid ylides, carboxylic acids, and nitriles. This reaction features a carbenic phosphorus-nitrile hybrid ylide formation/trapping cascade, in which a photo-generated α-phosphonium carbene acts as a sequence trigger. This catalyst- and additive-free transformation exhibits high efficiency and broad substrate scope for synthesizing diverse oxazoles.

## Abstract 2

A photochemical three-component reaction for synthesizing tri-substituted oxazoles has been reported. This protocol featured simple starting materials, mild reaction conditions, broad substrate scope and wide synthetic utility.

## Graphical abstract

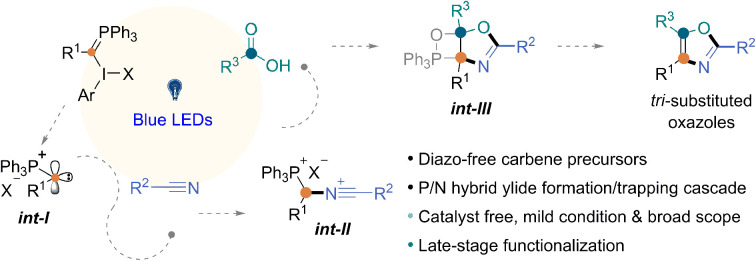

## Introduction

**Fig. 1:**
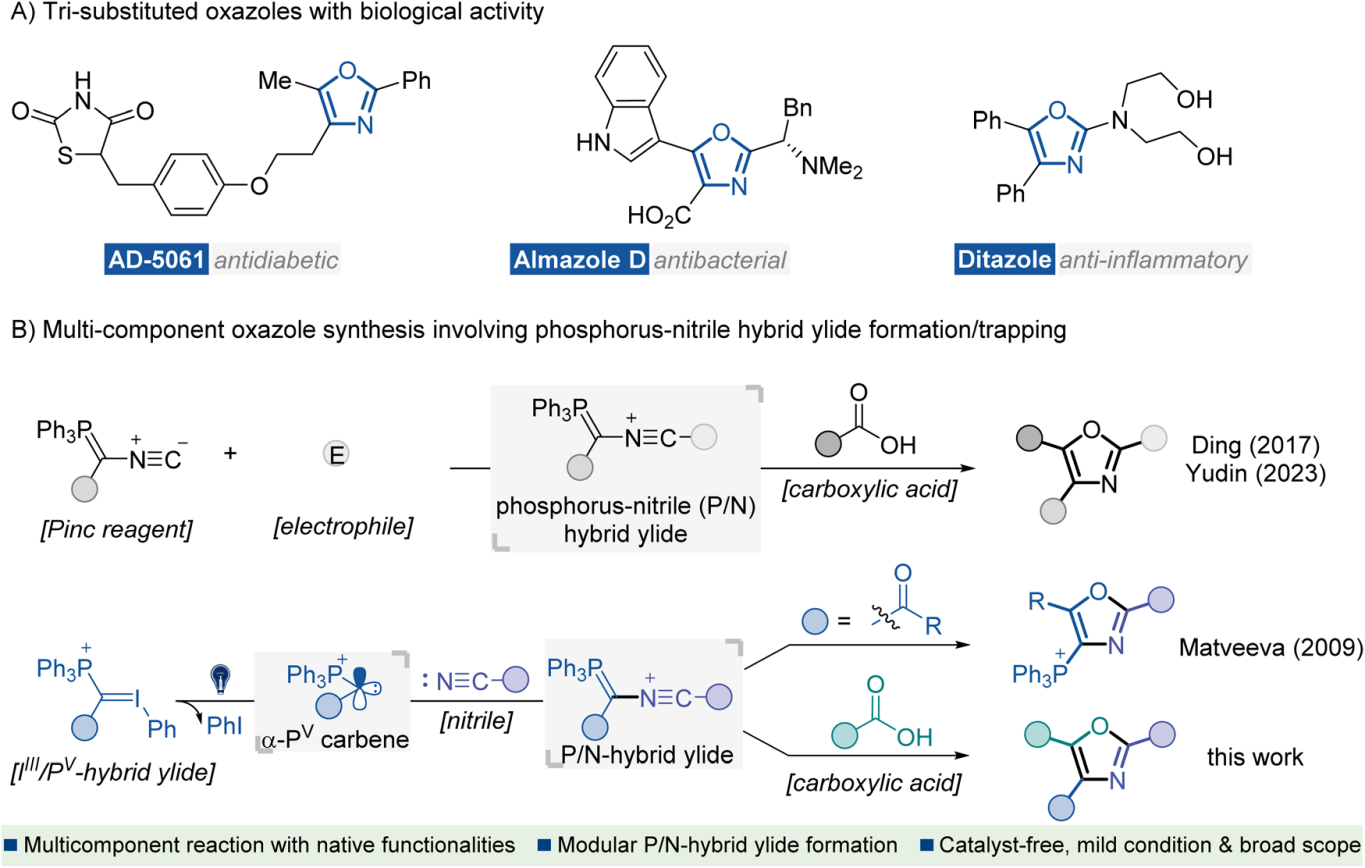
Introduction. (A) Representative bioactive molecules containing tri-substituted oxazole motifs. (B) MCRs for the synthesis of oxazoles *via* a P/N hybrid ylide formation/trapping cascade.

Heterocyclic compounds containing nitrogen and oxygen atoms are key structural units found in many biologically active natural products and synthetic pharmaceutical agents.^1^ In particular, oxazoles represent an important subclass of this family which exhibits versatile therapeutic activities, such as antidiabetic, antibacterial, and anti-inflammatory activities (Fig. 1A).^2^ Consequently, the development of efficient methods for preparing oxazole scaffolds has been a hot and challenging topic for organic synthetic chemists.^3^ In the past few decades, several catalytic and other methods for the synthesis of oxazole derivatives have been well documented, such as intramolecular^4^ or intermolecular^5^ cyclization of acyclic precursors, metal-catalyzed coupling of functionalized oxazoles with suitable reagents,^6^ and oxidations of oxazolines,^7^ or other relevant protocols.^8^ Despite significant advances, most methods face the limitation of inaccessible starting materials and the utilization of transition-metal catalysts or additional oxidants. Moreover, reports on the direct approach to fully functionalized oxazoles are scarce. Developing an eco-friendly catalyst-free synthetic procedure for preparing highly substituted oxazole derivatives from readily available starting materials is of great interest.

Nitrile ylides are highly reactive 1,3-dipoles containing a CNC framework with six electrons distributed across π and n orbitals.^9^ They have proven to be key intermediates in versatile synthetic methods for rapidly assembling complex N-heterocycles.^10^ Recently, the renaissance of multi-component reactions (MCRs) has been driven, not only due to their high efficiency, convergent nature and straightforward experimental procedures but also because of their value to the pharmaceutical industry for construction of structurally complex and functionally diverse molecules from several easily accessible components.^11^ In this context, MCRs involving the formation of nitrile ylides have shown robust efficiency in the construction of highly substituted oxazoles.^8*b*,12^ Representative studies have been disclosed by Ding^12*a*^ and Yudin^12*c*^ (Fig. 1B, up), which involve electrophilic interceptions with the Pinc reagent^13^ to generate phosphorus-nitrile hybrid ylide (P/N ylide), thus initiating a carboxylic acid trapping/olefination cascade for the rapid construction of polyfunctionalized oxazoles. Key to these studies was the use of multifunctional Pinc reagent decorated bonds with an isocyanide moiety and a phosphorus ylide functionality. However, the multi-step synthetic procedure towards the Pinc reagent and inherent limited substitution types afflicted their application in modular synthesis of oxazoles with structural diversity.

In 1984, Moriarty *et al.* reported the synthesis of a new series of stable iodonium ylides through the nucleophilic addition of a phosphorus ylide to an activated hypervalent iodine compound.^14^ Owing to d-orbital stabilization from the phosphorus atom, these compounds exist as bench-stable hybrid ylides (I^III^-ylide and P^V^-ylide) with partial C=P and C=I bonding on the central carbon. Recently, Matveeva reported novel UV-light driven reactions between these iodonium-phosphonium hybrid ylides (I^III^/P^V^ ylides) and nitriles to yield phosphonium substituted oxazoles.^15^ Mechanistic study indicated that the oxazole ring probably formed *via* intramolecular cyclization of P/N ylide, which originated from nitrile addition to *in situ* photo-generated singlet α-phosphonium carbene (α-P^V^ carbene) species.^16^ Encouraged by this complementary P/N ylide formation strategy and our continuous interest in reactivity exploration of I^III^/P^V^ hybrid ylides,^17^ we envision that the *in situ* generated α-P^V^ carbene species might be compatible for the successive interception of nitriles and carboxylic acids to enable an expeditious MCR for access to tri-substituted oxazole compounds (Fig. 1B, down).There are several challenges associated with this novel cascade MCR: (1) multiple active intermediates, including α-P^V^ carbenes, nitrile-phosphorus ylides and carboxylate ions are involved, which may cause predictable, competitive reactions, such as direct O–H insertion of a carboxylic acid into carbene, ylide formation between carbonyl groups and carbene species, as well as the intramolecular cyclization of nitrile-phosphorus ylides (Matveeva's work); (2) poor reactivity of the nitrile group; an additional activator or excess amount of nitrile partner may be needed and (3) as a kind of reagent bearing two hypervalent leaving groups, I^III^/P^V^ hybrid ylides are highly oxidative and fragile, which could easily oxidize carboxylic acids and thus decompose under photo-irradiation conditions.^18^

## Results and discussion

To validate the feasibility of the above-mentioned hypothesis, our investigation commenced with the three-component reaction of I^III^/P^V^ mixed ylide 1a, *p*-tolyl carboxylic acid 2a and acetonitrile (as solvent). Considering the high leaving group ability of I^III^ and UV-vis absorption spectra of I^III^/P^V^ ylides (see the ESI† for details), we envisioned that these molecules might be activated under visible light photochemical conditions. After screening various reaction parameters, we found that the desired 2,4,5-trisubstituted oxazole 3a could be obtained in good yield after irradiation with 36 W blue LEDs (*λ*_max_ = 438 nm) for 7 hours under a nitrogen atmosphere at room temperature with Na_2_CO_3_ as a base additive (Table 1, entry 1). The counter ion of mixed ylides (1b and 1c) moderately influenced the overall conversion (entries 2 and 3). Further reduced conversion for the desired product was observed using the neutral cyclic reagent 1d, likely due to the lower tendency for I^(III)^ to be excited under photoactivation (entry 4). The reaction became sluggish under aerobic conditions, and no product was formed in the dark (entries 5 and 6). Increasing the reaction temperature did not help improve the yield (entry 7). Organic base Et_3_N performed poorly, and stronger base Cs_2_CO_3_ hindered the reaction significantly (entries 8 and 9). Irradiating the reaction mixture with white or less intensive blue LEDs resulted in slightly lower yields (entries 10 and 11). No noticeable improvement was achieved when extending the reaction time to 24 hours (entry 12). The corresponding sodium carboxylate could also afford the desired product in comparable yield while not meeting the step economy from a practical perspective (entry 13). Due to the inert reactivity of the cyanide functional group, replacing the solvent with 1,2-dichloroethane and using 10.0 equivalents of acetonitrile almost quenched the transformation.

**Table 1:** UV-vis spectra of 1 at 10^−4^ M and optimization of photochemical three-component synthesis of tri-substituted oxazoles^a^

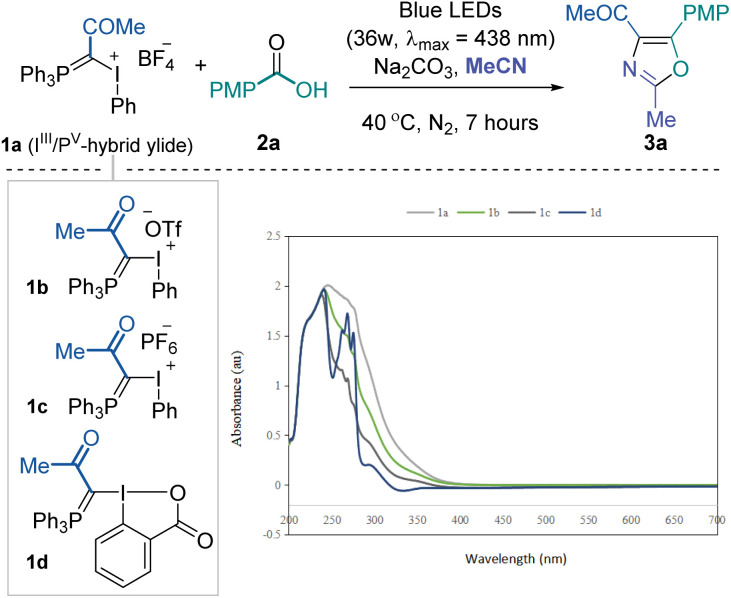		
Entry	Deviation from standard conditions	3a^b^ [%]
1	None	75 (74)
2	1b instead of 1a	66
3	1c instead of 1a	48
4	1d instead of 1a	10
5	No degas	45
6	No light	N.D.
7	At 60 °C	69
8	Et_3_N as base	6
9	Cs_2_CO_3_ as base	27
10	White LEDs	66
11	1 W blue LEDs	72
12	24 h reaction time	66
13	Sodium carboxylate instead of acid	57
14	DCE as solvent, 10.0 equiv. of MeCN	Trace

a Reaction conditions: I^III^/P^V^ hybrid ylide reagent 1 (0.1 mmol), *p*-toluic acid 2a (0.12 mmol), Na_2_CO_3_ (0.24 mmol) in degassed acetonitrile (1.0 mL), under irradiation of 36 W blue LEDs at 40 °C for 7 hours.

b Yields are reported based on ^1^H-NMR analysis using 1,3,5-(OMe)_3_C_6_H_3_ as the internal standard. The number in paratheses reflects isolated yield. PMP, *p*-methyl phenyl. DCE, 1,2-dichloroethane.

With the optimal reaction conditions established, we investigated the generality of this three-component conjunctive reaction (Table 2). First, a wide range of aromatic carboxylic acids was evaluated. Different para-substituted benzoic acids were compatible, providing desired products in moderate to good yields (3a–3p). To illustrate the synthetic utility of the reaction, a 5.0 mmol scale reaction was implemented under the standard conditions, producing the oxazole product 3a in 70% yield (0.75 g). The reactivity was almost consistent between electron-withdrawing (3b–3i) and electron-donating (3a, 3j–3n) groups. In particular, terminal alkynyl (3o) and vinyl (3p) substituents were compatible with the reaction conditions; no [2 + 1] cyclopropane product from these unsaturated bonds with free α-phosphonium carbene intermediate was observed. The *meta*- and *ortho*-substituted benzoic acids proceeded well to give the desired products regardless of the electronic properties of the substituents (3q–3s). Heteroaryl-substituted acids were also competent substrates for this reaction to afford modest yields (3t–3v). Tetrafluoroterephthalic acid was examined, and the desired di-oxazole product was isolated in 38% yield (3w). Base additives were critical for this transformation, different carboxylic acids, which differ in electronic or steric properties require different base additives to form nucleophilic carboxylic anion efficiently. Next, a broad array of aliphatic carboxylic acids was subjected to the reaction system and reacted with 1a in acetonitrile. Generally, higher yields were achieved when aliphatic sodium carboxylates were used as substrates instead of corresponding carboxylic acids. Various primary sodium carboxylates bearing linear substituents were suitable substrates, providing the corresponding oxazoles in good yields (3x–3aa). Exclusive chemo-selectivity was realized with substrate containing ester functionality (3ab). The compatibility with alkyl bromide highlighted the complementarity of this methodology to the traditional Wittig approach, which usually involves phosphonium slats generation *via* nucleophilic attack from phosphine to alkyl halides (3ac). It could also be applied to naturally occurring carboxylic acid (3ad) and common pharmaceutical motifs with steric bulky secondary and tertiary substitutes (3ae–3af). Successful transformation of α,β-unsaturated cinnamic acid further demonstrated the chemo-selectivity of this transformation (3ag–3ah). We tested our methodology on a series of I^III^/P^V^ mixed ylide reagents and substituted nitriles. Several benzoyl-substituted I^III^/P^V^-ylides smoothly underwent the desired reaction pathway (3ai–3ak). With benzoyl substituted I^III^/P^V^ mixed ylide reagent, oxazoles bearing –CF_3_ (3al) and *ortho*-phenyl groups (3am) could be assembled, which are elusive to synthesize following the reported methods. The highest reaction efficiency was achieved with the isopropyl acyl-substituted ylide reagent, possibly due to a delicate balance of reactivity and stability (3an). A similar yield was obtained for *n*-propyl substitutions (3ao). Low yield was observed for the ester-type I^III^/P^V^ ylide, even with AuCl(PPh_3_) as a nitrile activator (3ap, 21%).^19^

**Table 2:** Evaluation of substrate scope^a^

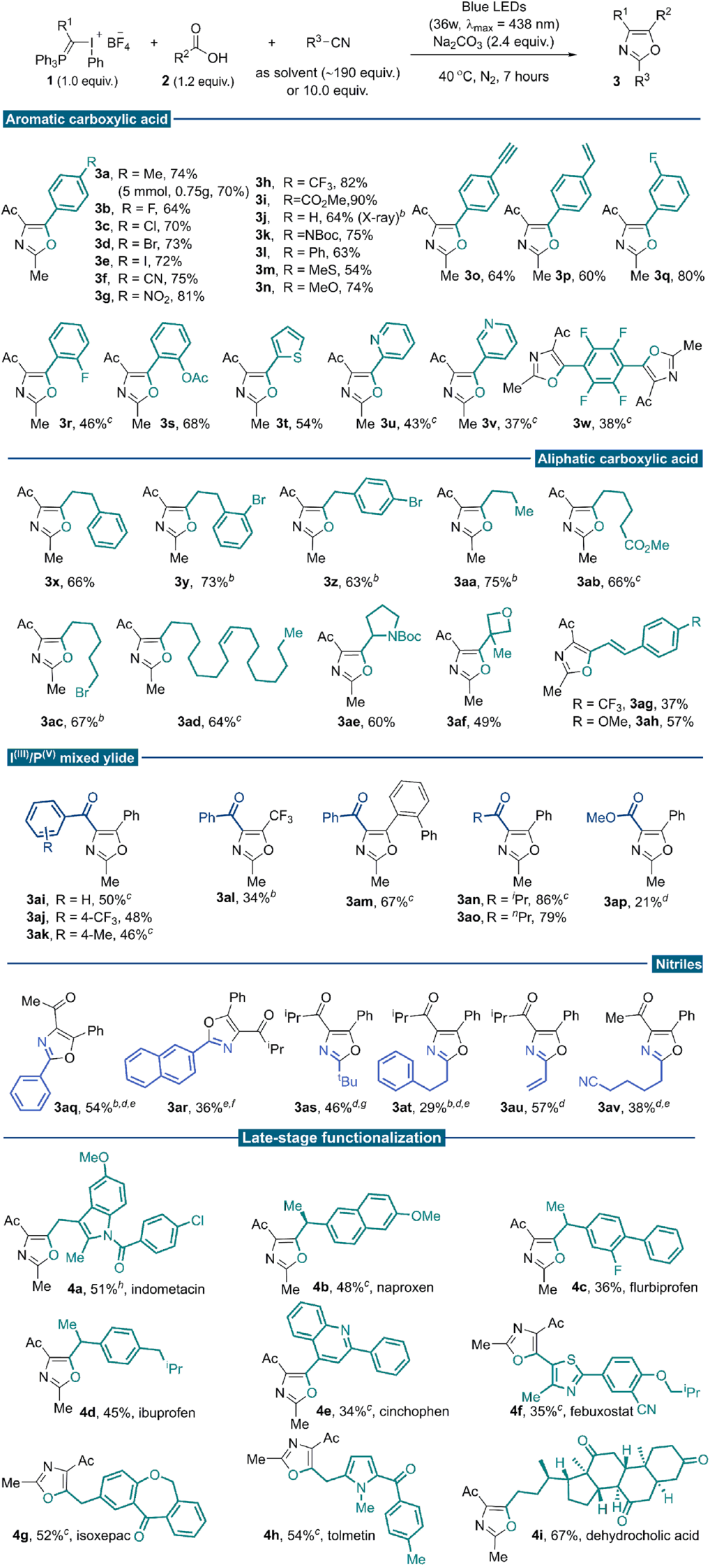

a Reaction conditions: reagent 1 (0.1 mmol), carboxylic acid 2 (0.12 mmol), Na_2_CO_3_ (0.24 mmol) in degassed acetonitriles (1.0 mL), under irradiation of 36 W blue LEDs at 40 °C for 7 hours. Data are reported as isolated yields of the purified compound.

b Sodium carboxylates (0.12 mmol) were used instead of carboxylic acids.

c Cs_2_CO_3_ (0.24 mmol) as a base.

d Au(PPh_3_)Cl (5 mol%) was added as a catalyst.

e 1,2-dichloroethane (1.0 mL) was used as the solvent, with 1.0 mmol of nitrile substrate (10.0 equiv.) and Au(PPh_3_)Cl (5 mol%).

f K_3_PO_4_ (0.24 mmol) as base.

g Ammonium benzoate (0.12 mmol) was used instead of benzoic acids.

h CsF (0.24 mmol) as base.

This reaction tolerated other nitrile solvents, such as trimethylacetonitrile (3as) and acrylonitrile (3au); decent yields were obtained in these two cases. Having demonstrated the broad reaction scope with nitrile as the solvent, we turned to situations where the nitrile is expensive and/or not commercially available. Further condition screening identified that 10.0 equivalents of acetonitrile in 1,2-diethylchloride could lead to a 42% yield merging a gold catalysis synergy.^20^ With further optimized conditions, benzonitrile (3aq), 2-naphthonitrile (3ar) and 3-phenylpropanenitrile (3at) reacted smoothly to afford functionalized oxazoles in moderate yields. With 1,6-hexanedinitrile as the partner, a selective mono-annulation gave oxazole nitrile 3av an acceptable yield.

Carboxylic acids are prevalent in more than 450 marketed drug molecules.^21^ Therefore, the chemoselectivity of this reaction offers a unique opportunity to pursue late-stage oxazole installation of bioactive molecules richly decorated with reactive functionalities. It features a metal-free protocol that could better accommodate the C_sp_^2^–X bonds and heterocyclic moieties. Several carboxylic acids bearing the moieties of marketed drugs and natural products were investigated under standard reaction conditions. As a result, tri-substituted oxazoles were successfully installed onto potential bioactive compounds such as indometacin, naproxen, flurbiprofen, ibuprofen, cinchophen, febuxostat, isoxepac, tolmetin and dehydrocholic acid to give products 4a–4i in moderate yields. Gratifyingly, versatile functionalities such as amides, ketones and heterocyclic skeletons could be well tolerated under standard photochemical conditions. These derivatives otherwise could only be accessible through multi-step functional group interconversions or *de novo* synthesis (Table 2, late-stage functionalization part).

**Fig. 2:**
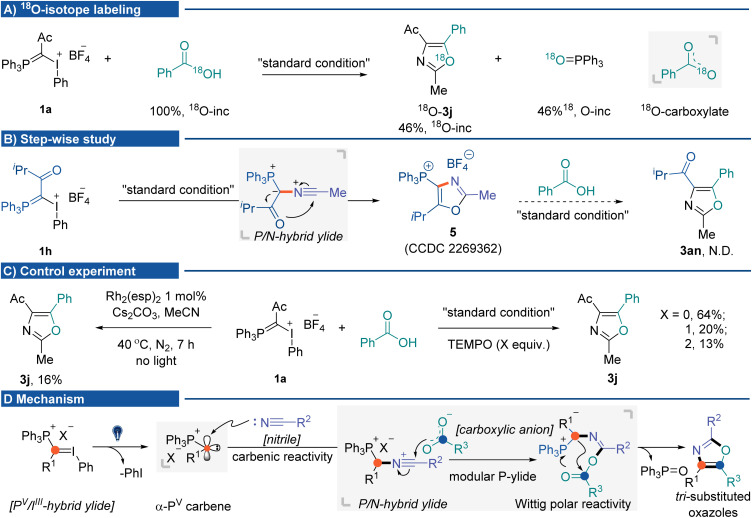
Mechanistic studies. (A) ^18^O-Labeling experiment. (B) Step-wise experiment. (C) Control experiment. (D) Possible mechanism.

The mechanistic insights into the photochemical three-component oxazole synthesis were then investigated. The coupling of I^III^/P^V^ hybrid ylide 1a with PhCO^18^ OH was performed to trace the source of oxygen in the oxazole scaffold. It gave ^18^O-labeled oxazole 3j in 60% yield (46% ^18^O-inc), with the generation of PPh_3_=^18^O (46% ^18^O-inc). The ^18^O-labeling experiment supported two equivalent oxygen atoms from the carboxylate anion nucleophilically attacking P/N hybrid ylide (Fig. 2A). A step-wise study indicated that without carboxylic acid, the P/N hybrid ylide cyclized intramolecularly, affording phosphonium-substituted oxazole 5.^16^ Once 5 was formed, it could not be transformed into targeted product 3an during the subsequent reaction process in the presence of carboxylic acid and base (Fig. 2B).^22^ Several control experiments were conducted to understand mechanistic details (Fig. 2C) better. The three-component reaction could also be promoted by dirhodium catalysts in the absence of light, albeit in lower yield (16%, using Du Bois' catalyst Rh_2_(esp)_2_).^23^ The addition of 2,2,6,6-tetramethylpiperidinyloxy (TEMPO) did not significantly affect the product formation. These results suggested carbene formation rather than radical generation during the reaction process. Currently, initial efforts toward isolating α-P^V^ carbene and P/N hybrid ylide species have been unsuccessful. On the basis of the results herein and previous studies, a plausible mechanism for this cascade MCR is proposed (Fig. 2D). Initially, photo-activation of I^III^/P^V^ hybrid ylides generates ionic α-P^V^ carbene. Due to the inductive electron-withdrawing effect of the cationic phosphorus atom, this electrophilic Fischer carbene species would react with nitriles to afford the P/N hybrid ylide intermediate. Subsequent nucleophilic attack from the carboxylic anion to the nitrilium ion at the carbon would generate the second phosphonium ylide containing intermediates, which undergo an intramolecular Wittig olefination pathway to afford the tri-substituted oxazole products.

## Conclusions

In summary, we have developed a visible light driven three-component reaction through a programmed P/N hybrid ylide formation-carboxylic anion trapping-Wittig olefination cascade, providing expeditious access to tri-substituted oxazole fragments with high efficiency and remarkable chemoselectivity. A preliminary mechanistic study revealed several forms of phosphonium ylide, among which the P/N hybrid type species was conclusively proven to bridge the carbene reactivity and the electrophilicity towards carboxylic acid. The discovery of more multi-component reactions using such a strategy is currently in progress in our laboratory.

## Data availability

All experimental data are provided in the ESI.†

## Author contributions

X. C. Y. performed the experiments with contributions from H. J. P.; Z. F. W. conceived the study; Y. H., J. A. C. and Z. F. W. supervised the project; Z. F. W. wrote the manuscript with input from all authors. All authors have read and approved the final manuscript.

## Conflicts of interest

The authors declare no competing financial interest.

## Supplementary Material

SC-015-D4SC01355G-s001

SC-015-D4SC01355G-s002
