# Supplementary material for: Photochemical three-component assembly of tri-substituted oxazoles through a carbenic phosphorus-nitrile hybrid ylide formation/trapping cascade
Source: Chem Sci. 2024 Apr 5;15(17):6515–21. doi: 10.1039/d4sc01355g (PMC11062088; doi:10.1039/d4sc01355g)
Supplement: SC-015-D4SC01355G-s001 [file SC-015-D4SC01355G-s001.pdf]

Electronic Supplementary Material (ESI) for Chemical Science.
This journal is © The Royal Society of Chemistry 2024

Supporting Information

Photochemical three-component assembly of *tri*-substituted oxazoles through a carbenic phosphorus-nitrile hybrid ylide formation/trapping cascade

Xingchen Ye, Huaijin Pan, Yong Huang,* Jiean Chen* and Zhaofeng Wang*

*correspondence to: yonghuang@ust.hk; chenja@szbl.ac.cn; zfwangchem@hnu.edu.cn

Table of Contents

1. General Information S2
2. Synthesis of Substrates S3
    2.1 General procedure for preparation of hypervalent iodine substrates 1 .... S3
    2.2 Characterization of substrates S5
3. Synthesis of 2,4,5-trisubstituted oxazoles S12
    3.1 *General Procedure A* S12
    3.2 *General Procedure B* for condition optimization of solid nitrile S13
    3.3 Characterization of 2,4,5-trisubstituted oxazoles S14
    3.4 Late-stage functionalization of drugmolecules S41
4. Mechanistic Studies S48
    4.1 ^{18}O-isotope labeling S48
    4.2 Step-wise study S50
    4.3 Control experiment S51
    4.4 Gram-scale reaction S52
    4.5 UV-Visible absorption analysis S53
5. Supplementary Figures S54
6. References S141

1. General Information

Unless otherwise stated, all reactions were carried out under a nitrogen atmosphere with dry solvents under anhydrous conditions. Anhydrous acetonitrile purchased from Energy Chemical. All other reagents were purchased and used without further purification unless specified otherwise. Hypervalent iodine reagents were synthesized according to literature procedures[1] or modified procedures. Carboxylic acid derivatives were purchased from commercial suppliers. For chromatographic purification, 200-300 mesh silica gel (Yantai Xincheng Co., China., 200-300 mesh) was employed. For thin layer chromatography (TLC) analysis, high efficiency thin layer chromatography silica gel plates (HPTLC Silica Gel 60 GF254, 2.5*5.0 cm) were used. The ^{1}H NMR (400 MHz) chemical shifts were measured relative to tetramethylsilane or solvent residual of $CDCl_3$, DMSO-d_6 as an internal standard (TMS: δ = 0 ppm or $CDCl_3$: δ = 7.26 ppm, DMSO-d_6: δ = 2.50 ppm). The ^{13}C NMR (101 MHz) chemical shifts were given using $CDCl_3$ or DMSO-d_6 as the internal standard ($CDCl_3$: δ = 77.16 ppm, DMSO-d_6: δ = 39.52 ppm). High resolution mass spectra (HRMS) were recorded on an Agilent GC/MS 5975C system by Electrospray Ionisation (ESI), data were reported with ion mass/charge (m/z) ratios as values in atomic mass units.

2. Synthesis of Substrates

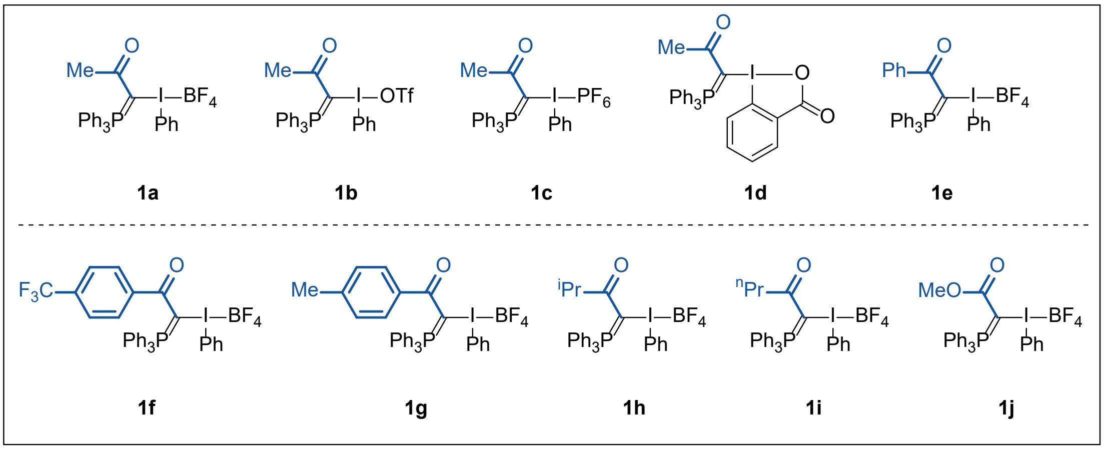

Supplementary Figure 1. Hypervalent iodine reagents **1a-1j**

2.1 General procedure for preparation of hypervalent iodine substrates 1

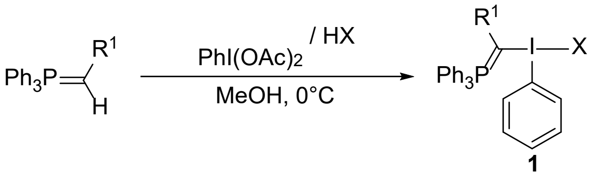

Substrates **1a, 1c, 1e, 1j** were prepared according to the literature procedures[1]. While stirring, the solution of $PhI(OAc)_2$ (1.61 g, 5 mmol) and HBF_4 / HPF_6 (5 mmol) in MeOH (5 mL) was added dropwise to the ice bath-cooled solution of ylide (5 mmol) in MeOH (5 mL) for 20 min. During the addition process, a large amount of precipitate was formed. After complete addition, the reaction mixture was stirred at 0 °C for 1.5 h. After filtration, the precipitate was washed with Et_2O (5 mL × 3), recrystallized from CH_2Cl_2-MeOH, and dried under vacuum to give corresponding substrates **1**.

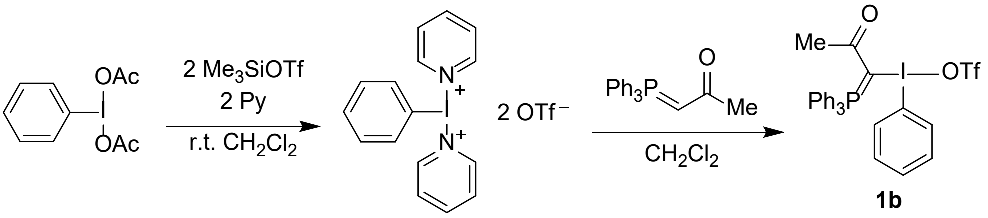

Substrate **1b** was prepared according to the literature procedures[3]. Firstly, $PhI(OAc)_2$ (0.81 g, 2.5 mmol) in CH_2Cl_2 (20 mL) was treated with Me_3SiOTf (1.11 g, 5 mmol),

and then pyridine (0.40 g, 5 mmol) in CH_2Cl_2 (10 mL) was added dropwise to this solution. The colorless precipitate was filtered, washed with CH_2Cl_2, and vacuum dried, to get the intermediate pyridinium complex of iodobenzene ditriflate. Then a solution of ylide (0.11 g, 0.335 mmol) in CH_2Cl_2 (3 mL) was added to a suspension of the intermediate (0.22 g, 0.335 mmol) under a nitrogen atmosphere at room temperature. The solution was additionally stirred for 3 h, washed several times with water, dried, and concentrated in a vacuum. Recrystallization of the residue from CH_2Cl_2/Et_2O got the substrate **1b** in the form of a white, microcrystalline solid.

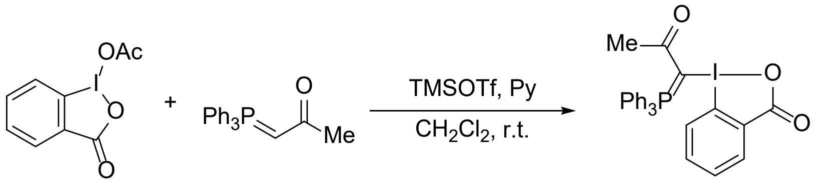

1d

The substrate **1d** was prepared according to the literature procedures[3]. A sample of 1-acetoxybenziodoxole (1.53 g, 5 mmol) was dissolved in dichloromethane (27 mL) and stirred under nitrogen for 30 min, then trimethylsilyl triflate (1.11 g, 5 mmol) was added, and after 10 min of stirring, a solution of pyridine (1.19 g, 15 mmol) in dichloromethane (4 mL) was added. The reaction mixture was additionally stirred for 2 h at room temperature. A solution of ylide 1-(triphenyl-λ^5-phosphanylidene)propan- 2-one (1.59 g, 5 mmol) in dichloromethane (0.8 mL) was then added to the reaction mixture. The mixture was stirred overnight at room temperature. The resulting clear solution was washed with distilled water and dried with anhydrous sodium sulfate. Solvents were removed in a vacuum to afford a colorless oil, which was recrystallized from dichloromethane and diethyl ether to get **1d** in the form of white crystals.

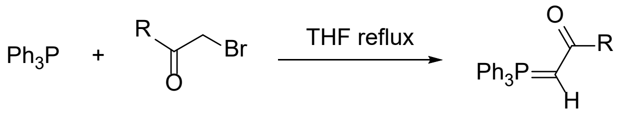

And the preparation of substrates **1f, 1g, 1h** require two steps. The first step, according to a reported protocol[2], aryl-substituted 2-bromoethanone (5 mmol) was added to a stirred solution of PPh_3 (1.44 g, 5.5 mmol) in THF (10 mL). Reflux the reaction mixture

for 4 h and then cooled to room temperature. Filter the solids and wash the solids with THF (20 mL × 3), then dry on the funnel. After transferring to a separatory funnel, add CH_2Cl_2 to dissolve solids and aqueous NaOH (20w%, 20 mL) were added and the mixture was shaken vigorously for 10 min. The phases were separated and the aqueous phase was extracted with CH_2Cl_2 (10 mL × 3). The combined organic layers were dried over anhydrous Na_2SO_4, filtered and concentrated under reduced pressure to yield the corresponding phosphonium ylide. Then through the procedures of substrates **1a, 1c, 1e, 1j** got the corresponding substrates.

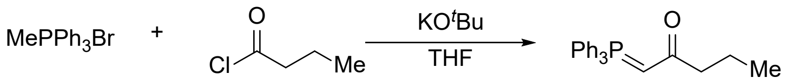

The preparation of substrates **1i** requires two steps. The first step, according to a procedure of R. Brückner et al.[4], KO*t*Bu (0.56 g, 5 mmol) was added to the THF (15 mL) solution of $MePPh_3Br$ (3.57 g, 10 mmol) cooled in an ice bath and stirred for 30 minutes. Then butyryl chloride (0.53 g, 5 mmol) was added and the mixture was stirred at room temperature for 3 h. Then, 35 mL of H_2O were added and the aqueous phase was extracted three times with 25 mL of M*t*BE. The organic phase was dried over $MgSO_4$ and the solvent was removed under reduced pressure to obtain the corresponding phosphonium ylide. Then through the literature procedures[1] got the corresponding substrate **1i**.

2.2 Characterization of substrates

(2-oxo-1-(triphenyl-λ^5-phosphanylidene)propyl)(phenyl)iodonium tetrafluoroborate (1a)

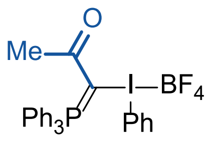

Prepared according to the literature procedures[1] using phenyliodoso diacetate (1.61 g, 5.0 mmol), HBF_4 (0.5 ml, 48% w/w, 5.0 mmol), 1-(triphenyl-λ^5-phosphanylidene)

propan-2-one (1.60 g, 5.0 mmol). After filtration, **1a** was collected as a white solid (2.5 g, 83% yield).

^{1}H NMR (400 MHz, DMSO-d_6) δ 7.74 (t, J = 7.6 Hz, 4H), 7.64 – 7.56 (m, 10H), 7.53 – 7.46 (m, 6H), 2.58 (s, 3H).

^{13}C NMR (101 MHz, DMSO-d_6) δ 192.2, 133.6, 133.5, 133.4, 132.2, 131.2, 131.1, 129.3, 129.2, 124.2, 123.2, 26.9.

^{19}F NMR (376 MHz, DMSO-d_6) δ -148.27 (d, J = 20.4 Hz).

^{31}P NMR (162 MHz, DMSO-d_6) δ 26.16.

^{11}B NMR (128 MHz, DMSO-d_6) δ -1.28.

HRMS (ESI-TOF) $[M\text{-}BF_4]^+$ calculated for $[C_{27}H_{23}IOP]^+$ m/z: 521.0526, found 521.0522.

(2-oxo-1-(triphenyl-l5-phosphanylidene)propyl)(phenyl)iodonium trifluoromethanesulfonate (1b)

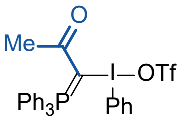

Prepared according to the literature procedures[3] using phenyliodoso diacetate (0.80 g, 2.5 mmol), Me_3SiOTf (1.11 g, 5 mmol), pyridine (0.40 g, 5 mmol), the intermediate can be obtained in 72% yield. Then 1,1'-(phenyl-l3-iodanediyl)bis(pyridin-1-ium) (0.72 g, 2.0 mmol), 1-(triphenyl-λ^5-phosphanylidene)propan-2-one (0.64 g, 2.0 mmol). After filtration, **1b** was collected as a white solid (1.06 g, 79% yield).

^{1}H NMR (400 MHz, DMSO-d_6) δ 7.73 (t, J = 7.5 Hz, 3H), 7.64 – 7.56 (m, 10H), 7.55 – 7.46 (m, 7H), 2.60 (s, 3H).

^{13}C NMR (101 MHz, DMSO-d_6) δ 192.0, 133.6 – 133.3 (m), 132.2, 131.2, 131.1, 129.3, 129.2, 124.2, 123.2, 119.4, 119.2, 26.9.

^{19}F NMR (376 MHz, DMSO-d_6) δ -77.63.

^{31}P NMR (162 MHz, DMSO-d_6) δ 26.19.

HRMS (ESI-TOF) $[M\text{-}OTf]^+$ calculated for $[C_{27}H_{23}IOP]^+$ m/z: 521.0526, found 521.0523.

(2-oxo-1-(triphenyl-l5-phosphanylidene)propyl)(phenyl)iodonium hexafluorophosphate (1c)

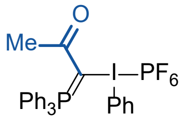

Prepared according to the literature procedures[1] using phenyliodoso diacetate (1.61 g, 5.0 mmol), HPF_6 (0.74 ml, 60% w/w, 5.0 mmol), 1-(triphenyl-λ^5-phosphanylidene) propan-2-one (1.60 g, 5.0 mmol). After filtration, **1c** was collected as a white solid (2.6 g, 78% yield).

^{1}H NMR (400 MHz, DMSO-d_6) δ 7.74 (t, J = 7.5 Hz, 3H), 7.63 – 7.57 (m, 9H), 7.54 – 7.43 (m, 8H), 2.59 (s, 3H).

^{13}C NMR (101 MHz, DMSO-d_6) δ 192.0, 133.6, 133.5, 132.2, 131.2, 131.1, 129.4, 129.2, 124.2, 123.3, 26.9.

^{19}F NMR (376 MHz, DMSO-d_6) δ -70.16 (d, J = 711 Hz).

^{31}P NMR (162 MHz, DMSO-d_6) δ 26.18, -144.15 (h, J = 711 Hz).

HRMS (ESI-TOF) $[M-PF_6]^+$ calculated for $[C_{27}H_{23}IOP]^+$ m/z: 521.0526, found 521.0523.

2-((2-oxo-1-(triphenyl-λ^5-phosphanylidene)propyl)iodonio)benzoate (1d)

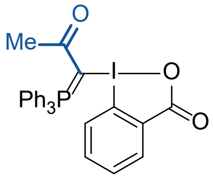

Prepared according to the literature procedures[3] using 1-acetoxybenziodoxole (1.53 g, 5 mmol), trimethylsilyl triflate (1.11 g, 5 mmol), pyridine (1.19 g, 15 mmol), 1-(triphenyl-λ^5-phosphanylidene)propan-2-one (1.59 g, 5 mmol). After filtration, **1d** was collected as a white solid (2.31 g, 82% yield).

^{1}H NMR (400 MHz, DMSO-d_6) δ 7.89 – 7.82 (m, 3H), 7.81 – 7.51 (m, 16H), 2.32 (s, 3H).

^{13}C NMR (101 MHz, Acetone-d_6) δ 207.4, 202.1, 136.8, 136.8, 135.8, 135.7, 132.1, 132.0, 124.9, 121.7, 121.4, 120.5, 118.5, 32.9.

^{31}P NMR (162 MHz, DMSO-d_6) δ 19.44.

HRMS (ESI-TOF) $[M+H]^+$ calculated for $[C_{28}H_{22}IO_3P]^+$ m/z: 565.0424, found 565.0424.

(2-oxo-2-phenyl-1-(triphenyl-λ⁵-phosphanylidene)ethyl)(phenyl)iodonium tetrafluoroborate (1e)

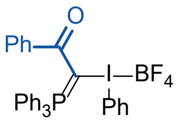

Prepared according to the literature procedures[1] using phenyliodoso diacetate (1.61 g, 5.0 mmol), HBF_4 (0.5 ml, 48% w/w, 5.0 mmol), 1-phenyl-2-(triphenyl-λ^5-phosphanylidene)ethan-1-one (1.90 g, 5.0 mmol). After filtration, **1e** was collected as a white solid (2.68 g, 80% yield).

^{1}H NMR (400 MHz, DMSO-*d*6) δ 7.81 – 7.74 (m, 3H), 7.71 (d, *J* = 5.6 Hz, 2H), 7.68 – 7.56 (m, 13H), 7.52 (s, 3H), 7.42 (t, *J* = 7.8 Hz, 2H), 7.28 (d, *J* = 7.8 Hz, 2H).

^{13}C NMR (101 MHz, DMSO-*d*6) δ 191.11, 140.00, 139.92, 133.66, 133.59, 133.56, 132.04, 131.39, 131.27, 130.15, 129.46, 129.33, 128.22, 127.57, 123.76, 122.84, 119.40, 119.37.

^{19}F NMR (376 MHz, DMSO-*d*6) δ -148.26, -148.31.

^{31}P NMR (162 MHz, DMSO-*d*6) δ 26.05.

^{11}B NMR (128 MHz, DMSO-*d*6) δ -1.27.

HRMS (ESI-TOF) $[[M-BF_4]^+$ calculated for $[C_{32}H_{25}IOP]^+$ m/z: 583.0682, found 583.0679.

(2-oxo-2-(4-(trifluoromethyl)phenyl)-1-(triphenyl-λ⁵-phosphanylidene)ethyl)(phenyl)iodonium tetrafluoroborate (1f)

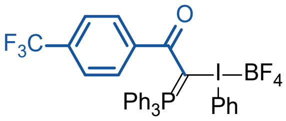

Prepared according to the literature procedures[1,2] using 2-bromo-1-(4-(trifluoromethyl) phenyl)ethan-1-one (1.33 g, 5.0 mmol), PPh_3 (1.31 g, 5.0 mmol), phenyliodoso diacetate (1.61 g, 5.0 mmol), HBF_4 (0.5 ml, 48% w/w, 5.0 mmol). After filtration, **1f** was collected as a white solid (2.29 g, 62% yield).

^{1}H NMR (400 MHz, DMSO-*d*$_6$) δ 7.93 (s, 4H), 7.78 (dd, J = 8.4, 5.2 Hz, 3H), 7.71 – 7.58 (m, 13H), 7.43 (t, J = 7.7 Hz, 2H), 7.32 (d, J = 7.9 Hz, 2H).

^{13}C NMR (101 MHz, DMSO-*d*$_6$) δ 189.8, 144.2, 133.7, 133.6, 132.0, 131.5, 131.4, 130.2, 129.9, 129.5, 129.4, 128.3, 125.3, 125.3, 123.4, 122.6, 122.4, 119.2.

^{19}F NMR (376 MHz, DMSO-*d*$_6$) δ -61.15, -148.34 (d, J = 20.1 Hz).

^{31}P NMR (162 MHz, DMSO-*d*$_6$) δ 26.07.

^{11}B NMR (128 MHz, DMSO-*d*$_6$) δ -1.27.

HRMS (ESI-TOF) $[M-BF_4]^+$ calculated for $[C_{33}H_{24}F_3IOP]^+$ m/z: 651.0556, found 651.0551.

(2-oxo-2-(*p*-tolyl)-1-(triphenyl-λ^5-phosphanylidene)ethyl)(phenyl)iodonium tetrafluoroborate (1g)

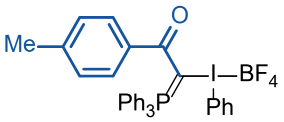

Prepared according to the literature procedures[1,2] using 2-bromo-1-(p-tolyl)ethan -1-one (1.06 g, 5.0 mmol), PPh_3 (1.31 g, 5.0 mmol), phenyliodoso diacetate (1.61 g, 5.0 mmol), HBF_4 (0.5 ml, 48% w/w, 5.0 mmol). After filtration, **1g** was collected as a white solid (2.26 g, 66% yield).

^{1}H NMR (400 MHz, DMSO-*d*$_6$) δ 7.76 (t, J = 6.9 Hz, 3H), 7.68 – 7.54 (m, 15H), 7.43 (t, J = 7.7 Hz, 2H), 7.31 (dd, J = 11.8, 7.7 Hz, 4H), 2.38 (s, 3H).

^{13}C NMR (101 MHz, DMSO-*d*$_6$) δ 191.1, 140.0, 137.1, 133.7, 133.5, 132.0, 131.3, 129.4, 129.3, 128.7, 127.7, 123.9, 123.0, 119.5, 21.0.

^{19}F NMR (376 MHz, DMSO-*d*$_6$) δ -148.29 (d, J = 19.8 Hz).

^{31}P NMR (162 MHz, DMSO-*d*$_6$) δ 26.09.

^{11}B NMR (128 MHz, DMSO-*d*$_6$) δ -1.27.

HRMS (ESI-TOF) $[M-BF_4]^+$ calculated for $[C_{33}H_{27}IOP]^+$ m/z: 597.0839, found 597.0836.

(3-methyl-2-oxo-1-(triphenyl-λ^5-phosphanylidene)butyl)(phenyl)iodonium tetrafluoroborate (1h)

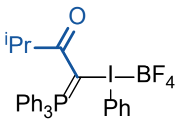

Prepared according to the literature procedures[1,2] using 1-bromo-3-methylbutan-2-one (0.82 g, 5.0 mmol), PPh_3 (1.31 g, 5.0 mmol), phenyliodoso diacetate (1.61 g, 5.0 mmol), HBF_4 (0.5 ml, 48% w/w, 5.0 mmol). After filtration, **1h** was collected as a white solid (2.0 g, 63% yield).

^{1}H NMR (400 MHz, DMSO-*d*6) δ 7.74 (t, *J* = 7.8 Hz, 4H), 7.64 – 7.58 (m, 8H), 7.56 – 7.48 (m, 8H), 3.57 (h, *J* = 6.6 Hz, 1H), 1.04 (d, *J* = 6.8 Hz, 6H).

^{13}C NMR (101 MHz, DMSO-*d*6) δ 198.1, 133.5, 133.4, 131.8, 131.3, 131.2, 129.3, 129.2, 124.3, 123.4, 34.1, 19.8.

^{19}F NMR (376 MHz, DMSO-*d*6) δ -148.30 (d, *J* = 20.5 Hz).

^{31}P NMR (162 MHz, DMSO-*d*6) δ 25.78.

^{11}B NMR (128 MHz, DMSO-*d*6) δ -1.27.

HRMS (ESI-TOF) $[M-BF_4]^+$ calculated for $[C_{29}H_{27}IOP]^+$ m/z: 549.0839, found 549.0838.

(2-oxo-1-(triphenyl-l5-phosphanylidene)pentyl)(phenyl)iodonium tetrafluoroborate (1i)

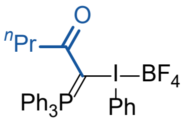

Prepared according to the literature procedures[1,4] using KO*t*Bu (0.56 g, 5 mmol), $MePPh_3Br$ (3.57 g, 10 mmol), butyryl chloride (0.53 g, 5 mmol), phenyliodoso diacetate (1.61 g, 5.0 mmol), HBF_4 (0.5 ml, 48% w/w, 5.0 mmol). After filtration, **1i** was collected as a white solid (2.13 g, 67% yield).

^{1}H NMR (400 MHz, DMSO-*d*6) δ 7.73 (d, *J* = 7.5 Hz, 3H), 7.63 – 7.56 (m, 10H), 7.53 – 7.45 (m, 7H), 3.04 – 2.70 (m, 2H), 1.54 (h, *J* = 6.9 Hz, 2H), 0.90 (t, *J* = 7.4 Hz, 3H).

^{13}C NMR (101 MHz, DMSO-*d*6) δ 193.94, 133.55, 133.45, 132.01, 131.23, 131.13, 129.32, 129.19, 124.27, 123.35, 42.87, 18.83, 13.68.

^{19}F NMR (376 MHz, DMSO-*d*6) δ -148.30 (d, *J* = 20.3 Hz).

^{31}P NMR (162 MHz, DMSO-*d*6) δ 26.05.

^{11}**B NMR** (128 MHz, DMSO-*d*$_6$) *δ* -1.27.

HRMS (ESI-TOF) $[M-BF_4]^+$ calculated for $[C_{29}H_{27}IOP]^+$ m/z: 549.0839, found 549.0837.

(2-methoxy-2-oxo-1-(triphenyl-l5-phosphanylidene)ethyl)(phenyl)iodonium tetrafluoroborate (1j)

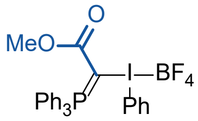

Prepared according to the literature procedures[1] using phenyliodoso diacetate (1.61 g, 5.0 mmol), HBF_4 (0.5 ml, 48% w/w, 5.0 mmol), methyl 2-(triphenyl-λ^5-phosphanylidene)acetate (1.67 g, 5.0 mmol). After filtration, **1j** was collected as a white solid (2.5 g, 83% yield).

^{1}**H NMR** (400 MHz, DMSO-*d*$_6$) *δ* 7.78 (t, *J* = 7.5 Hz, 3H), 7.68 – 7.60 (m, 7H), 7.59 – 7.51 (m, 8H), 7.47 (t, *J* = 7.7 Hz, 2H), 3.34 (s, 3H).

^{13}**C NMR** (101 MHz, DMSO-*d*$_6$) *δ* 190.8, 167.7, 167.5, 133.6, 133.5, 132.6, 131.3, 131.1, 129.5, 129.3, 52.1.

^{19}**F NMR** (376 MHz, DMSO-*d*$_6$) *δ* -148.31 (d, *J* = 20.3 Hz).

^{31}**P NMR** (162 MHz, DMSO-*d*$_6$) *δ* 28.10.

^{11}**B NMR** (128 MHz, DMSO-*d*$_6$) *δ* -1.27.

HRMS (ESI-TOF) $[M-BF_4]^+$ calculated for $[C_{27}H_{23}IO_2P]^+$ m/z: 537.0475, found 537.0470.

3. Synthesis of 2,4,5-trisubstituted oxazoles

3.1 *General Procedure A*

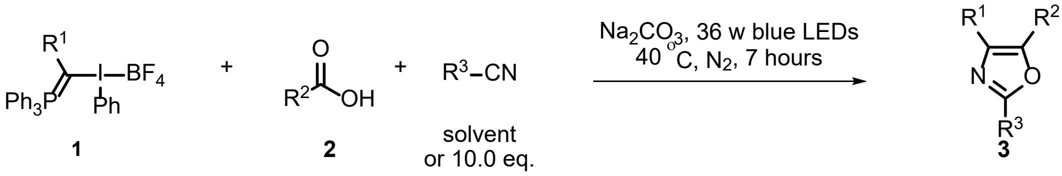

A dry clean test tube equipped with a stir bar was charged with the carboxylic acid derivative **2** (0.12 mmol), the corresponding base (0.24 mmol), and the hypervalent iodine **1** (0.1 mmol). And the cyano derivatives (1.0 mL) were added in a syringe (if liquid) under a nitrogen atmosphere. The resulting mixture was stirred for 7 h at 40 °C (water bath temperature) under blue light (λ_{max} = 438 nm) at 36 W. After this time, the tube was cooled to room temperature. After removing the volatiles under reduced pressure, the residue was then purified by column chromatography on silica gel (Petroleum ether / Ethyl acetate) to afford the desired product **3**. If the cyano derivative is solid, the corresponding product will be obtained using 1.0 mmol of cyano derivatives and 1.0 ml of 1,2-dichloroethane as solvent.

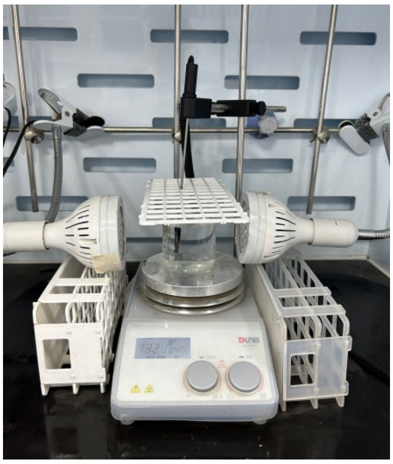

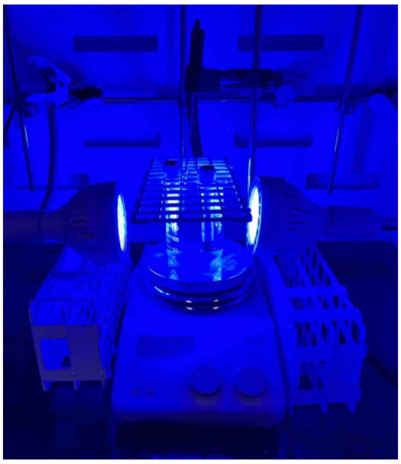

Supplementary Figure 2. Experimental setup for photochemical reaction

3.2 *General Procedure B* for condition optimization of solid nitrile

A dry clean test tube equipped with a stir bar was charged with benzoic acid (0.12 mmol), the hypervalent iodine **1a** (0.1 mmol), and cyanobenzene (1.0 mmol), and different bases (0.24 mmol) and catalysts (5 mol%). Then 1,2-dichloroethane (1.0 mL) was added in a syringe under a nitrogen atmosphere. The resulting mixture was stirred for 7 h at 40 °C (water bath temperature) under blue light at 36W. After removing the volatiles under reduced pressure, the residue was then purified by column chromatography on silica gel (Petroleum ether / Ethyl acetate) to afford the desired product **3aq**. The optimum conditions of the reaction were obtained by these yields.

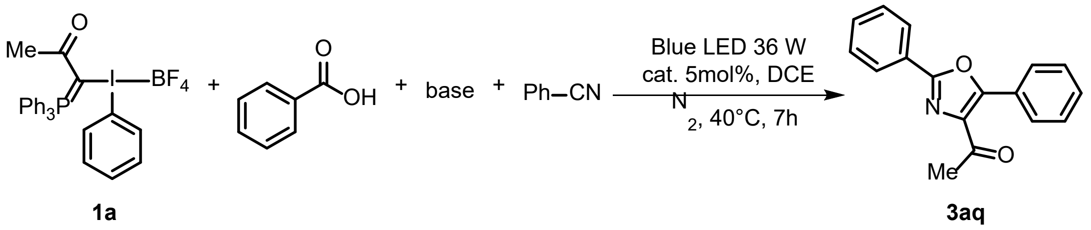

Supplementary Table 1.

entry	base	cat.	yield
1	Cs_2CO_3	/	12%
2	Na_2CO_3	/	15%
3	CsF	/	trace
4	Na_2CO_3	$(Ph_3P)AuCl$	54%
5	Na_2CO_3	$(CH_3)_2S(AuCl)$	24%
6	Na_2CO_3	Cat. A	27%

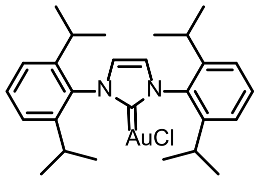

Cat. A

3.3 Characterization of 2,4,5-trisubstituted oxazoles

1-(2-methyl-5-(*p*-tolyl)oxazol-4-yl)ethan-1-one (3a）

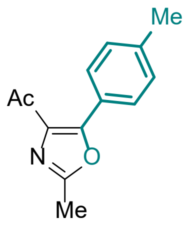

This compound was synthesized following the *general procedure A* using reagent **1a** (60.8 mg, 0.1 mmol), *p*-toluic acid (16.4 mg, 0.12 mmol), sodium carbonate (25.4 mg, 0.24 mmol), acetonitrile (1.0 ml). After flash column chromatography, eluting with Petroleum ether / Ethyl acetate mixtures, **3a** was collected as a white solid (15.9 mg, 74% yield).

^{1}H NMR (400 MHz, $CDCl_3$) δ 8.04 (d, J = 8.4 Hz, 2H), 7.25 (d, J = 8.4 Hz, 2H), 2.60 (s, 3H), 2.53 (s, 3H), 2.40 (s, 3H).

^{13}C NMR (101 MHz, $CDCl_3$) δ 194.3, 158.6, 153.7, 140.8, 134.0, 129.3, 128.0, 124.6, 29.1, 21.6, 13.9.

HRMS (ESI-TOF) $[M+H]^+$ calculated for $[C_{13}H_{14}NO_2]^+$ m/z: 216.1019, found 216.1020.

1-(5-(4-fluorophenyl)-2-methyloxazol-4-yl)ethan-1-one (3b)

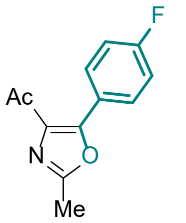

This compound was synthesized following the *general procedure A* using reagent **1a** (60.8 mg, 0.1 mmol), 4-fluorobenzoic acid (16.8 mg, 0.12 mmol), cesium carbonate (78.2 mg, 0.24 mmol), acetonitrile (1.0 ml). After flash column chromatography, eluting with Petroleum ether / Ethyl acetate mixtures, **3b** was collected as a white solid (14.0 mg, 64% yield).

^{1}H NMR (400 MHz, $CDCl_3$) δ 8.19 (dd, J = 9.0, 5.4 Hz, 2H), 7.20 – 7.06 (m, 2H), 2.61 (s, 3H), 2.54 (s, 3H).

^{13}C NMR (101 MHz, $CDCl_3$) δ 194.5, 165.2, 162.7, 158.8, 152.5, 130.3 (d, J = 8.5 Hz), 123.6 (d, J = 3.3 Hz), 115.7 (d, J = 21.8 Hz), 29.1, 13.9.

^{19}F NMR (376 MHz, $CDCl_3$) δ -109.12.

HRMS (ESI-TOF) $[M+H]^+$ calculated for $[C_{12}H_{11}FNO_2]^+$ m/z: 220.0768, found 220.0769.

1-(5-(4-chlorophenyl)-2-methyloxazol-4-yl)ethan-1-one (3c)

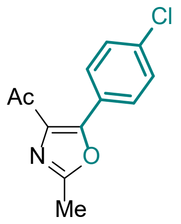

This compound was synthesized following the *general procedure A* using reagent **1a** (60.8 mg, 0.1 mmol), 4-chlorobenzoic acid (18.8 mg, 0.12 mmol), cesium carbonate (78.2 mg, 0.24 mmol), acetonitrile (1.0 ml). After flash column chromatography, eluting with Petroleum ether / Ethyl acetate mixtures, **3c** was collected as a white solid (16.5 mg, 70% yield).

^{1}H NMR (400 MHz, $CDCl_3$) δ 8.13 (d, J = 8.8 Hz, 2H), 7.41 (d, J = 8.8 Hz, 2H), 2.60 (s, 3H), 2.54 (s, 3H).

^{13}C NMR (101 MHz, $CDCl_3$) δ 194.5, 159.0, 152.3, 136.4, 134.7, 129.3, 128.8, 125.8, 29.2, 13.9.

HRMS (ESI-TOF) $[M+H]^+$ calculated for $[C_{12}H_{11}ClNO_2]^+$ m/z: 236.0473, found 236.0473.

1-(5-(4-bromophenyl)-2-methyloxazol-4-yl)ethan-1-one (3d)

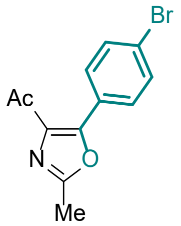

This compound was synthesized following the *general procedure A* using reagent **1a** (60.8 mg, 0.1 mmol), 4-bromobenzoic acid (24.1 mg, 0.12 mmol), cesium carbonate (78.2 mg, 0.24 mmol), acetonitrile (1.0 ml). After flash column chromatography, eluting with Petroleum ether / Ethyl acetate mixtures, **3d** was collected as a white solid (20.4 mg, 73% yield).

^{1}H NMR (400 MHz, $CDCl_3$) δ 8.05 (d, J = 8.8 Hz, 2H), 7.57 (d, J = 8.8 Hz, 2H), 2.60 (s, 3H), 2.54 (s, 3H).

^{13}C NMR (101 MHz, $CDCl_3$) δ 194.5, 159.1, 152.3, 134.7, 131.8, 129.5, 126.2, 124.8, 29.2, 13.9.

HRMS (ESI-TOF) $[M+H]^+$ calculated for $[C_{12}H_{11}BrNO_2]^+$ m/z: 279.9968, found 279.9972.

1-(5-(4-iodophenyl)-2-methyloxazol-4-yl)ethan-1-one (3e)

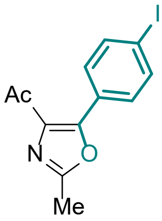

This compound was synthesized following the *general procedure A* using reagent **1a** (60.8 mg, 0.1 mmol), 4-iodobenzoic acid (29.8 mg, 0.12 mmol), cesium carbonate (78.2 mg, 0.24 mmol), acetonitrile (1.0 ml). After flash column chromatography, eluting with Petroleum ether / Ethyl acetate mixtures, **3e** was collected as a white solid (23.5 mg, 72% yield).

^{1}H NMR (400 MHz, $CDCl_3$) δ 7.90 (d, J = 8.8 Hz, 1H), 7.78 (d, J = 8.8 Hz, 1H), 2.60 (s, 2H), 2.54 (s, 2H).

^{13}C NMR (101 MHz, $CDCl_3$) δ 194.5, 159.1, 152.4, 137.8, 134.9, 129.5, 126.8, 97.0, 29.2, 13.9.

HRMS (ESI-TOF) $[M+H]^+$ calculated for $[C_{12}H_{11}BrNO_2]^+$ m/z: 327.9829, found 327.9828.

4-(4-acetyl-2-methyloxazol-5-yl)benzonitrile (3f)

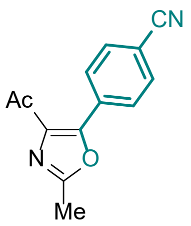

This compound was synthesized following the *general procedure A* using reagent **1a** (60.8 mg, 0.1 mmol), 4-cyanobenzoic acid (17.7 mg, 0.12 mmol), cesium carbonate (78.2 mg, 0.24 mmol), acetonitrile (1.0 ml). After flash column chromatography, eluting with Petroleum ether / Ethyl acetate mixtures, **3f** was collected as a white solid (17.0 mg, 75% yield).

^{1}H NMR (400 MHz, $CDCl_3$) δ 8.31 (d, J = 8.4 Hz, 2H), 7.72 (d, J = 8.4 Hz, 2H), 2.63 (s, 3H), 2.57 (s, 3H).

^{13}C NMR (101 MHz, $CDCl_3$) δ 194.6, 160.1, 150.9, 136.2, 132.3, 131.3, 128.3, 118.5, 113.5, 29.2, 14.0.

HRMS (ESI-TOF) $[M+H]^+$ calculated for $[C_{13}H_{11}N_2O_2]^+$ m/z: 227.0815, found 227.0814.

1-(2-methyl-5-(4-nitrophenyl)oxazol-4-yl)ethan-1-one (3g)

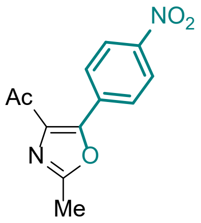

This compound was synthesized following the *general procedure A* using reagent **1a** (60.8 mg, 0.1 mmol), p-nitrobenzoic acid (20.1 mg, 0.12 mmol), cesium carbonate (78.2 mg, 0.24 mmol), acetonitrile (1.0 ml). After flash column chromatography, eluting with Petroleum ether / Ethyl acetate mixtures, **3g** was collected as a white solid (19.9 mg, 81% yield).

^{1}H NMR (400 MHz, $CDCl_3$) δ 8.39 (d, J = 8.8 Hz, 1H), 8.28 (d, J = 8.8 Hz, 1H), 2.65 (s, 2H), 2.59 (s, 2H).

^{13}C NMR (101 MHz, $CDCl_3$) δ 194.7, 160.4, 150.5, 148.4, 136.5, 133.1, 131.1, 128.7, 123.8, 29.2, 14.0.

HRMS (ESI-TOF) $[M+H]^+$ calculated for $[C_{12}H_{11}N_2O_4]^+$ m/z: 247.0713, found 247.0713.

1-(2-methyl-5-(4-(trifluoromethyl)phenyl)oxazol-4-yl)ethan-1-one (3h)

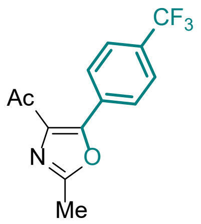

This compound was synthesized following the *general procedure A* using reagent **1a** (60.8 mg, 0.1 mmol), 4-(trifluoromethyl)benzoic acid (22.8 mg, 0.12 mmol), cesium carbonate (78.2 mg, 0.24 mmol), acetonitrile (1.0 ml). After flash column chromatography, eluting with Petroleum ether / Ethyl acetate mixtures, **3h** was collected as a white solid (22.1 mg, 82% yield).

^{1}H NMR (400 MHz, $CDCl_3$) δ 8.29 (d, J = 8.4 Hz, 2H), 7.70 (d, J = 8.4 Hz, 2H), 2.64 (s, 3H), 2.58 (s, 3H).

^{13}C NMR (101 MHz, $CDCl_3$) δ 194.6, 159.7, 151.6, 135.6, 131.9 (d, J = 32.5 Hz), 130.6, 128.3, 125.5 (q, J = 3.8 Hz), 125.3, 29.2, 14.0.

^{19}F NMR (376 MHz, $CDCl_3$) δ -62.98.

HRMS (ESI-TOF) $[M+H]^+$ calculated for $[C_{13}H_{11}F_3NO_2]^+$ m/z: 270.0736, found 270.0737.

methyl 4-(4-acetyl-2-methyloxazol-5-yl)benzoate (3i)

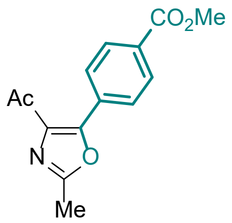

This compound was synthesized following the *general procedure A* using reagent **1a** (60.8 mg, 0.1 mmol), mono-methyl terephthalate (21.6 mg, 0.12 mmol), cesium carbonate (78.2 mg, 0.24 mmol), acetonitrile (1.0 ml). After flash column

chromatography, eluting with Petroleum ether / Ethyl acetate mixtures, **3i** was collected as a white solid (23.3 mg, 90% yield).

^{1}H NMR (400 MHz, $CDCl_3$) δ 8.24 (d, J = 8.8 Hz, 2H), 8.10 (d, J = 8.8 Hz, 2H), 3.93 (s, 3H), 2.62 (s, 3H), 2.56 (s, 3H).

^{13}C NMR (101 MHz, $CDCl_3$) δ 194.5, 166.6, 159.6, 152.0, 135.6, 131.4, 131.3, 129.8, 127.8, 52.4, 29.2, 14.0.

HRMS (ESI-TOF) $[M+Na]^+$ calculated for $[C_{14}H_{13}NO_4Na]^+$ m/z: 282.0737, found 282.0732.

1-(2-methyl-5-phenyloxazol-4-yl)ethan-1-one (3j)

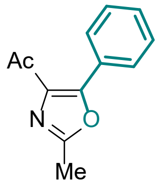

This compound was synthesized following the *general procedure A* using reagent **1a** (60.8 mg, 0.1 mmol), benzoic acid (14.7 mg, 0.12 mmol), cesium carbonate (78.2 mg, 0.24 mmol), acetonitrile (1.0 ml). After flash column chromatography, eluting with Petroleum ether / Ethyl acetate mixtures, **3j** was collected as a white solid (12.9 mg, 64% yield).

^{1}H NMR (400 MHz, $CDCl_3$) δ 8.14 (d, J = 8.0 Hz, 2H), 7.55 – 7.37 (m, 3H), 2.60 (s, 3H), 2.54 (s, 3H).

^{13}C NMR (101 MHz, $CDCl_3$) δ 194.4, 158.9, 153.4, 134.4, 130.5, 128.5, 128.0, 127.4, 29.2, 13.9.

HRMS (ESI-TOF) $[M+H]^+$ calculated for $[C_{12}H_{12}NO_2]^+$ m/z: 202.0863, found 202.0864.

The crystal structure of **3j** has been deposited at the Cambridge Crystallographic Data Centre, CCDC2166324.

***tert*-butyl (4-(4-acetyl-2-methyloxazol-5-yl)phenyl)-l2-azanecarboxylate (3k)**

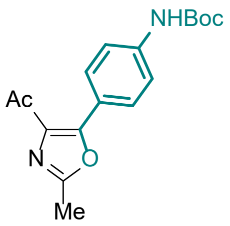

This compound was synthesized following the *general procedure A* using reagent **1a** (60.8 mg, 0.1 mmol), Boc-4-Abz-OH(28.5 mg, 0.12 mmol), caesium fluoride (36.5 mg, 0.24 mmol), acetonitrile (1.0 ml). After flash column chromatography, eluting with Petroleum ether / Ethyl acetate mixtures, **3k** was collected as a white solid (23.7 mg, 75% yield).

^{1}H NMR (400 MHz, $CDCl_3$) δ 8.14 (d, J = 8.4 Hz, 2H), 7.47 (d, J = 8.4 Hz, 2H), 6.77 (s, 1H), 2.60 (s, 3H), 2.53 (s, 3H), 1.53 (s, 9H).

^{13}C NMR (101 MHz, $CDCl_3$) δ 194.5, 158.4, 153.5, 152.5, 140.5, 133.7, 129.1, 121.9, 117.9, 81.1, 29.2, 28.5, 13.9.

HRMS (ESI-TOF) $[M+H]^+$ calculated for $[C_{17}H_{21}N_2O_4]^+$ m/z: 317.1496, found 317.1498.

1-(5-([1,1'-biphenyl]-4-yl)-2-methyloxazol-4-yl)ethan-1-one (3l)

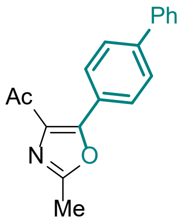

This compound was synthesized following the *general procedure A* using reagent **1a** (60.8 mg, 0.1 mmol), 4-biphenylcarboxylic acid(23.8 mg, 0.12 mmol), sodium carbonate (25.4 mg, 0.24 mmol), acetonitrile (1.0 ml). After flash column chromatography, eluting with Petroleum ether / Ethyl acetate mixtures, **3l** was collected as a white solid (17.5 mg, 63% yield).

^{1}H NMR (400 MHz, $CDCl_3$) δ 8.25 (d, J = 6.4 Hz, 2H), 7.69 (d, J = 6.4 Hz, 2H), 7.65 (d, J = 5.6 Hz, 2H), 7.52 – 7.42 (m, 2H), 7.39 (dd, J = 7.2, 4.4 Hz, 1H), 2.64 (s, 3H), 2.57 (s, 3H).

^{13}**C NMR** (101 MHz, $CDCl_3$) δ 194.5, 158.9, 153.2, 143.1, 140.4, 134.5, 129.0, 128.5, 128.0, 127.3, 127.2, 126.3, 29.2, 14.0.

HRMS (ESI-TOF) $[M+H]^+$ calculated for $[C_{18}H_{16}NO_2]^+$ m/z: 278.1176, found 278.1178.

1-(2-methyl-5-(4-(methylthio)phenyl)oxazol-4-yl)ethan-1-one (3m)

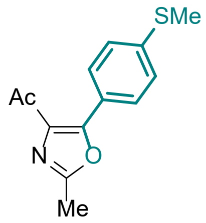

This compound was synthesized following the *general procedure A* using reagent **1a** (60.8 mg, 0.1 mmol), 4-(methylthio)benzoic acid (20.2 mg, 0.12 mmol), cesium carbonate (78.2 mg, 0.24 mmol), acetonitrile (1.0 ml). After flash column chromatography, eluting with Petroleum ether / Ethyl acetate mixtures, **3m** was collected as a white solid (13.3 mg, 54% yield).

^{1}**H NMR** (400 MHz, $CDCl_3$) δ 8.11 (d, J = 8.4 Hz, 2H), 7.29 (d, J = 8.2 Hz, 2H), 2.61 (s, 3H), 2.54 (s, 3H), 2.52 (s, 3H).

^{13}**C NMR** (101 MHz, $CDCl_3$) δ 194.4, 158.6, 153.2, 142.1, 134.1, 128.8, 128.2, 125.6, 123.7, 29.2, 15.2, 13.9.

HRMS (ESI-TOF) $[M+H]^+$ calculated for $[C_{13}H_{14}NO_2S]^+$ m/z: 248.0740, found 248.0740.

1-(5-(4-methoxyphenyl)-2-methyloxazol-4-yl)ethan-1-one (3n)

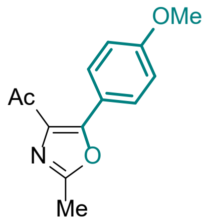

This compound was synthesized following the *general procedure A* using reagent **1a** (60.8 mg, 0.1 mmol), 4-methoxybenzoic acid (18.3 mg, 0.12 mmol), cesium carbonate (78.2 mg, 0.24 mmol), acetonitrile (1.0 ml). After flash column chromatography,

eluting with Petroleum ether / Ethyl acetate mixtures, **3n** was collected as a white solid (17.1 mg, 74% yield).

^{1}H NMR (400 MHz, $CDCl_3$) δ 8.15 (d, J = 8.8 Hz, 2H), 6.96 (d, J = 8.8 Hz, 2H), 3.86 (s, 3H), 2.59 (s, 3H), 2.52 (s, 3H).

^{13}C NMR (101 MHz, $CDCl_3$) δ 194.3, 161.4, 158.2, 153.7, 133.4, 132.7, 129.8, 120.0, 114.0, 55.5, 29.1, 13.9.

HRMS (ESI-TOF) $[M+H]^+$ calculated for $[C_{13}H_{14}NO_3]^+$ m/z: 232.0968, found 232.0972.

1-(5-(4-ethynylphenyl)-2-methyloxazol-4-yl)ethan-1-one (3o)

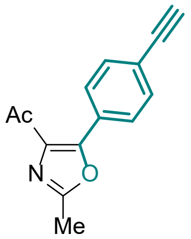

This compound was synthesized following the *general procedure A* using reagent **1a** (60.8 mg, 0.1 mmol), 4-ethynyl-benzoic acid (17.5 mg, 0.12 mmol), sodium carbonate (25.4 mg, 0.24 mmol), acetonitrile (1.0 ml). After flash column chromatography, eluting with Petroleum ether / Ethyl acetate mixtures, **3o** was collected as a white solid (14.4 mg, 64% yield).

^{1}H NMR (400 MHz, $CDCl_3$) δ 8.15 (d, J = 8.6 Hz, 2H), 7.56 (d, J = 8.6 Hz, 2H), 3.19 (s, 1H), 2.62 (s, 3H), 2.55 (s, 3H).

^{13}C NMR (101 MHz, $CDCl_3$) δ 194.5, 159.2, 152.4, 135.0, 132.3, 127.8, 127.5, 124.1, 83.4, 79.3, 29.2, 13.9.

HRMS (ESI-TOF) $[M+H]^+$ calculated for $[C_{14}H_{12}NO_2]^+$ m/z: 226.0863, found 226.0862.

1-(2-methyl-5-(4-vinylphenyl)oxazol-4-yl)ethan-1-one (3p)

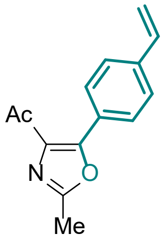

This compound was synthesized following the *general procedure A* using reagent **1a** (60.8 mg, 0.1 mmol), 4-vinylbenzoic acid (17.8 mg, 0.12 mmol), cesium carbonate (78.2 mg, 0.24 mmol), acetonitrile (1.0 ml). After flash column chromatography, eluting with Petroleum ether / Ethyl acetate mixtures, **3p** was collected as a white solid (13.6 mg, 60% yield).

^{1}H NMR (400 MHz, $CDCl_3$) δ 8.14 (d, J = 8.0 Hz, 2H), 7.48 (d, J = 8.0 Hz, 2H), 6.74 (dd, J = 17.6, 10.8 Hz, 1H), 5.84 (d, J = 17.6 Hz, 1H), 5.34 (d, J = 10.8 Hz, 1H), 2.61 (s, 3H), 2.55 (s, 3H).

^{13}C NMR (101 MHz, $CDCl_3$) δ 194.4, 158.8, 153.2, 139.6, 136.3, 134.5, 131.0, 128.2, 126.6, 126.4, 115.6, 29.2, 13.9.

HRMS (ESI-TOF) $[M+H]^+$ calculated for $[C_{14}H_{14}NO_2]^+$ m/z: 228.1019, found 228.1017.

1-(5-(3-fluorophenyl)-2-methyloxazol-4-yl)ethan-1-one (3q)

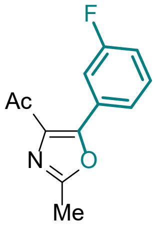

This compound was synthesized following the *general procedure A* using reagent **1a** (60.8 mg, 0.1 mmol), 3-fluorobenzoic acid (16.8 mg, 0.12 mmol), cesium carbonate (78.2 mg, 0.24 mmol), acetonitrile (1.0 ml). After flash column chromatography, eluting with Petroleum ether / Ethyl acetate mixtures, **3q** was collected as a white solid (17.5 mg, 80% yield).

^{1}H NMR (400 MHz, $CDCl_3$) δ 8.04 – 7.89 (m, 2H), 7.42 (q, J = 7.2 Hz, 1H), 7.13 (t, J = 8.4 Hz, 1H), 2.62 (s, 3H), 2.56 (s, 3H).

^{13}C **NMR** (101 MHz, $CDCl_3$) δ 194.5, 162.7 (d, J = 245.4 Hz), 159.2, 151.9 (d, J = 1.8 Hz), 135.0, 130.2 (d, J = 8.2 Hz), 129.2 (d, J = 9.0 Hz), 123.6 (d, J = 2.9 Hz), 117.4 (d, J = 21.3 Hz), 114.9 (d, J = 24.7 Hz), 29.3, 13.9.

^{19}F **NMR** (376 MHz, $CDCl_3$) δ -112.22.

HRMS (ESI-TOF) $[M+H]^+$ calculated for $[C_{12}H_{11}FNO_2]^+$ m/z: 220.0768, found 220.0767.

1-(5-(2-fluorophenyl)-2-methyloxazol-4-yl)ethan-1-one (3r)

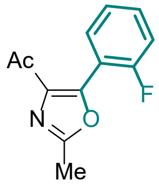

This compound was synthesized following the *general procedure A* using reagent **1a** (60.8 mg, 0.1 mmol), 2-fluorobenzoic acid (16.8 mg, 0.12 mmol), cesium carbonate (78.2 mg, 0.24 mmol), acetonitrile (1.0 ml). After flash column chromatography, eluting with Petroleum ether / Ethyl acetate mixtures, **3r** was collected as a white solid (10.1 mg, 46% yield).

^{1}H **NMR** (400 MHz, $CDCl_3$) δ 7.67 (t, J = 7.4 Hz, 1H), 7.45 (q, J = 5.7 Hz, 1H), 7.23 (t, J = 7.6 Hz, 1H), 7.20 – 7.12 (m, 1H), 2.55 (s, 3H), 2.54 (s, 3H).

^{13}C **NMR** (101 MHz, $CDCl_3$) δ 193.6, 160.0 (d, J = 253.8 Hz), 160.5, 148.0, 136.3, 132.3 (d, J = 8.5 Hz), 131.3 (d, J = 1.9 Hz), 124.1 (d, J = 3.7 Hz), 116.2 (d, J = 21.6 Hz), 116.0, 28.5, 14.0.

^{19}F **NMR** (376 MHz, $CDCl_3$) δ -110.79.

HRMS (ESI-TOF) $[M+H]^+$ calculated for $[C_{12}H_{11}FNO_2]^+$ m/z: 220.0768, found 220.0770.

2-(4-acetyl-2-methyloxazol-5-yl)phenyl acetate (3s)

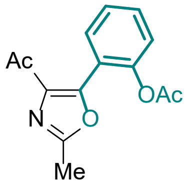

This compound was synthesized following the *general procedure A* using reagent **1a** (60.8 mg, 0.1 mmol), acetylsalicylic acid (21.6 mg, 0.12 mmol), potassium phosphate tribasic (50.9 mg, 0.24 mmol), acetonitrile (1.0 ml). After flash column chromatography, eluting with Petroleum ether / Ethyl acetate mixtures, **3s** was collected as a white solid (17.6 mg, 68% yield).

^{1}H NMR (400 MHz, $CDCl_3$) δ 7.71 (d, J = 8.0 Hz, 1H), 7.50 (t, J = 7.6 Hz, 1H), 7.33 (t, J = 7.6 Hz, 1H), 7.23 (d, J = 8.0 Hz, 1H), 2.52 (s, 3H), 2.50 (s, 3H), 2.17 (s, 3H).

^{13}C NMR (101 MHz, $CDCl_3$) δ 193.3, 168.9, 160.3, 149.7, 148.5, 136.0, 131.7, 131.5, 125.9, 123.3, 120.9, 28.5, 20.9, 13.9.

HRMS (ESI-TOF) $[M+H]^+$ calculated for $[C_{14}H_{14}NO_4]^+$ m/z: 260.0917, found 260.0917.

(2,5-dimethyloxazol-4-yl)(thiophen-2-yl)methanone (3t)

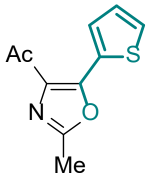

This compound was synthesized following the *general procedure A* using reagent **1a** (60.8 mg, 0.1 mmol), 2-thiophenecarboxylic acid (15.4 mg, 0.12 mmol), cesium carbonate (78.2 mg, 0.24 mmol), acetonitrile (1.0 ml). After flash column chromatography, eluting with Petroleum ether / Ethyl acetate mixtures, **3t** was collected as a white solid (11.2 mg, 54% yield).

^{1}H NMR (400 MHz, $CDCl_3$) δ 8.15 (d, J = 2.4 Hz, 1H), 7.49 (d, J = 5.0 Hz, 1H), 7.18 – 7.12 (m, 1H), 2.60 (s, 3H), 2.53 (s, 3H).

^{13}C NMR (101 MHz, $CDCl_3$) δ 194.1, 158.2, 149.5, 132.6, 129.9, 129.5, 129.2, 127.9, 28.4, 13.9.

HRMS (ESI-TOF) $[M+H]^+$ calculated for $[C_{11}H_{10}N_2O_2]^+$ m/z: 208.0427, found 208.0426.

1-(2-methyl-5-(pyridin-2-yl)oxazol-4-yl)ethan-1-one (3u)

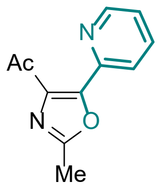

This compound was synthesized following the *general procedure A* using reagent **1a** (60.8 mg, 0.1 mmol), 2-picolinic acid (14.8 mg, 0.12 mmol), cesium carbonate (78.2 mg, 0.24 mmol), acetonitrile (1.0 ml). After flash column chromatography, eluting with Petroleum ether / Ethyl acetate mixtures, **3u** was collected as a white solid (8.7 mg, 43% yield).

^{1}H NMR (400 MHz, $CDCl_3$) δ 8.74 (d, J = 4.0 Hz, 1H), 8.58 (d, J = 7.8 Hz, 1H), 7.82 (t, J = 7.8 Hz, 1H), 7.39 – 7.29 (m, 1H), 2.64 (s, 3H), 2.60 (s, 3H).

^{13}C NMR (101 MHz, $CDCl_3$) δ 194.7, 160.5, 151.5, 149.9, 146.4, 136.9, 136.3, 124.7, 124.5, 29.3, 14.1.

HRMS (ESI-TOF) $[M+H]^+$ calculated for $[C_{11}H_{11}N_2O_2]^+$ m/z: 203.0815, found 203.0813.

1-(2-methyl-5-(pyridin-3-yl)oxazol-4-yl)ethan-1-one (3v)

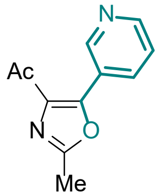

This compound was synthesized following the *general procedure A* using reagent **1a** (60.8 mg, 0.1 mmol), nicotinic acid (14.8 mg, 0.12 mmol), cesium carbonate (78.2 mg, 0.24 mmol), acetonitrile (1.0 ml). After flash column chromatography, eluting with Petroleum ether / Ethyl acetate mixtures, **3v** was collected as a white solid (7.5 mg, 37% yield).

^{1}H NMR (400 MHz, $CDCl_3$) δ 9.21 (s, 1H), 8.68 – 8.58 (m, 2H), 7.39 (dd, J = 8.2, 4.7 Hz, 1H), 2.62 (s, 3H), 2.57 (s, 3H).

^{13}C NMR (101 MHz, $CDCl_3$) δ 194.5, 159.8, 150.9, 150.6, 148.7, 135.8, 135.4, 123.8, 123.3, 29.0, 13.9.

HRMS (ESI-TOF) $[M+H]^+$ calculated for $[C_{11}H_{11}N_2O_2]^+$ m/z: 203.0815, found 203.0814.

1,1'-((perfluoro-1,4-phenylene)bis(2-methyloxazole-5,4-diyl))bis(ethan-1-one) (3w)

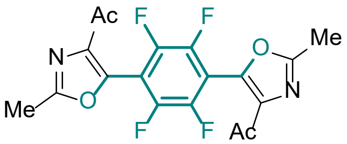

This compound was synthesized following the *general procedure A* using reagent **1a** (121.6 mg, 0.2 mmol), tetrafluoroterephthalic acid (28.6 mg, 0.12 mmol), cesium carbonate (78.2 mg, 0.24 mmol), acetonitrile (1.0 ml). After flash column chromatography, eluting with Petroleum ether / Ethyl acetate mixtures, **3w** was collected as a white solid (15.1 mg, 38% yield).

^{1}H NMR (400 MHz, $CDCl_3$) δ 2.60 (s, 6H), 2.59 (s, 6H).

^{13}C NMR (101 MHz, $CDCl_3$) δ 193.2, 162.8, 158.9 (d, J = 151.1 Hz), 143.1 (d, J = 11.0 Hz), 139.2, 138.73, 3.10, 14.2.

^{19}F NMR (376 MHz, $CDCl_3$) δ -136.78.

HRMS (ESI-TOF) $[M+H]^+$ calculated for $[C_{18}H_{13}F_4N_2O_4]^+$ m/z: 397.0806, found 397.0806.

1-(2-methyl-5-phenethyloxazol-4-yl)ethan-1-one (3x)

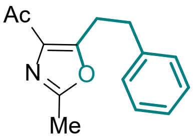

This compound was synthesized following the *general procedure A* using reagent **1a** (60.8 mg, 0.1 mmol), sodium phenylpropionate (20.7 mg, 0.12 mmol), acetonitrile (1.0 ml). After flash column chromatography, eluting with Petroleum ether / Ethyl acetate mixtures, **3x** was collected as a white solid (15.1 mg, 66% yield).

^{1}H NMR (400 MHz, $CDCl_3$) δ 7.26 (d, J = 6.0 Hz, 2H), 7.23 – 7.14 (m, 3H), 3.29 (t, J = 10.0 Hz, 2H), 2.96 (t, J = 10.0 Hz, 2H), 2.48 (s, 3H), 2.43 (s, 3H).

^{13}C NMR (101 MHz, $CDCl_3$) δ 194.8, 158.7, 157.2, 140.4, 134.7, 128.6, 128.5, 126.4, 33.8, 28.1, 28.1, 13.8.

HRMS (ESI-TOF) $[M+H]^+$ calculated for $[C_{14}H_{16}NO_2]^+$ m/z: 230.1176, found 230.1176.

1-(5-(2-bromophenethyl)-2-methyloxazol-4-yl)ethan-1-one (3y)

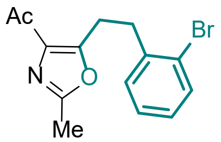

This compound was synthesized following the *general procedure A* using reagent **1a** (60.8 mg, 0.1 mmol), sodium 3-(2-bromophenyl)propanoate (30.1 mg, 0.12 mmol), acetonitrile (1.0 ml). After flash column chromatography, eluting with Petroleum ether / Ethyl acetate mixtures, **3y** was collected as a white solid (22.5 mg, 73% yield).

^{1}H NMR (400 MHz, $CDCl_3$) δ 7.58 – 7.43 (m, 1H), 7.25 – 7.13 (m, 2H), 7.12 – 6.94 (m, 1H), 3.35 – 3.25 (m, 2H), 3.14 – 3.02 (m, 2H), 2.48 (s, 3H), 2.43 (s, 3H).

^{13}C NMR (101 MHz, $CDCl_3$) δ 194.8, 158.9, 156.8, 139.7, 134.8, 133.0, 130.6, 128.2, 127.7, 124.5, 34.1, 28.1, 26.5, 13.9.

HRMS (ESI-TOF) $[M+H]^+$ calculated for $[C_{14}H_{15}BrNO_2]^+$ m/z: 308.0281, found 308.0281.

1-(5-(4-bromobenzyl)-2-methyloxazol-4-yl)ethan-1-one (3z)

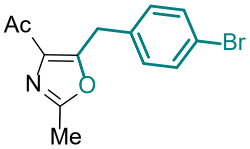

This compound was synthesized following the *general procedure A* using reagent **1a** (60.8 mg, 0.1 mmol), sodium 2-(4-bromophenyl)acetate (28.4 mg, 0.12 mmol), acetonitrile (1.0 ml). After flash column chromatography, eluting with Petroleum ether / Ethyl acetate mixtures, **3z** was collected as a white solid (18.5 mg, 63% yield).

^{1}H NMR (400 MHz, $CDCl_3$) δ 7.40 (d, J = 8.4 Hz, 2H), 7.16 (d, J = 8.4 Hz, 2H), 4.27 (s, 2H), 2.53 (s, 3H), 2.41 (s, 3H).

^{13}C NMR (101 MHz, $CDCl_3$) δ 195.1, 159.4, 155.1, 135.6, 134.6, 131.9, 130.7, 121.1, 31.6, 28.2, 13.9.

HRMS (ESI-TOF) $[M+H]^+$ calculated for $[C_{13}H_{13}BrNO_2]^+$ m/z: 294.0124, found 294.0121.

1-(2-methyl-5-propyloxazol-4-yl)ethan-1-one (3aa)

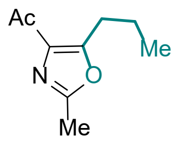

This compound was synthesized following the *general procedure A* using reagent **1a** (60.8 mg, 0.1 mmol), sodium butyrate (13.2 mg, 0.12 mmol), acetonitrile (1.0 ml). After flash column chromatography, eluting with Petroleum ether / Ethyl acetate mixtures, **3aa** was collected as a white solid (12.5 mg, 75% yield).

^{1}H NMR (400 MHz, $CDCl_3$) δ 2.95 (t, J = 7.5 Hz, 2H), 2.49 (s, 3H), 2.43 (s, 3H), 1.72 – 1.60 (m, 2H), 0.94 (t, J = 7.4 Hz, 3H).

^{13}C NMR (101 MHz, $CDCl_3$) δ 194.9, 158.6, 158.3, 134.5, 28.2, 28.1, 21.1, 13.9, 13.8.

HRMS (ESI-TOF) $[M+H]^+$ calculated for $[C_9H_{14}NO_2]^+$ m/z: 168.1019, found 168.1020.

methyl 5-(4-acetyl-2-methyloxazol-5-yl)pentanoate (3ab)

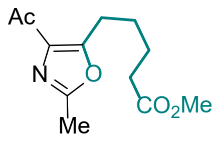

This compound was synthesized following the *general procedure A* using reagent **1a** (60.8 mg, 0.1 mmol), sodium 6-methoxy-6-oxohexanoate (21.9 mg, 0.12 mmol), acetonitrile (1.0 ml). After flash column chromatography, eluting with Petroleum ether/Ethyl acetate mixtures, **3ab** was collected as a white solid (15.8 mg, 66% yield).

^{1}H NMR (400 MHz, $CDCl_3$) δ 3.63 (s, 3H), 2.97 (t, J = 6.8 Hz, 2H), 2.46 (s, 3H), 2.40 (s, 3H), 2.31 (t, J = 6.8 Hz, 2H), 1.73 – 1.57 (m, 4H).

^{13}C NMR (101 MHz, $CDCl_3$) δ 194.9, 173.9, 158.7, 157.6, 134.5, 51.6, 33.7, 28.1, 27.0, 25.7, 24.4, 13.8.

HRMS (ESI-TOF) $[M+H]^+$ calculated for $[C_{12}H_{18}NO_4]^+$ m/z: 240.1230, found 240.1229.

1-(5-(5-bromopentyl)-2-methyloxazol-4-yl)ethan-1-one (3ac)

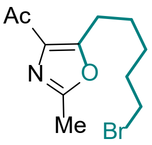

This compound was synthesized following the *general procedure A* using reagent **1a** (60.8 mg, 0.1 mmol), sodium 6-bromohexanoate (26 mg, 0.12 mmol), acetonitrile (1.0 ml). After flash column chromatography, eluting with Petroleum ether / Ethyl acetate mixtures, **3ac** was collected as a white solid (18.4 mg, 67% yield).

^{1}H NMR (400 MHz, $CDCl_3$) δ 3.39 (t, J = 6.6 Hz, 2H), 2.99 (t, J = 7.4 Hz, 2H), 2.49 (s, 3H), 2.43 (s, 3H), 1.96 – 1.80 (m, 2H), 1.72 – 1.61 (m, 2H), 1.56 – 1.39 (m, 2H).

^{13}C NMR (101 MHz, $CDCl_3$) δ 195.0, 158.7, 157.9, 134.5, 33.6, 32.3, 28.2, 27.6, 26.7, 25.9, 13.9.

HRMS (ESI-TOF) $[M+Na]^+$ calculated for $[C_{11}H_{16}BrNO_2Na]^+$ m/z: 298.0236, found 298.0234.

(Z)-1-(5-(heptadec-8-en-1-yl)-2-methyloxazol-4-yl)ethan-1-one (3ad)

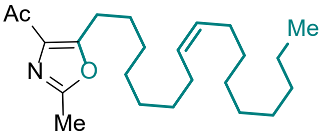

This compound was synthesized following the *general procedure A* using reagent **1a** (60.8 mg, 0.1 mmol), oleic acid (33.9 mg, 0.12 mmol), cesium carbonate (78.2 mg, 0.24 mmol), acetonitrile (1.0 ml). After flash column chromatography, eluting with Petroleum ether / Ethyl acetate mixtures, **3ad** was collected as a white solid (23.1 mg, 64% yield).

^{1}H NMR (400 MHz, $CDCl_3$) δ 5.45 – 5.21 (m, 2H), 2.96 (t, J = 7.6 Hz, 2H), 2.48 (s, 3H), 2.43 (s, 3H), 2.09 – 1.91 (m, 4H), 1.70 – 1.55 (m, 2H), 1.34 – 1.22 (m, 20H), 0.86 (t, J = 6.5 Hz, 3H).

^{13}**C NMR** (101 MHz, $CDCl_3$) δ 194.9, 158.5, 158.5, 134.4, 130.1, 129.9, 32.0, 29.9, 29.8, 29.7, 29.6, 29.5, 29.4, 29.3, 29.2, 28.2, 27.7, 27.4, 27.3, 27.3, 26.2, 25.8, 22.8, 14.2, 13.9.

HRMS (ESI-TOF) $[M+H]^+$ calculated for $[C_{23}H_{40}NO_2]^+$ m/z: 362.3054, found 362.3056.

***tert*-butyl 2-(4-acetyl-2-methyloxazol-5-yl)pyrrolidine-1-carboxylate (3ae)**

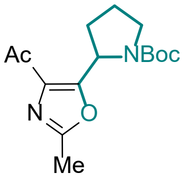

This compound was synthesized following the *general procedure A* using reagent **1a** (60.8 mg, 0.1 mmol), Boc-DL-Pro-OH (25.8 mg, 0.12 mmol), sodium carbonate (25.4 mg, 0.24 mmol), acetonitrile (1.0 ml). After flash column chromatography, eluting with Petroleum ether / Ethyl acetate mixtures, **3ae** was collected as a white solid (17.6 mg, 60% yield).

^{1}**H NMR** (400 MHz, $CDCl_3$) δ 5.53 – 5.23 (m, 1H), 3.54 (d, J = 7.6 Hz, 2H), 2.47 (s, 3H), 2.42 (s, 3H), 2.02 – 1.81 (m, 4H), 1.21 (s, 9H).

^{13}**C NMR** (101 MHz, $CDCl_3$) δ 194.5, 158.6, 158.4, 154.0, 134.1, 79.7, 52.9, 46.8, 32.7, 28.2, 28.0, 24.0, 13.9.

HRMS (ESI-TOF) $[M+H]^+$ calculated for $[C_{15}H_{23}N_2O_4]^+$ m/z: 295.1652, found 295.1652.

1-(2-methyl-5-(3-methyloxetan-3-yl)oxazol-4-yl)ethan-1-one (3af)

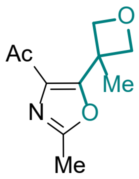

This compound was synthesized following the *general procedure A* using reagent **1a** (60.8 mg, 0.1 mmol), 3-methyloxetane-3-carboxylic acid (13.9 mg, 0.12 mmol), sodium carbonate (25.4 mg, 0.24 mmol), acetonitrile (1.0 ml). After flash column

chromatography, eluting with Petroleum ether / Ethyl acetate mixtures, **3af** was collected as a white solid (9.6 mg, 49% yield).

^{1}H NMR (400 MHz, $CDCl_3$) δ 4.88 (d, J = 6.4 Hz, 2H), 4.57 (d, J = 6.4 Hz, 2H), 2.48 (s, 3H), 2.45 (s, 3H), 1.68 (s, 3H).

^{13}C NMR (101 MHz, $CDCl_3$) δ 194.6, 160.0, 158.5, 133.6, 80.4, 39.3, 27.9, 24.2, 13.8.

HRMS (ESI-TOF) $[M+H]^+$ calculated for $[C_{10}H_{14}NO_3]^+$ m/z: 196.0968, found 196.0971.

(*E*)-1-(2-methyl-5-(4-(trifluoromethyl)styryl)oxazol-4-yl)ethan-1-one (3ag)

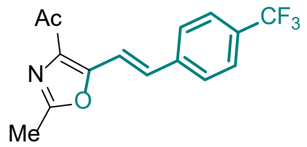

This compound was synthesized following the *general procedure A* using reagent **1a** (60.8 mg, 0.1 mmol), 4-(trifluoromethyl)cinnamic acid (25.9 mg, 0.12 mmol), sodium carbonate (25.4 mg, 0.24 mmol), acetonitrile (1.0 ml). After flash column chromatography, eluting with Petroleum ether / Ethyl acetate mixtures, **3ag** was collected as a white solid (10.9 mg, 37% yield).

^{1}H NMR (400 MHz, $CDCl_3$) δ 7.80 (d, J = 1.6 Hz, 2H), 7.76 (d, J = 8.0 Hz, 2H), 7.64 (d, J = 8.0 Hz, 2H), 2.67 (s, 3H), 2.48 (s, 3H).

^{13}C NMR (101 MHz, $CDCl_3$) δ 184.8, 158.7, 156.3, 141.5, 138.5, 135.0, 131.9 (d, J = 32.7 Hz), 128.9, 125.9 (q, J = 4.0 Hz), 125.2, 13.9, 12.5.

^{19}F NMR (376 MHz, $CDCl_3$) δ -62.82.

HRMS (ESI-TOF) $[M+H]^+$ calculated for $[C_{15}H_{13}F_3NO_2]^+$ m/z: 296.0893, found 296.0898.

(*E*)-1-(5-(4-methoxystyryl)-2-methyloxazol-4-yl)ethan-1-one (3ah)

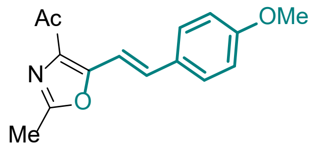

This compound was synthesized following the *general procedure A* using reagent **1a** (60.8 mg, 0.1 mmol), 4-methoxycinnamic acid (21.4 mg, 0.12 mmol), sodium carbonate (25.4 mg, 0.24 mmol), acetonitrile (1.0 ml). After flash column chromatography, eluting with Petroleum ether / Ethyl acetate mixtures, **3ah** was collected as a white solid (14.7 mg, 57% yield).

^{1}H NMR (400 MHz, $CDCl_3$) δ 7.78 (t, J = 16.0 Hz, 2H), 7.67 – 7.61 (m, 2H), 6.91 (d, J = 8.4 Hz, 2H), 3.84 (s, 3H), 2.65 (s, 3H), 2.47 (s, 3H).

^{13}C NMR (101 MHz, $CDCl_3$) δ 185.2, 161.7, 158.5, 155.6, 143.5, 135.2, 130.7, 127.9, 120.7, 114.4, 55.5, 13.9, 12.5.

HRMS (ESI-TOF) $[M+H]^+$ calculated for $[C_{15}H_{16}NO_3]^+$ m/z: 258.1125, found 258.1127.

(2-methyl-5-phenyloxazol-4-yl)(phenyl)methanone (3ai)

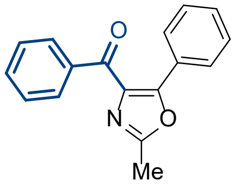

This compound was synthesized following the *general procedure A* using reagent **1e** (67 mg, 0.1 mmol), benzoic acid (14.7 mg, 0.12 mmol), cesium carbonate (78.2 mg, 0.24 mmol), acetonitrile (1.0 ml). After flash column chromatography, eluting with Petroleum ether / Ethyl acetate mixtures, **3ai** was collected as a white solid (13.2 mg, 50% yield).

^{1}H NMR (400 MHz, $CDCl_3$) δ 8.09 – 8.02 (m, 2H), 8.00 – 7.89 (m, 2H), 7.58 – 7.52 (m, 1H), 7.46 (d, J = 7.9 Hz, 2H), 7.44 – 7.40 (m, 3H), 2.58 (s, 3H).

^{13}C NMR (101 MHz, $CDCl_3$) δ 189.0, 159.2, 154.8, 137.6, 133.9, 133.0, 130.4, 130.1, 128.6, 128.3, 127.8, 127.5, 14.0.

HRMS (ESI-TOF) $[M+H]^+$ calculated for $[C_{17}H_{14}NO_2]^+$ m/z: 264.1019, found 264.1024.

(2-methyl-5-phenyloxazol-4-yl)(4-(trifluoromethyl)phenyl)methanone (3aj)

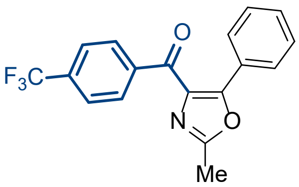

This compound was synthesized following the *general procedure A* using reagent **1f** (73.8 mg, 0.1 mmol), benzoic acid (14.7 mg, 0.12 mmol), sodium carbonate (25.4 mg, 0.24 mmol), acetonitrile (1.0 ml). After flash column chromatography, eluting with Petroleum ether / Ethyl acetate mixtures, **3aj** was collected as a white solid (15.9 mg, 48% yield).

^{1}H NMR (400 MHz, $CDCl_3$) δ 8.17 (d, J = 8.0 Hz, 2H), 8.07 – 7.98 (m, 2H), 7.72 (d, J = 8.0 Hz, 2H), 7.45 (dd, J = 4.2, 2.6 Hz, 3H), 2.59 (s, 3H).

^{13}C NMR (101 MHz, $CDCl_3$) δ 187.6, 159.3, 156.0, 140.8, 137.6, 134.2, 133.9, 133.5, 130.7 (d, J = 3.0 Hz), 128.7, 128.0, 127.3, 125.3 (q, J = 3.8 Hz), 14.0.

^{19}F NMR (376 MHz, $CDCl_3$) δ -63.12.

HRMS (ESI-TOF) $[M+H]^+$ calculated for $[C_{18}H_{13}F_3NO_2]^+$ m/z: 332.0893, found 332.0895.

(2-methyl-5-phenyloxazol-4-yl)(*p*-tolyl)methanone (3ak)

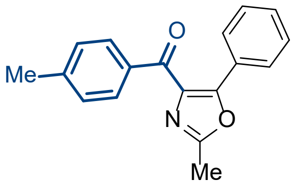

This compound was synthesized following the *general procedure A* using reagent **1g** (68.4 mg, 0.1 mmol), benzoic acid (14.7 mg, 0.12 mmol), cesium carbonate (78.2 mg, 0.24 mmol), acetonitrile (1.0 ml). After flash column chromatography, eluting with Petroleum ether / Ethyl acetate mixtures, **3ak** was collected as a white solid (12.8 mg, 46% yield).

^{1}H NMR (400 MHz, $CDCl_3$) δ 8.04 – 7.86 (m, 4H), 7.46 – 7.34 (m, 3H), 7.25 (d, J = 8.0 Hz, 2H), 2.58 (s, 3H), 2.40 (s, 3H).

^{13}C NMR (101 MHz, $CDCl_3$) δ 194.5, 166.6, 159.6, 152.0, 135.6, 131.4, 131.3, 129.8, 127.8, 52.4, 29.2, 14.0.

HRMS (ESI-TOF) $[M+H]^+$ calculated for $[C_{18}H_{16}NO_2]^+$ m/z: 278.1176, found 278.1179.

(2-methyl-5-(trifluoromethyl)oxazol-4-yl)(phenyl)methanone (3al)

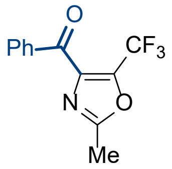

This compound was synthesized following the *general procedure A* using reagent **1e** (67 mg, 0.1 mmol), sodium trifluoroacetate (16.3 mg, 0.12 mmol), acetonitrile (1.0 ml). After flash column chromatography, eluting with Petroleum ether / Ethyl acetate mixtures, **3al** was collected as a white solid (8.7 mg, 34% yield).

^{1}H NMR (400 MHz, $CDCl_3$) δ 8.09 (d, J = 8.01 Hz, 2H), 7.67 – 7.58 (m, 1H), 7.54 – 7.44 (m, 2H), 2.61 (s, 3H).

^{13}C NMR (101 MHz, $CDCl_3$) δ 185.8, 162.0, 141.1 (q, J = 44.3 Hz), 139.0 (q, J = 2.1 Hz), 135.9, 134.1, 130.4, 128.7, 118.6 (q, J = 269.0 Hz), 14.0.

^{19}F NMR (376 MHz, $CDCl_3$) δ -61.96.

HRMS (ESI-TOF) $[M+Na]^+$ calculated for $[C_{12}H_8F_3NO_2Na]^+$ m/z: 278.0399, found 278.0400.

(5-([1,1'-biphenyl]-2-yl)-2-methyloxazol-4-yl)(phenyl)methanone (3am)

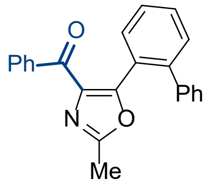

This compound was synthesized following the *general procedure A* using reagent **1e** (67 mg, 0.1 mmol), 2-biphenylcarboxylic acid (23.8 mg, 0.12 mmol), cesium carbonate (78.2 mg, 0.24 mmol), acetonitrile (1.0 ml). After flash column chromatography, eluting with Petroleum ether / Ethyl acetate mixtures, **3am** was collected as a white solid (22.7 mg, 67% yield).

^{1}**H NMR** (400 MHz, $CDCl_3$) δ 7.84 (d, J = 8.0 Hz, 2H), 7.61 (d, J = 7.6 Hz, 1H), 7.49 (q, J = 7.2 Hz, 2H), 7.41 (t, J = 8.0 Hz, 2H), 7.35 (t, J = 7.2 Hz, 2H), 7.20 (t, J = 7.2 Hz, 2H), 7.17 – 7.06 (m, 3H), 2.40 (s, 3H).

^{13}**C NMR** (101 MHz, $CDCl_3$) δ 187.5, 159.6, 155.3, 142.5, 140.6, 137.0, 135.4, 132.7, 130.9, 130.4, 130.3, 130.2, 128.9, 128.2, 128.0, 127.3, 127.2, 126.6, 13.8.

HRMS (ESI-TOF) $[M+H]^+$ calculated for $[C_{23}H_{18}NO_2]^+$ m/z: 340.1332, found 340.1330.

2-methyl-1-(2-methyl-5-phenyloxazol-4-yl)propan-1-one (3an)

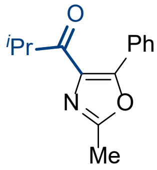

This compound was synthesized following the *general procedure A* using reagent **1h** (63.6 mg, 0.1 mmol), benzoic acid (14.7 mg, 0.12 mmol), cesium carbonate (78.2 mg, 0.24 mmol), acetonitrile (1.0 ml). After flash column chromatography, eluting with Petroleum ether / Ethyl acetate mixtures, **3an** was collected as a white solid (19.7 mg, 86% yield).

^{1}**H NMR** (400 MHz, $CDCl_3$) δ 8.13 (d, J = 7.5 Hz, 2H), 7.48 – 7.39 (m, 3H), 3.77 – 3.60 (m, 1H), 2.55 (s, 3H), 1.21 (s, 3H), 1.19 (s, 3H).

^{13}**C NMR** (101 MHz, $CDCl_3$) δ 200.8, 158.9, 154.0, 133.6, 130.3, 128.5, 128.1, 127.5, 37.6, 18.6, 14.0.

HRMS (ESI-TOF) $[M+H]^+$ calculated for $[C_{14}H_{16}NO_2]^+$ m/z: 230.1176, found 230.1176.

1-(2-methyl-5-phenyloxazol-4-yl)butan-1-one (3ao)

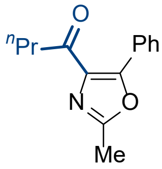

This compound was synthesized following the *general procedure A* using reagent **1i** (63.6 mg, 0.1 mmol), benzoic acid (14.7 mg, 0.12 mmol), cesium carbonate (78.2 mg, 0.24 mmol), acetonitrile (1.0 ml). After flash column chromatography, eluting with Petroleum ether / Ethyl acetate mixtures, **3ao** was collected as a white solid (18.1 mg, 79% yield).

^{1}H NMR (400 MHz, $CDCl_3$) δ 8.15 (d, J = 8.2 Hz, 2H), 7.49 – 7.39 (m, 3H), 3.01 (t, J = 7.2 Hz, 2H), 2.54 (s, 3H), 1.74 (q, J = 7.2 Hz, 2H), 0.99 (t, J = 7.2 Hz, 3H).

^{13}C NMR (101 MHz, $CDCl_3$) δ 196.8, 158.9, 153.3, 134.3, 130.4, 128.5, 128.1, 127.5, 43.0, 17.4, 13.9, 13.9.

HRMS (ESI-TOF) $[M+H]^+$ calculated for $[C_{14}H_{16}NO_2]^+$ m/z: 230.1176, found 230.1175.

methyl 2-methyl-5-phenyloxazole-4-carboxylate (3ap)

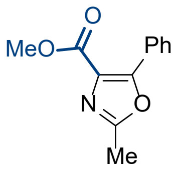

This compound was synthesized following the *general procedure A* using reagent **1j** (63.4 mg, 0.1 mmol), benzoic acid (14.7 mg, 0.12 mmol), sodium carbonate (25.4 mg, 0.24 mmol), Au(PPh_3)Cl (2.5 mg, 5 mol%), acetonitrile (1.0 ml). After flash column chromatography, eluting with Petroleum ether / Ethyl acetate mixtures, **3ap** was collected as a white solid (4.6 mg, 21% yield).

^{1}H NMR (400 MHz, $CDCl_3$) δ 8.05 (d, J = 7.6 Hz, 2H), 7.50 – 7.41 (m, 3H), 3.92 (s, 3H), 2.55 (s, 3H).

^{13}C NMR (101 MHz, $CDCl_3$) δ 162.7, 160.0, 155.6, 130.3, 128.5, 128.3, 127.1, 126.7, 52.3, 13.9.

HRMS (ESI-TOF) $[M+H]^+$ calculated for $[C_{12}H_{12}NO_3]^+$ m/z: 218.0812, found 218.0814.

1-(2,5-diphenyloxazol-4-yl)ethan-1-one (3aq)

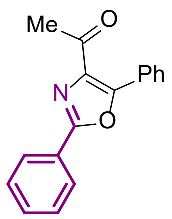

This compound was synthesized following the *general procedure B* using reagent **1a** (60.8 mg, 0.1 mmol), benzoic acid (14.7 mg, 0.12 mmol), sodium carbonate (25.4 mg, 0.24 mmol), Au(PPh_3)Cl (2.5 mg, 5 mol%), benzonitrile (103.1 mg, 1.0 mmol), 1,2-dichloroethane (1.0 mL). After flash column chromatography, eluting with Petroleum ether / Ethyl acetate mixtures, **3aq** was collected as a white solid (14.2 mg, 54% yield).

^{1}H NMR (400 MHz, $CDCl_3$) δ 8.26 (d, J = 8.0 Hz, 2H), 8.17 – 8.10 (m, 2H), 7.53 – 7.49 (m, 4H), 7.49 – 7.46 (m, 2H), 2.72 (s, 3H).

^{13}C NMR (101 MHz, $CDCl_3$) δ 194.8, 158.8, 153.2, 135.6, 132.3, 131.1, 130.6, 129.0, 128.6, 128.2, 127.4, 126.8, 29.2.

HRMS (ESI-TOF) $[M+H]^+$ calculated for $[C_{17}H_{14}NO_2]^+$ m/z: 264.1019, found 264.1020.

1-(2-(naphthalen-2-yl)-5-phenyloxazol-4-yl)ethan-1-one (3ar)

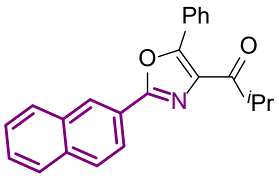

This compound was synthesized following the *general procedure B* using reagent **1h** (63.6 mg, 0.1 mmol), benzoic acid (14.7 mg, 0.12 mmol), potassium phosphate tribasic (50.9 mg, 0.24 mmol), Au(PPh_3)Cl (2.5 mg, 5 mol%), 2-naphthonitrile (306.4 mg, 2.0 mmol), 1,2-dichloroethane (1.0 mL). After flash column chromatography, eluting with Petroleum ether / Ethyl acetate mixtures, **3ar** was collected as a white solid (12.3 mg, 36% yield).

^{1}H NMR (400 MHz, $CDCl_3$) δ 8.62 (s, 1H), 8.31 (d, J = 7.6 Hz, 2H), 8.22 (d, J = 8.6 Hz, 1H), 7.97 (t, J = 6.0 Hz, 2H), 7.93 – 7.85 (m, 1H), 7.61 – 7.46 (m, 5H), 3.94 (p, J = 6.8 Hz, 1H), 1.29 (d, J = 6.8 Hz, 6H).

^{13}C NMR (101 MHz, $CDCl_3$) δ 201.2, 159.1, 154.0, 135.0, 134.6, 133.2, 130.6, 129.0, 128.9, 128.6, 128.4, 128.1, 127.7, 127.6, 127.1, 126.8, 124.2, 123.5, 37.7, 18.7.

HRMS (ESI-TOF) $[M+H]^+$ calculated for $[C_{23}H_{20}NO_2]^+$ m/z: 342.1489, found 342.1466.

1-(2-(tert-butyl)-5-phenyloxazol-4-yl)-2-methylpropan-1-one (3as)

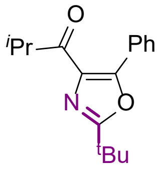

This compound was synthesized following the *general procedure A* using reagent **1h** (63.6 mg, 0.1 mmol), ammonium benzoate (16.7 mg, 0.12 mmol), $Au(PPh_3)Cl$ (2.5 mg, 5 mol%), trimethylacetonitrile (1.0 mL). After flash column chromatography, eluting with Petroleum ether / Ethyl acetate mixtures, **3as** was collected as a white solid (12.5 mg, 46% yield).

^{1}H NMR (400 MHz, $CDCl_3$) δ 8.15 (d, J = 7.8 Hz, 2H), 7.51 – 7.35 (m, 3H), 3.83 – 3.68 (m, 1H), 1.45 (s, 9H), 1.21 (s, 3H), 1.19 (s, 3H).

^{13}C NMR (101 MHz, $CDCl_3$) δ 201.4, 168.5, 153.3, 133.3, 130.2, 128.5, 128.1, 127.9, 37.6, 33.8, 28.7, 18.6.

HRMS (ESI-TOF) $[M+H]^+$ calculated for $[C_{17}H_{22}NO_2]^+$ m/z: 272.1645, found 272.1643.

1-(2-phenethyl-5-phenyloxazol-4-yl)ethan-1-one (3at)

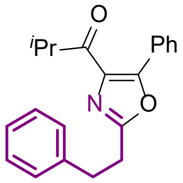

This compound was synthesized following the *general procedure B* using reagent **1h** (63.6 mg, 0.1 mmol), sodium carbonate (25.4 mg, 0.24 mmol), $Au(PPh_3)Cl$ (2.5 mg, 5 mol%), 3-phenylpropionitrile (131.2 mg, 1.0 mmol), 1,2-dichloroethane (1.0 mL). After flash column chromatography, eluting with Petroleum ether / Ethyl acetate

mixtures, **3at** was collected as a white solid (9.3 mg, 29% yield).

^{1}H NMR (400 MHz, $CDCl_3$) δ 8.10 (d, J = 8.2 Hz, 2H), 7.44 (d, J = 6.6 Hz, 3H), 7.30 (t, J = 7.4 Hz, 2H), 7.26 – 7.20 (m, 3H), 3.71 (hept, J = 6.8 Hz, 1H), 3.16 (s, 4H), 1.19 (d, J = 6.8 Hz, 6H).

^{13}C NMR (101 MHz, $CDCl_3$) δ 200.9, 161.4, 153.9, 140.1, 133.5, 130.4, 128.7, 128.5, 128.5, 128.2, 127.6, 126.6, 37.6, 33.2, 30.0, 18.6.

HRMS (ESI-TOF) $[M+H]^+$ calculated for $[C_{21}H_{22}NO_2]^+$ m/z: 320.1645, found 320.1648.

2-methyl-1-(5-phenyl-2-vinyloxazol-4-yl)propan-1-one (3au)

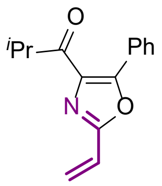

This compound was synthesized following the *general procedure A* using reagent **1h** (63.6 mg, 0.1 mmol), benzoic acid (14.7 mg, 0.12 mmol), sodium carbonate (25.4 mg, 0.24 mmol), Au(PPh_3)Cl (2.5 mg, 5 mol%), acrylonitrile (1.0 ml). After flash column chromatography, eluting with Petroleum ether / Ethyl acetate mixtures, **3au** was collected as a white solid (12.8 mg, 57% yield).

^{1}H NMR (400 MHz, $CDCl_3$) δ 8.18 (d, J = 8.0 Hz, 2H), 7.51 – 7.42 (m, 3H), 6.65 (dd, J = 17.6, 11.2 Hz, 1H), 6.30 (d, J = 18.0 Hz, 1H), 5.76 (d, J = 10.8 Hz, 1H), 3.80 – 3.69 (m, 1H), 1.22 (s, 3H), 1.20 (s, 3H).

^{13}C NMR (101 MHz, $CDCl_3$) δ 200.9, 158.1, 153.7, 134.5, 130.7, 128.6, 128.4, 127.4, 123.4, 123.1, 37.6, 18.6.

HRMS (ESI-TOF) $[M+H]^+$ calculated for $[C_{15}H_{16}NO_2]^+$ m/z: 242.1176, found 242.1179.

5-(4-acetyl-5-phenyloxazol-2-yl)pentanenitrile (3av)

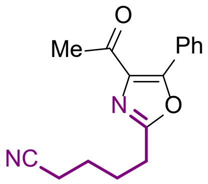

This compound was synthesized following the *general procedure B* using reagent **1a** (60.8 mg, 0.1 mmol), benzoic acid (14.7 mg, 0.12 mmol), sodium carbonate (25.4 mg, 0.24 mmol), Au(PPh3)Cl (2.5 mg, 5 mol%), adiponitrile (108.1 mg, 1.0 mmol), 1,2-dichloroethane (1.0 mL). After flash column chromatography, eluting with Petroleum ether / Ethyl acetate mixtures, **3av** was collected as a white solid (10.2 mg, 38% yield).

^{1}H NMR (400 MHz, $CDCl_3$) δ 8.12 (d, J = 8.0 Hz, 2H), 7.44 (p, J = 5.0 Hz, 3H), 2.89 (t, J = 7.2 Hz, 2H), 2.60 (s, 3H), 2.47 – 2.37 (m, 4H), 2.05 – 1.97 (m, 2H).

^{13}C NMR (101 MHz, $CDCl_3$) δ 194.4, 161.0, 153.3, 134.3, 130.5, 128.5, 128.0, 127.2, 119.3, 29.1, 27.1, 25.8, 24.8, 24.4, 17.0, 16.7.

HRMS (ESI-TOF) $[M+H]^+$ calculated for $[C_{16}H_{17}N_2O_2]^+$ m/z: 269.1285, found 269.1285.

3.4 Late-stage functionalization of drugmolecules

1-(5-((1-(4-chlorobenzoyl)-5-methoxy-2-methyl-1*H*-indol-3-yl)methyl)-2-methyloxazol-4-yl)ethan-1-one (4a)

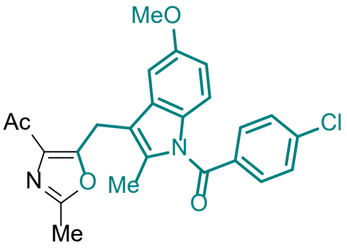

This compound was synthesized following the *general procedure A* using reagent **1a** (60.8 mg, 0.1 mmol), indometacin (42.9 mg, 0.12 mmol), caesium fluoride (36.5 mg, 0.24 mmol), acetonitrile (1.0 ml). After flash column chromatography, eluting with Petroleum ether / Ethyl acetate mixtures, **4a** was collected as a white solid (22.3 mg, 51% yield).

^{1}H NMR (400 MHz, $CDCl_3$) δ 7.64 (d, J = 6.0 Hz, 2H), 7.45 (d, J = 6.0 Hz, 2H), 7.16 – 6.94 (m, 1H), 6.90 – 6.72 (m, 1H), 6.70 – 6.49 (m, 1H), 4.55 – 4.14 (m, 2H), 3.81 (s, 3H), 2.56 (s, 3H), 2.47 (s, 3H), 2.39 (s, 3H).

^{13}C NMR (101 MHz, $CDCl_3$) δ 195.5, 168.5, 159.2, 156.2, 154.9, 139.4, 135.9, 134.3, 134.0, 131.3, 131.3, 130.8, 130.7, 129.2, 129.2, 114.9, 114.7, 112.0, 101.5, 55.7, 55.7, 28.3, 28.3, 21.1, 13.9, 13.5, 13.4.

HRMS (ESI-TOF) $[M+H]^+$ calculated for $[C_{24}H_{22}ClN_2O_4]^+$ m/z: 437.1263, found 437.1262.

(S)-1-(5-(1-(6-methoxynaphthalen-2-yl)ethyl)-2-methyloxazol-4-yl)ethan-1-one (4b)

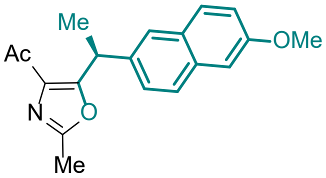

This compound was synthesized following the *general procedure A* using reagent **1a** (60.8 mg, 0.1 mmol), naproxen (27.6 mg, 0.12 mmol), cesium carbonate (78.2 mg, 0.24 mmol), acetonitrile (1.0 ml). After flash column chromatography, eluting with Petroleum ether / Ethyl acetate mixtures, **4b** was collected as a white solid (14.8 mg, 48% yield).

^{1}H NMR (400 MHz, $CDCl_3$) δ 7.73 (s, 1H), 7.69 (dd, J = 15.2, 8.8 Hz, 2H), 7.46 (d, J = 8.4 Hz, 1H), 7.13 (dt, J = 8.8, 2.0 Hz, 1H), 7.09 (s, 1H), 5.20 (q, J = 7.2 Hz, 1H), 3.90 (s, 3H), 2.53 (s, 3H), 2.44 (s, 3H), 1.71 (d, J = 7.2 Hz, 3H).

^{13}C NMR (101 MHz, $CDCl_3$) δ 195.2, 159.9, 158.9, 157.7, 137.2, 133.7, 133.3, 129.4, 129.0, 127.2, 126.6, 125.7, 119.1, 105.7, 55.4, 36.2, 28.4, 19.0, 14.0.

HRMS (ESI-TOF) $[M+H]^+$ calculated for $[C_{19}H_{20}NO_3]^+$ m/z: 310.1438, found 310.1443.

1-(5-(1-(2-fluoro-[1,1'-biphenyl]-4-yl)ethyl)-2-methyloxazol-4-yl)ethan-1-one (4c)

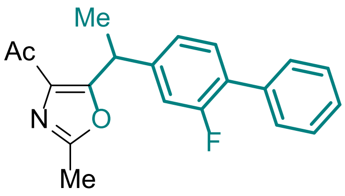

This compound was synthesized following the *general procedure A* using reagent **1a** (60.8 mg, 0.1 mmol), flubiprofen (29.3 mg, 0.12 mmol), sodium carbonate (25.4 mg, 0.24 mmol), acetonitrile (1.0 ml). After flash column chromatography, eluting with Petroleum ether / Ethyl acetate mixtures, **4c** was collected as a white solid (11.6 mg, 36% yield).

^{1}H NMR (400 MHz, $CDCl_3$) δ 7.51 (d, J = 8.4 Hz, 2H), 7.42 (t, J = 7.6 Hz, 2H), 7.39 – 7.32 (m, 2H), 7.21 (dd, J = 8.0, 1.6 Hz, 1H), 7.16 (dd, J = 11.8, 1.6 Hz, 1H), 5.10 (q, J = 7.6 Hz, 1H), 2.53 (s, 3H), 2.47 (s, 3H), 1.65 (d, J = 7.6 Hz, 3H).

^{13}C NMR (101 MHz, $CDCl_3$) δ 195.2, 161.1, 159.2, 158.9, 158.6, 143.5 (d, J = 7.6 Hz), 135.7, 133.6, 130.9 (d, J = 3.9 Hz), 129.1 (d, J = 3.0 Hz), 128.6, 127.8, 127.8, 127.7, 123.7 (d, J = 3.4 Hz), 115.2 (d, J = 23.5 Hz), 35.9, 28.3, 18.9, 14.0.

^{19}F NMR (376 MHz, $CDCl_3$) δ -117.57.

HRMS (ESI-TOF) $[M+H]^+$ calculated for $[C_{20}H_{19}FNO_2]^+$ m/z: 324.1394, found 324.1394.

1-(5-(1-(4-isobutylphenyl)ethyl)-2-methyloxazol-4-yl)ethan-1-one (4d)

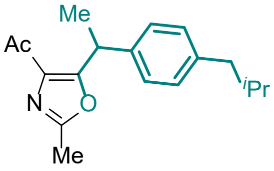

This compound was synthesized following the *general procedure A* using reagent **1a** (60.8 mg, 0.1 mmol), ibuprofen (24.8 mg, 0.12 mmol), sodium carbonate (25.4 mg, 0.24 mmol), acetonitrile (1.0 ml). After flash column chromatography, eluting with Petroleum ether / Ethyl acetate mixtures, **4d** was collected as a white solid (12.8 mg, 45% yield).

^{1}H NMR (400 MHz, CDCl$_3$) δ 7.27 (d, J = 8.0 Hz, 2H), 7.07 (d, J = 8.0 Hz, 2H), 5.04 (q, J = 7.2 Hz, 1H), 2.50 (s, 3H), 2.43 (s, 3H), 2.41 (s, 2H), 1.83 (p, J = 6.6 Hz, 1H), 1.61 (d, J = 7.2 Hz, 3H), 0.89 (s, 3H), 0.88 (s, 3H).

^{13}C NMR (101 MHz, CDCl$_3$) δ 195.1, 160.0, 158.8, 140.5, 139.3, 133.2, 129.4, 127.3, 45.1, 35.9, 30.3, 28.3, 22.5, 19.1, 14.0.

HRMS (ESI-TOF) $[M+H]^+$ calculated for $[C_{18}H_{24}NO_2]^+$ m/z: 286.1802, found 286.1811.

1-(2-methyl-5-(2-phenylquinolin-4-yl)oxazol-4-yl)ethan-1-one (4e)

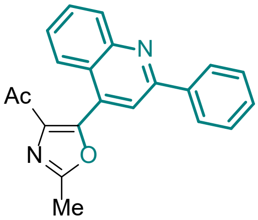

This compound was synthesized following the *general procedure A* using reagent **1a** (60.8 mg, 0.1 mmol), cinchophen (30 mg, 0.12 mmol), cesium carbonate (78.2 mg, 0.24 mmol), acetonitrile (1.0 ml). After flash column chromatography, eluting with Petroleum ether / Ethyl acetate mixtures, **4e** was collected as a white solid (11.2 mg, 34% yield).

^{1}H NMR (400 MHz, CDCl$_3$) δ 8.25 (d, J = 8.4 Hz, 1H), 8.21 (s, 1H), 8.20 (d, J = 3.2 Hz, 2H), 7.78 (t, J = 7.6 Hz, 2H), 7.54 (t, J = 7.6 Hz, 3H), 7.47 (t, J = 7.2 Hz, 1H), 2.65 (s, 3H), 2.58 (s, 3H).

^{13}C NMR (101 MHz, CDCl$_3$) δ 193.4, 161.1, 156.9, 150.4, 148.8, 139.2, 137.5, 133.8, 130.6, 130.0, 129.7, 129.0, 127.8, 127.2, 124.9, 124.5, 121.3, 28.7, 14.1.

HRMS (ESI-TOF) $[M+H]^+$ calculated for $[C_{21}H_{17}N_2O_2]^+$ m/z: 329.1285, found 329.1290.

5-(5-(4-acetyl-2-methyloxazol-5-yl)-4-methylthiazol-2-yl)-2-isobutoxybenzonitrile (4f)

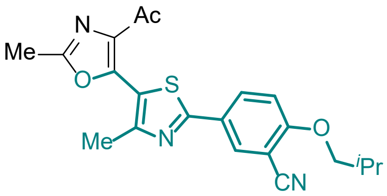

This compound was synthesized following the *general procedure A* using reagent **1a** (60.8 mg, 0.1 mmol), febuxostat (38 mg, 0.12 mmol), cesium carbonate (78.2 mg, 0.24 mmol), acetonitrile (1.0 ml). After flash column chromatography, eluting with Petroleum ether / Ethyl acetate mixtures, **4f** was collected as a white solid (13.8 mg, 35% yield).

^{1}H NMR (400 MHz, $CDCl_3$) δ 8.20 (d, J = 2.4 Hz, 1H), 8.08 (dd, J = 8.8, 2.4 Hz, 1H), 6.99 (d, J = 8.8 Hz, 1H), 3.89 (d, J = 6.4 Hz, 2H), 2.65 (s, 3H), 2.59 (s, 3H), 2.56 (s, 3H), 2.23 – 2.16 (m, 1H), 1.09 (s, 3H), 1.07 (s, 3H).

^{13}C NMR (101 MHz, $CDCl_3$) δ 194.1, 165.7, 162.2, 159.4, 156.5, 146.6, 134.6, 132.5, 132.0, 126.3, 117.8, 115.6, 112.7, 103.0, 75.8, 28.4, 28.3, 19.2, 18.4, 14.0.

HRMS (ESI-TOF) $[M+H]^+$ calculated for $[C_{21}H_{22}N_3O_3S]^+$ m/z: 396.1376, found 396.1376.

2-((4-acetyl-2-methyloxazol-5-yl)methyl)dibenzo[b,e]oxepin-11(6*H*)-one (4g)

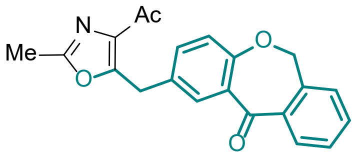

This compound was synthesized following the *general procedure A* using reagent **1a** (60.8 mg, 0.1 mmol), isoxepac (32.2 mg, 0.12 mmol), cesium carbonate (78.2 mg, 0.24 mmol), acetonitrile (1.0 ml). After flash column chromatography, eluting with Petroleum ether / Ethyl acetate mixtures, **4g** was collected as a white solid (18.1 mg, 52% yield).

^{1}H NMR (400 MHz, $CDCl_3$) δ 8.12 (d, J = 2.4 Hz, 1H), 7.89 (d, J = 7.6 Hz, 1H), 7.58 – 7.52 (m, 1H), 7.50 – 7.40 (m, 2H), 7.35 (d, J = 7.2 Hz, 1H), 6.98 (d, J = 8.4 Hz, 1H), 5.17 (s, 2H), 4.34 (s, 2H), 2.54 (s, 3H), 2.42 (s, 3H).

^{13}C NMR (101 MHz, $CDCl_3$) δ 195.1, 191.0, 160.5, 159.4, 155.4, 140.6, 136.2, 135.7, 134.6, 132.9, 131.9, 130.4, 129.6, 129.4, 127.9, 125.4, 121.3, 73.8, 31.3, 28.2, 14.0.

HRMS (ESI-TOF) $[M+H]^+$ calculated for $[C_{21}H_{18}NO_4]^+$ m/z: 348.1230, found 348.1235.

1-(2-methyl-5-((1-methyl-5-(4-methylbenzoyl)-1*H*-pyrrol-2-yl)methyl)oxazol-4-yl)ethan-1-one (4h)

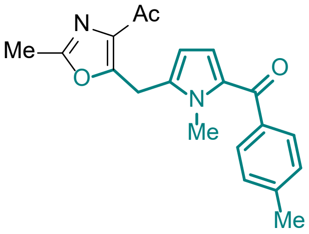

This compound was synthesized following the *general procedure A* using reagent **1a** (60.8 mg, 0.1 mmol), tolmetin (30.9 mg, 0.12 mmol), cesium carbonate (78.2 mg, 0.24 mmol), acetonitrile (1.0 ml). After flash column chromatography, eluting with Petroleum ether / Ethyl acetate mixtures, **4h** was collected as a white solid (18.2 mg, 54% yield).

^{1}H NMR (400 MHz, $CDCl_3$) δ 7.69 (d, J = 8.0 Hz, 2H), 7.23 (d, J = 8.0 Hz, 2H), 6.64 (d, J = 4.0 Hz, 1H), 6.05 (d, J = 4.0 Hz, 1H), 4.43 (s, 2H), 3.95 (s, 3H), 2.55 (s, 3H), 2.45 (s, 3H), 2.41 (s, 3H).

^{13}C NMR (101 MHz, $CDCl_3$) δ 195.3, 186.0, 159.8, 152.6, 142.1, 137.4, 136.5, 134.8, 131.5, 129.6, 128.8, 122.5, 109.2, 33.4, 28.2, 24.0, 21.7, 14.0.

HRMS (ESI-TOF) $[M+H]^+$ calculated for $[C_{20}H_{21}N_2O_3]^+$ m/z: 337.1547, found 337.1547.

(5*S*,8*R*,9*S*,10*S*,13*R*,14*S*,17*R*)-17-((*R*)-4-(4-acetyl-2-methyloxazol-5-yl)butan-2-yl)-10,13-dimethyldodecahydro-3*H*-cyclopenta[*a*]phenanthrene-3,7,12(2*H*,4*H*)-trione (4i)

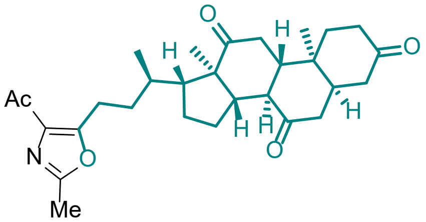

This compound was synthesized following the *general procedure A* using reagent **1a** (60.8 mg, 0.1 mmol), dehydrocholic acid (48.3 mg, 0.12 mmol), sodium carbonate (25.4 mg, 0.24 mmol), acetonitrile (1.0 ml). After flash column chromatography, eluting with Petroleum ether / Ethyl acetate mixtures, **4i** was collected as a white solid (32.2 mg, 67% yield).

^{1}H NMR (400 MHz, $CDCl_3$) δ 3.04 – 2.81 (m, 6H), 2.49 (s, 3H), 2.43 (s, 3H), 2.35 – 2.24 (m, 5H), 2.16 – 1.99 (m, 7H), 1.61 (td, J = 14.4, 5.0 Hz, 2H), 1.44 (dd, J = 9.2, 4.9 Hz, 1H), 1.39 (s, 3H), 1.29 – 1.23 (m, 3H), 1.06 (s, 3H), 0.92 (d, J = 6.6 Hz, 3H).

^{13}C NMR (101 MHz, $CDCl_3$) δ 212.1, 209.2, 208.9, 194.9, 158.8, 158.6, 134.2, 57.0, 51.9, 49.1, 47.0, 45.7, 45.7, 45.1, 42.9, 38.7, 36.6, 36.1, 35.4, 33.4, 28.2, 27.8, 25.3, 23.7, 22.0, 18.9, 13.9, 12.0.

HRMS (ESI-TOF) $[M+H]^+$ calculated for $[C_{29}H_{40}NO_5]^+$ m/z: 482.2901, found 482.2901.

4. Mechanistic Studies

4.1 ^{18}O-isotope labeling

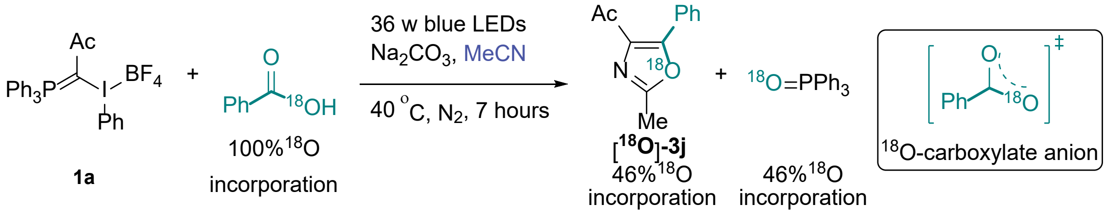

A dry, clean test tube equipped with a stir bar was charged with the substrate **1a** (0.1 mmol), **2b** (0.12 mmol), sodium carbonate (0.24 mmol), and acetonitrile (1.0 mL) was added using a syringe under a nitrogen atmosphere. The resulting mixture was stirred for 7 h at 40 °C (water bath temperature) under blue light(λ_{max} = 438 nm) at 36W. After this time, the tube was cooled to room temperature. After removing the volatiles under reduced pressure, the residue was then purified by column chromatography on silica gel (Petroleum ether / Ethyl acetate) to afford the compounds.

1-(2-methyl-5-phenyloxazol-4-yl-1-^{18}*O*)ethan-1-one ([^{18}O]-3j)

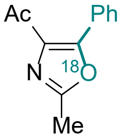

^{1}H NMR (400 MHz, $CDCl_3$) δ 8.19 – 8.04 (m, 2H), 7.43 (d, *J* = 6.8 Hz, 3H), 2.60 (s, 3H), 2.53 (s, 3H).

^{13}C NMR (101 MHz, $CDCl_3$) δ 194.3, 158.9, 153.3, 134.4, 130.4, 128.5, 128.0, 127.3, 29.1, 13.9.

HRMS (ESI-TOF) [M+Na]$^+$ calculated for $[C_{12}H_{11}N^{18}O_2Na]^+$ m/z: 224.0682, found 224.06865.

Supplementary Table 2. GC-MS date of [^{18}O]-3j.

m/z	Absolute Intensity	Relative Intensity	m/z	Absolute Intensity	Relative Intensity
150.00	135	0.02	179.00	356	0.04
151.00	223	0.03	180.00	134	0.02
152.00	239	0.03	181.00	212	0.02
153.00	322	0.04	182.00	842	0.09
154.00	1148	0.13	183.00	4378	0.49
155.00	737	0.08	184.00	20439	2.30
156.05	1313	0.15	185.05	5365	0.60
157.05	2695	0.30	186.00	348663	39.25
158.00	310508	34.96	187.00	48582	5.47
159.00	157057	17.68	188.00	322230	36.28
160.00	167572	18.87	189.00	39258	4.42
161.00	122950	13.84	190.00	3010	0.34
162.00	25676	2.89	190.95	576	0.06
163.00	2534	0.29	191.90	156	0.02
164.00	247	0.03	192.90	945	0.11
165.00	212	0.02	193.80	305	0.03
166.00	292	0.03	194.80	218	0.02
167.00	330	0.04	195.80	97	0.01
168.00	111	0.01	196.80	114	0.01
168.90	321	0.04	197.95	371	0.04
170.10	630	0.07	199.05	3855	0.43
171.05	408	0.05	200.00	856850	96.46
172.05	25836	2.91	201.00	888250	100.00
173.05	11312	1.27	202.00	866324	97.53
174.00	2940	0.33	203.00	767929	86.45
175.00	895	0.10	204.00	103665	11.67
176.05	445	0.05	205.00	8724	0.98
177.00	318	0.04	206.05	569	0.06
178.00	246	0.03	207.00	1081	0.12

Supplementary Table 3. GC-MS date of ^{18}O=PPh$_3$.

m/z	Absolute Intensity	Relative Intensity	m/z	Absolute Intensity	Relative Intensity
230.95	7545	0.09	257.95	19184	0.23
231.95	1918	0.02	258.95	38135	0.46
232.95	19078	0.23	259.95	10170	0.12
233.90	3740	0.05	261.00	11737	0.14
234.90	622	0.01	262.00	30650	0.37
236.00	446	0.01	262.95	5980	0.07
237.00	270	0.00	263.95	635	0.01
238.00	220	0.00	264.90	394	0.00

239.00	239	0.00	265.90	137	0.00
240.00	89	0.00	266.95	490	0.01
240.90	145	0.00	268.00	174	0.00
241.95	709	0.01	269.00	242	0.00
242.95	938	0.01	270.00	98	0.00
243.95	10227	0.12	271.00	178	0.00
244.95	4550	0.05	271.90	71	0.00
245.95	1777	0.02	272.95	1770	0.02
246.95	1972	0.02	273.95	520	0.01
248.00	786	0.01	274.95	7286	0.09
249.00	1690	0.02	276.05	87106	1.05
249.95	691	0.01	277.05	8303359	100.00
250.90	1785	0.02	278.00	4974625	59.91
251.85	419	0.01	279.00	7096058	85.46
252.95	1306	0.02	280.00	2550927	30.72
253.95	361	0.00	280.95	396223	4.77
254.90	4254	0.05	281.90	34252	0.41
255.95	1794	0.02	282.95	2422	0.03
256.95	65699	0.79	283.90	161	0.00

4.2 Step-wise study

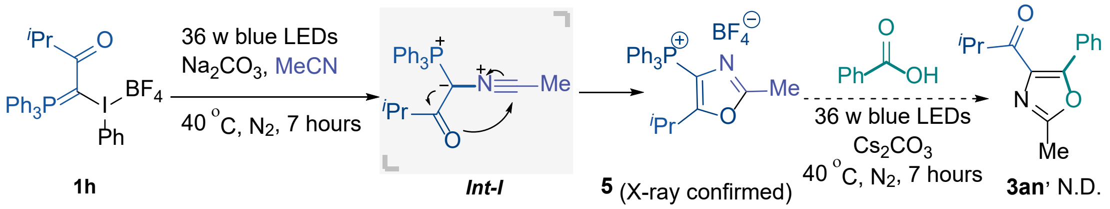

A dry, clean test tube equipped with a stir bar was charged with the substrate **1h** (0.1 mmol). Acetonitrile (1.0 mL) was added using a syringe under a nitrogen atmosphere. The resulting mixture was stirred for 7 h at 40 °C (water bath temperature) under blue light(λ_{max} = 438 nm) at 36 W. After this time, the tube was cooled to room temperature. Then, half of the solution was taken for post-treatment to separate the intermediate product. The other half of the solution was mixed with benzoic acid and cesium carbonate to continue the reaction under standard conditions. After the usual post-treatment, it was found that the product **3an** was not obtained.

(5-isopropyl-2-methyloxazol-4-yl)triphenylphosphonium tetrafluoroborate (5)

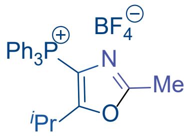

^{1}H NMR (400 MHz, CDCl3) δ 7.90 – 7.82 (m, 3H), 7.78 – 7.70 (m, 9H), 7.70 – 7.67 (m, 3H), 2.57 (s, 3H), 1.27 – 1.19 (m, 1H), 1.06 (s, 3H), 1.04 (s, 3H).

^{13}C NMR (101 MHz, CDCl3) δ 171.1 (d, J = 30.7 Hz), 163.7 (d, J = 20.2 Hz), 136.0, 136.0, 134.3, 134.2, 130.8, 130.7, 117.9, 117.0, 113.9, 112.6, 26.4, 21.1, 14.0.

^{19}F NMR (376 MHz, CDCl3) δ -153.93 (d, J = 19.6 Hz).

^{31}P NMR (162 MHz, CDCl3) δ 9.75.

HRMS (ESI-TOF) $[M-BF_4]^+$ calculated for $[C_{25}H_{25}NOP]^+$ m/z: 386.1668, found 386.1666.

The crystal structure of **5** has been deposited at the Cambridge Crystallographic Data Centre, CCDC2269362.

4.3 Control experiment

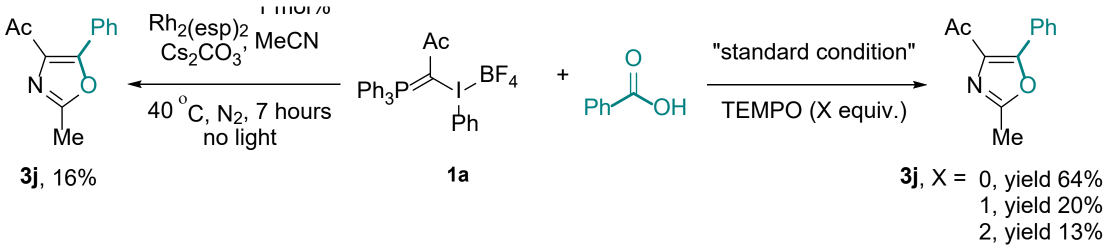

Rhodium catalytic reaction

A dry, clean test tube equipped with a stir bar was charged with the benzoic acid (0.12 mmol), cesium carbonate (0.24 mmol), and the hypervalent iodine **1a** (0.1 mmol), and $Rh_2(esp)_2$ (1 mol%). And acetonitrile (1.0 mL) were added in a syringe under a nitrogen atmosphere. The resulting mixture was stirred for 7 h at 40 °C (water bath temperature) with no light. After this time, the tube was cooled to room temperature. After removing the volatiles under reduced pressure, the residue was then purified by column chromatography on silica gel (Petroleum ether / Ethyl acetate) to afford the product **3j** (16% yield).

Radical capture reaction

A dry, clean test tube equipped with a stir bar was charged with the benzoic acid (0.12 mmol), cesium carbonate (0.24 mmol), and the hypervalent iodine **1a** (0.1 mmol), and TEMPO (0 mmol, 0 equiv; or 0.1 mmol, 1.0 equiv; or 0.2 mmol, 2.0 equiv). And acetonitrile (1.0 mL) were added using a syringe under a nitrogen atmosphere. The resulting mixture was stirred for 7 h at 40 °C (water bath temperature) under blue light at 36W. After the reaction was completed, the results were determined by ^{1}H NMR analysis using trimethoxybenzene as an internal standard, and 64%, 20%, 13% yields of **3j** were obtained, respectively.

4.4 Gram-scale reaction

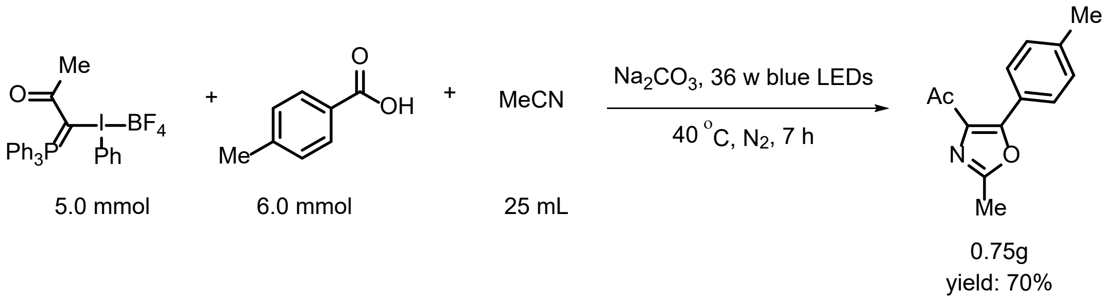

A dry, clean round flask equipped with a stir bar was charged with the substrate **1a** (5.0 mmol), *p*-toluic acid (6.0 mmol), sodium carbonate (12.0 mmol), and acetonitrile (25 mL) was added using a syringe under a nitrogen atmosphere. The resulting mixture was stirred for 7 h at 40 °C (water bath temperature) under blue light(λ_{max} = 438 nm) at 36W. After the reaction was completed, the result was determined by ^{1}H NMR analysis using trimethoxybenzene as an internal standard, and 70% yields of **3a** were obtained.

4.5 UV-Visible absorption analysis

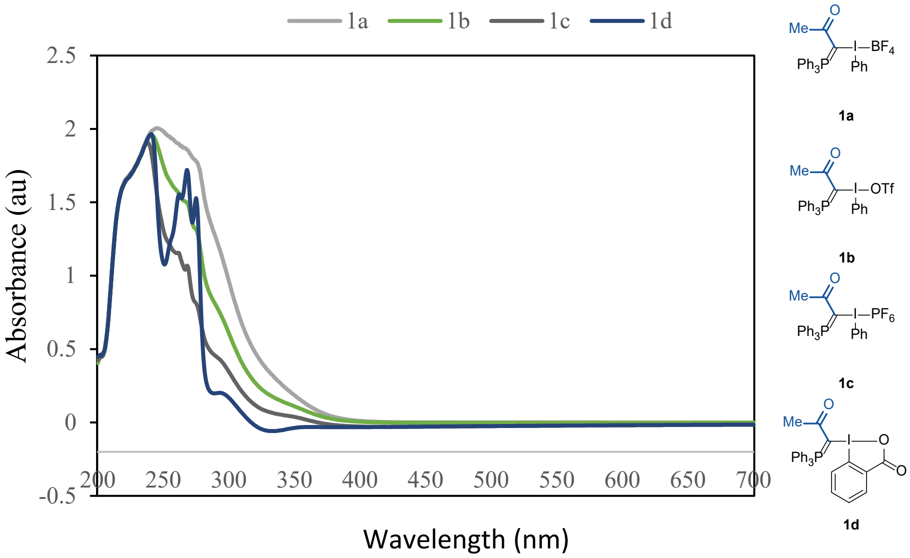

Supplementary Figure 3. UV-Vis spectra of $I^{(III)}/P^{(V)}$ Reagents **1** at 10^{-4} M in MeCN.

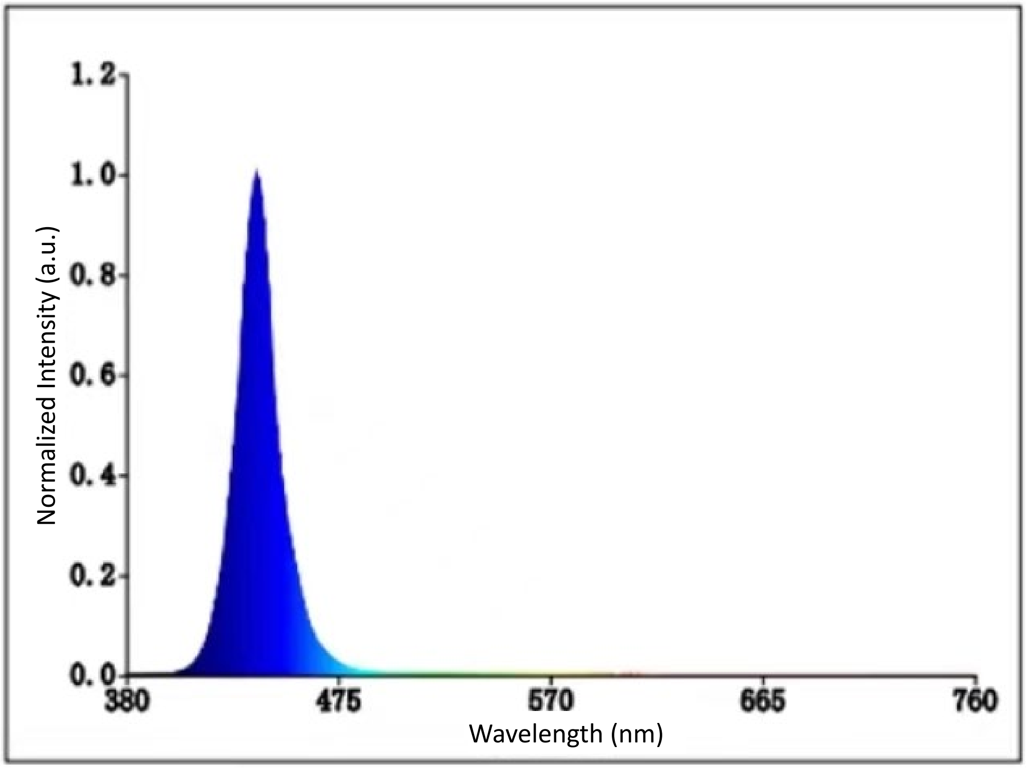

Supplementary Figure 4. Emission spectra of the blue LEDs used in this reaction. λ_{max} = 438 nm, color purity = 99.3%.

5. Supplementary Figures

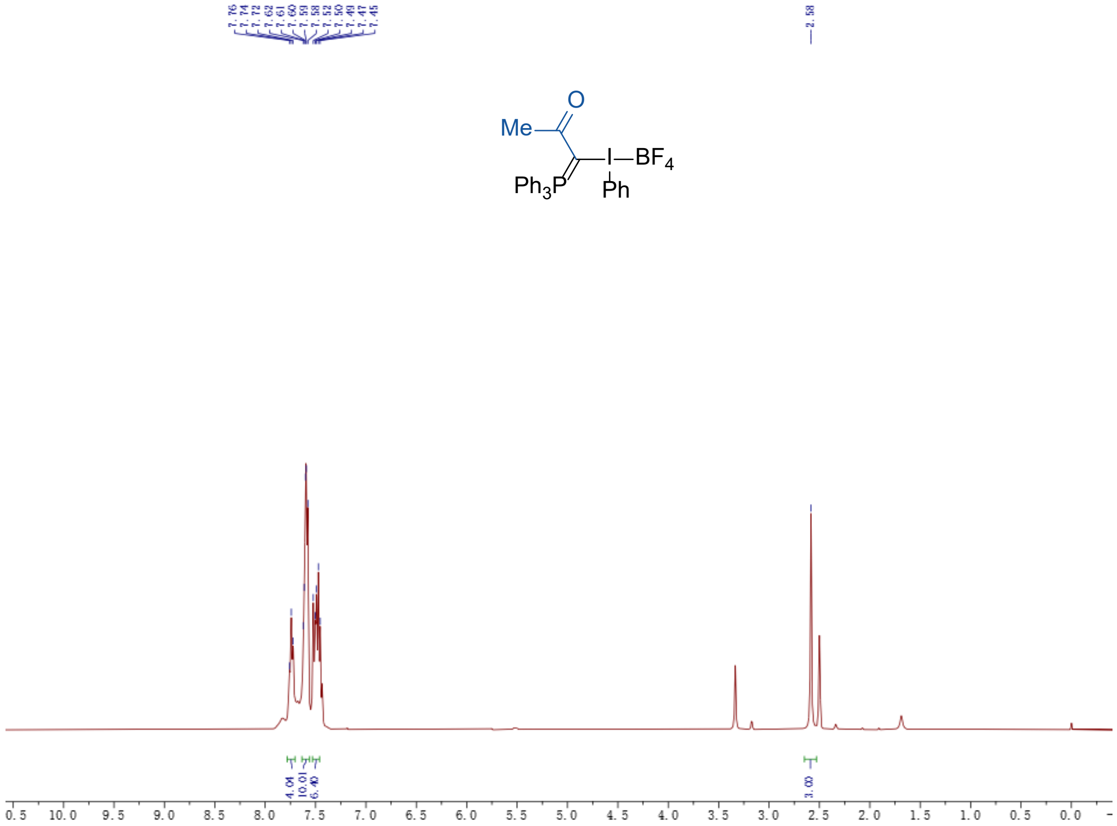

^{1}**H NMR** of the **1a** (400 MHz, 25 °C in DMSO-*d6*)

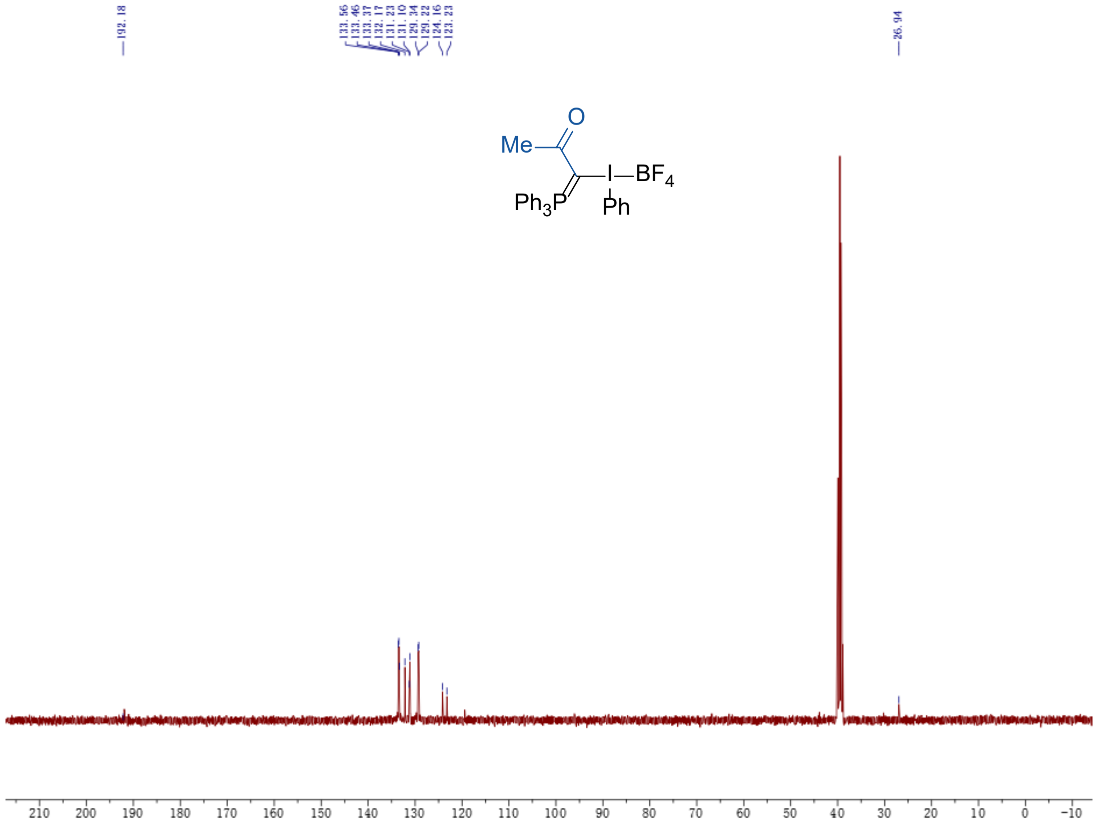

^{13}**C NMR** of the **1a** (101 MHz, 25 °C in DMSO-*d6*)

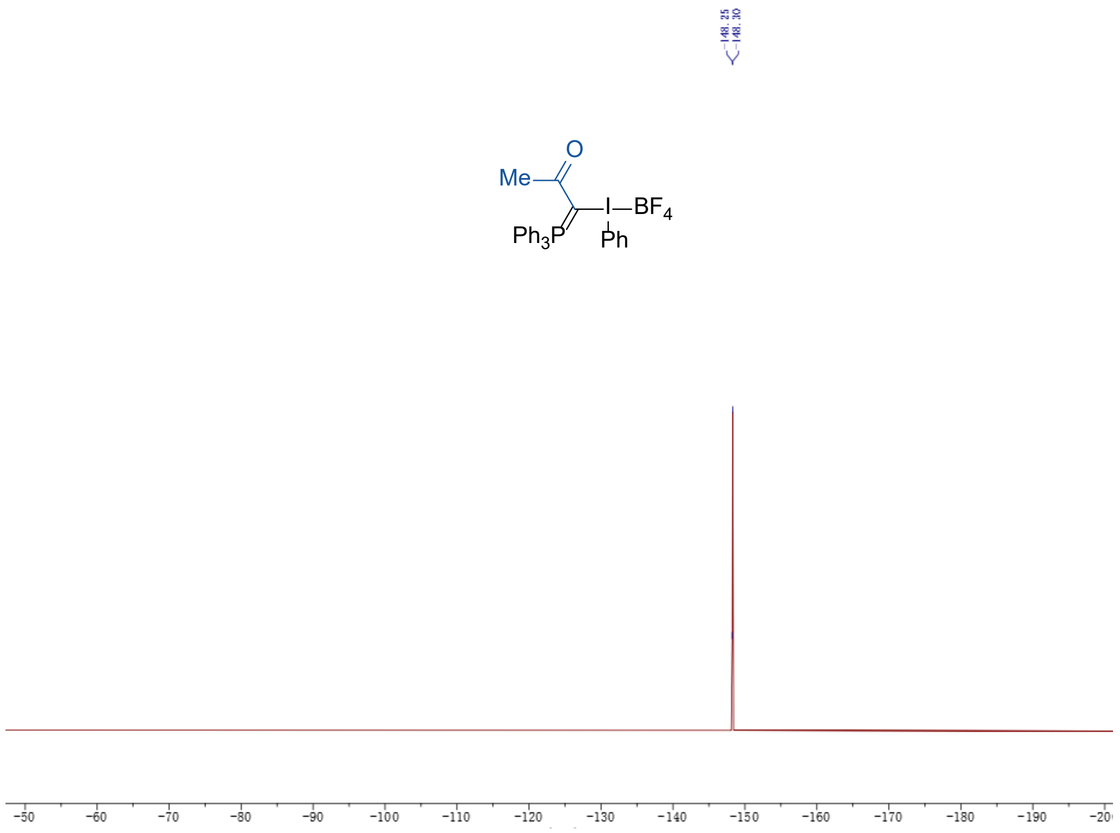

` ^{19}**F NMR** of the **1a** (376 MHz, 25 °C in DMSO-*d6*)

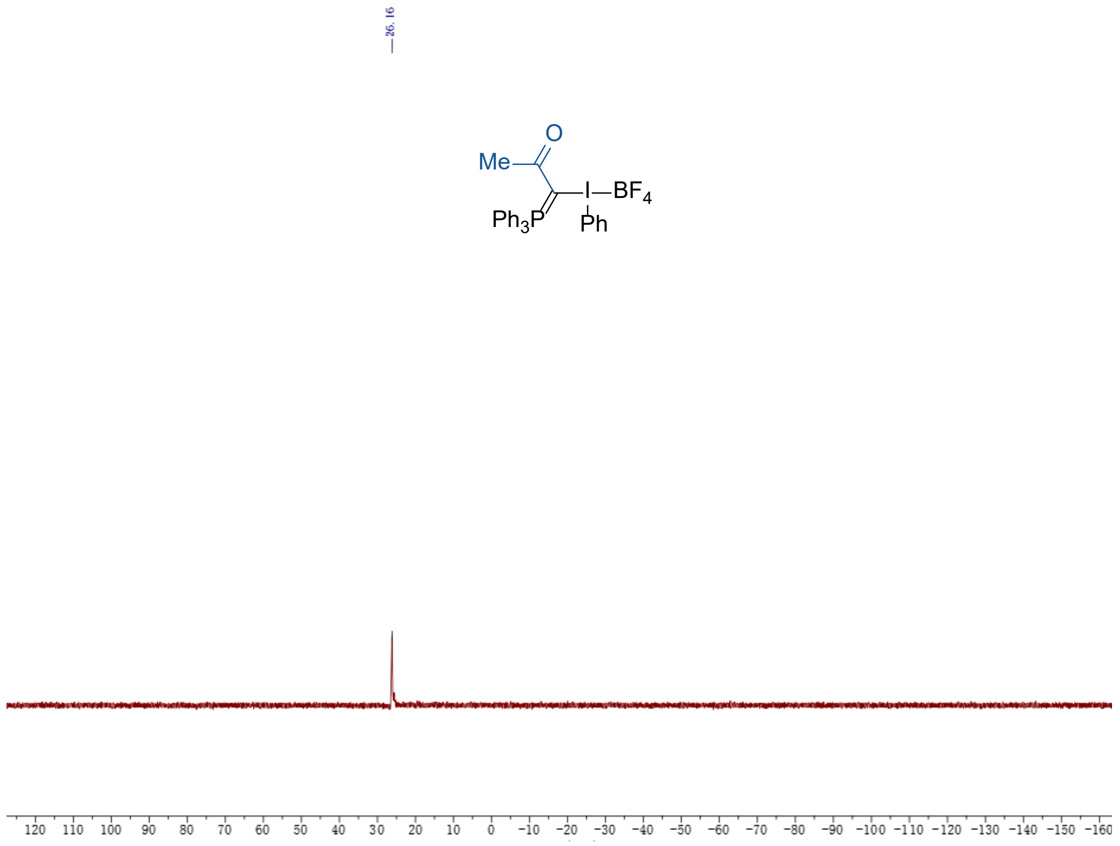

^{31}**P NMR** of the **1a** (162 MHz, 25 °C in DMSO-*d6*)

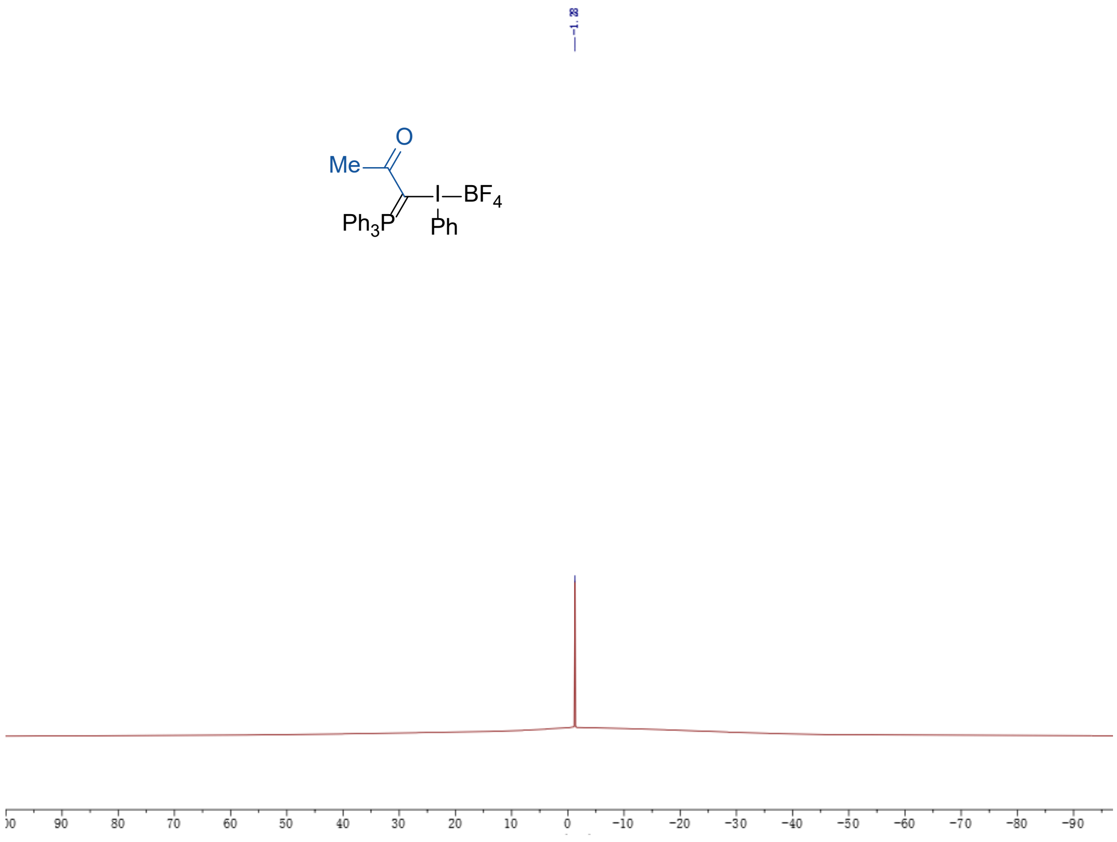

^{11}B NMR of the **1a** (128 MHz, 25 °C in DMSO-*d*$_6$)

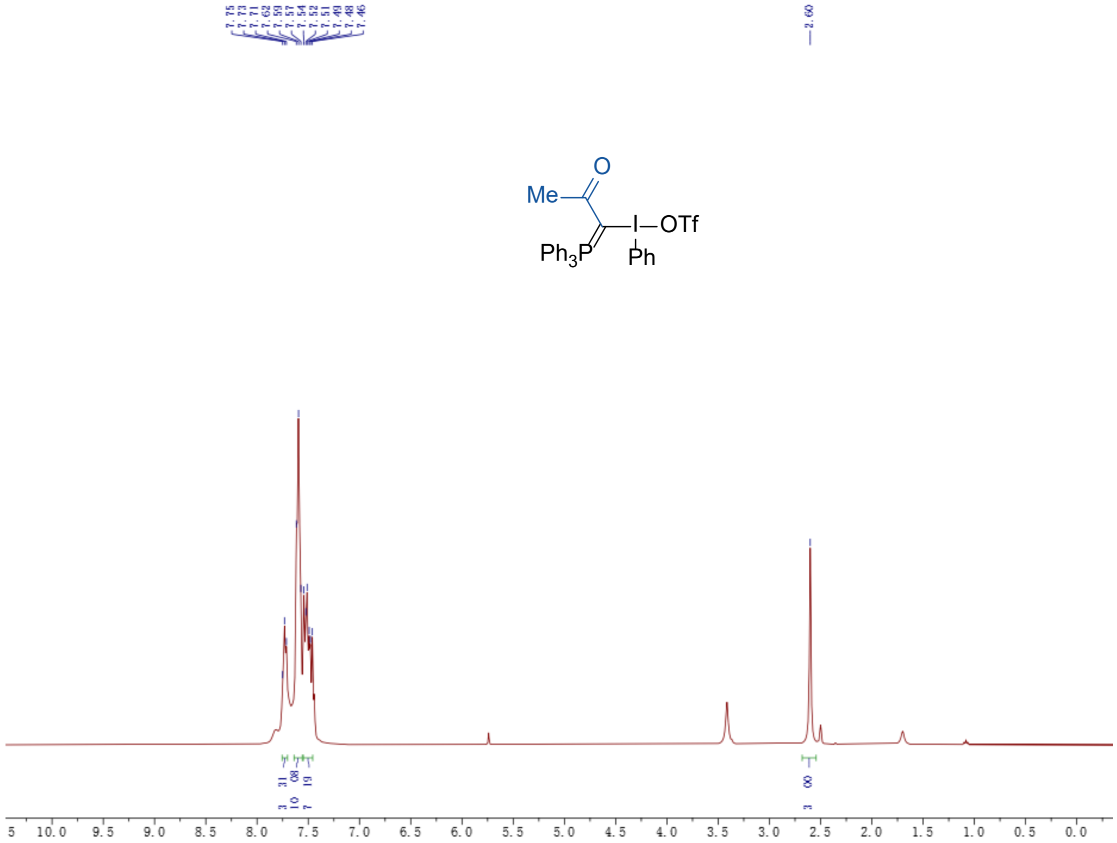

^{1}H NMR of the **1b** (400 MHz, 25 °C in DMSO-*d*$_6$)

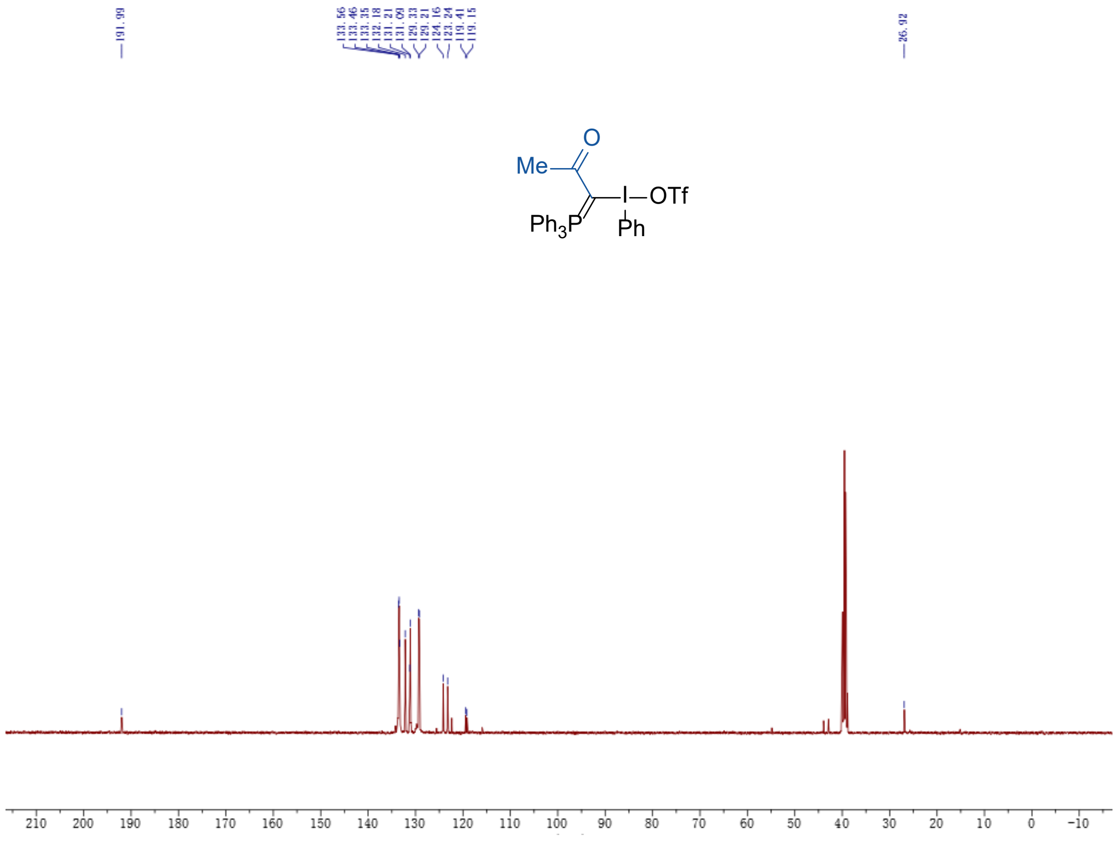

^{13}C **NMR** of the **1b** (100 MHz, 25 °C in DMSO-d_6)

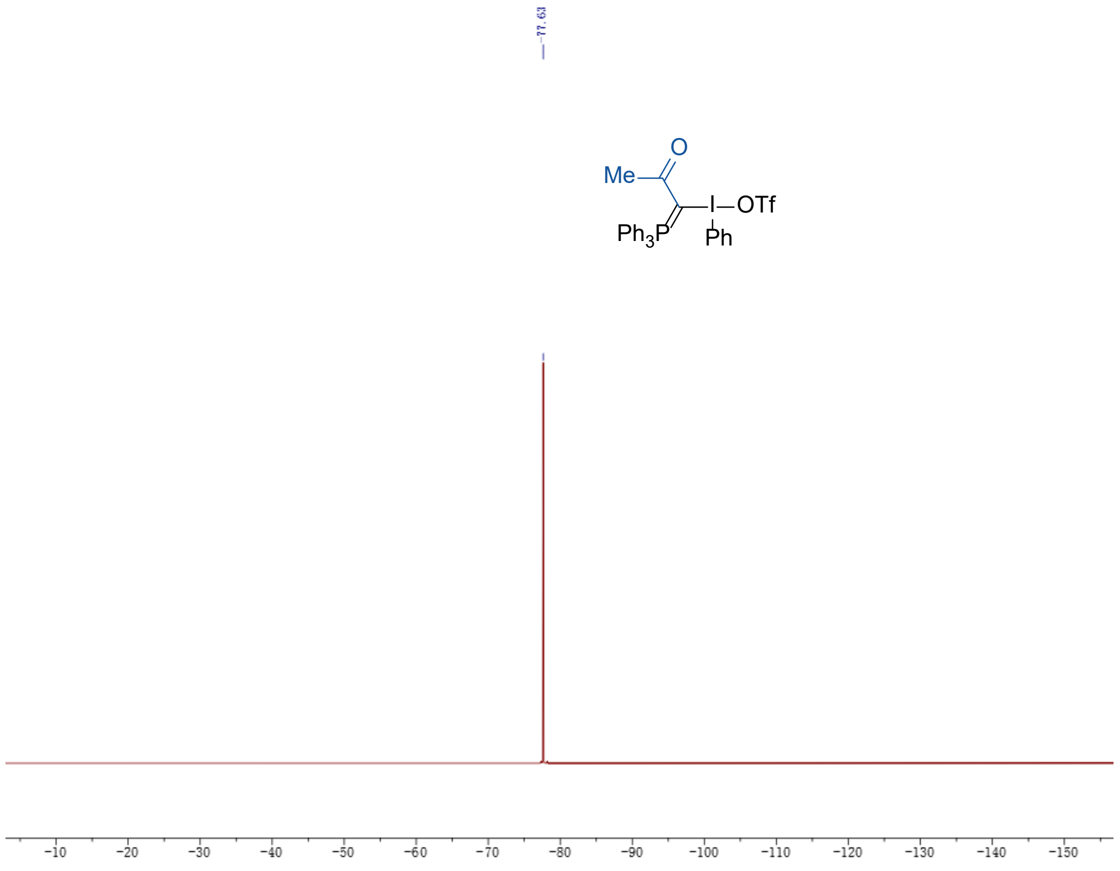

^{19}F **NMR** of the **1b** (376 MHz, 25 °C in DMSO-d_6)

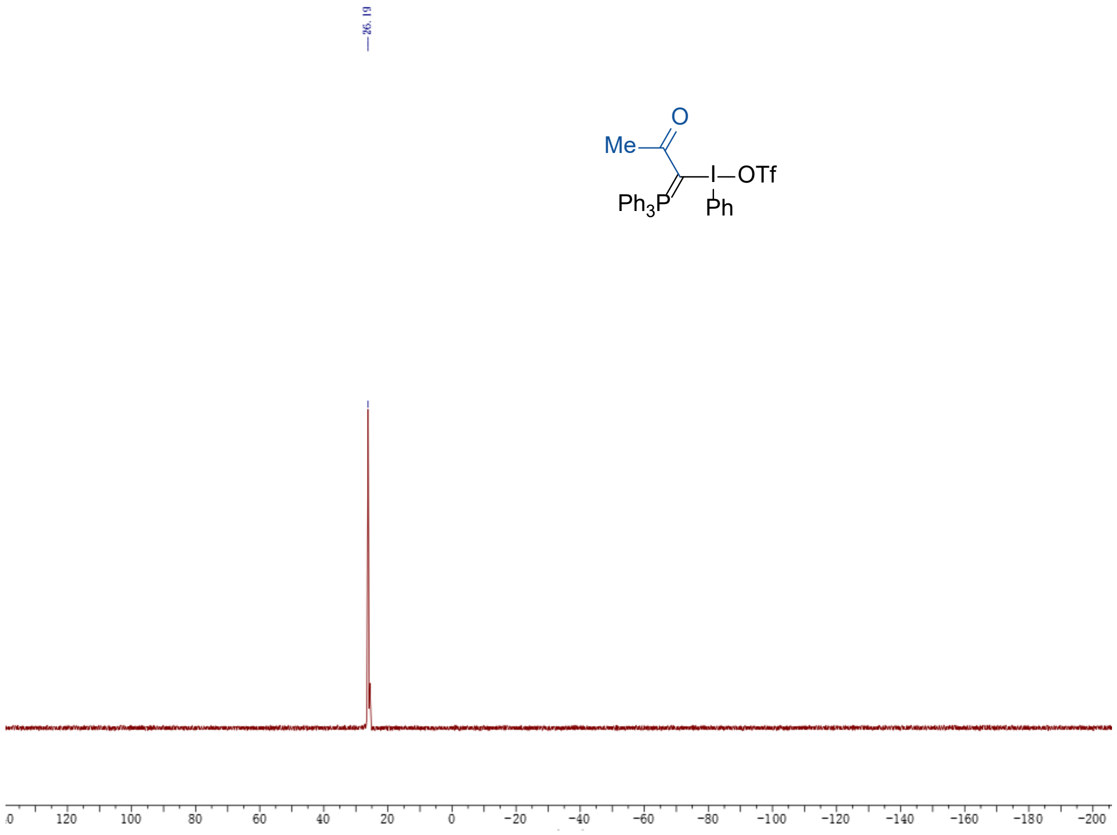

^{31}P NMR of the **1b** (162 MHz, 25 °C in DMSO-d_6)

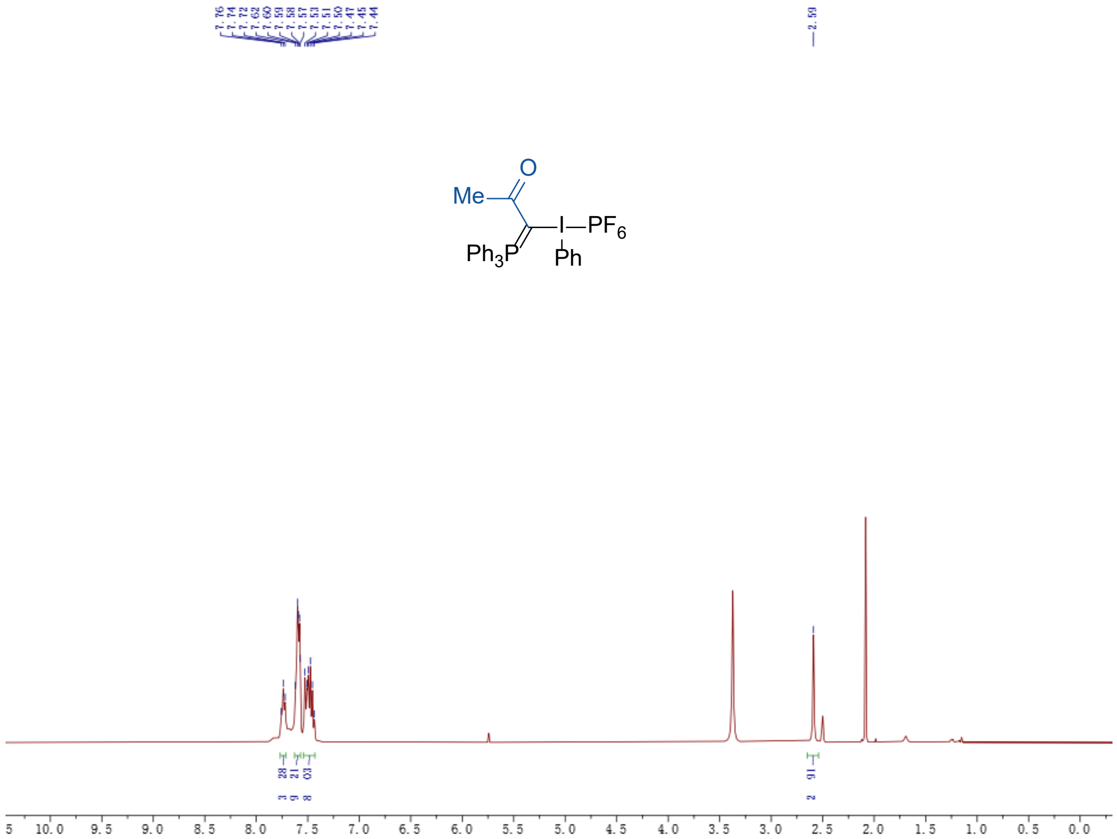

^{1}H NMR of the **1c** (400 MHz, 25 °C in DMSO-d_6)

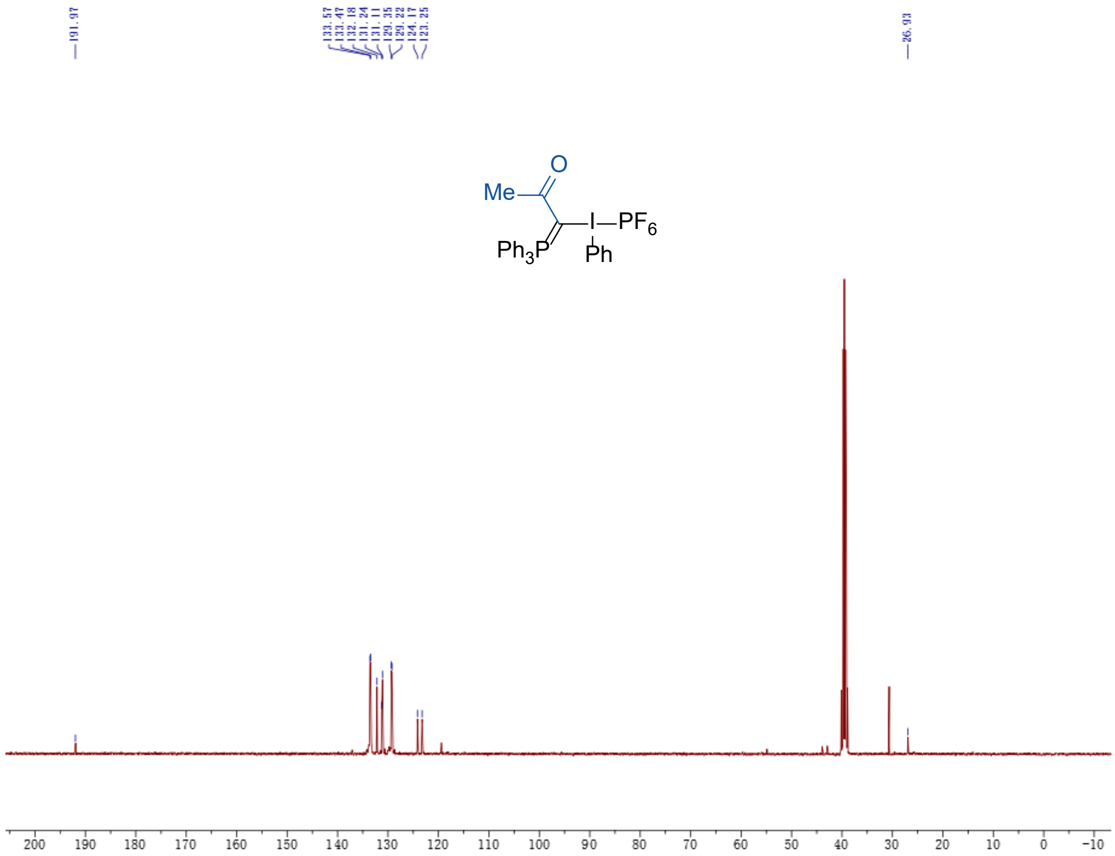

^{13}C NMR of the **1c** (100 MHz, 25 °C in DMSO-d_6)

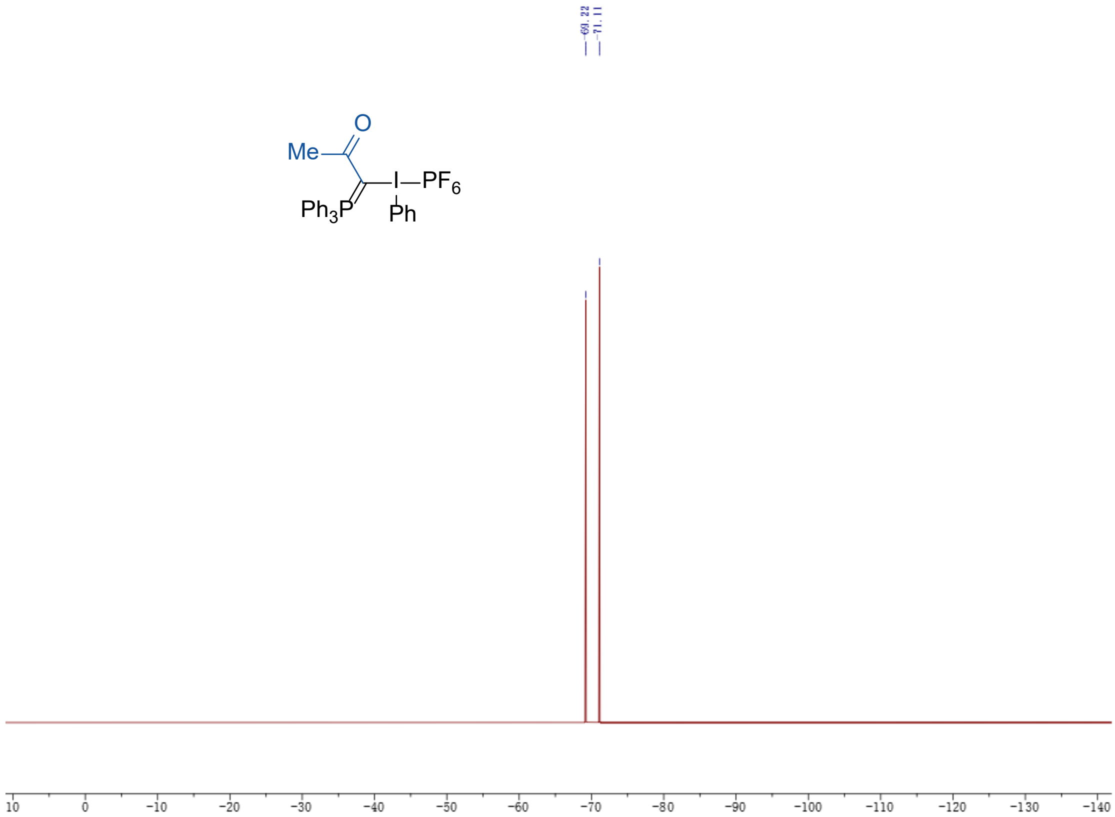

^{19}F NMR of the **1c** (376 MHz, 25 °C in DMSO-d_6)

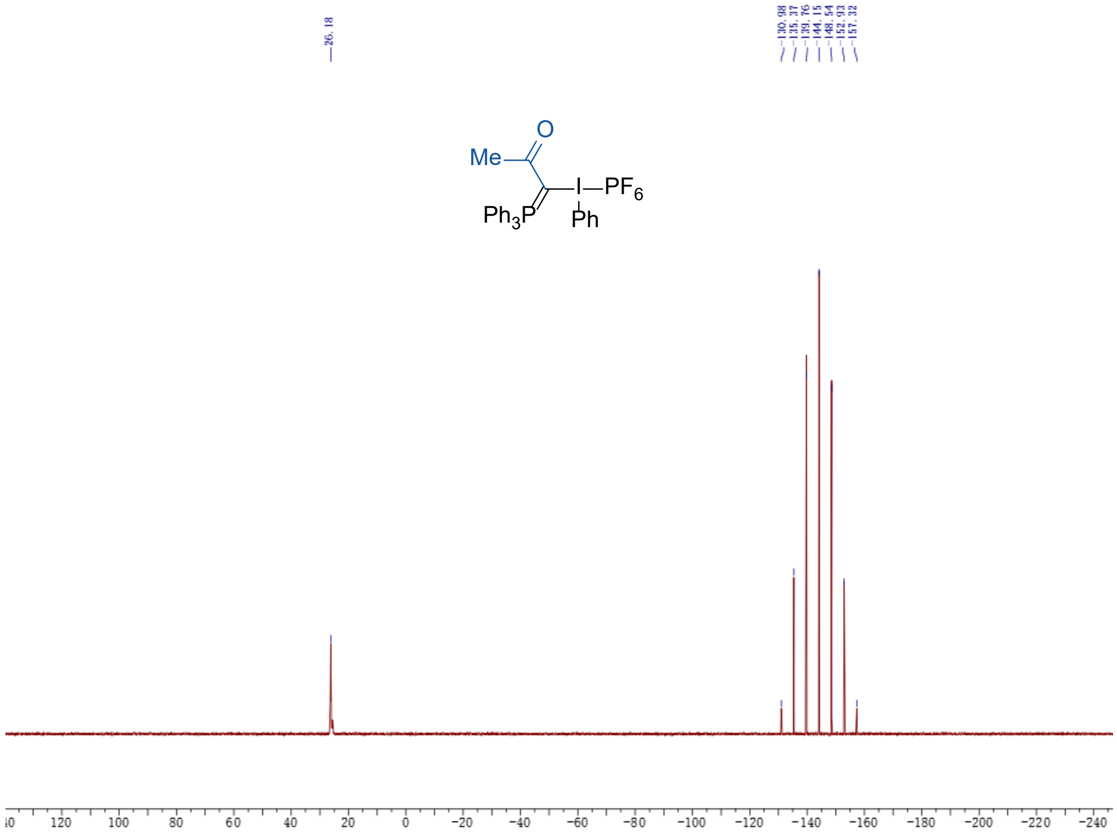

^{31}P NMR of the **1c** (162 MHz, 25 °C in DMSO-*d6*)

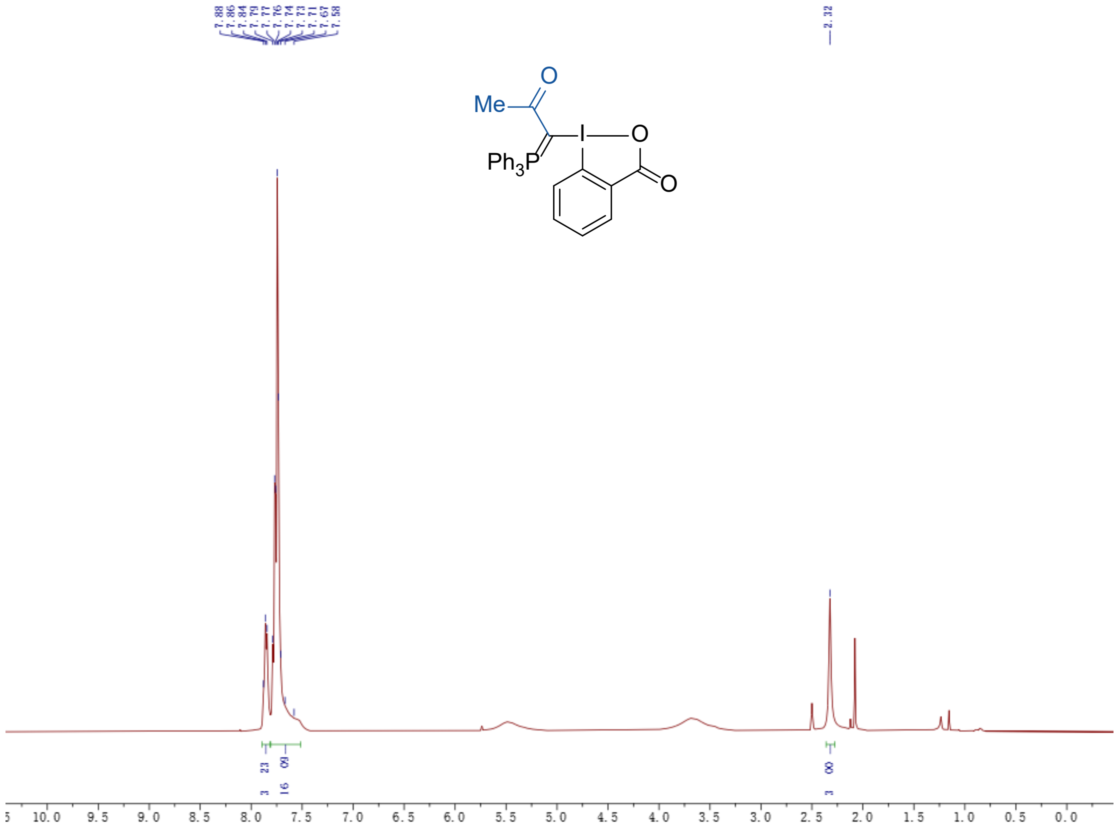

^{1}H NMR of the **1d** (400 MHz, 25 °C in DMSO-*d6*)

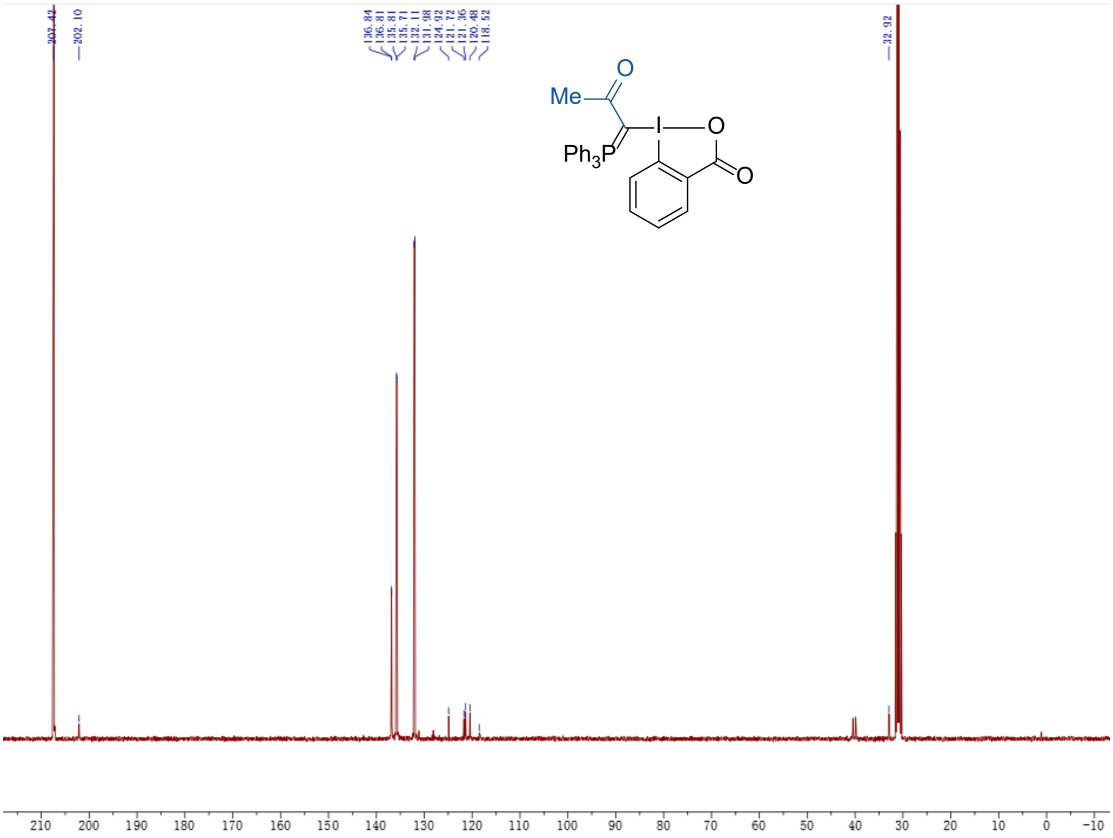

^{13}C NMR of the **1d** (100 MHz, 25 °C in Acetone-*d₆*)

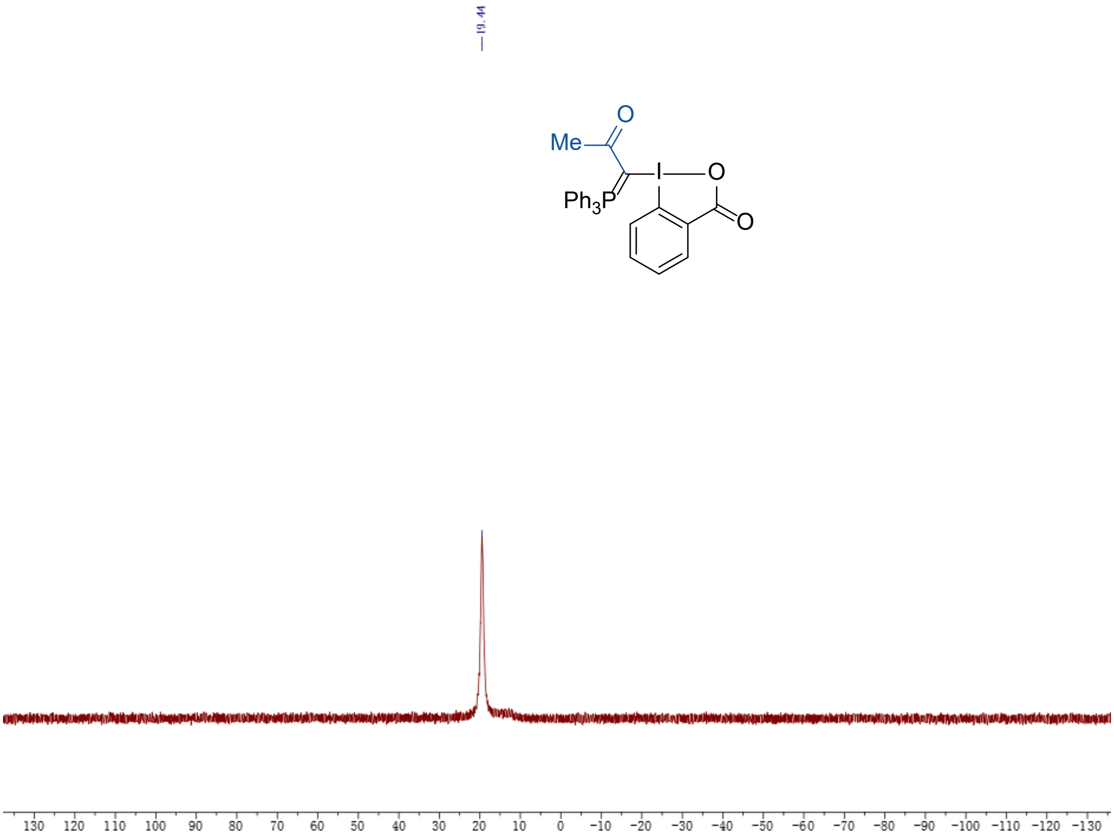

^{31}P NMR of the **1d** (162 MHz, 25 °C in DMSO-*d₆*)

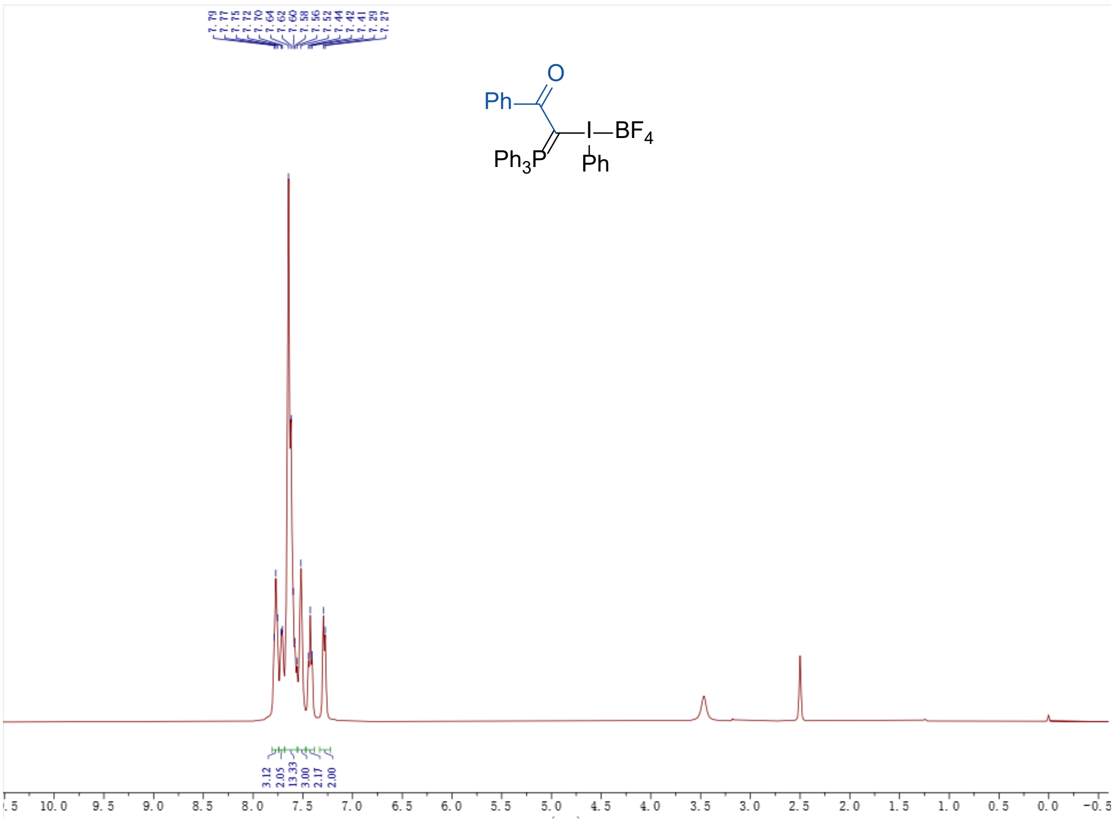

^{1}H NMR of the **1e** (400 MHz, 25 °C in DMSO-*d*$_6$)

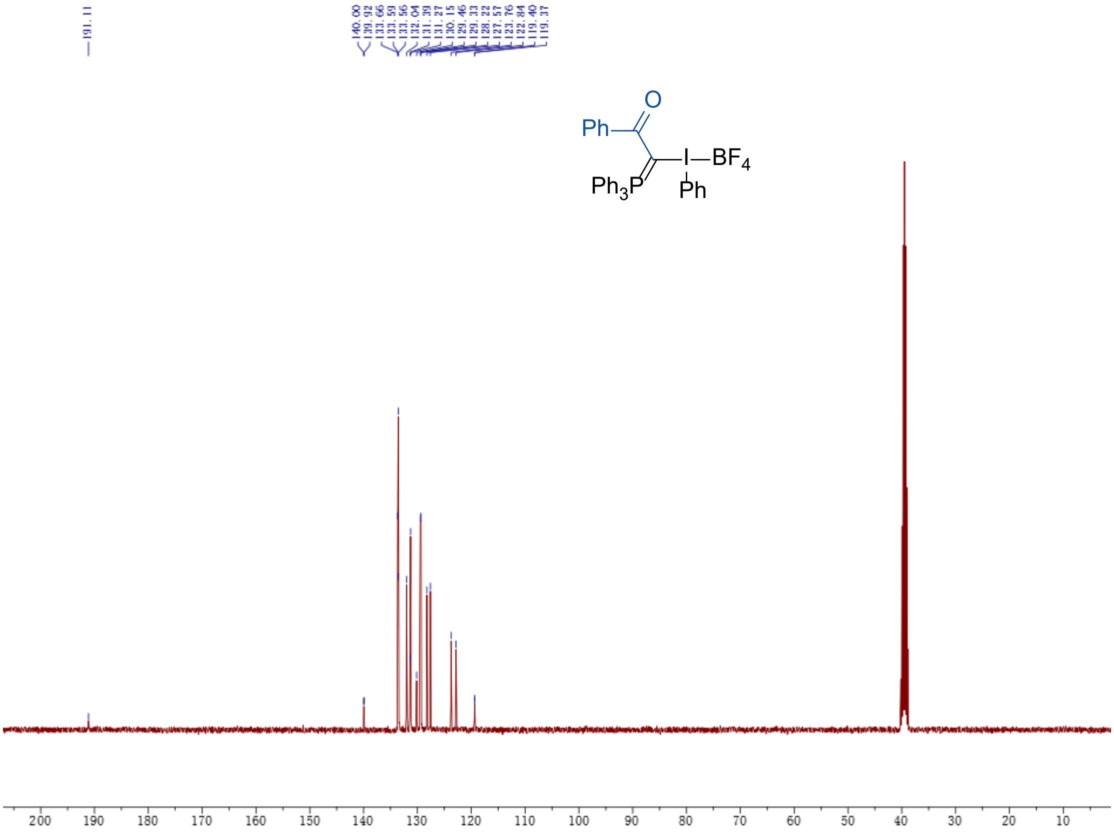

^{13}C NMR of the **1e** (100 MHz, 25 °C in DMSO-*d*$_6$)

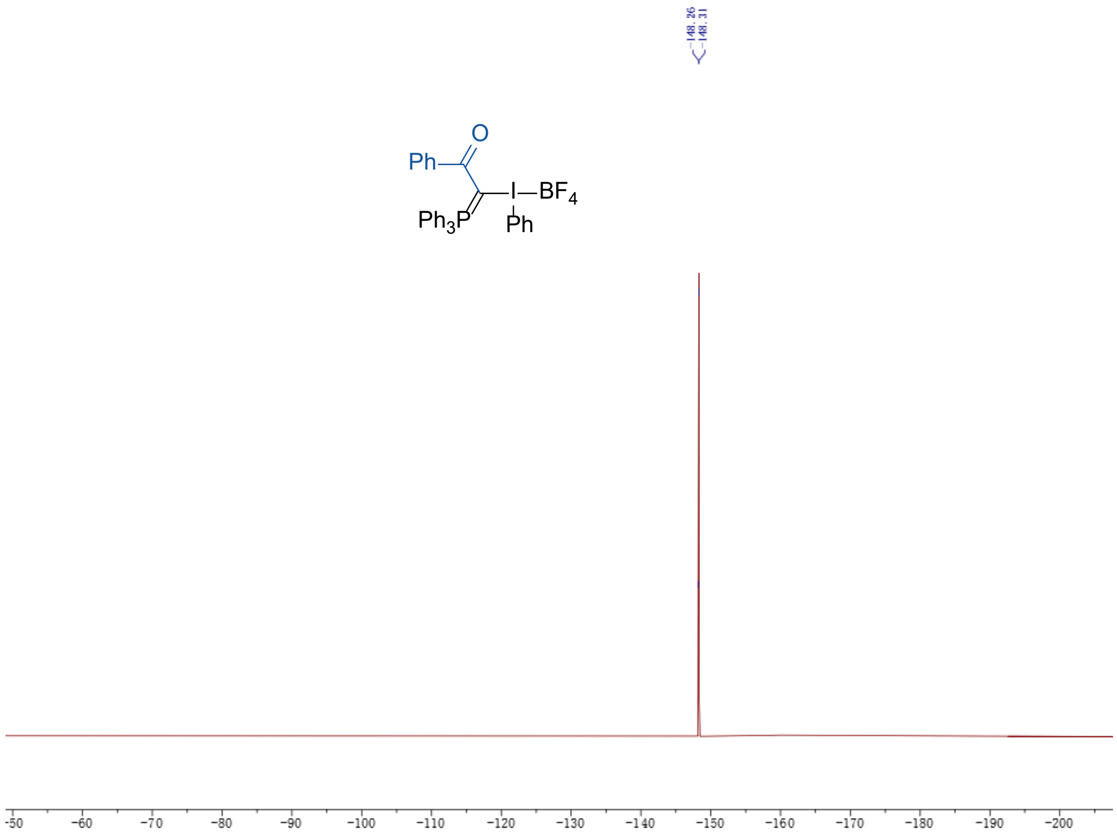

^{19}F NMR of the **1e** (376 MHz, 25 °C in DMSO-*d6*)

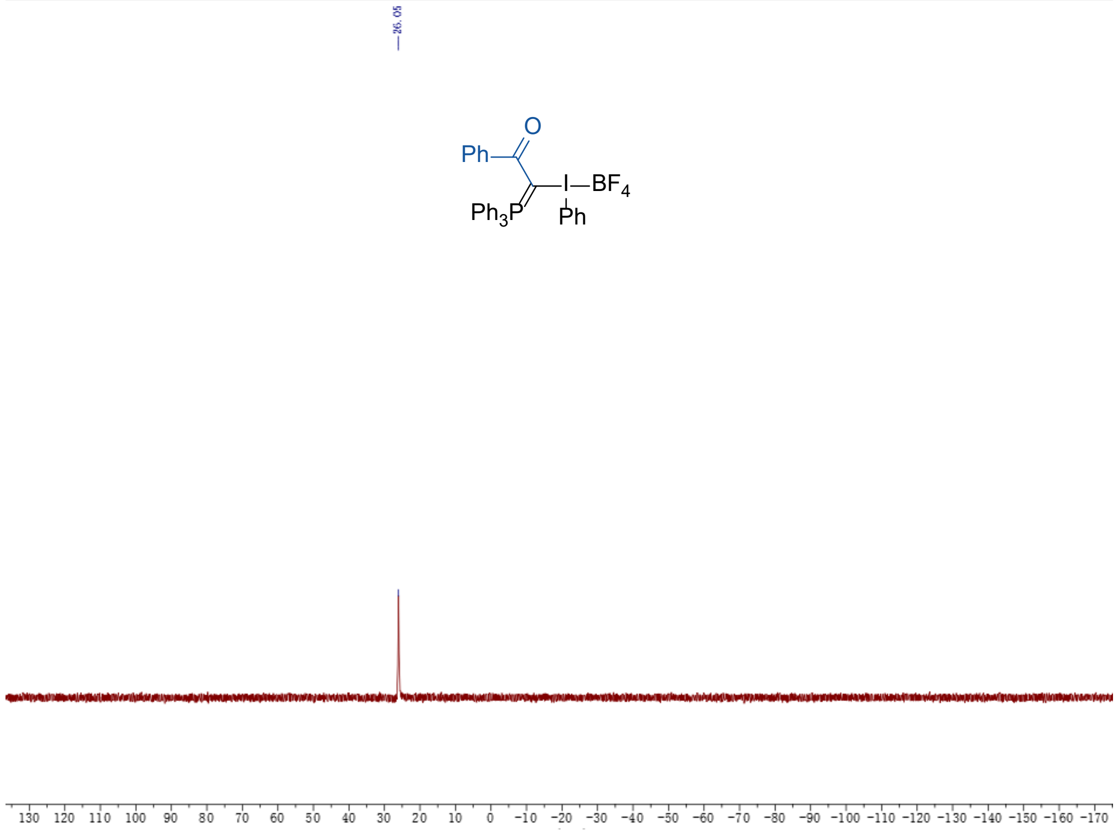

^{31}P NMR of the **1e** (162 MHz, 25 °C in DMSO-*d6*)

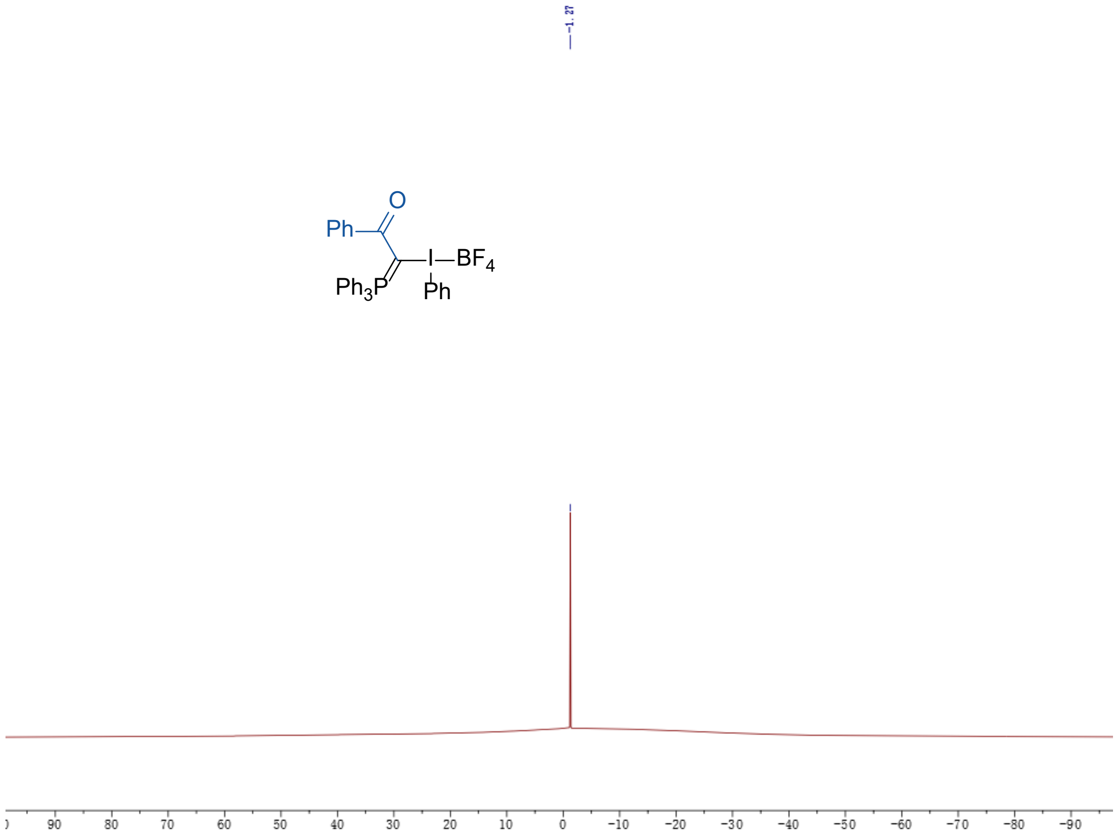

^{11}**B NMR** of the **1e** (128 MHz, 25 °C in DMSO-*d6*)

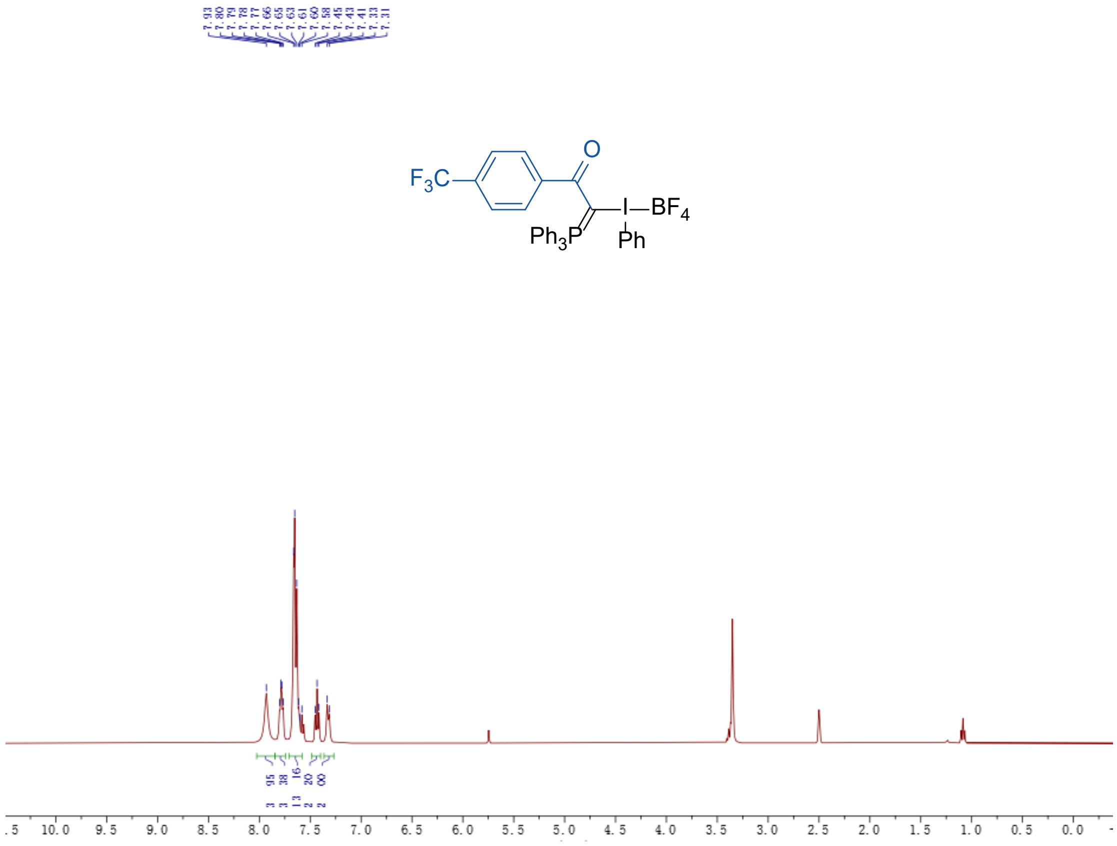

^{1}**H NMR** of the **1f** (400 MHz, 25 °C in DMSO-*d6*)

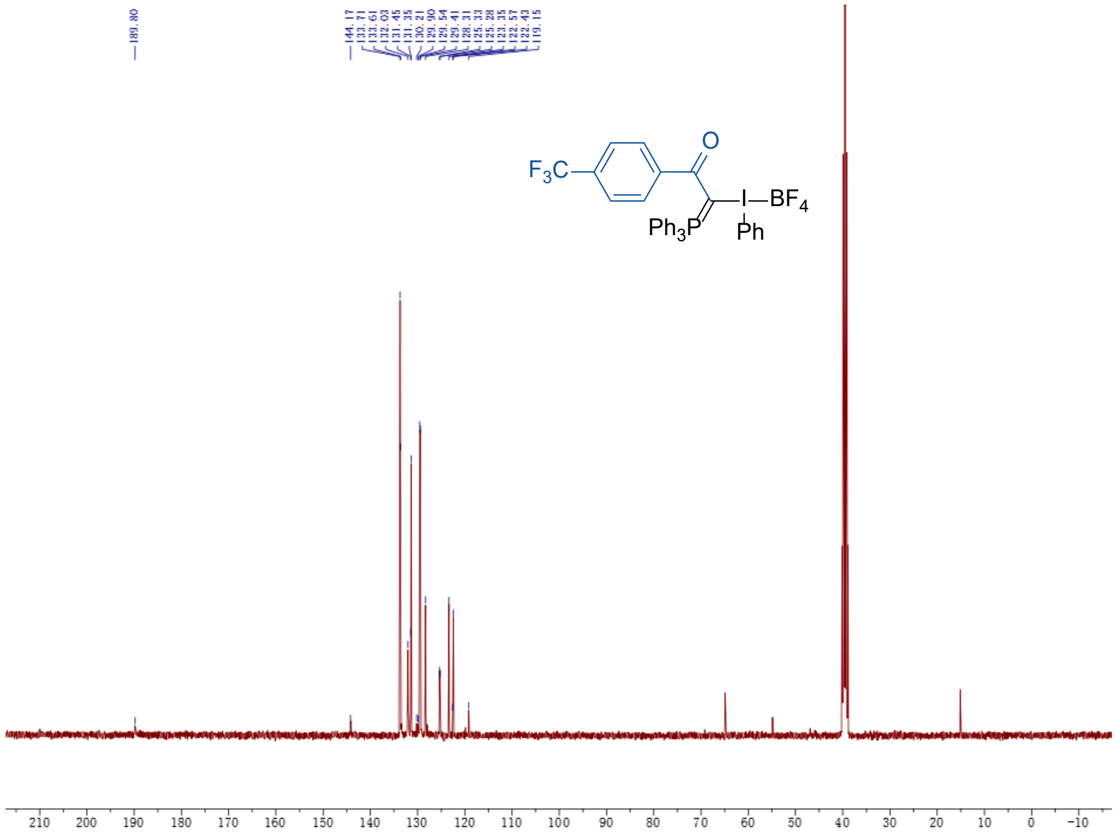

^{13}C NMR of the **1f** (101 MHz, 25 °C in DMSO-*d6*)

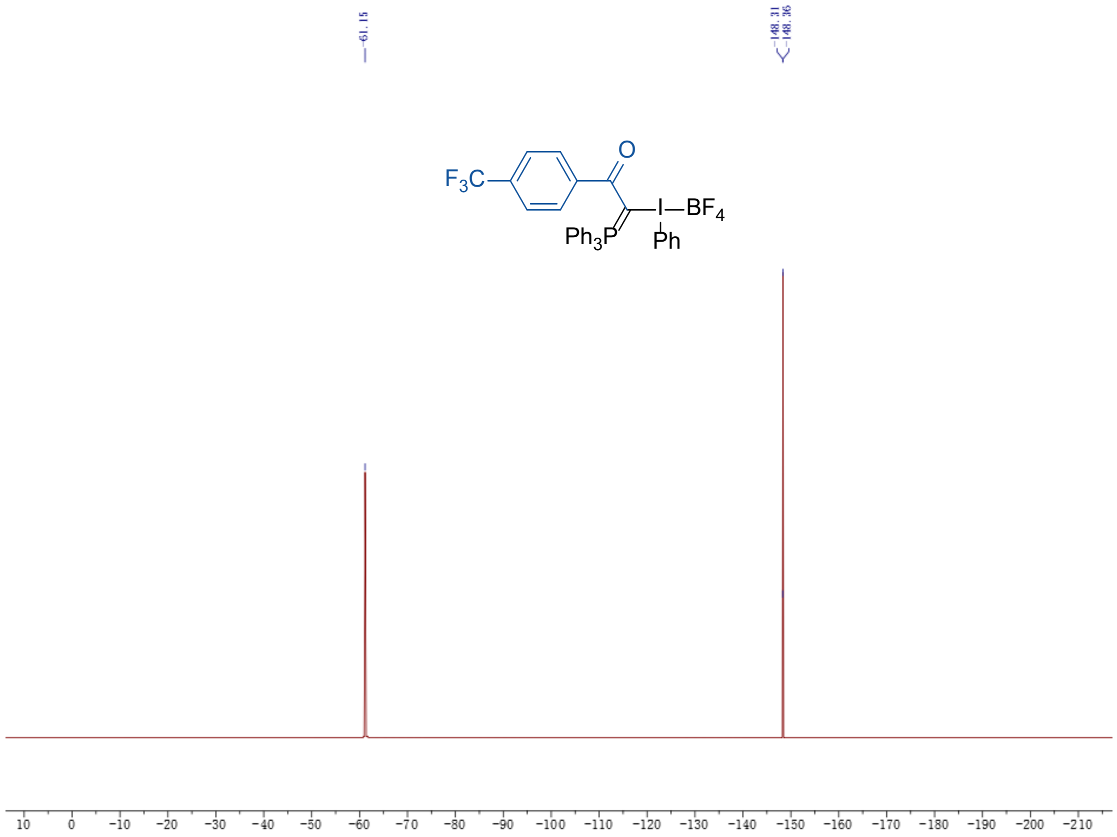

^{19}F NMR of the **1f** (376 MHz, 25 °C in DMSO-*d6*)

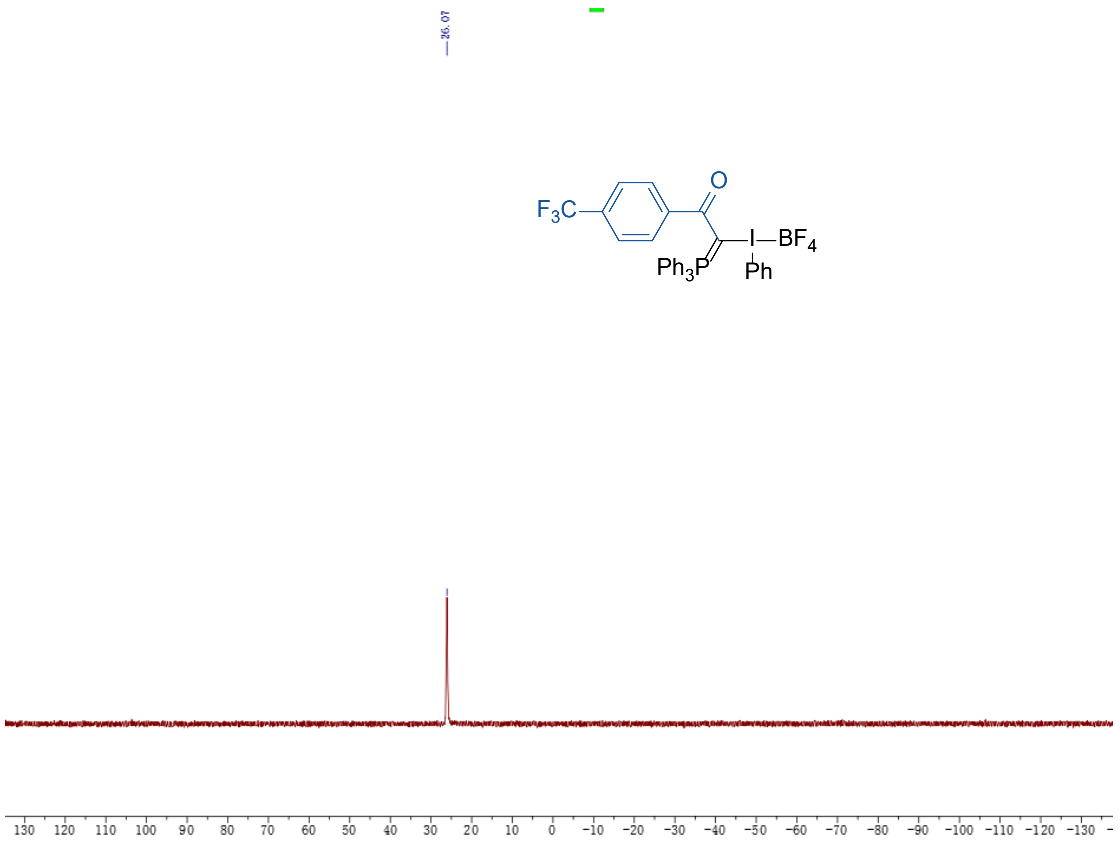

^{31}P NMR of the **1f** (162 MHz, 25 °C in DMSO-*d6*)

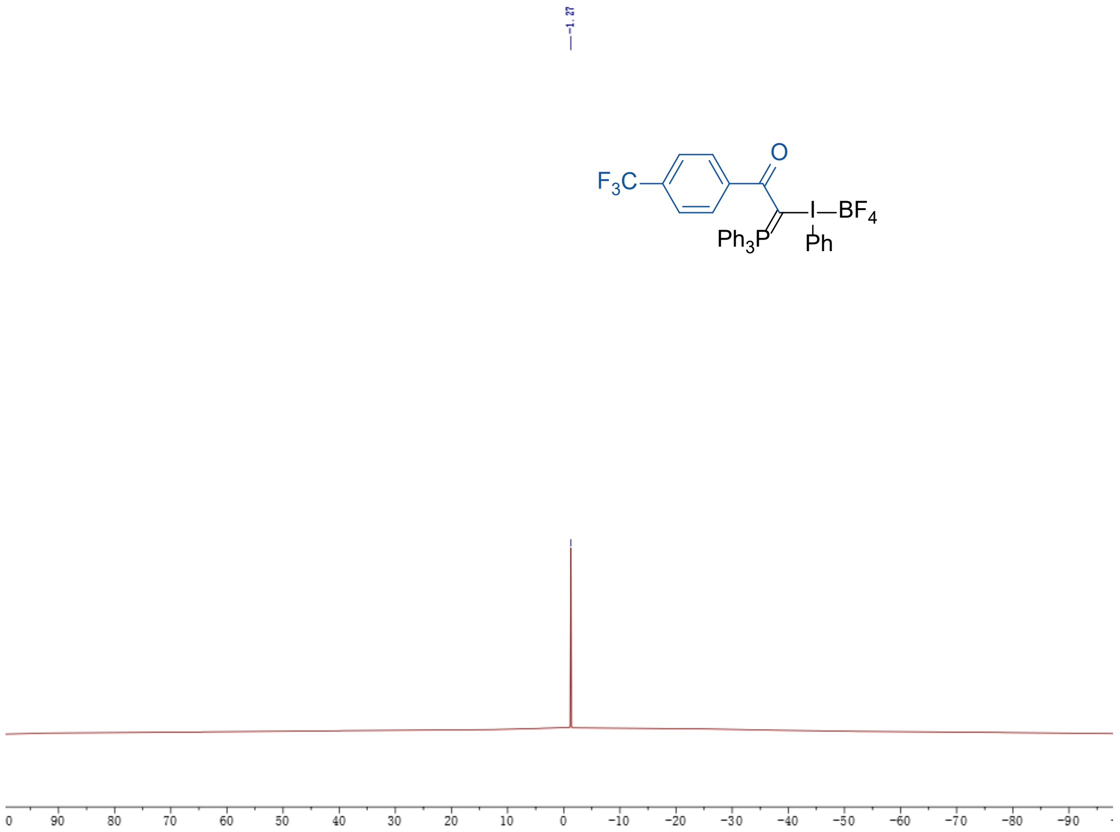

^{11}B NMR of the **1f** (128 MHz, 25 °C in DMSO-*d6*)

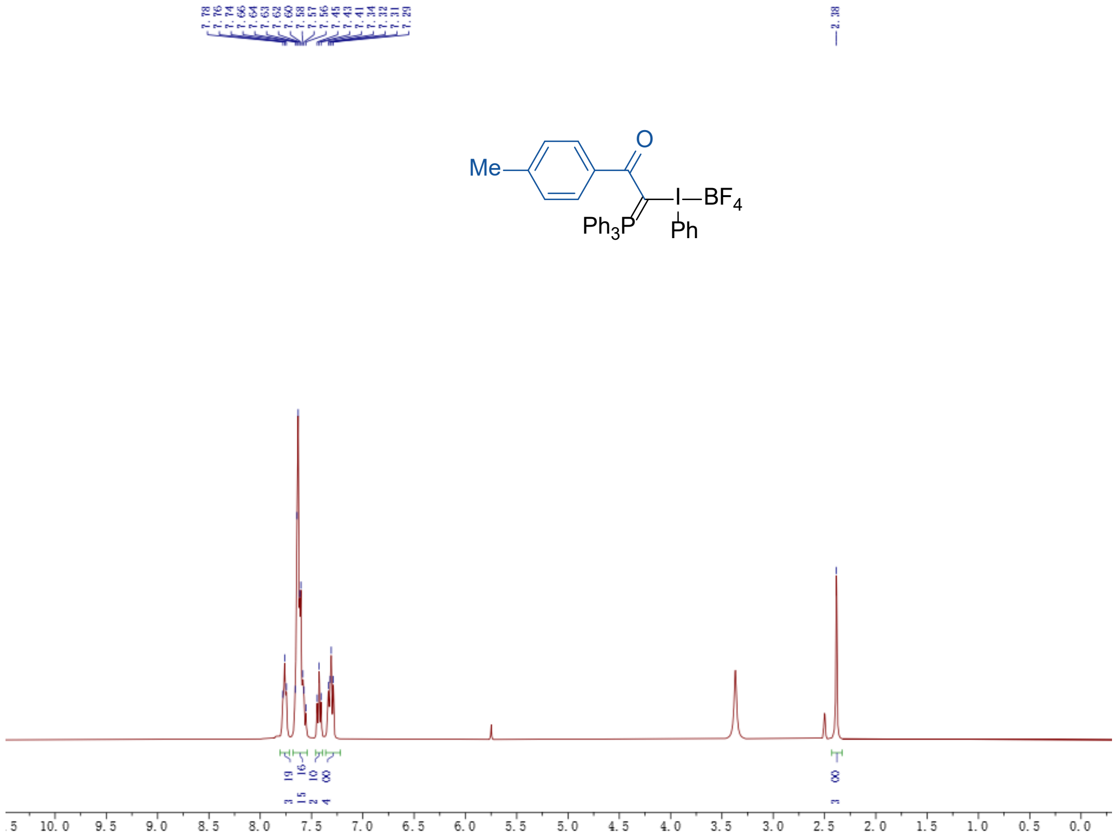

^{1}H NMR of the **1g** (400 MHz, 25 °C in DMSO-*d6*)

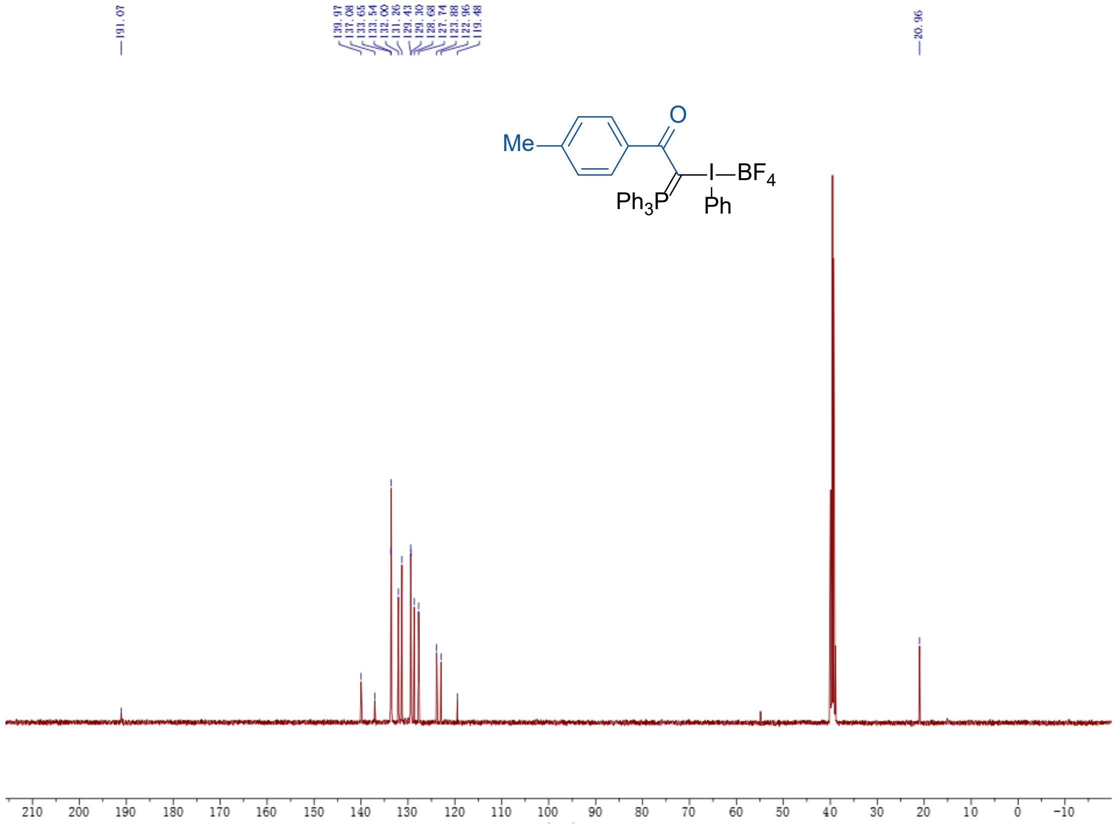

^{13}C NMR of the **1g** (101 MHz, 25 °C in DMSO-*d6*)

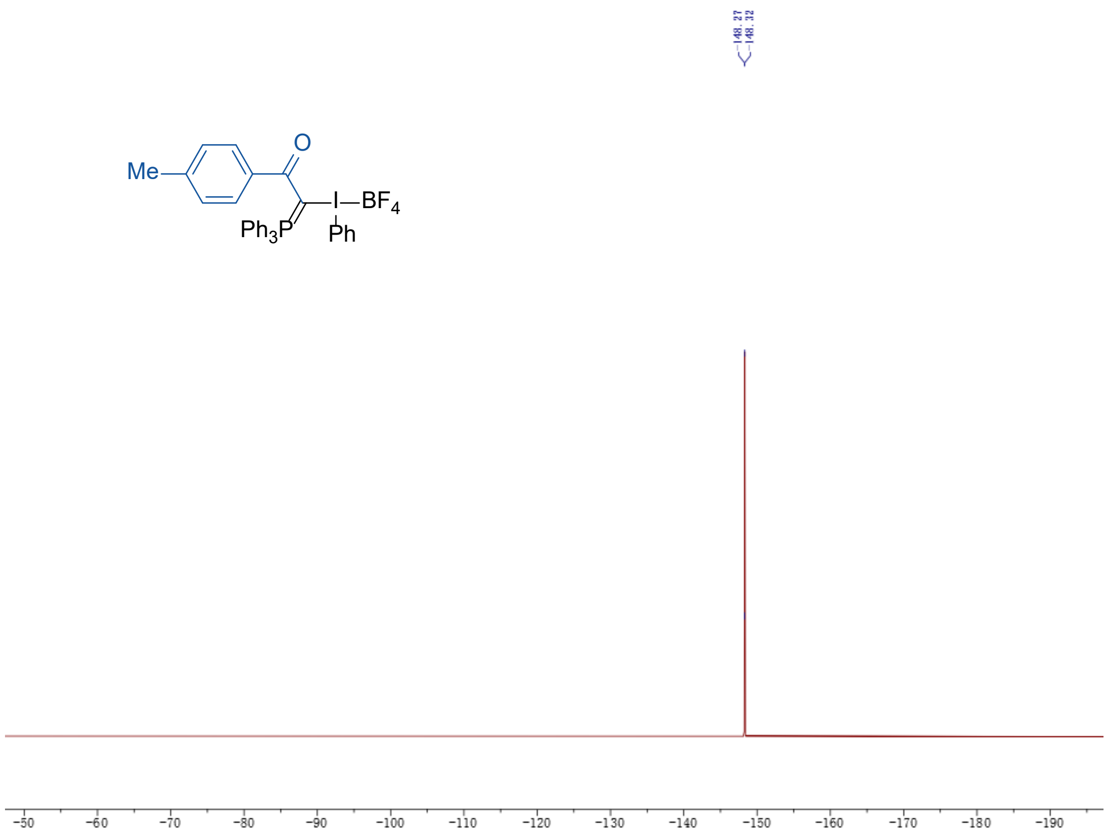

^{19}F NMR of the **1g** (376 MHz, 25 °C in DMSO-*d6*)

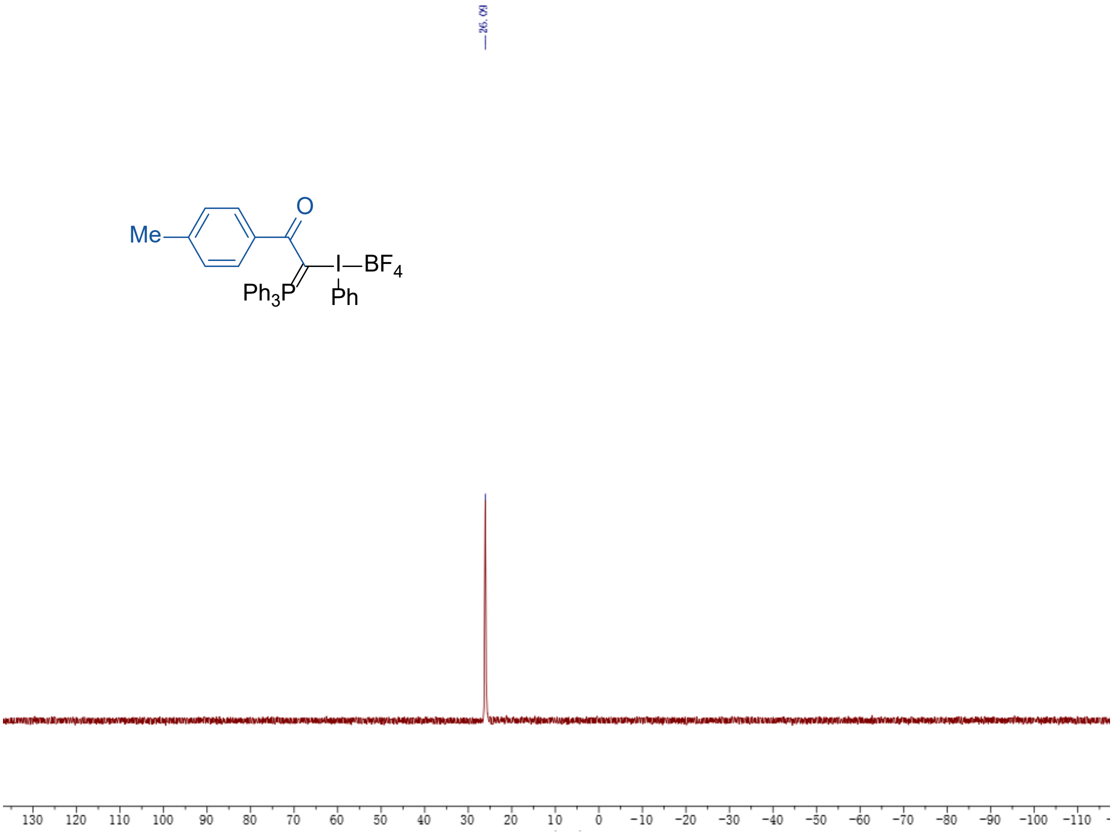

^{31}P NMR of the **1g** (162 MHz, 25 °C in DMSO-*d6*)

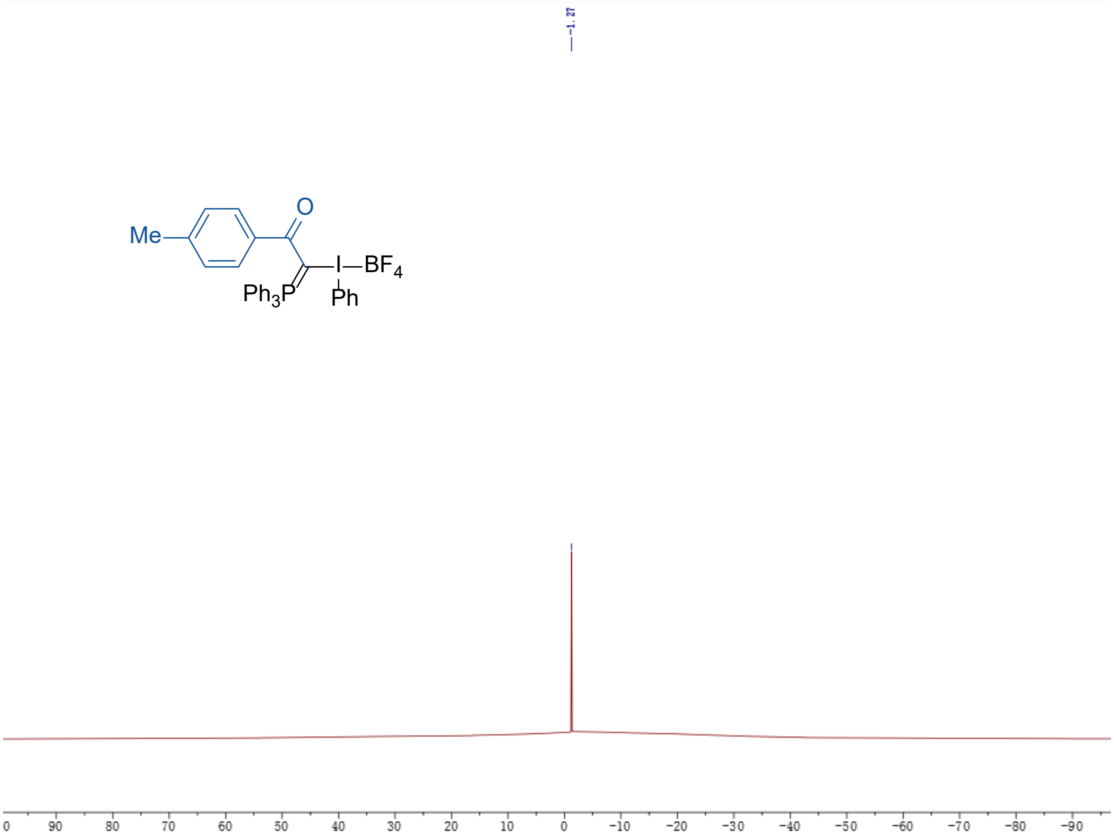

^{11}**B NMR** of the **1g** (128 MHz, 25 °C in DMSO-d_6)

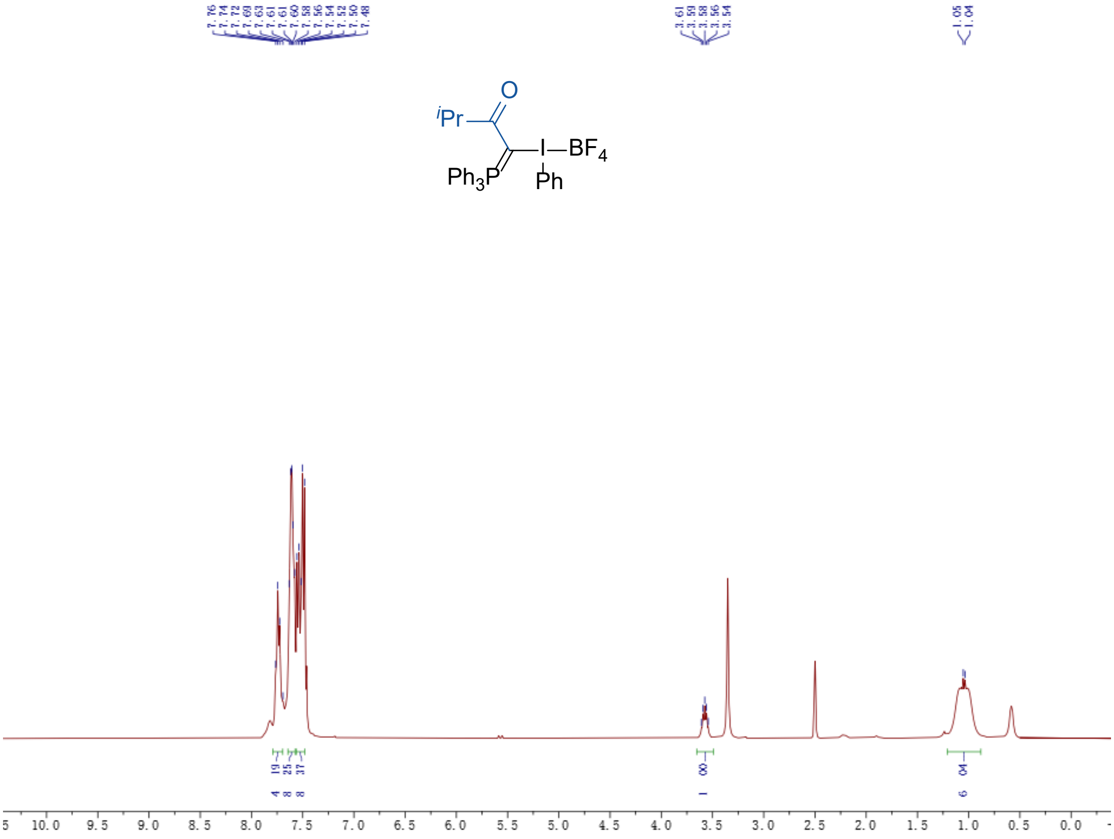

^{1}**H NMR** of the **1h** (400 MHz, 25 °C in DMSO-d_6)

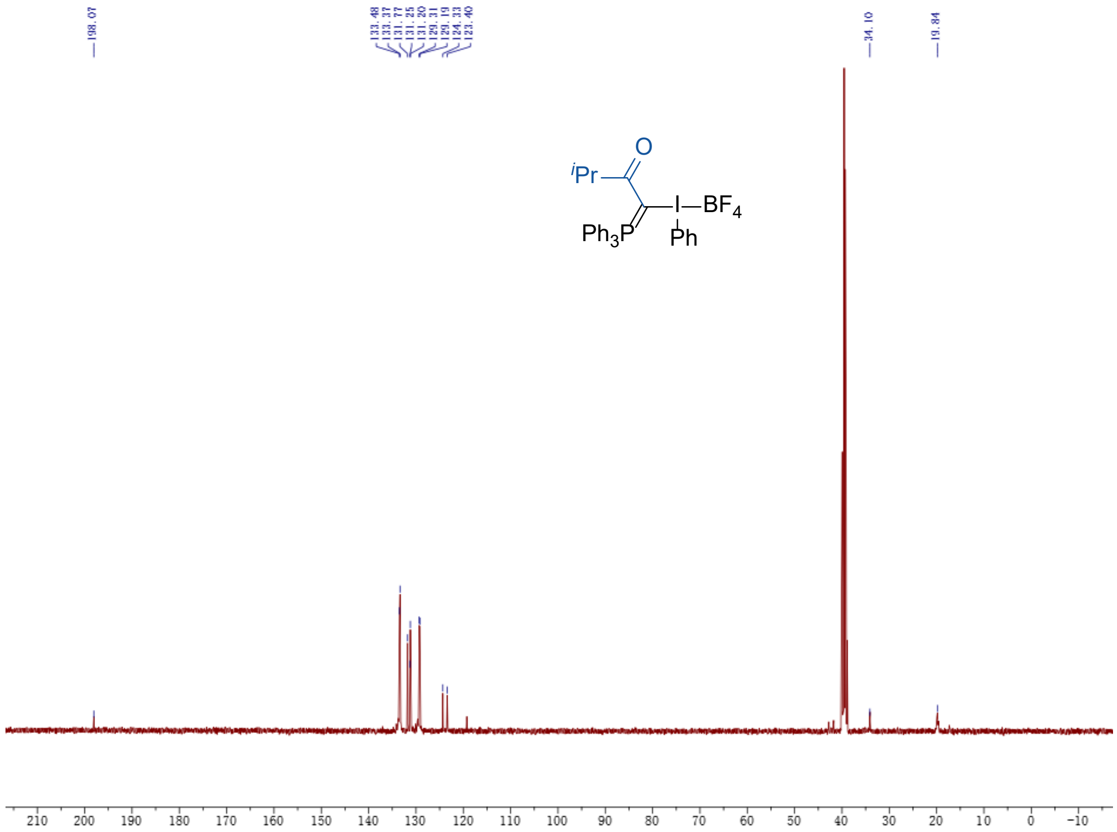

^{13}C NMR of the **1h** (101 MHz, 25 °C in DMSO-*d*6)

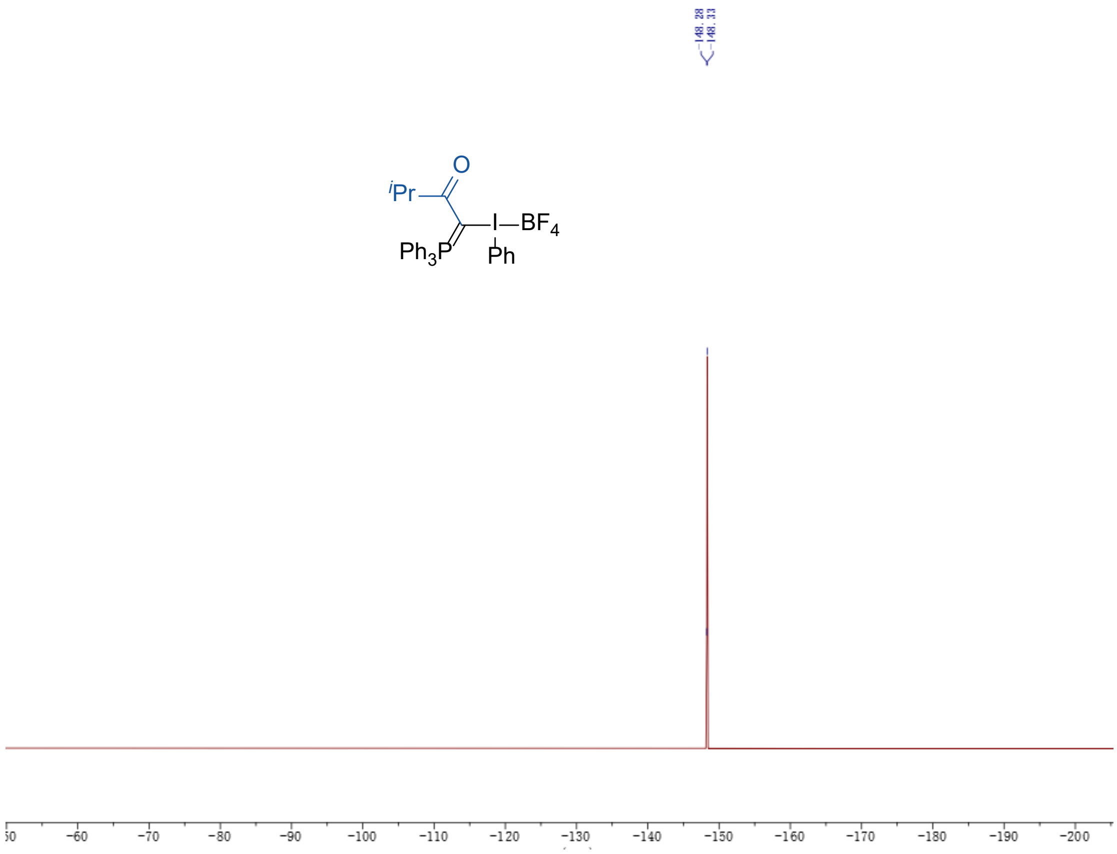

^{19}F NMR of the **1h** (376 MHz, 25 °C in DMSO-*d*6)

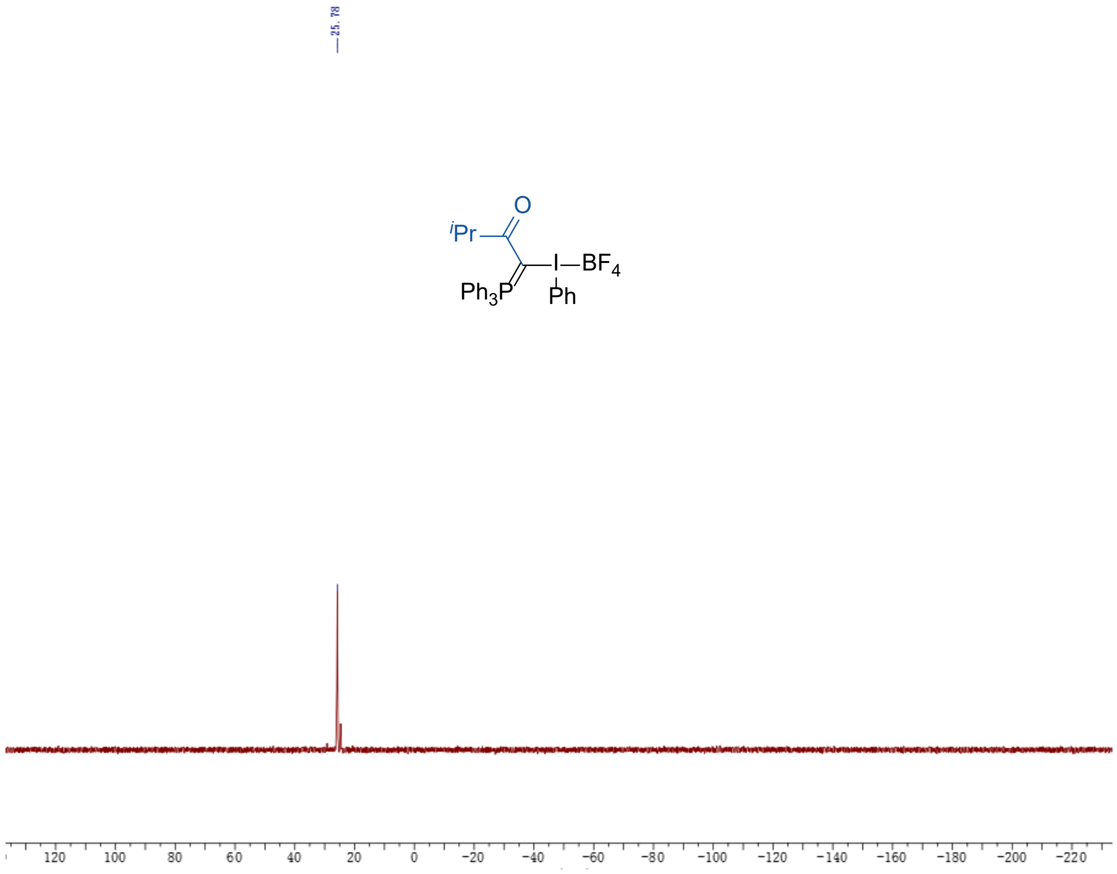

^{31}P NMR of the **1h** (162 MHz, 25 °C in DMSO-*d6*)

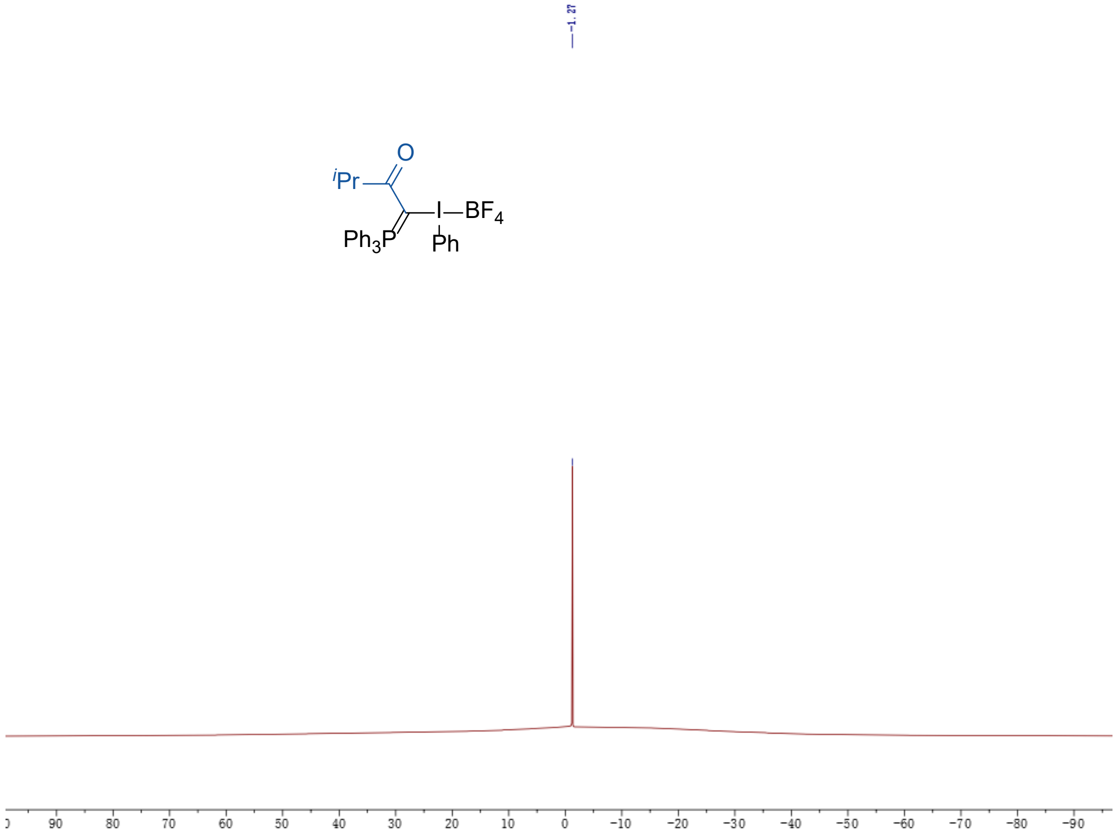

^{11}B NMR of the **1h** (128 MHz, 25 °C in DMSO-*d6*)

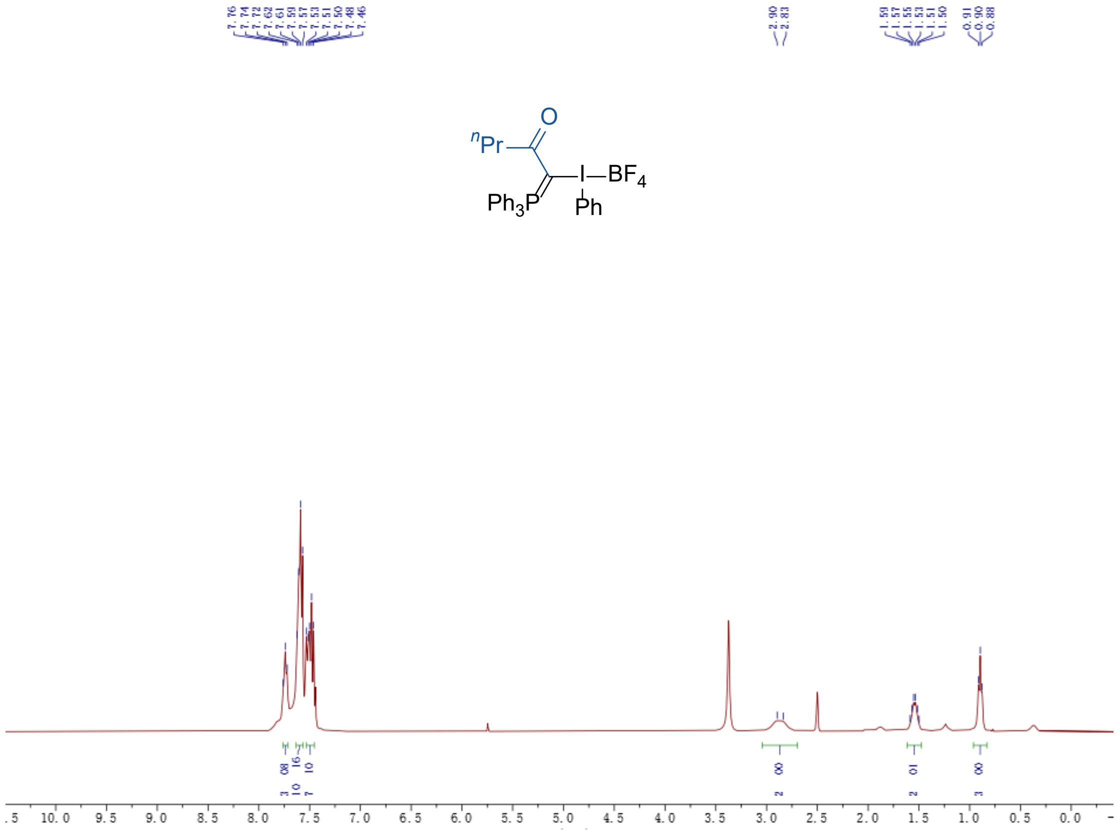

¹H NMR of the **1i** (400 MHz, 25 °C in DMSO-*d6*)

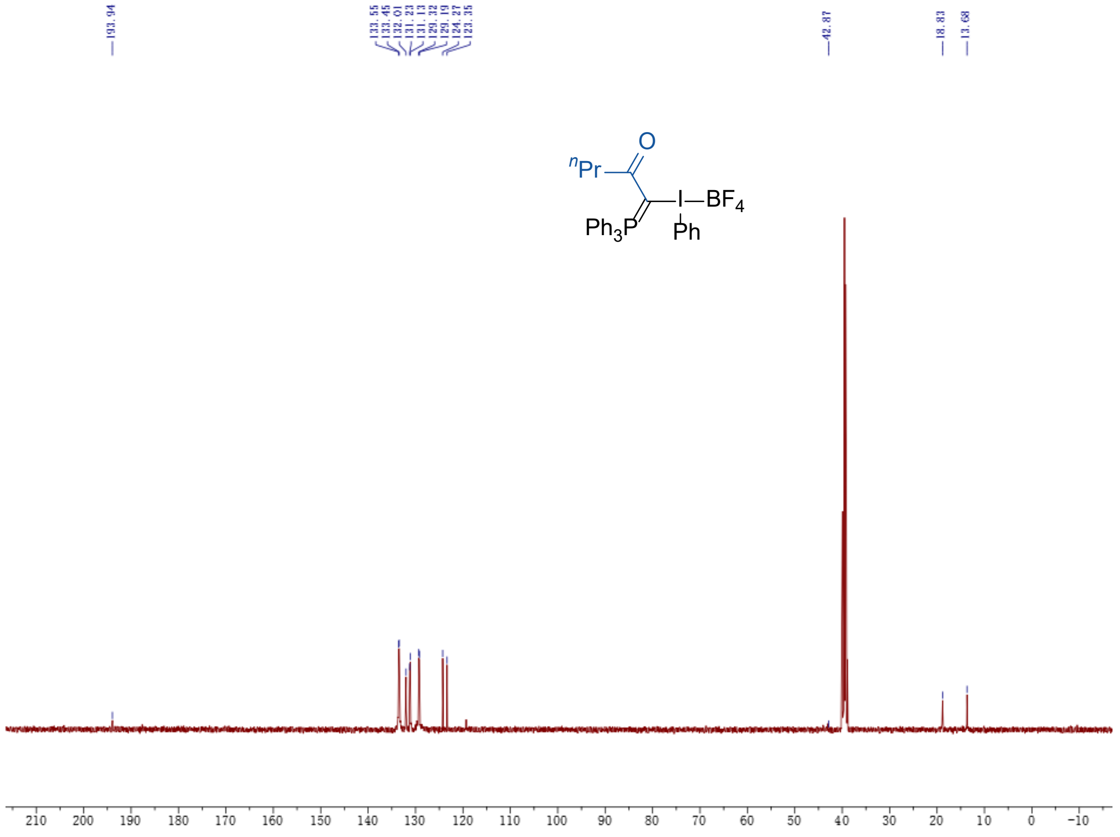

¹³C NMR of the **1i** (101 MHz, 25 °C in DMSO-*d6*)

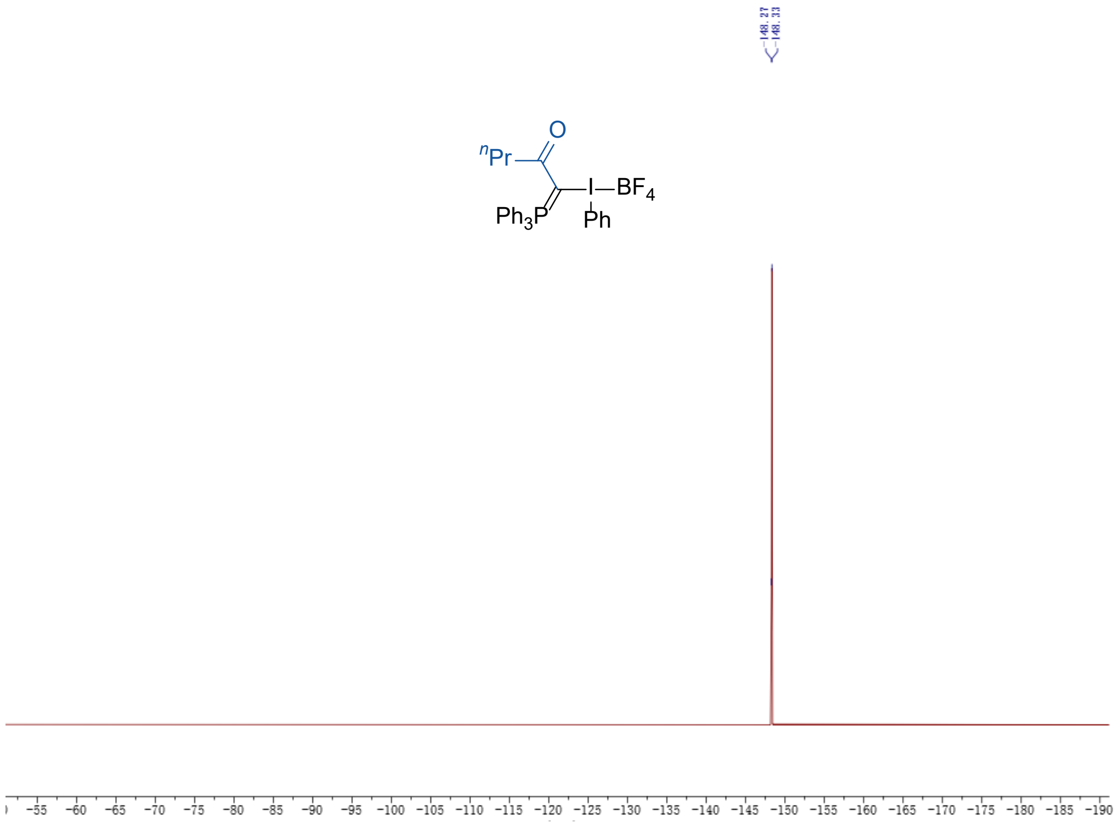

^{19}F NMR of the **1i** (376 MHz, 25 °C in DMSO-*d*$_6$)

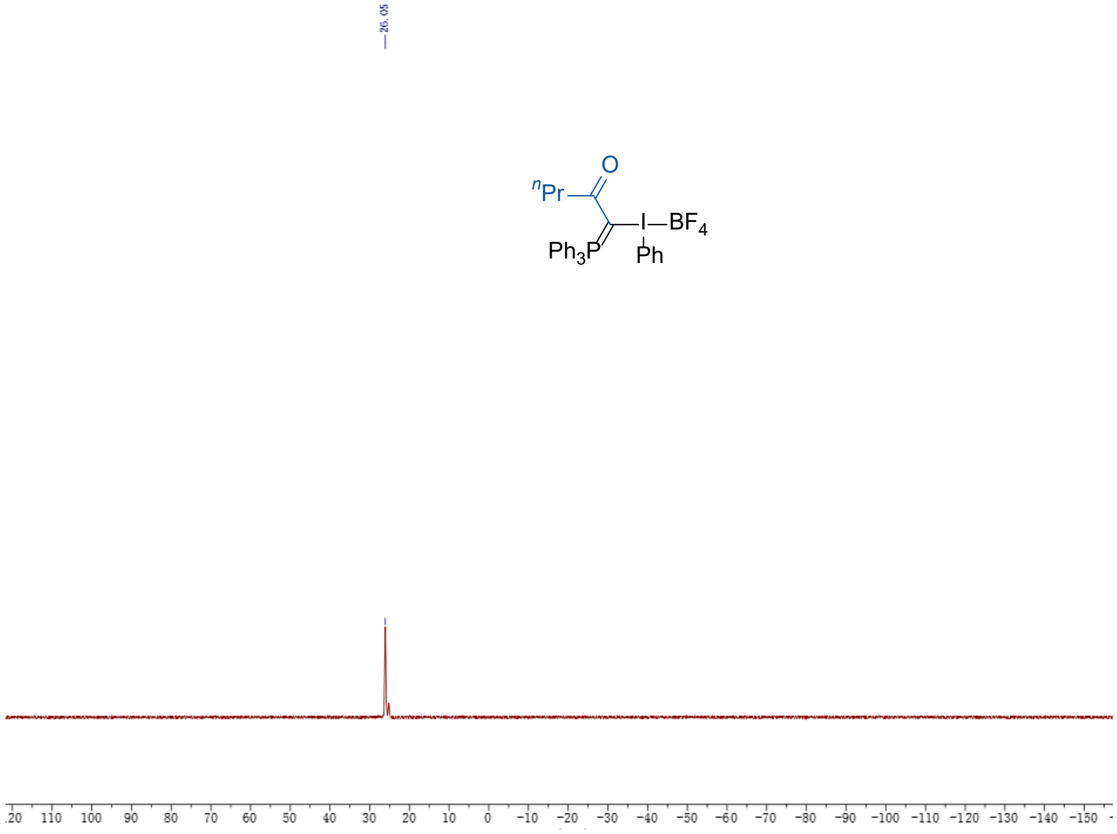

^{31}P NMR of the **1i** (162 MHz, 25 °C in DMSO-*d*$_6$)

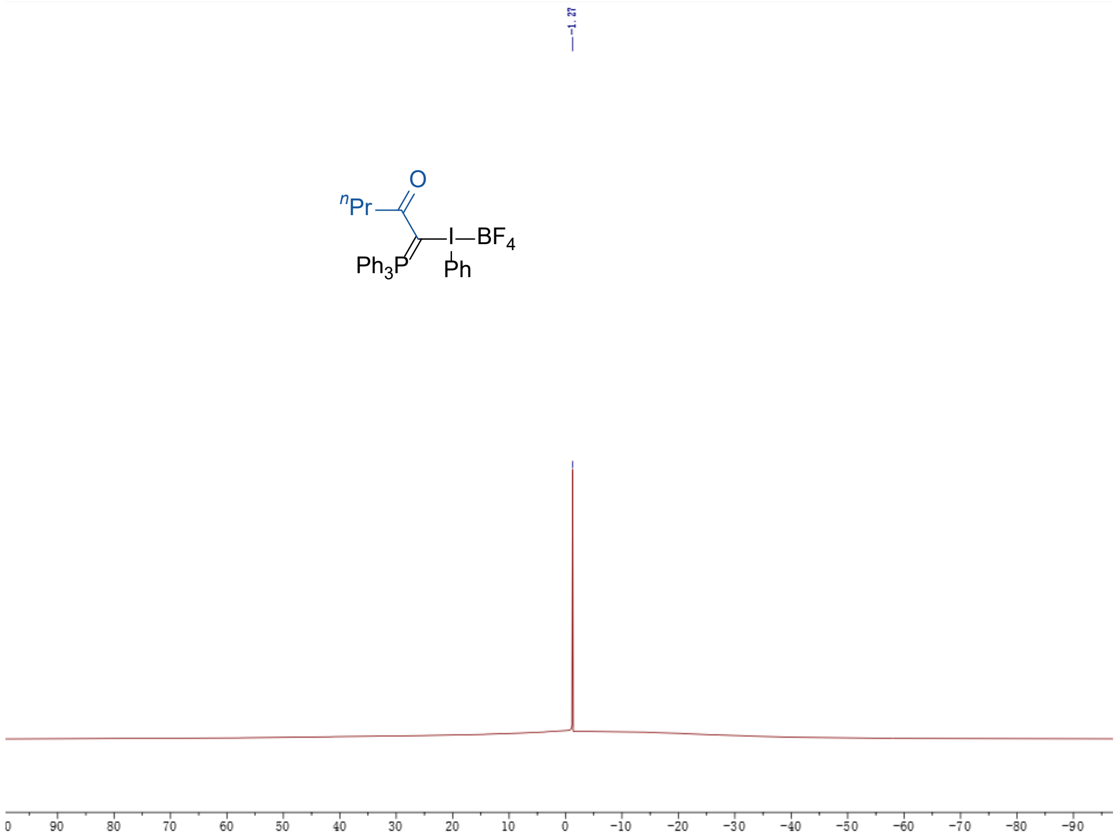

^{11}B NMR of the **1i** (128 MHz, 25 °C in DMSO-*d6*)

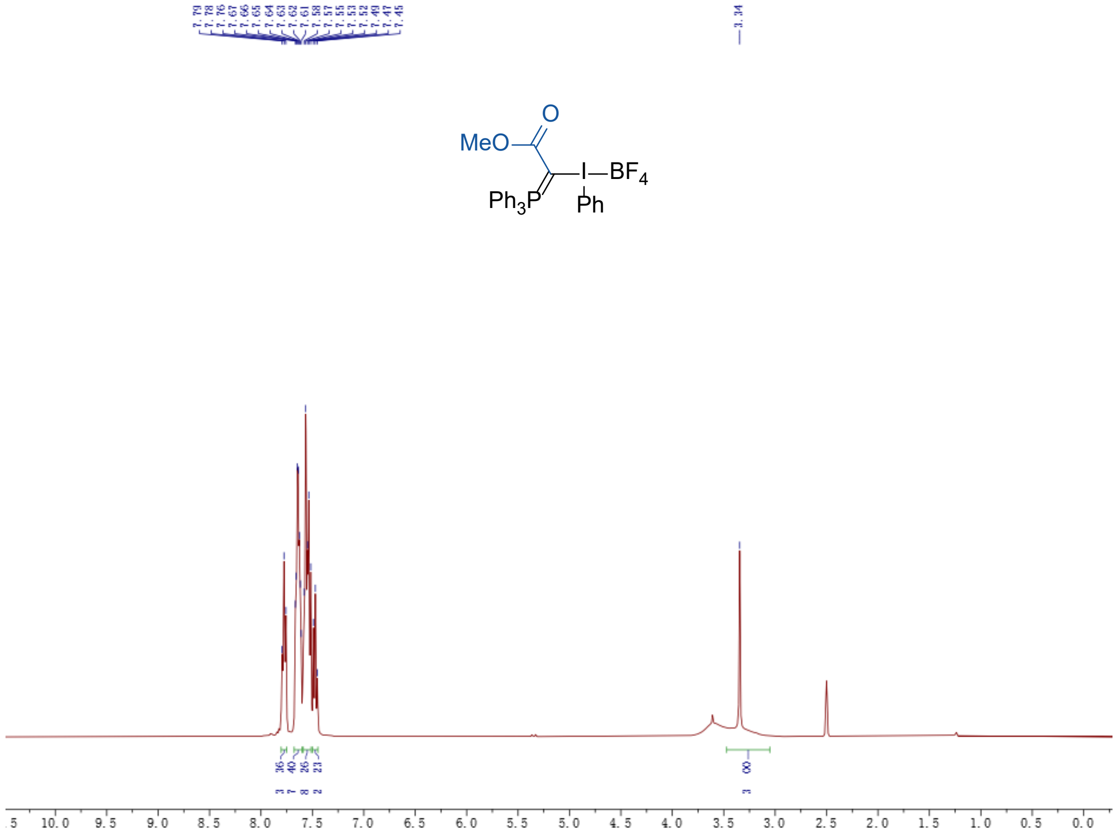

^{1}H NMR of the **1j** (400 MHz, 25 °C in DMSO-*d6*)

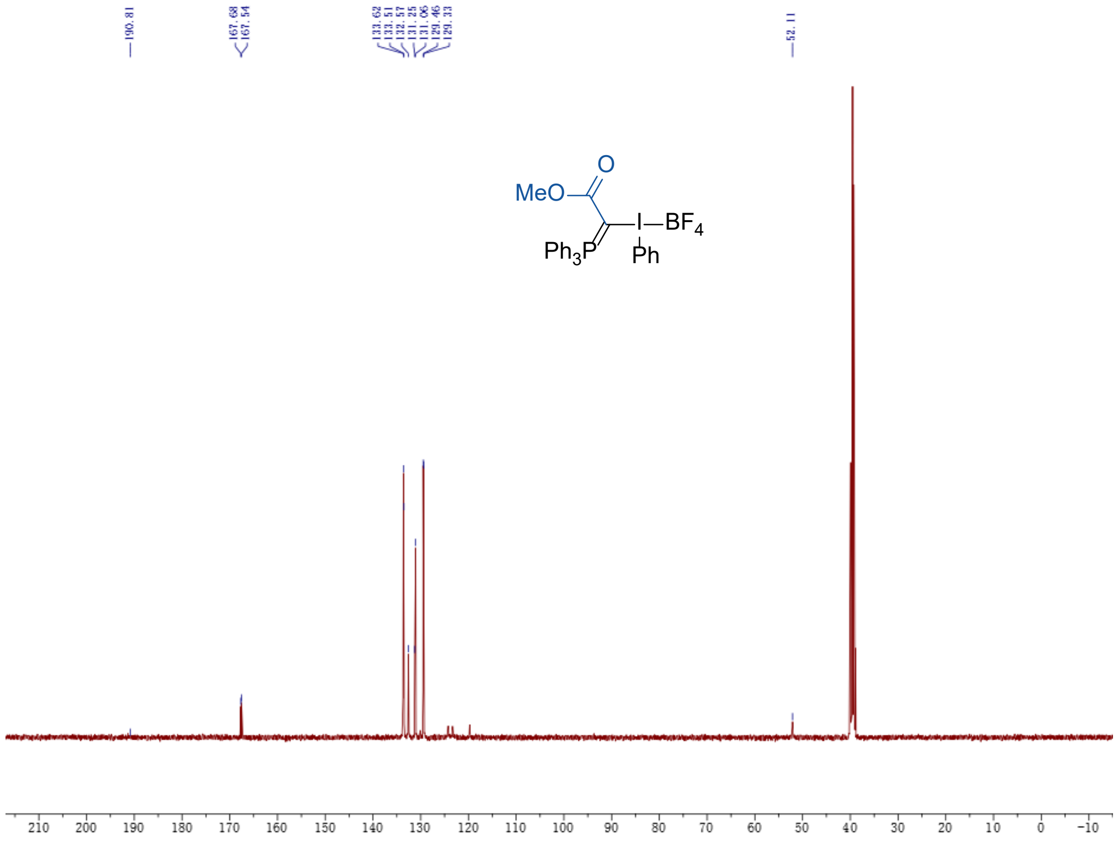

^{13}C NMR of the **1j** (101 MHz, 25 °C in DMSO-d_6)

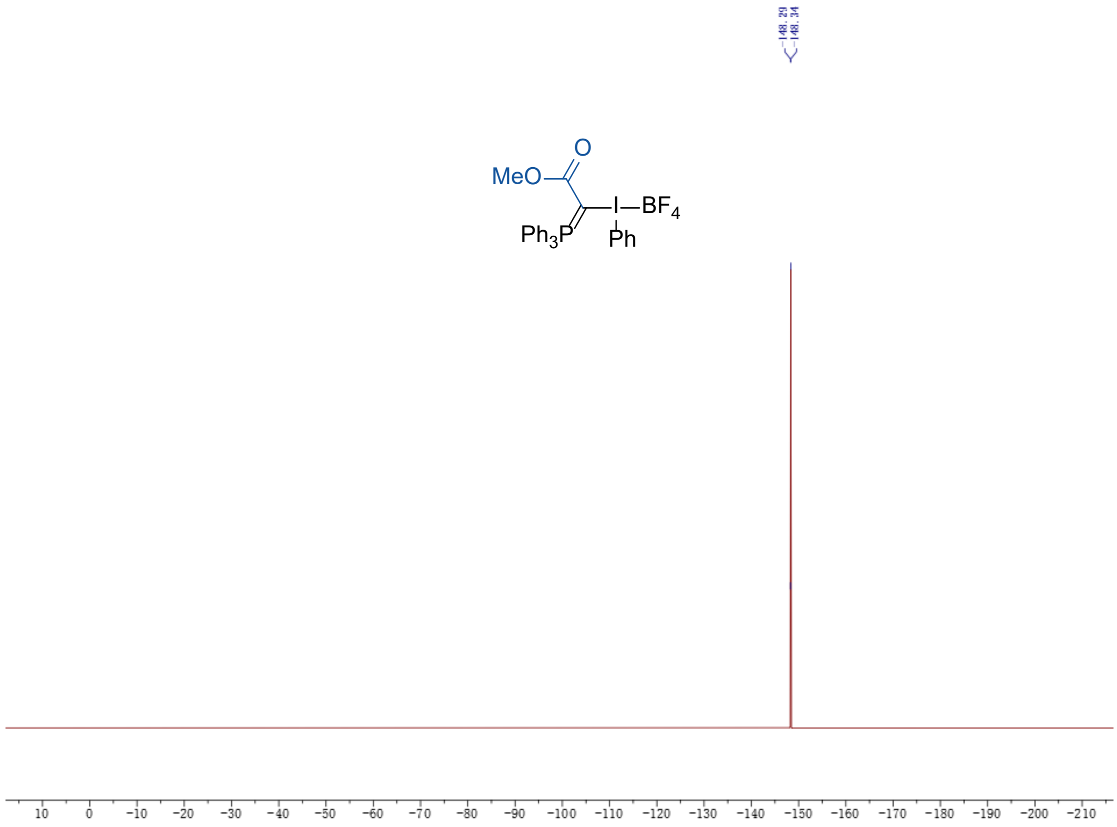

^{19}F NMR of the **1j** (376 MHz, 25 °C in DMSO-d_6)

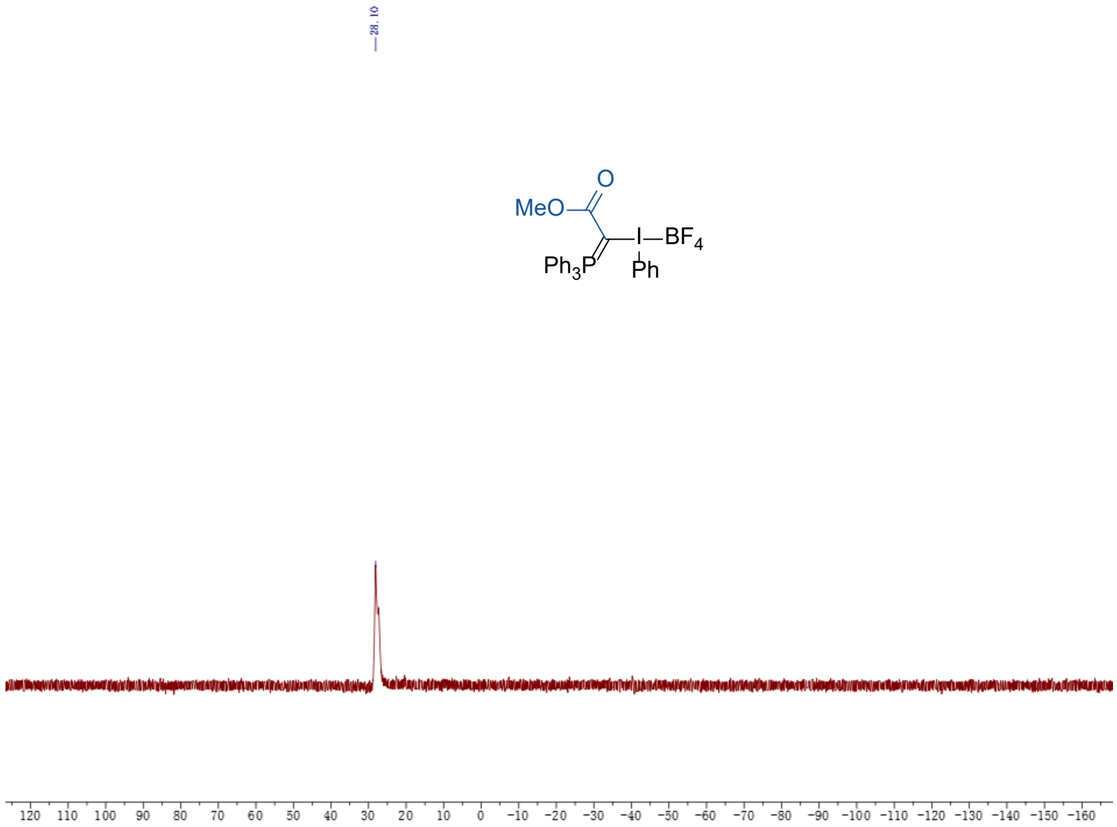

^{31}P NMR of the **1j** (162 MHz, 25 °C in DMSO-*d6*)

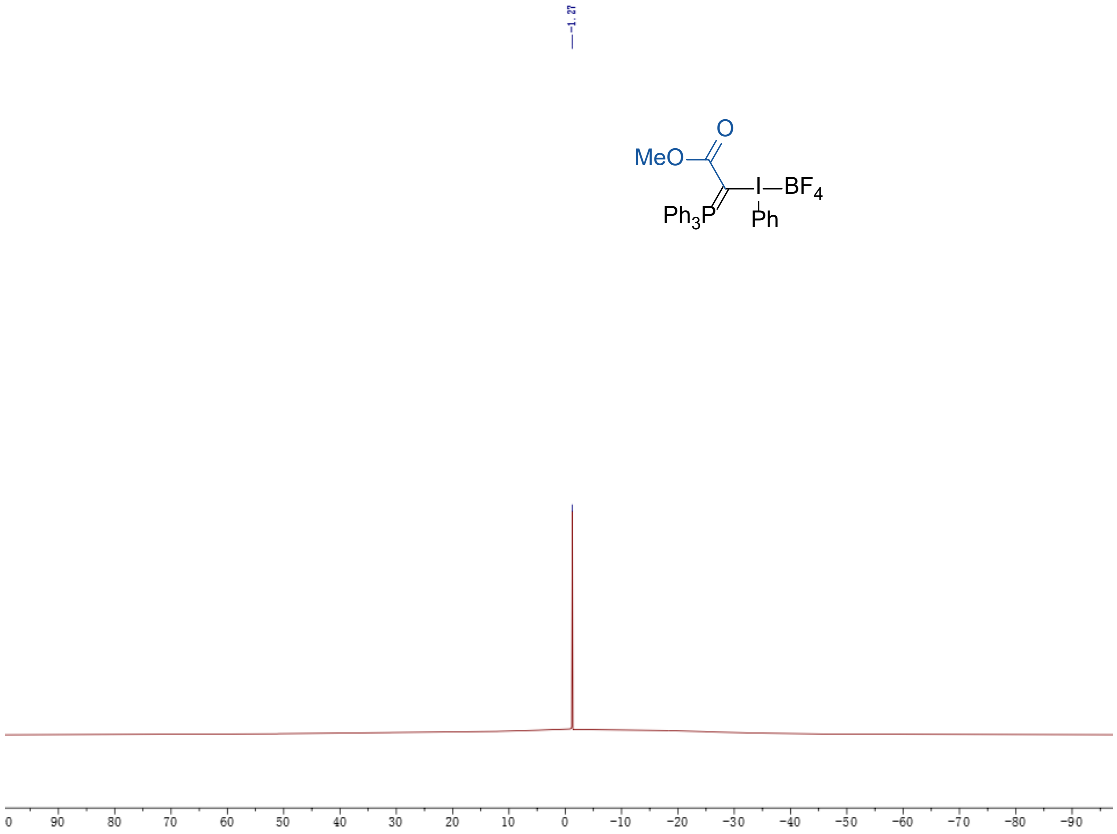

^{11}B NMR of the **1j** (128 MHz, 25 °C in DMSO-*d6*)

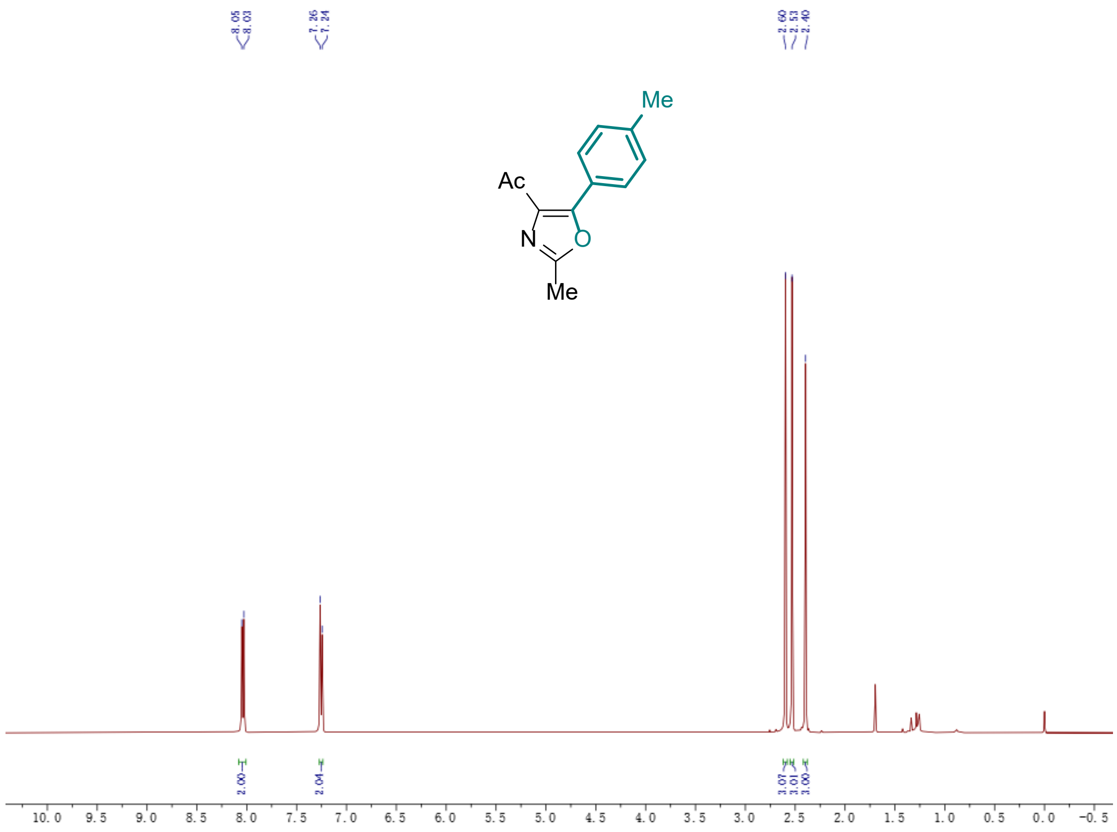

^{1}**H NMR** of the **3a** (400 MHz, 25 °C in $CDCl_3$)

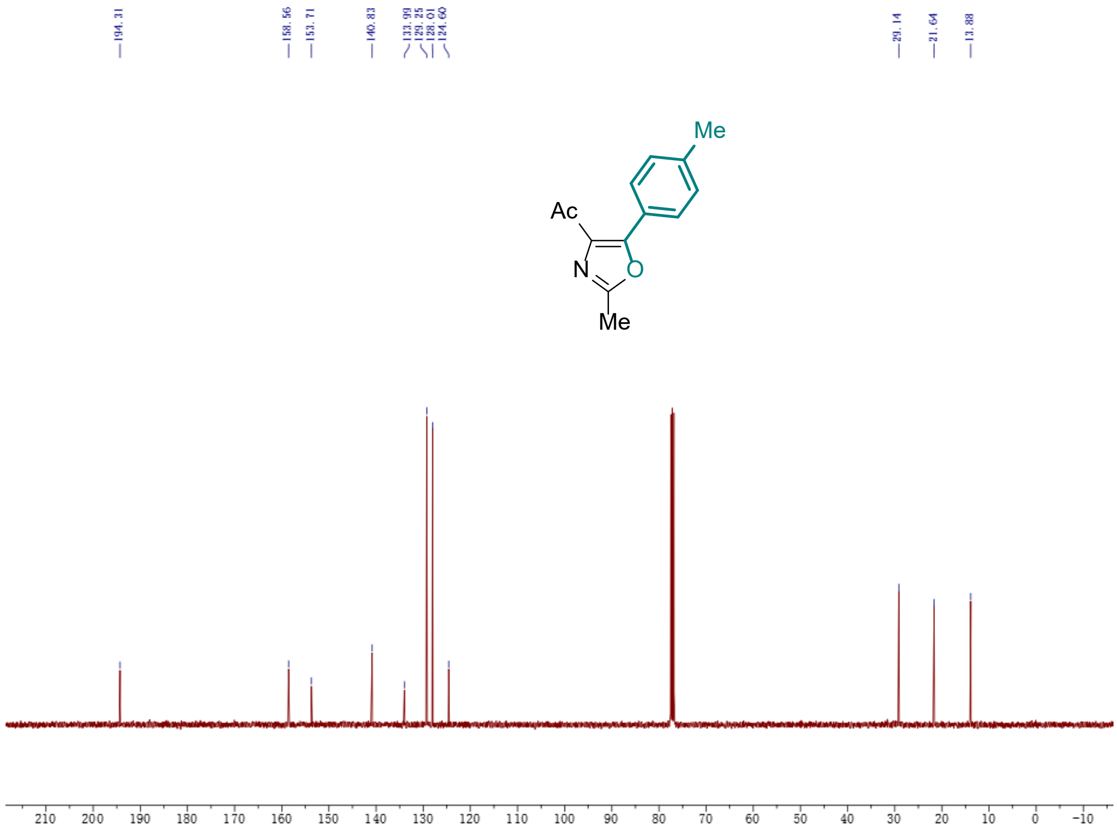

^{13}**C NMR** of the **3a** (101 MHz, 25 °C in $CDCl_3$)

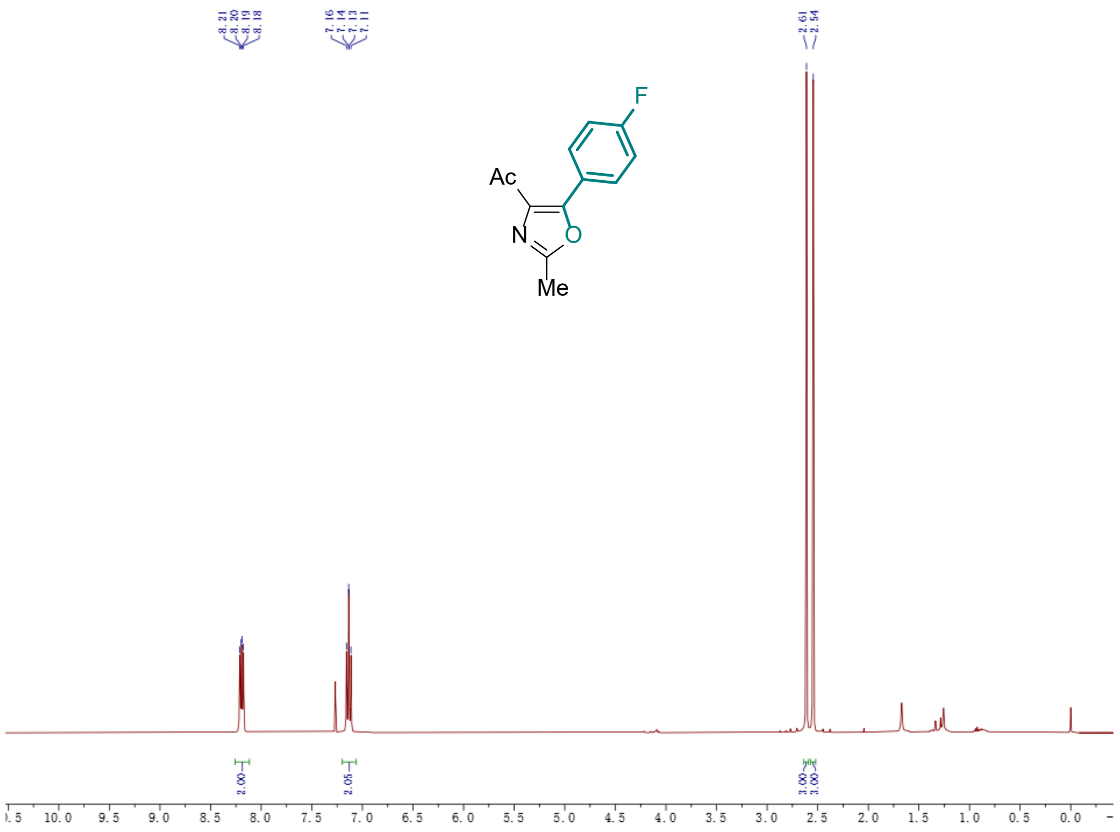

^{1}H NMR of the **3b** (400 MHz, 25 °C in $CDCl_3$)

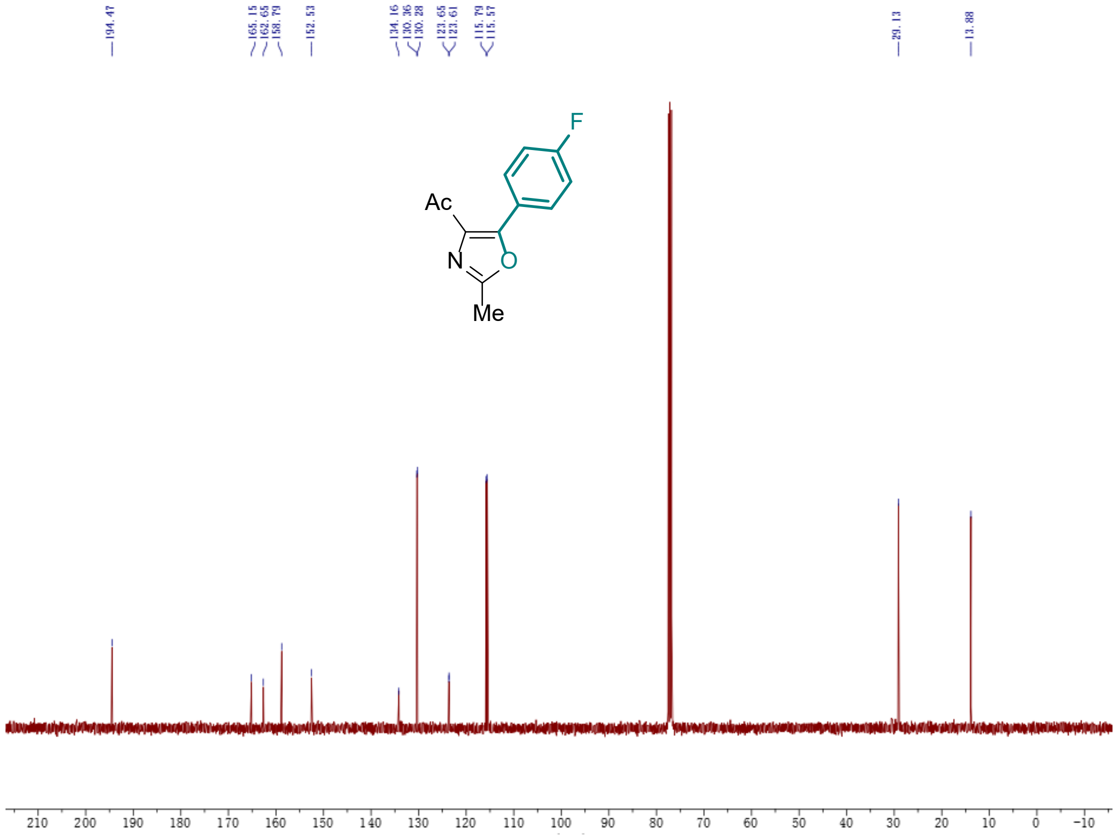

^{13}C NMR of the **3b** (101 MHz, 25 °C in $CDCl_3$)

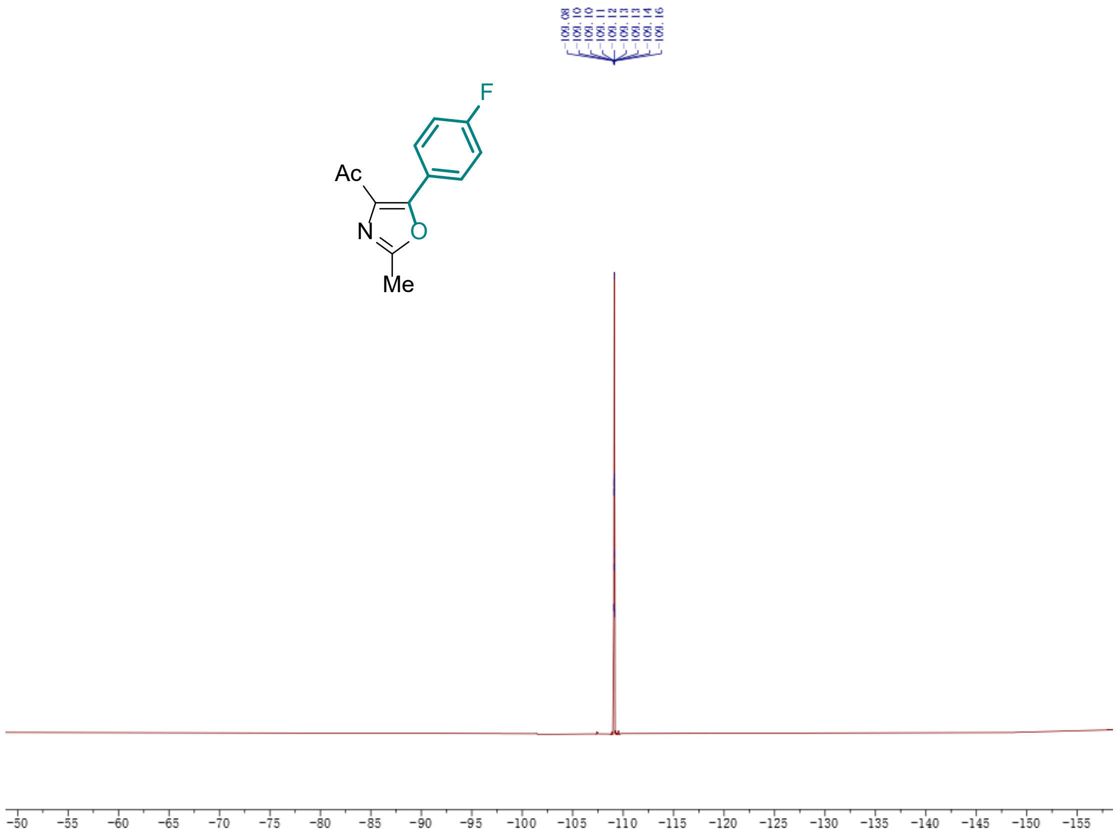

^{19}F NMR of the **3b** (376 MHz, 25 °C in $CDCl_3$)

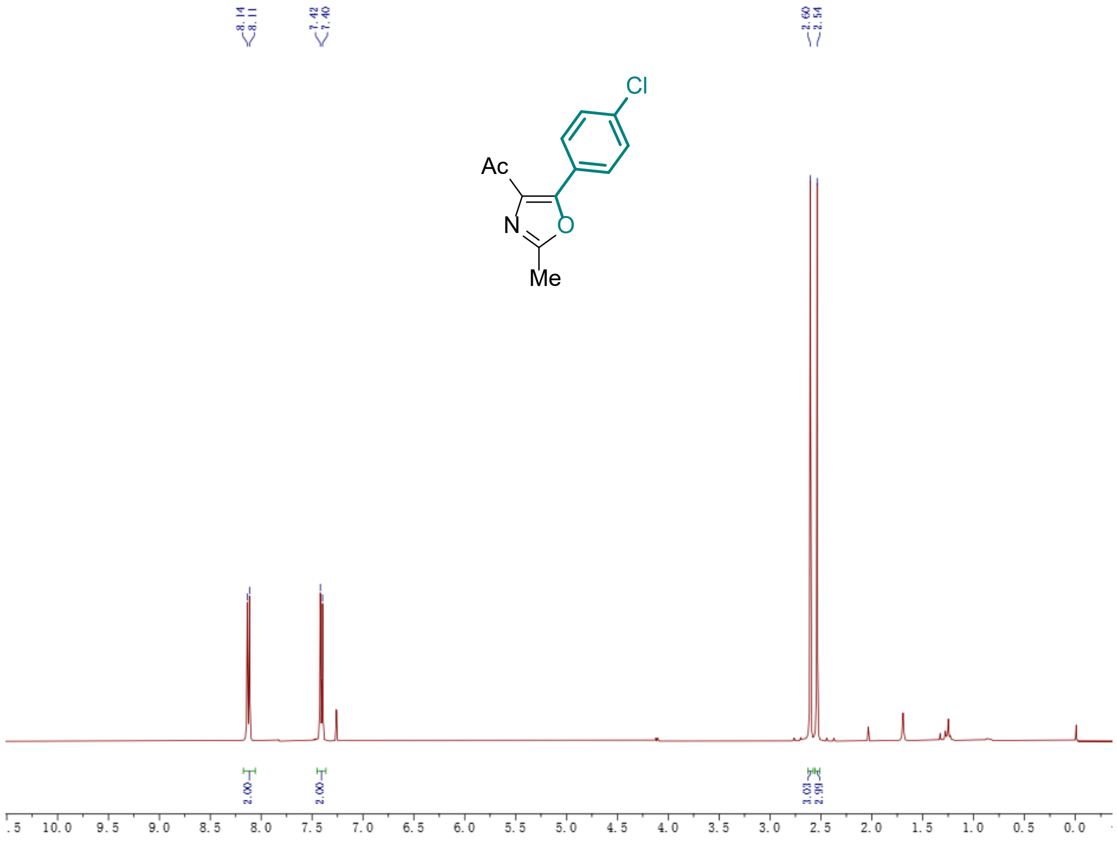

^{1}H NMR of the **3c** (400 MHz, 25 °C in $CDCl_3$)

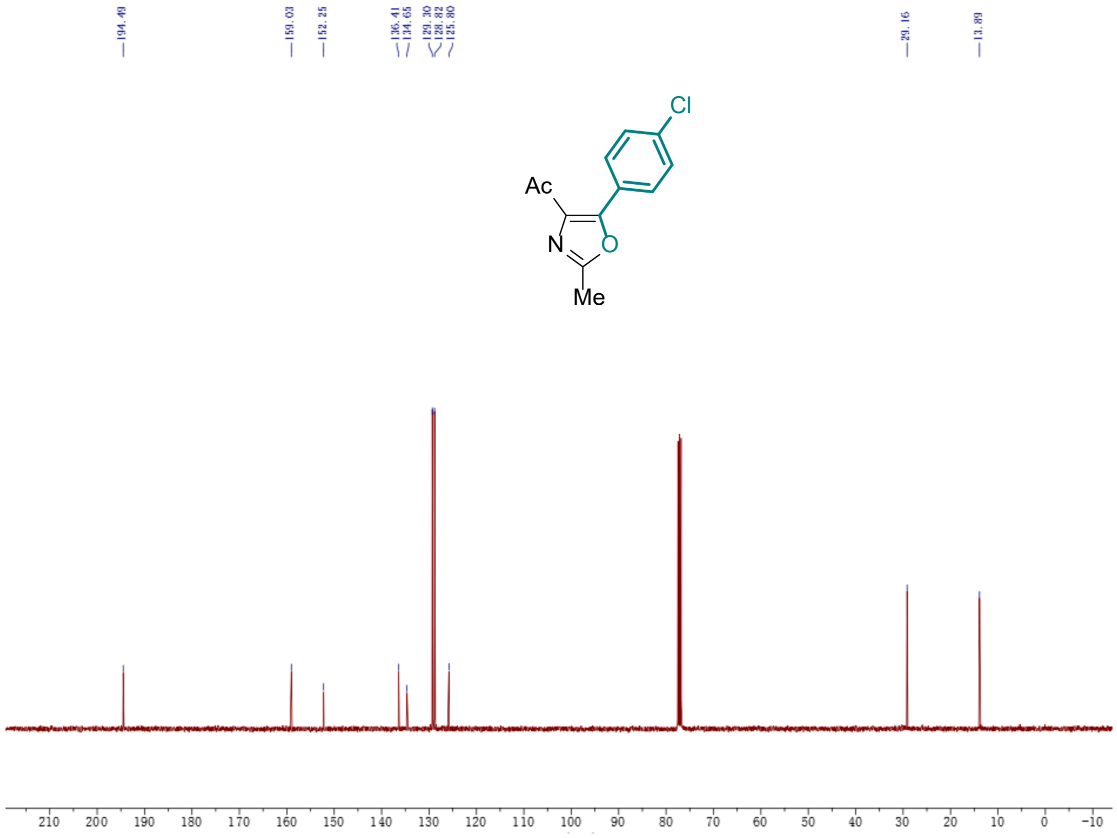

^{13}C NMR of the **3c** (101 MHz, 25 °C in $CDCl_3$)

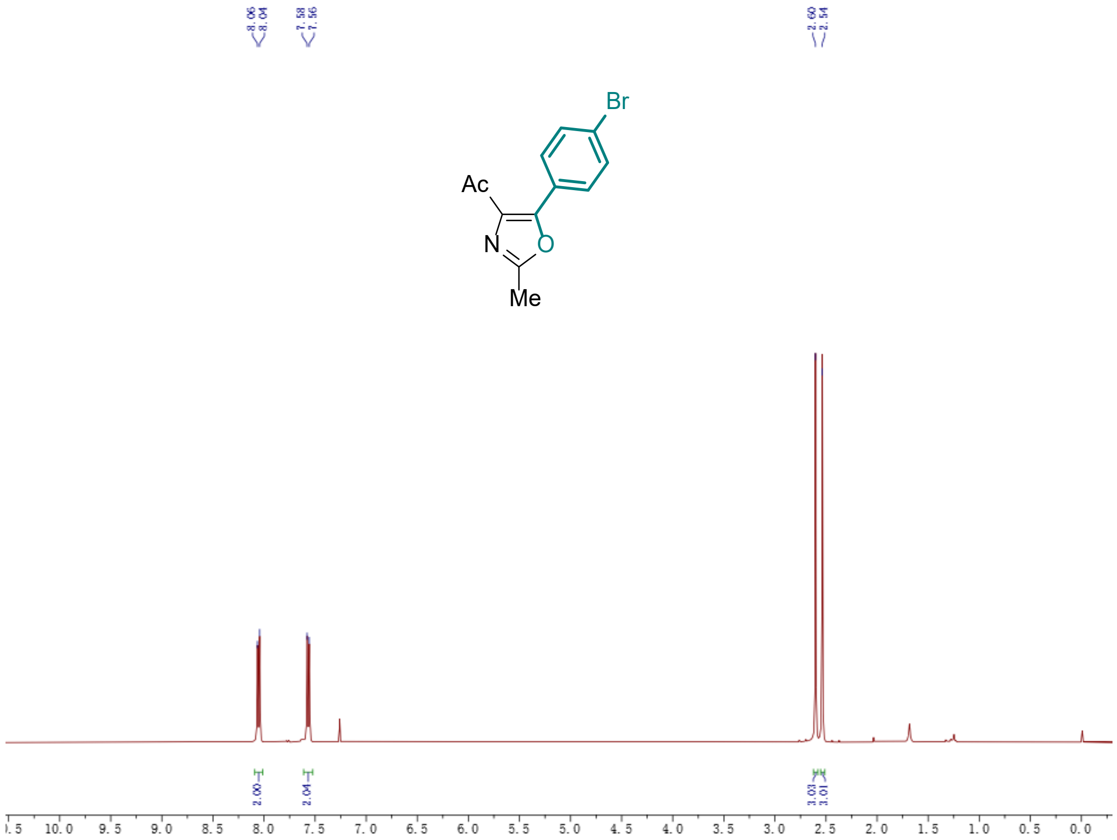

^{1}H NMR of the **3d** (400 MHz, 25 °C in $CDCl_3$)

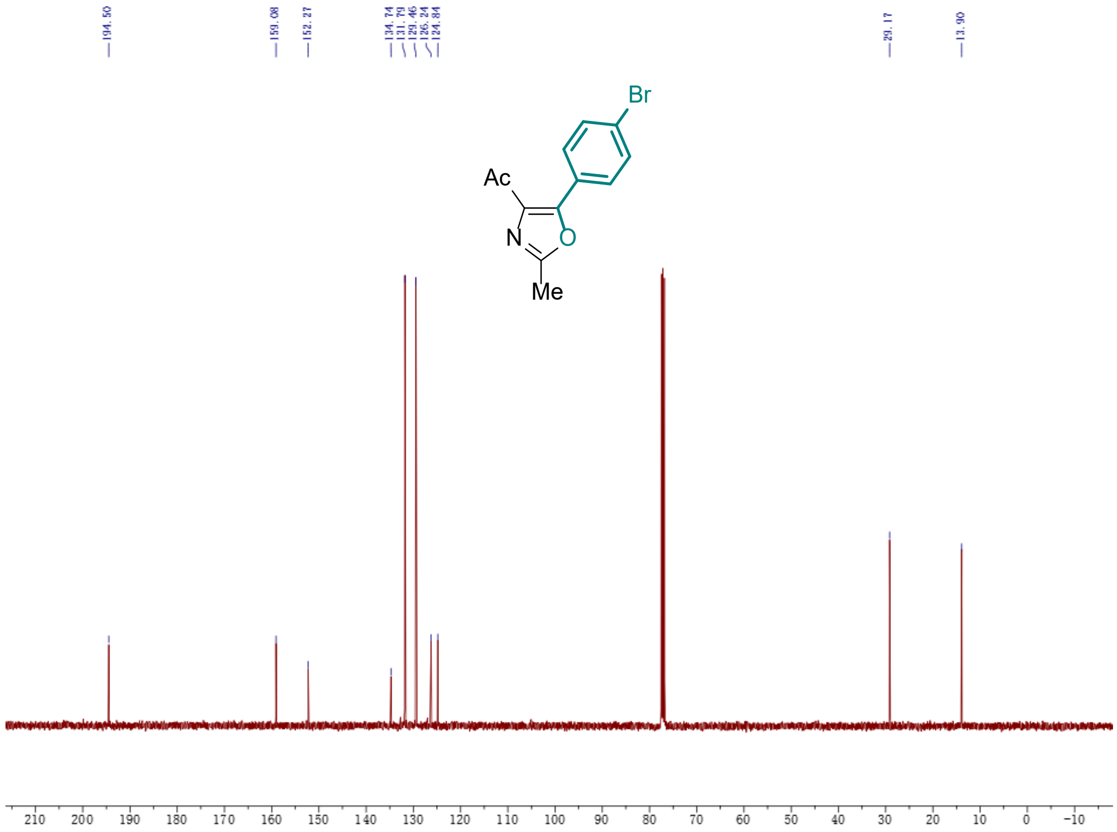

^{13}C **NMR** of the **3d** (101 MHz, 25 °C in $CDCl_3$)

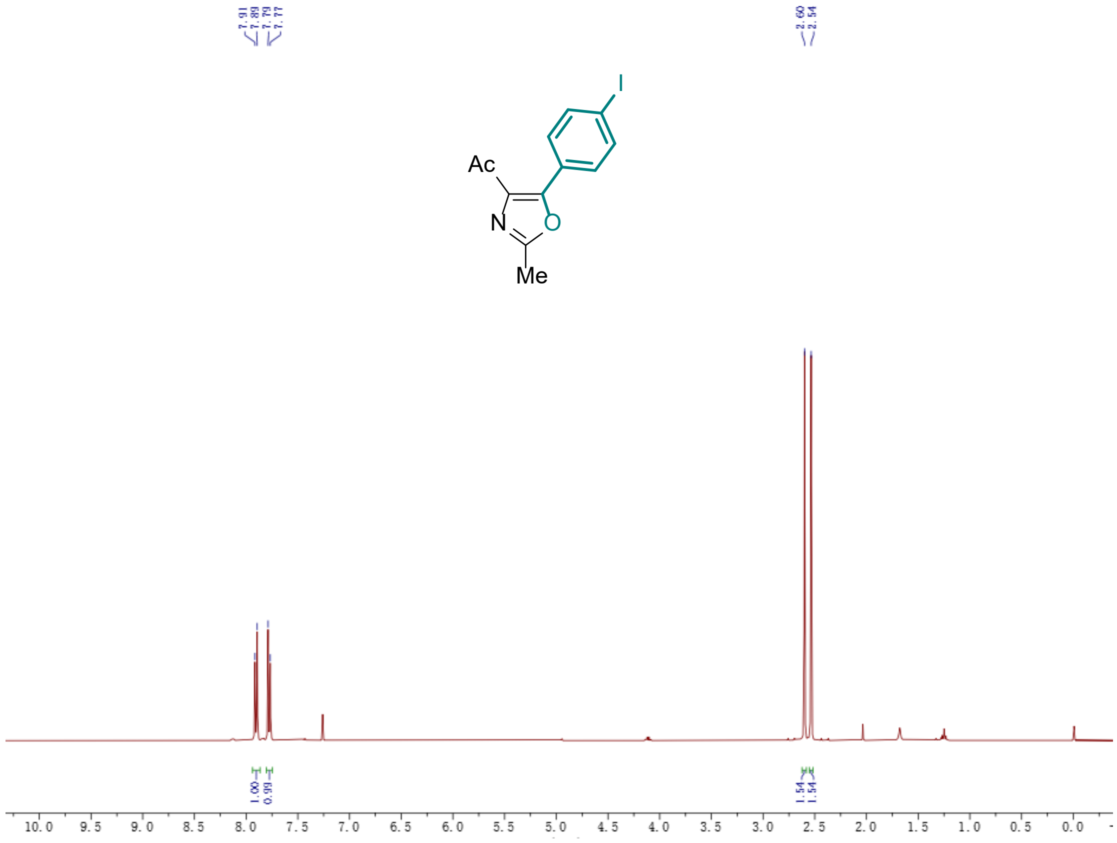

^{1}H **NMR** of the **3e** (400 MHz, 25 °C in $CDCl_3$)

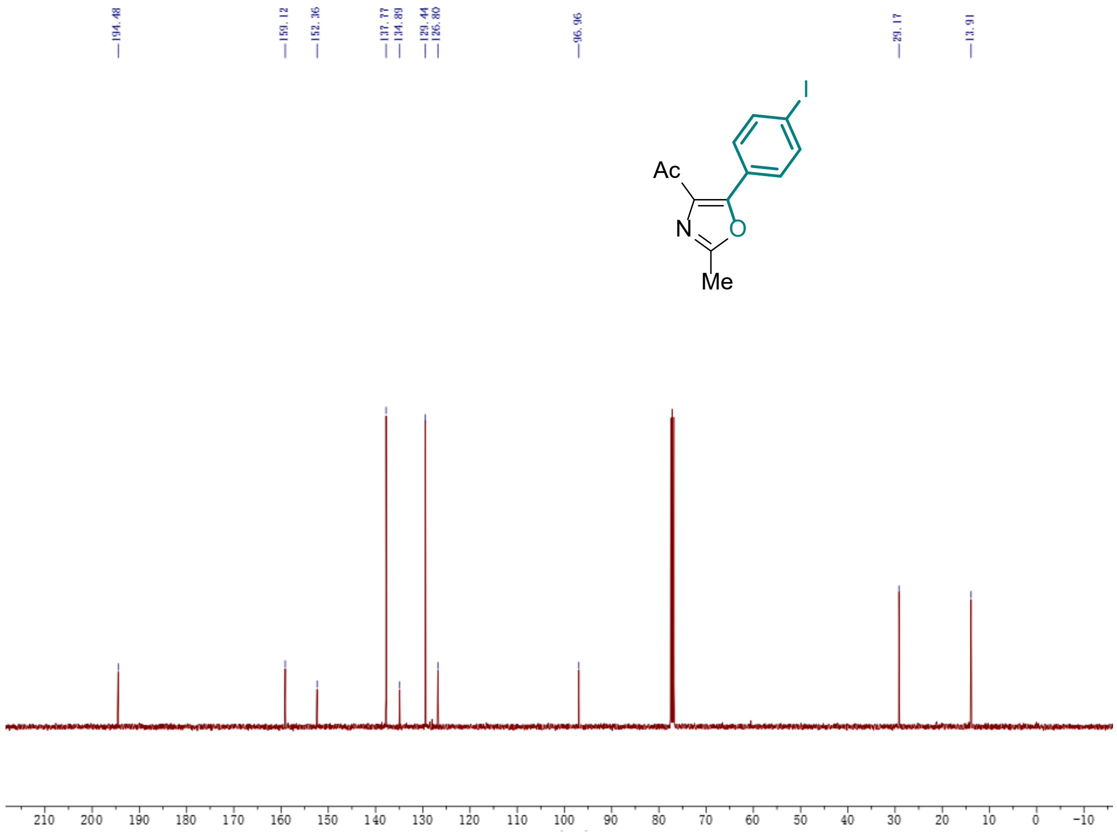

^{13}C NMR of the **3e** (101 MHz, 25 °C in $CDCl_3$)

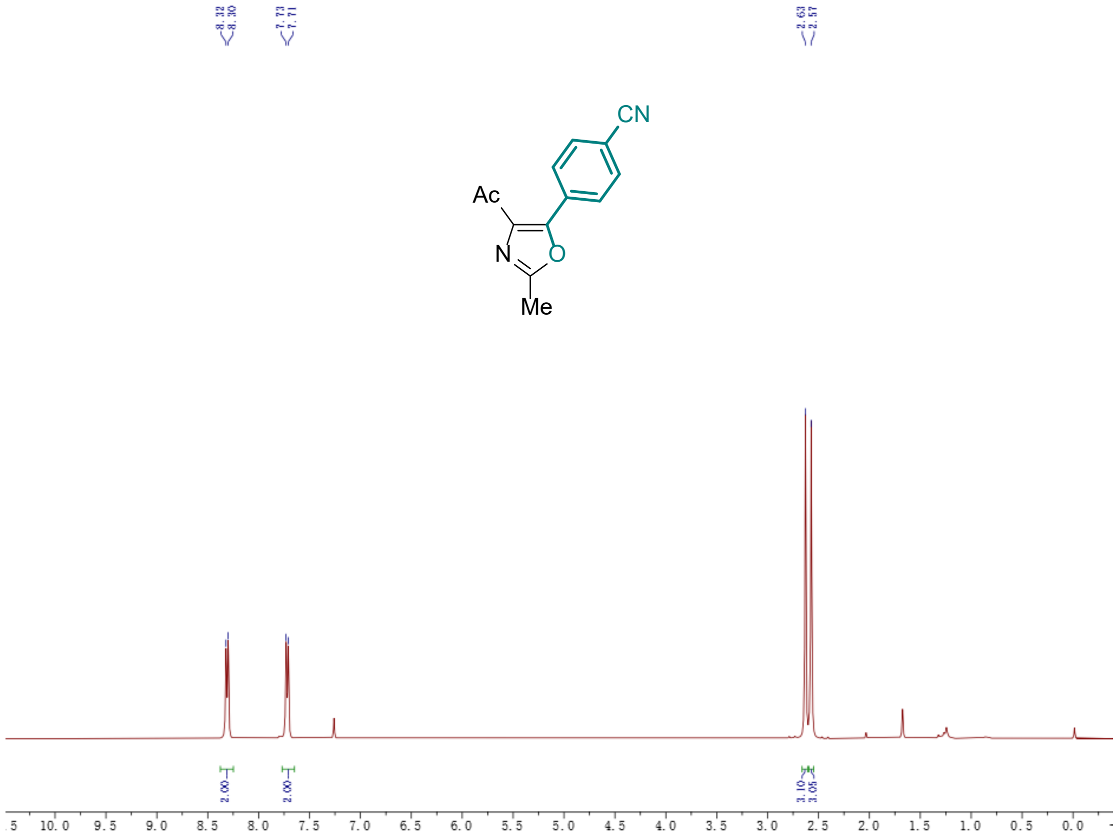

^{1}H NMR of the **3f** (400 MHz, 25 °C in $CDCl_3$)

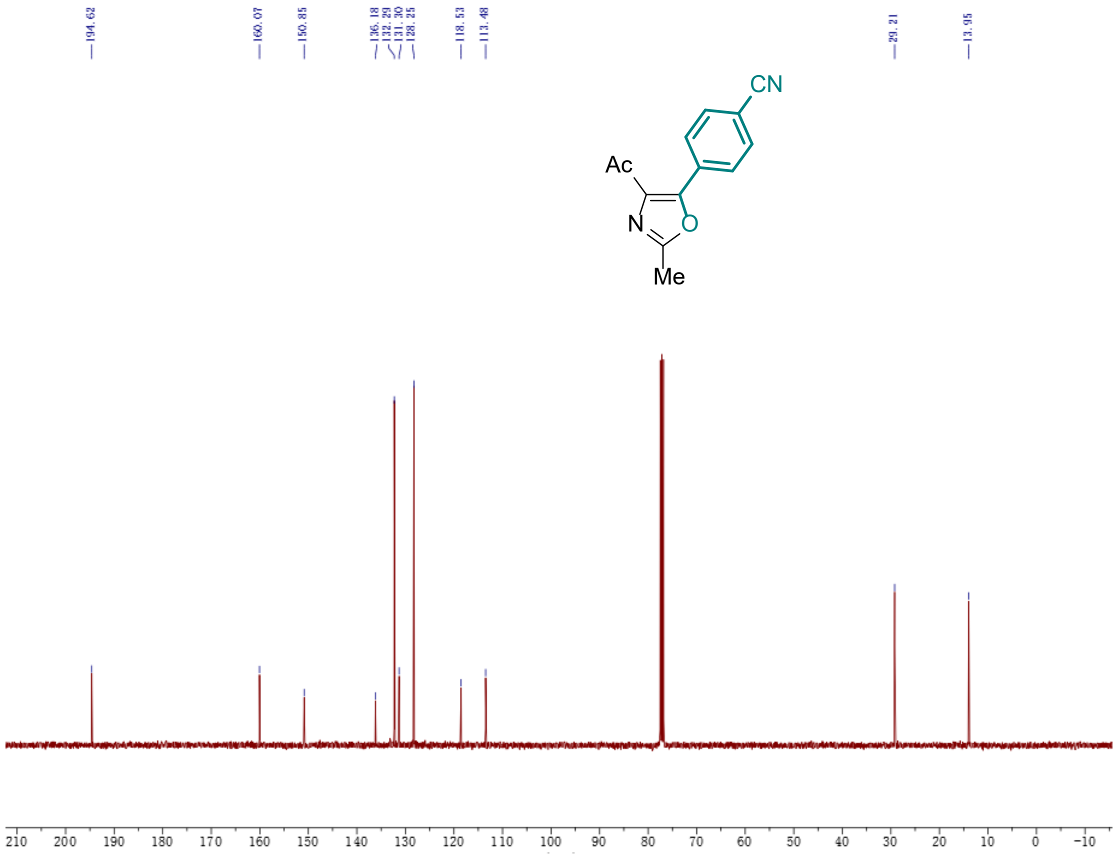

^{13}C **NMR** of the **3f** (101 MHz, 25 °C in $CDCl_3$)

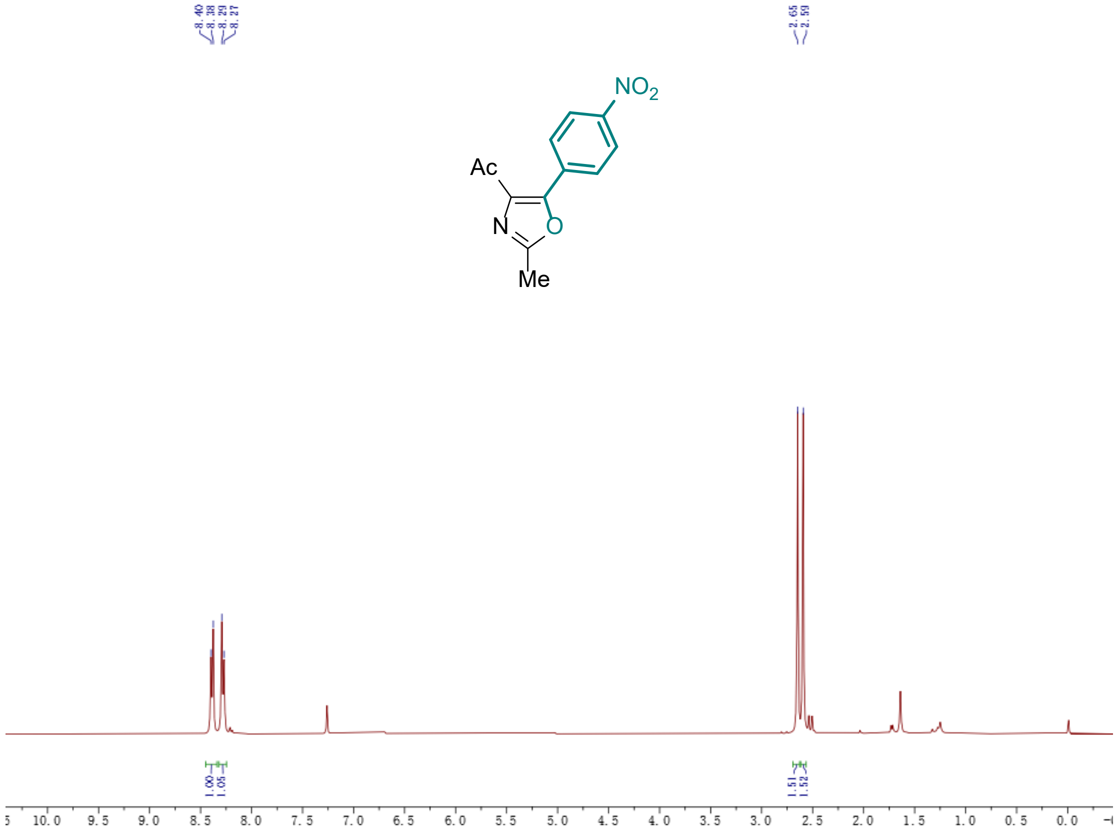

^{1}H **NMR** of the **3g** (400 MHz, 25 °C in $CDCl_3$)

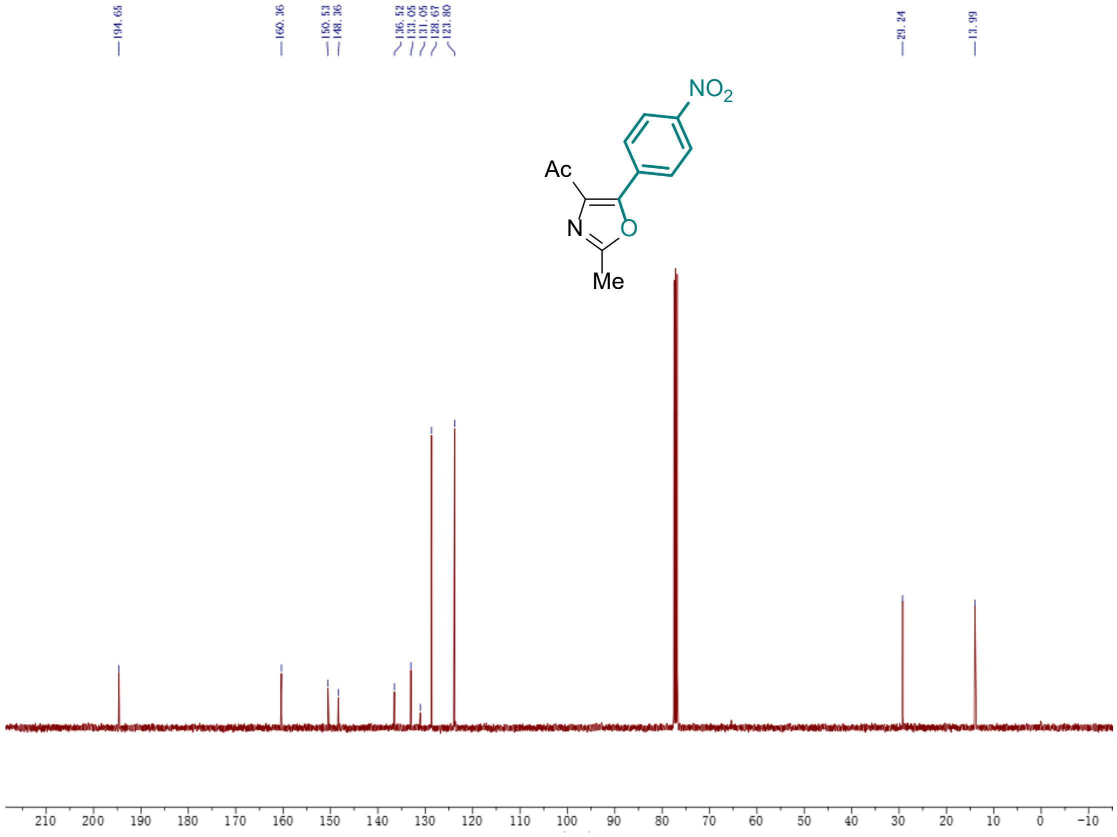

^{13}C NMR of the **3g** (101 MHz, 25 °C in $CDCl_3$)

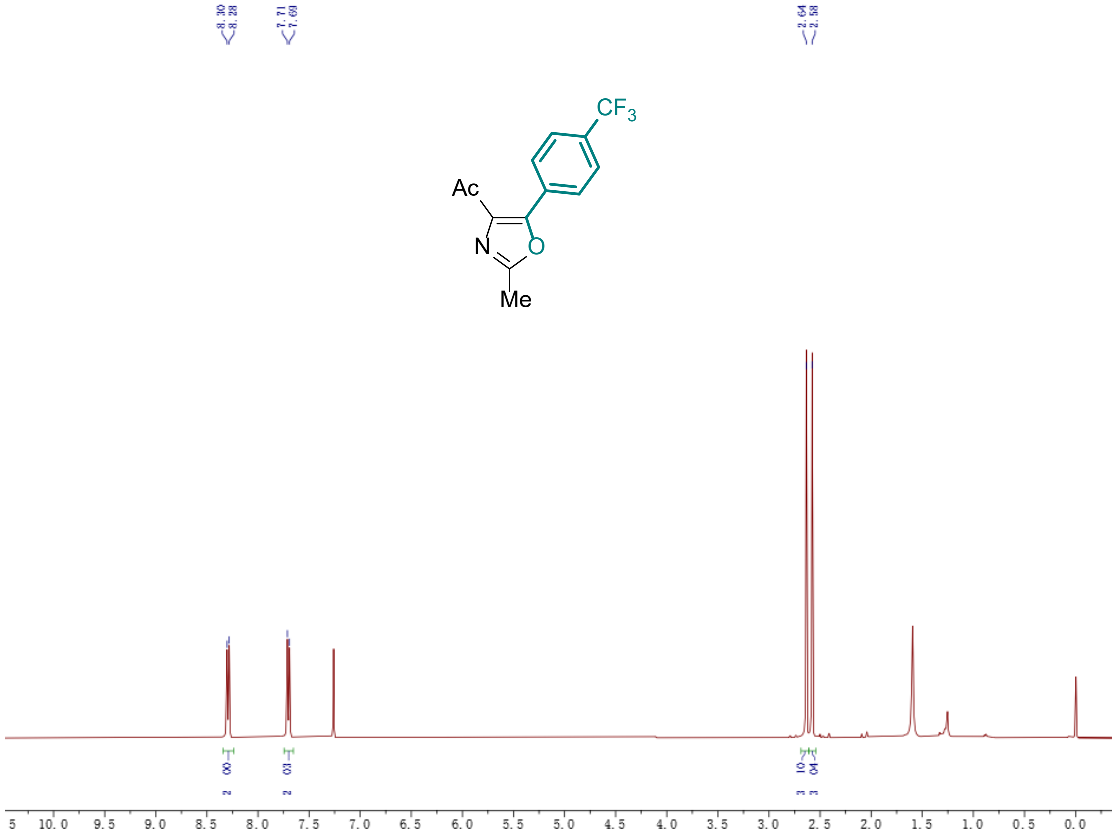

^{1}H NMR of the **3h** (400 MHz, 25 °C in $CDCl_3$)

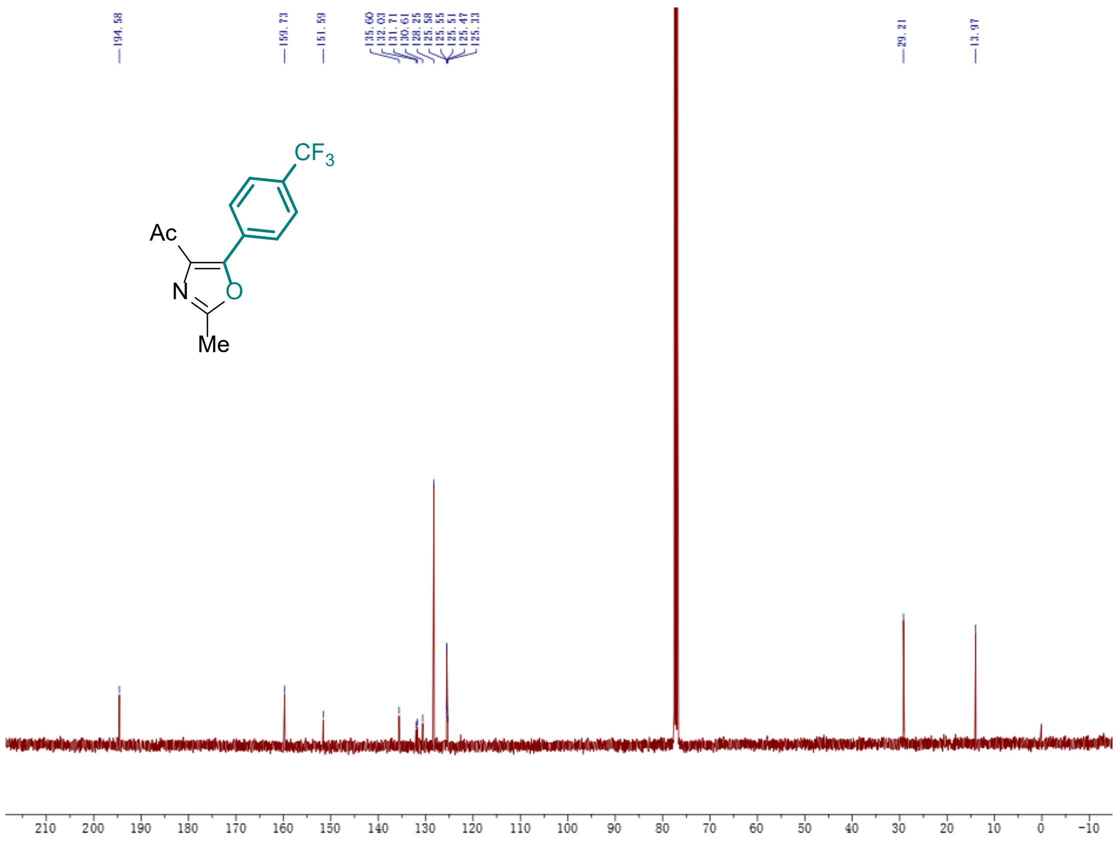

^{13}C NMR of the **3h** (101 MHz, 25 °C in $CDCl_3$)

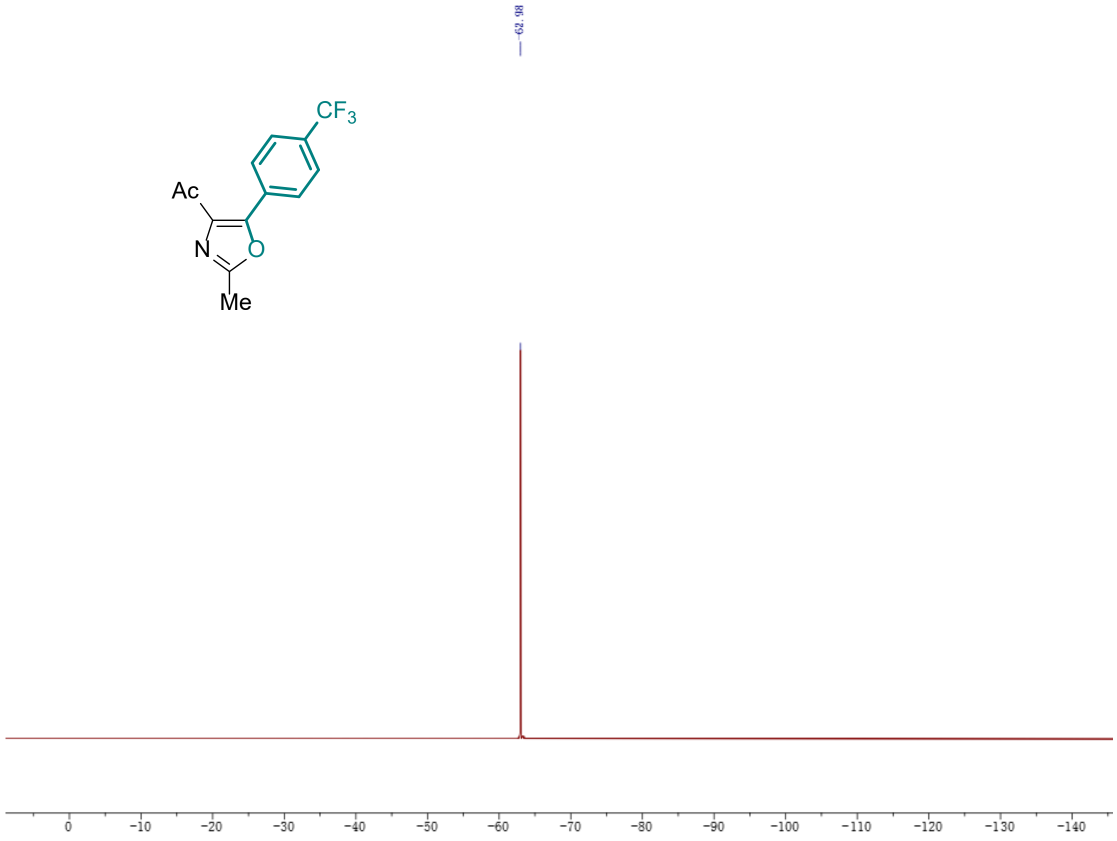

^{19}F NMR of the **3h** (376 MHz, 25 °C in $CDCl_3$)

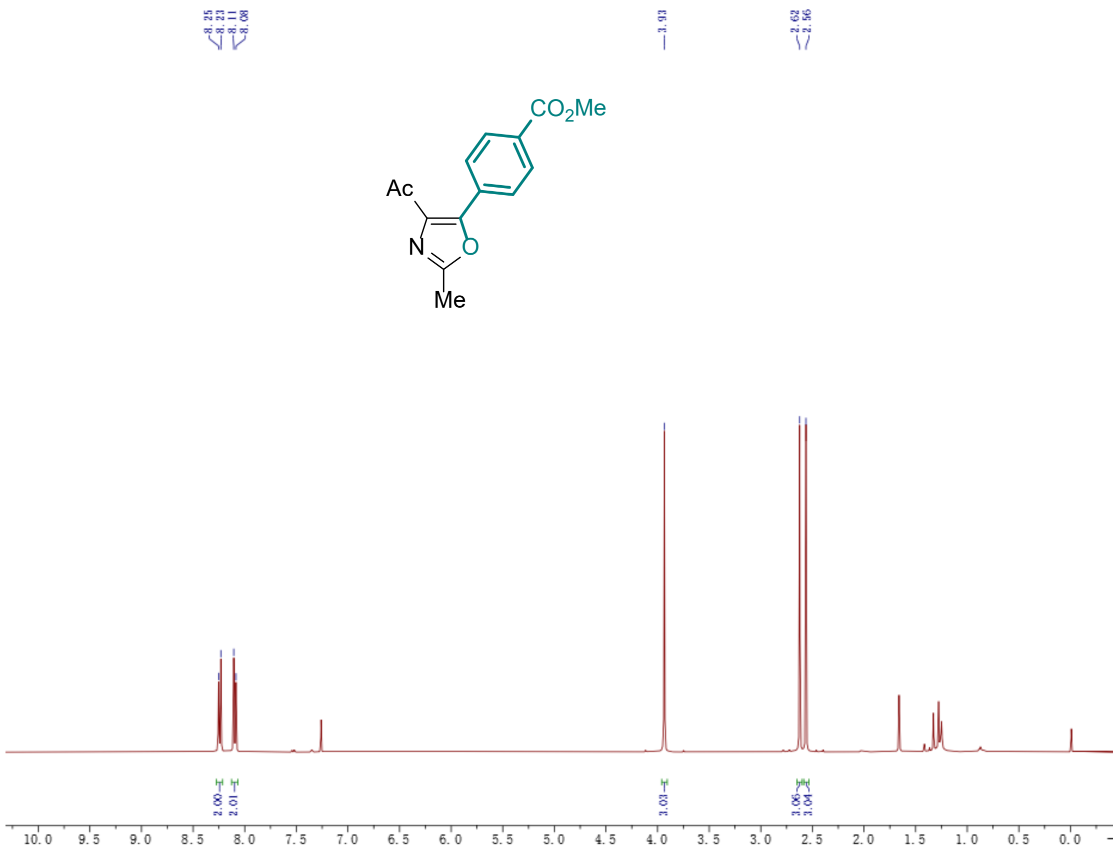

^{1}H NMR of the **3i** (400 MHz, 25 °C in $CDCl_3$)

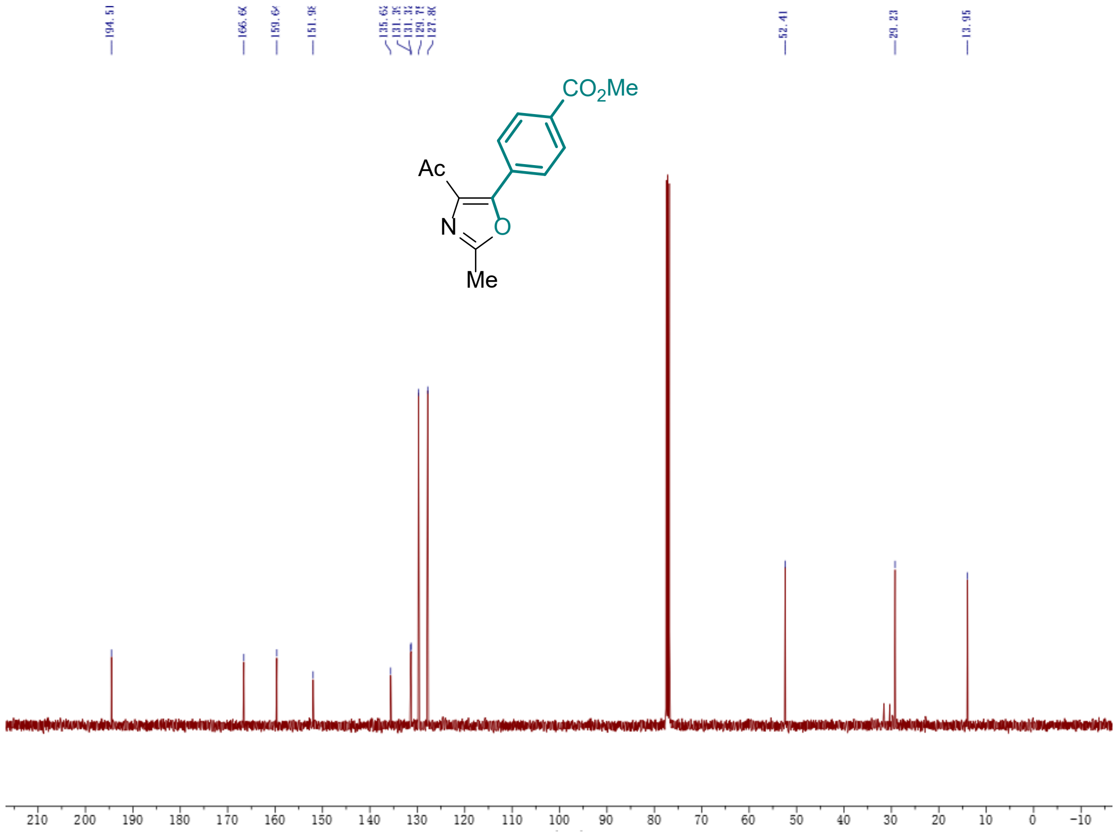

^{13}C NMR of the **3i** (101 MHz, 25 °C in $CDCl_3$)

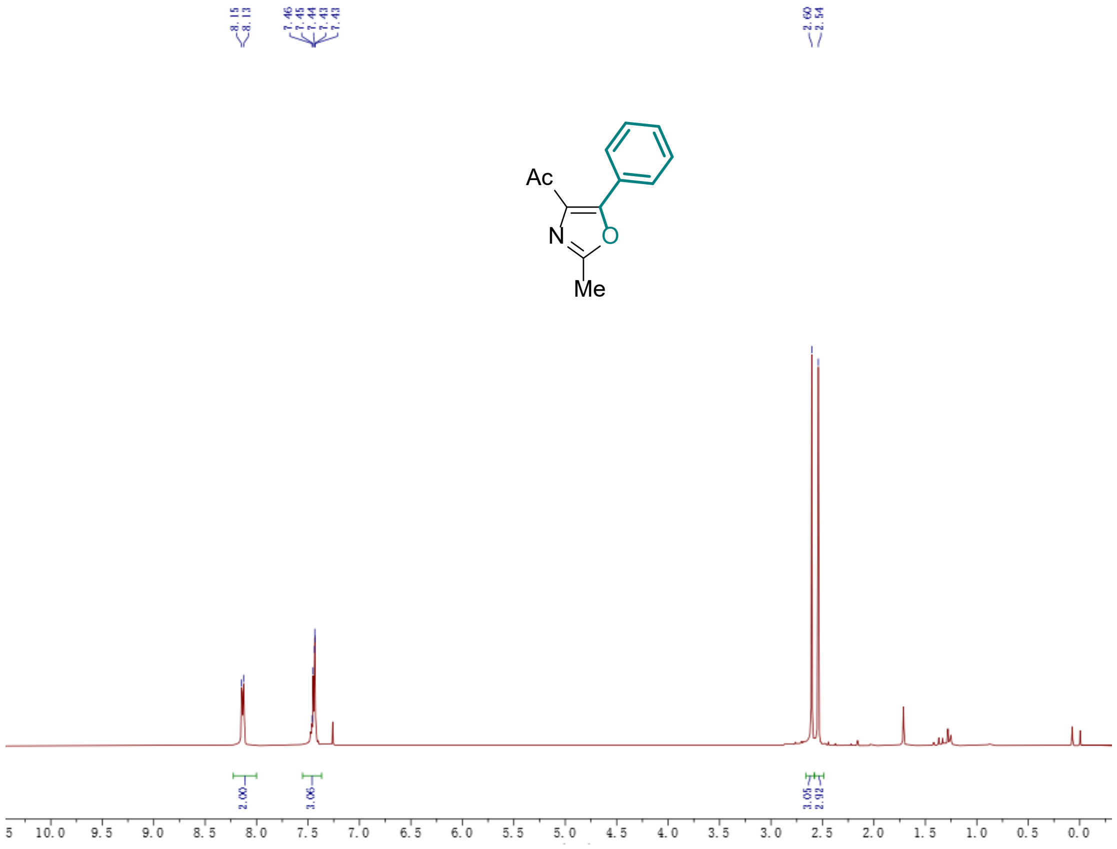

^{1}H NMR of the **3j** (400 MHz, 25 °C in $CDCl_3$)

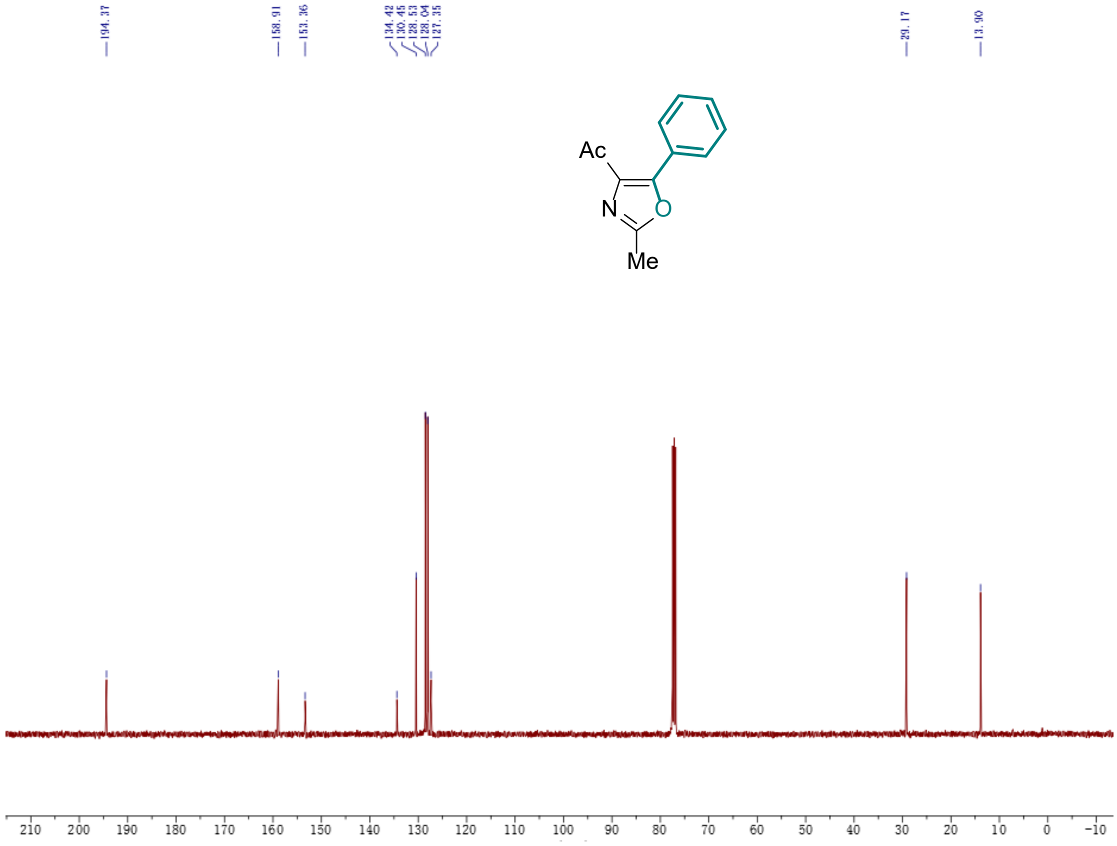

^{13}C NMR of the **3j** (101 MHz, 25 °C in $CDCl_3$)

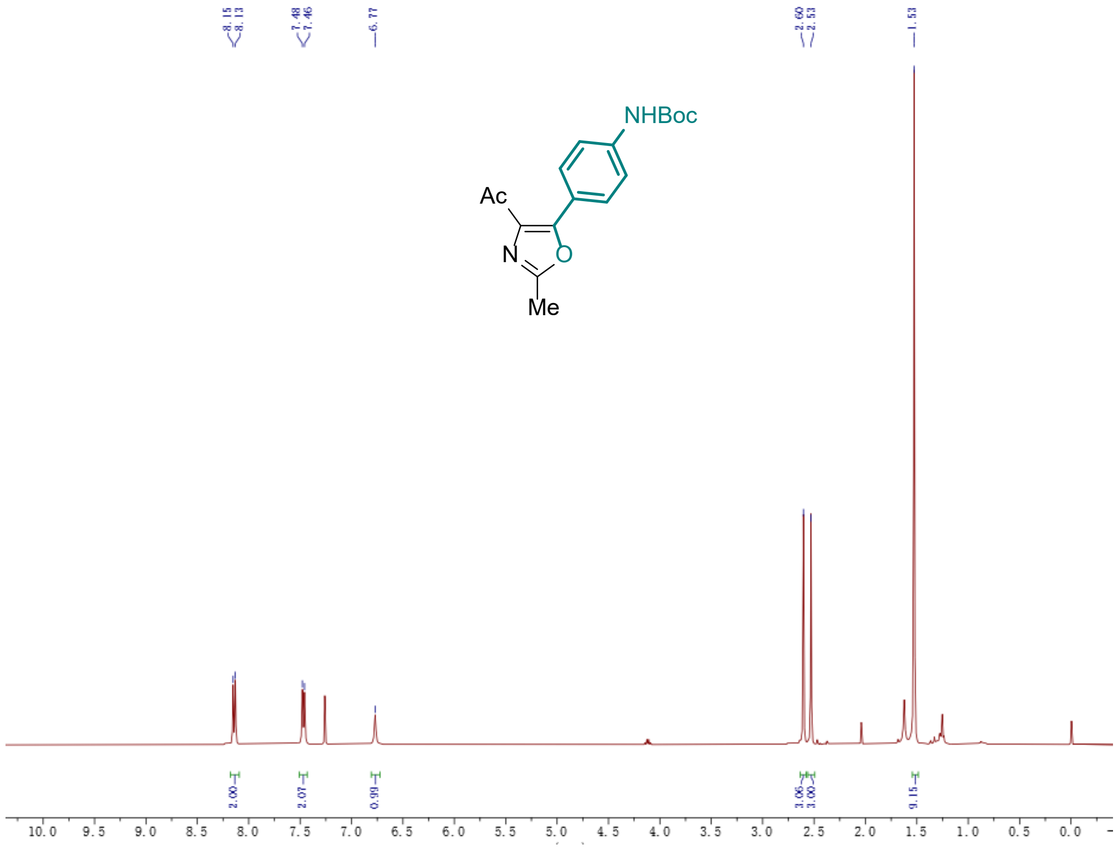

^{1}H NMR of the **3k** (400 MHz, 25 °C in $CDCl_3$)

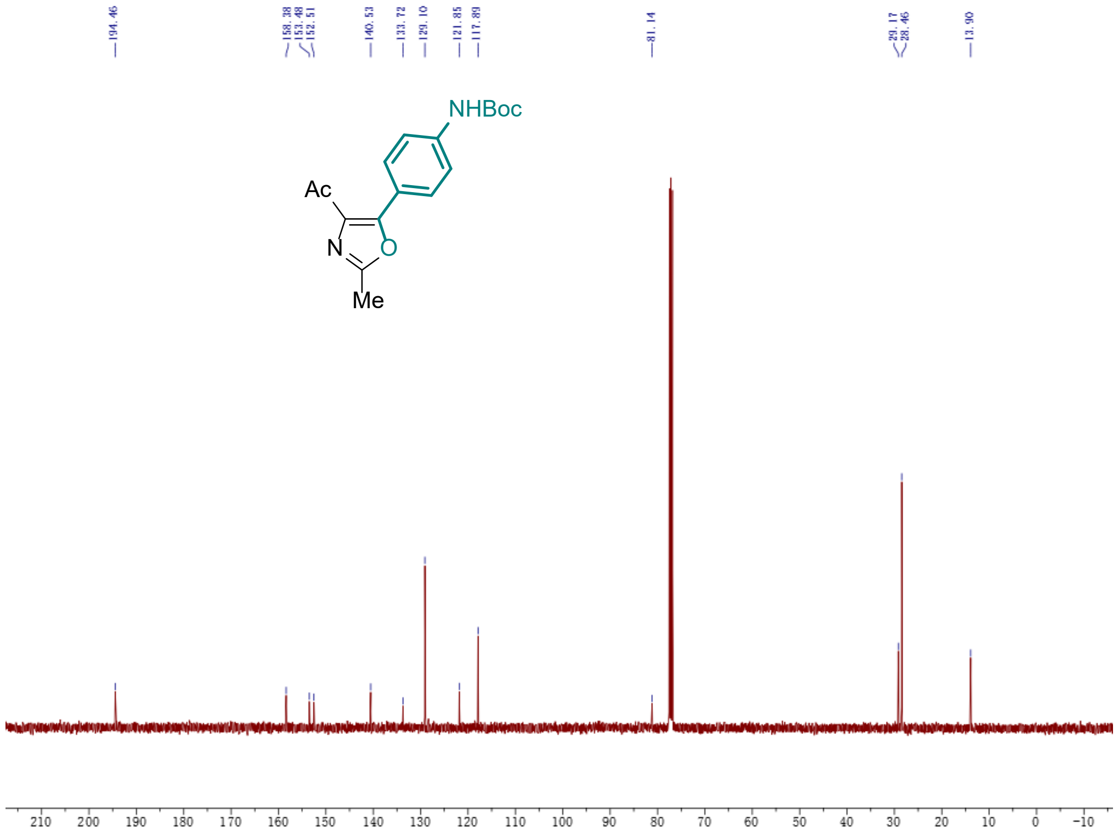

^{13}C NMR of the **3k** (101 MHz, 25 °C in $CDCl_3$)

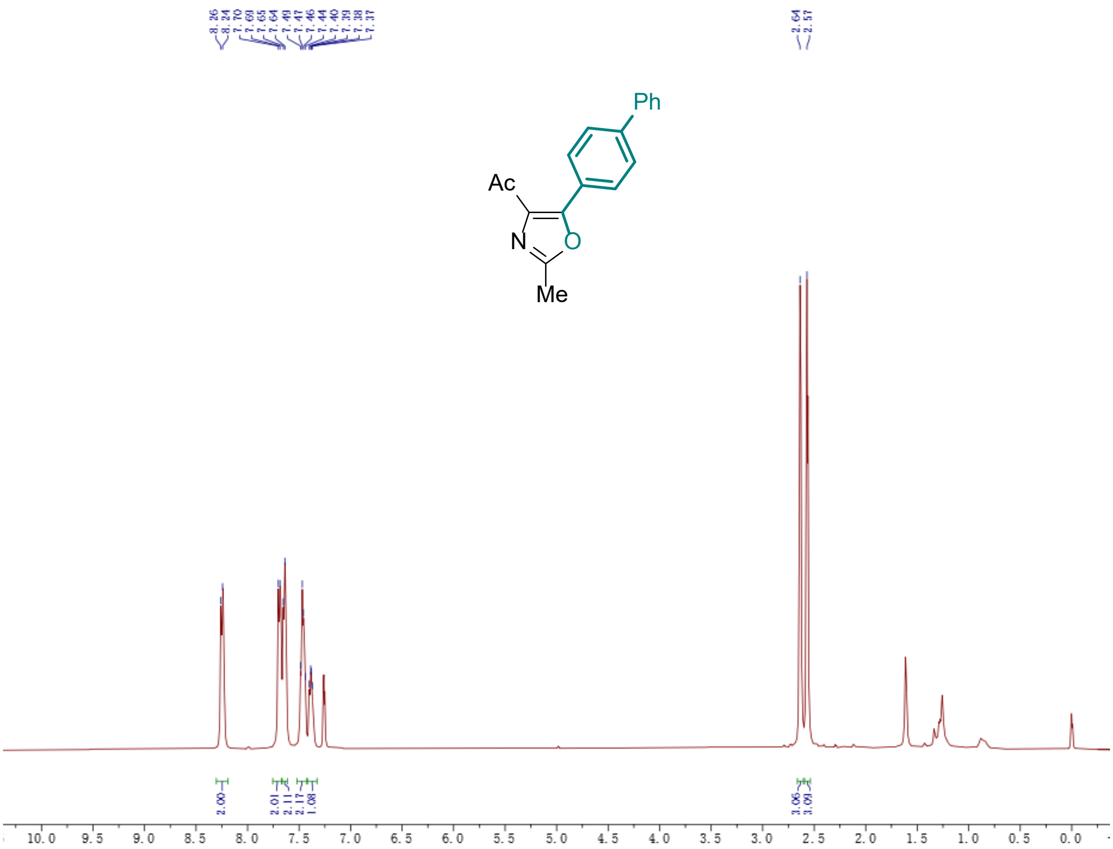

^{1}H NMR of the **3l** (400 MHz, 25 °C in $CDCl_3$)

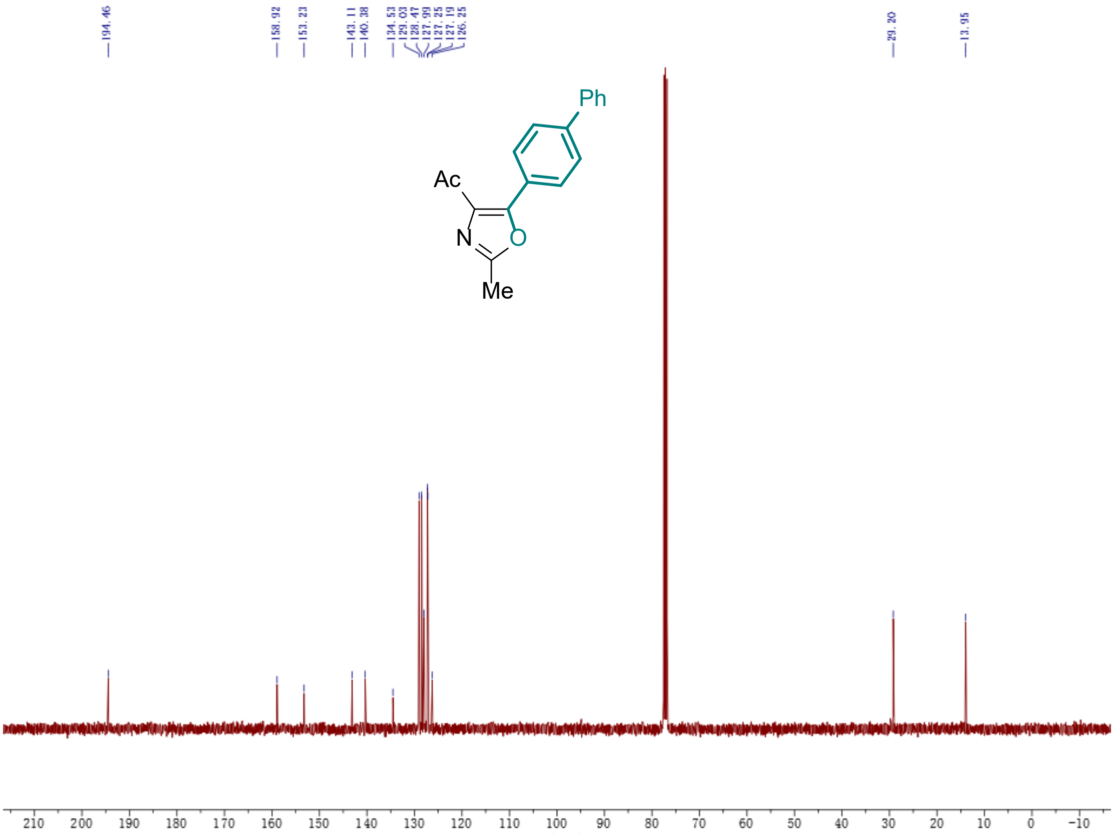

^{13}C NMR of the **3l** (101 MHz, 25 °C in $CDCl_3$)

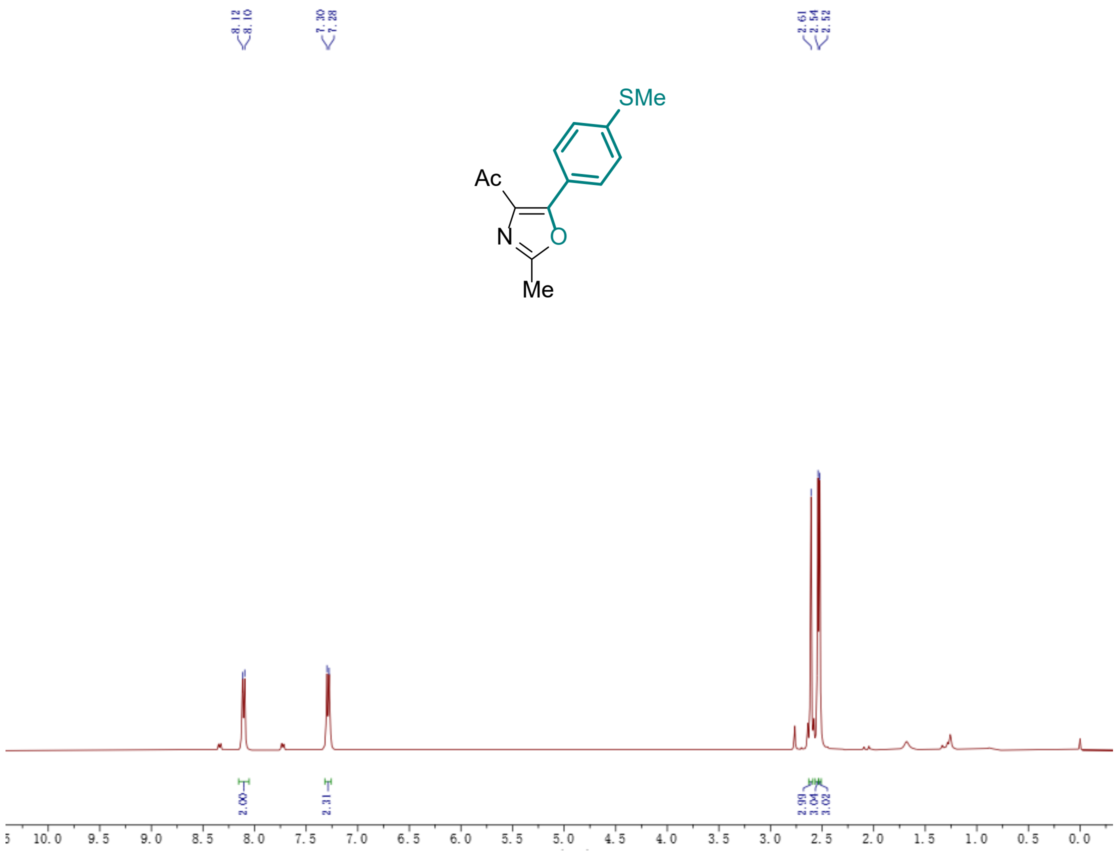

^{1}H NMR of the **3m** (400 MHz, 25 °C in $CDCl_3$)

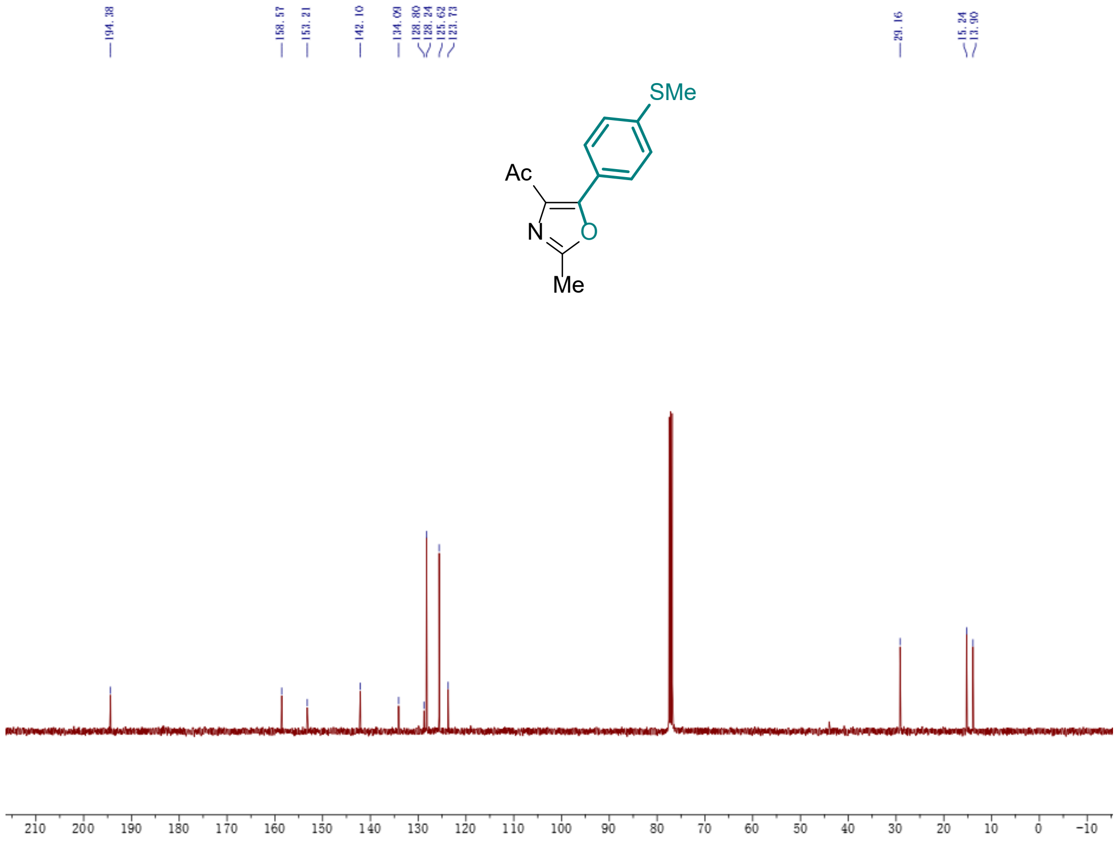

^{13}C NMR of the **3m** (101 MHz, 25 °C in $CDCl_3$)

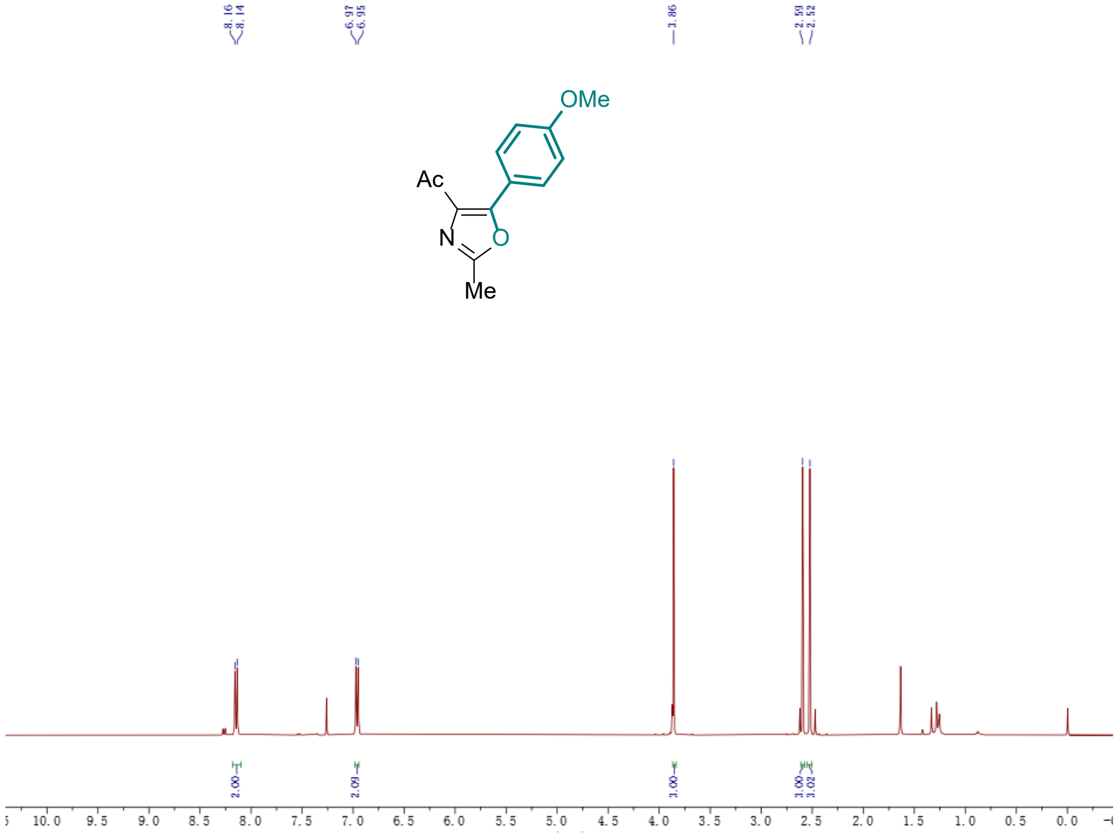

^{1}H NMR of the **3n** (400 MHz, 25 °C in $CDCl_3$)

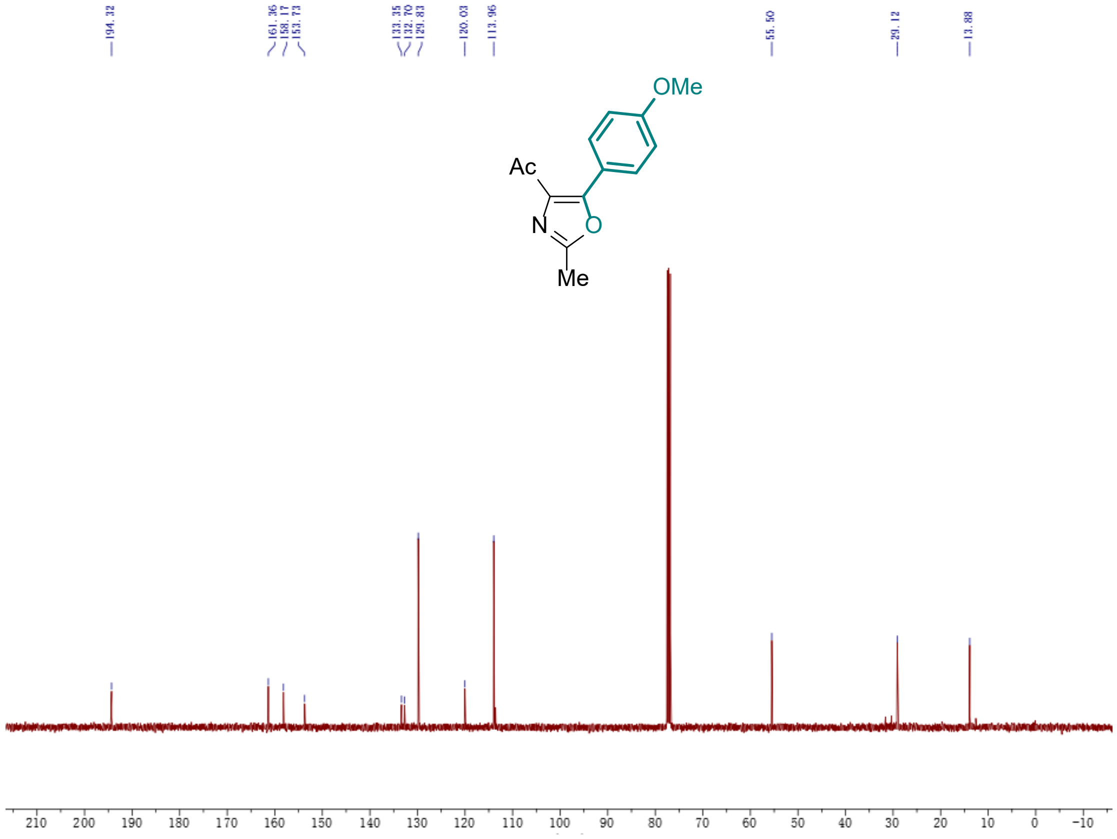

^{13}C NMR of the **3n** (101 MHz, 25 °C in $CDCl_3$)

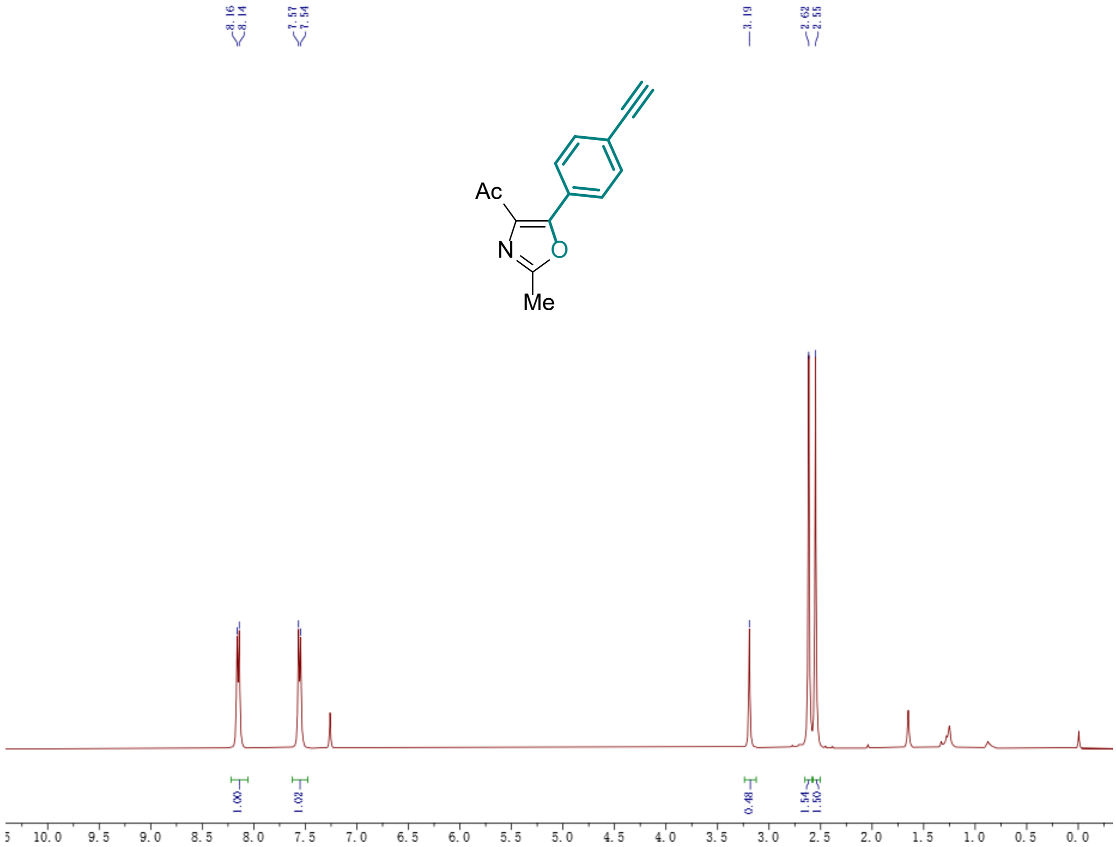

¹H NMR of the **3o** (400 MHz, 25 °C in $CDCl_3$)

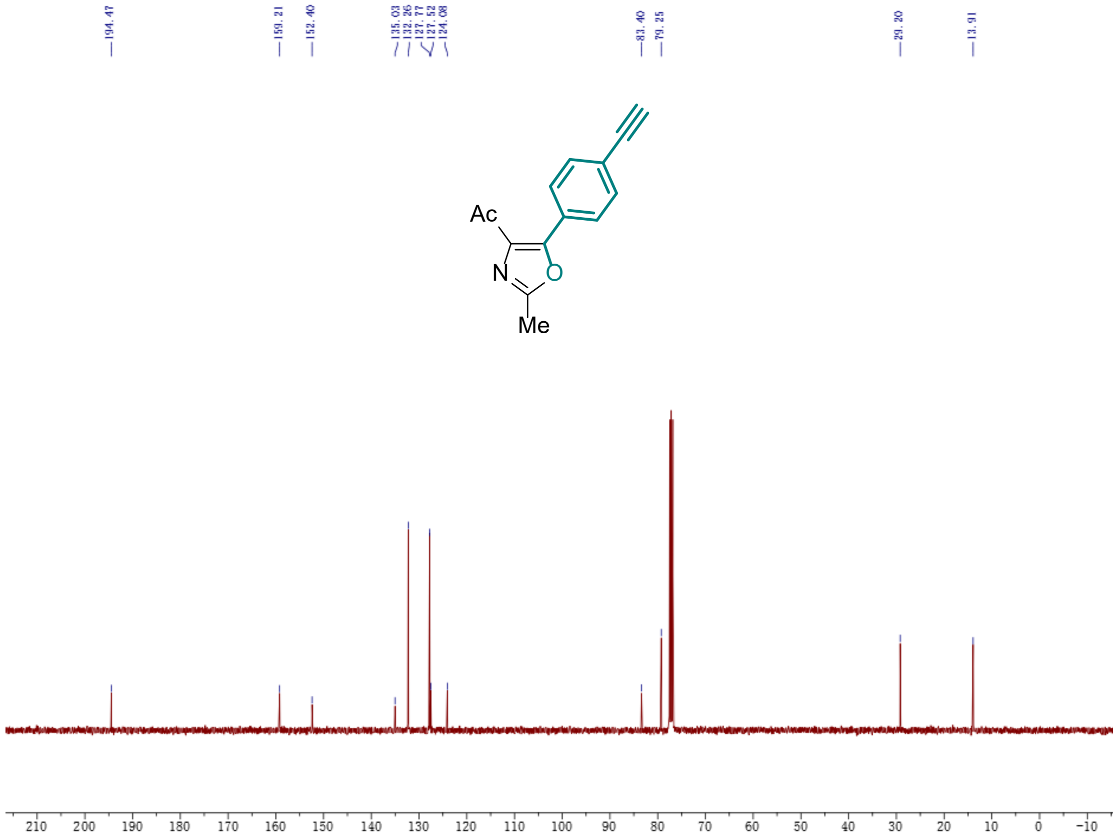

¹³C NMR of the **3o** (101 MHz, 25 °C in $CDCl_3$)

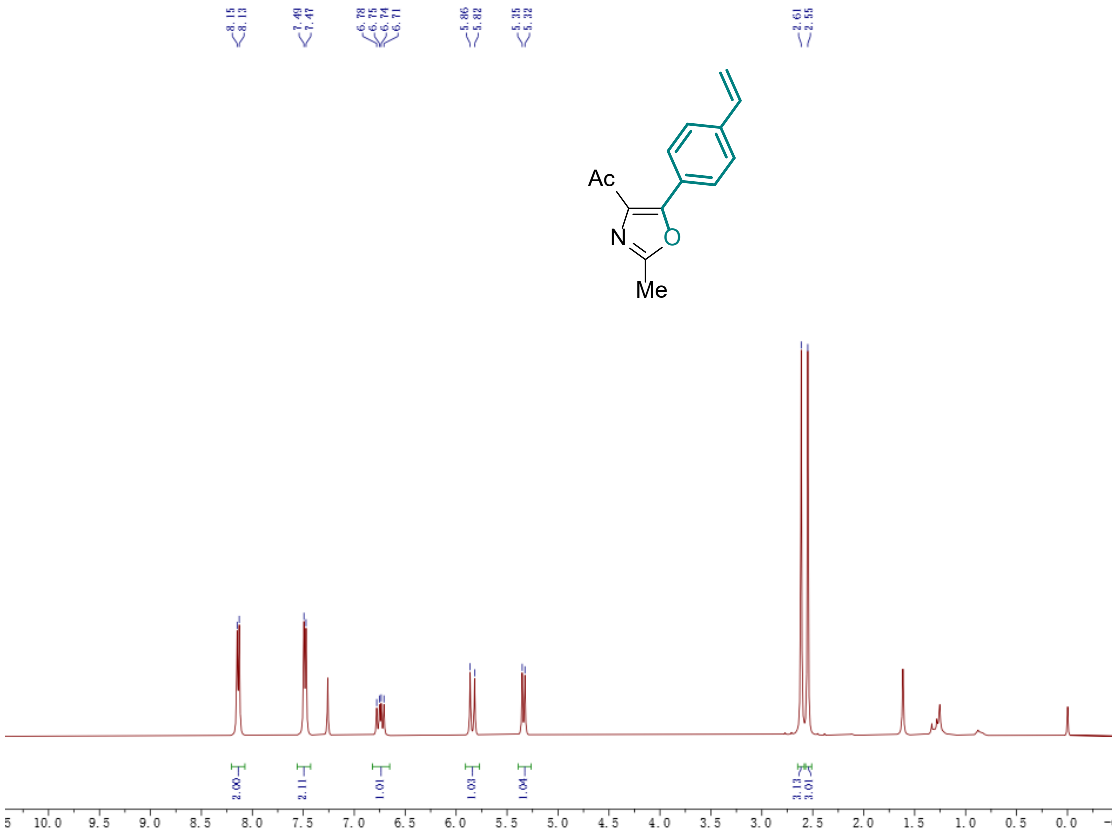

^{1}**H NMR** of the **3p** (400 MHz, 25 °C in $CDCl_3$)

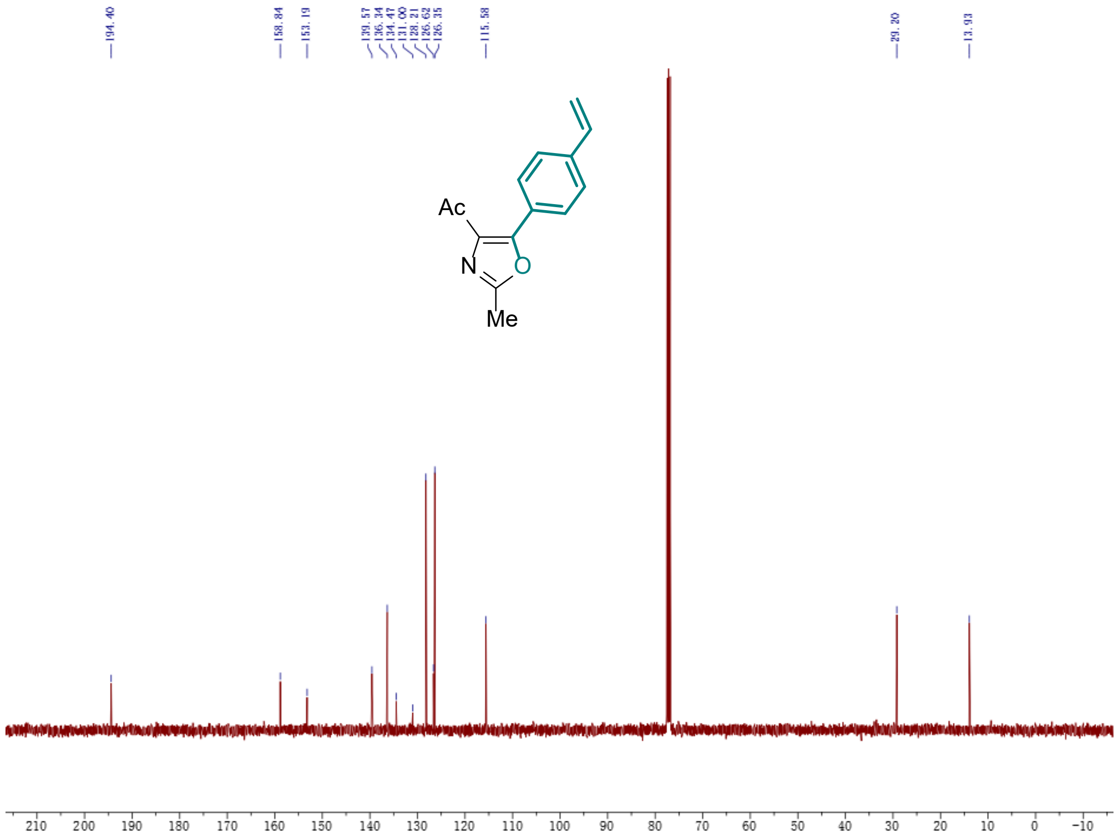

^{13}**C NMR** of the **3p** (101 MHz, 25 °C in $CDCl_3$)

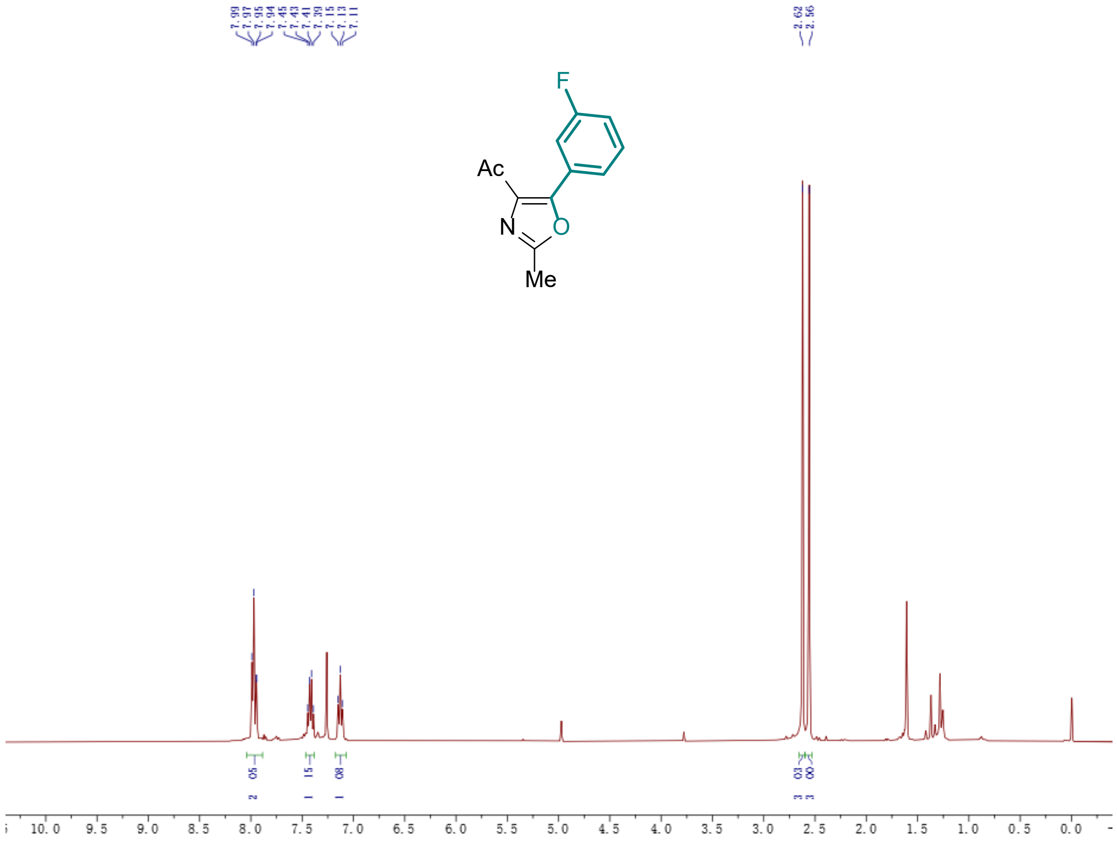

Supplementary Figure 82.

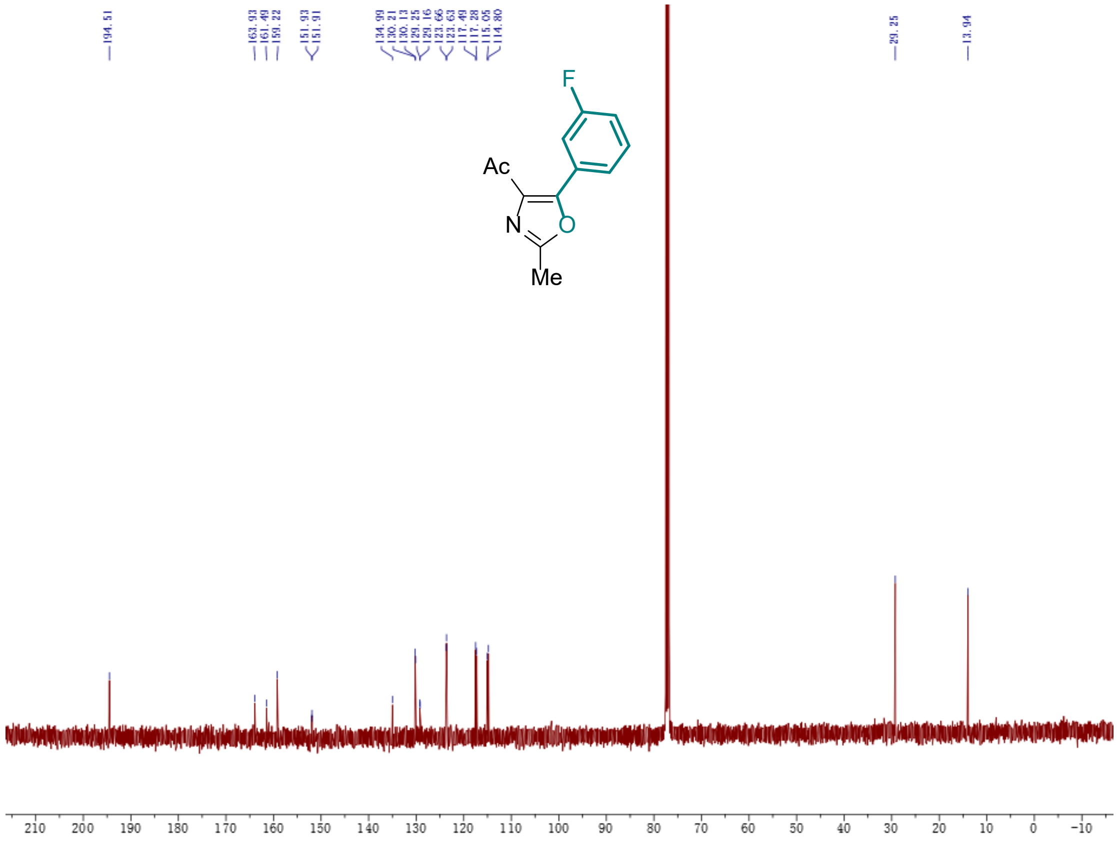

^{13}C NMR of the **3q** (101 MHz, 25 °C in $CDCl_3$)

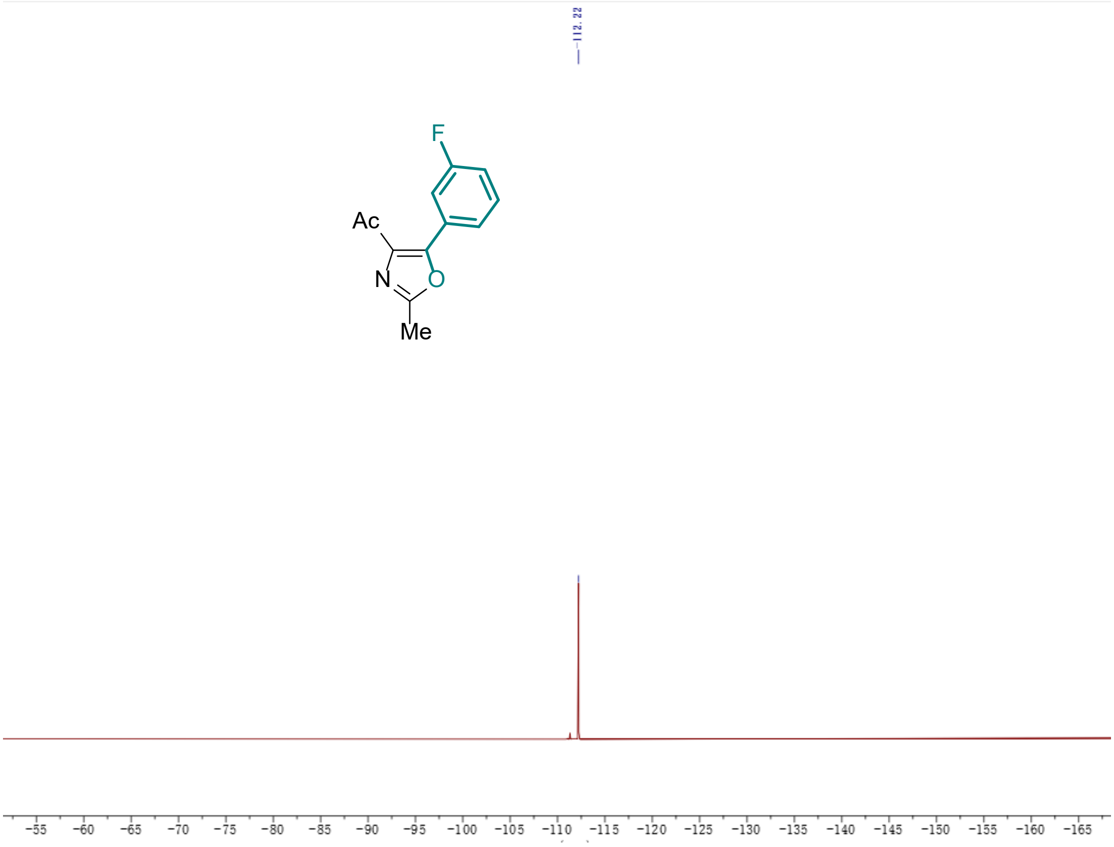

^{19}F NMR of the **3q** (376 MHz, 25 °C in $CDCl_3$)

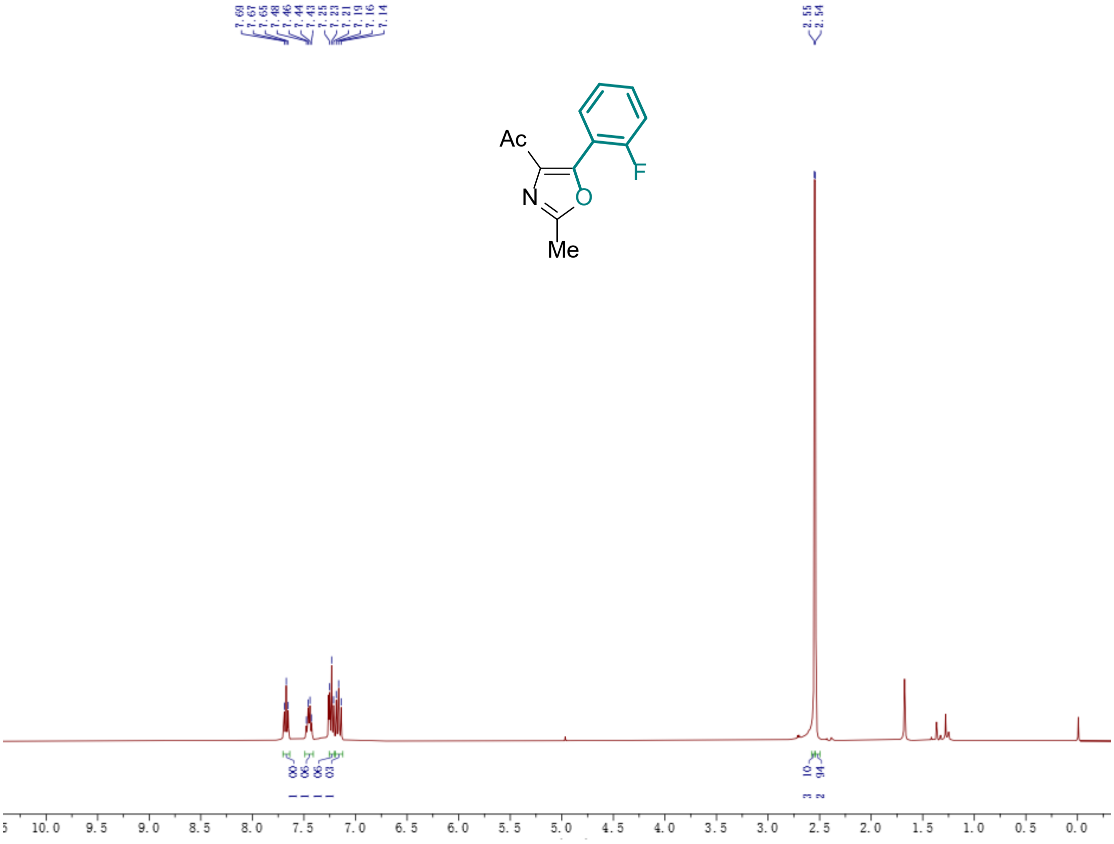

^{1}H NMR of the **3r** (400 MHz, 25 °C in $CDCl_3$)

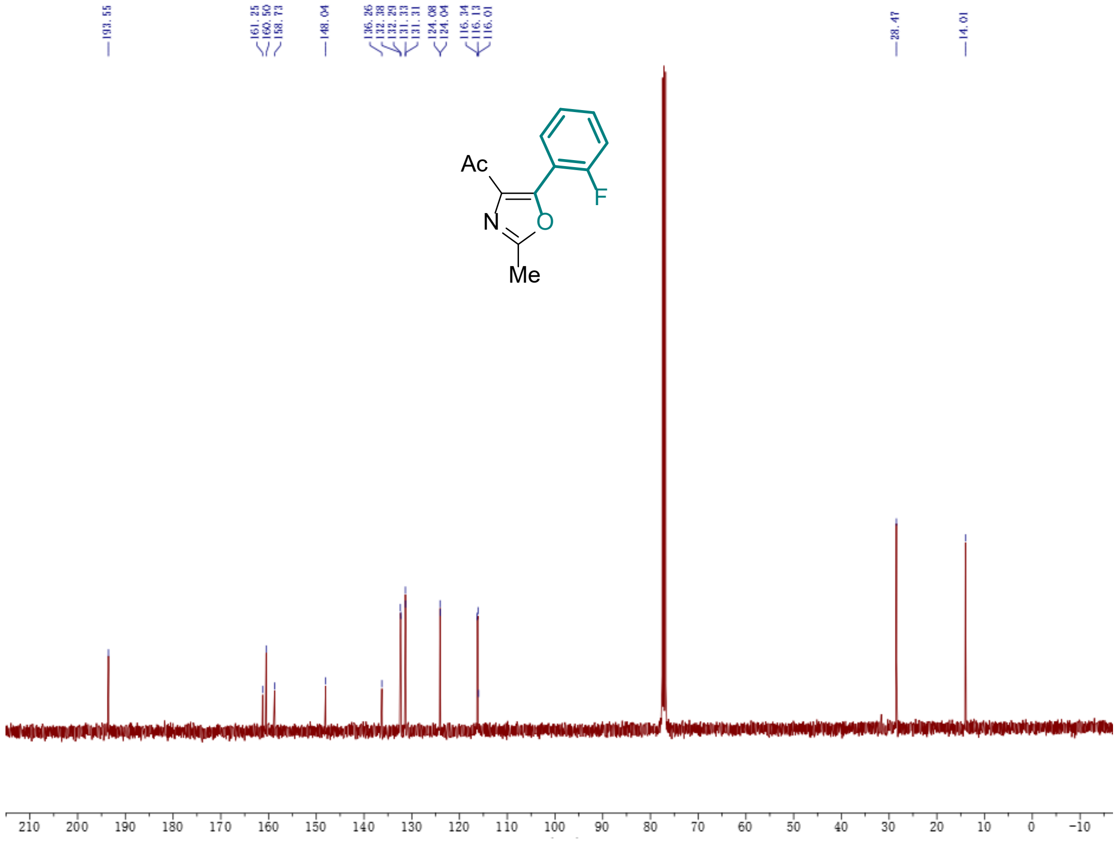

^{13}C NMR of the **3r** (101 MHz, 25 °C in $CDCl_3$)

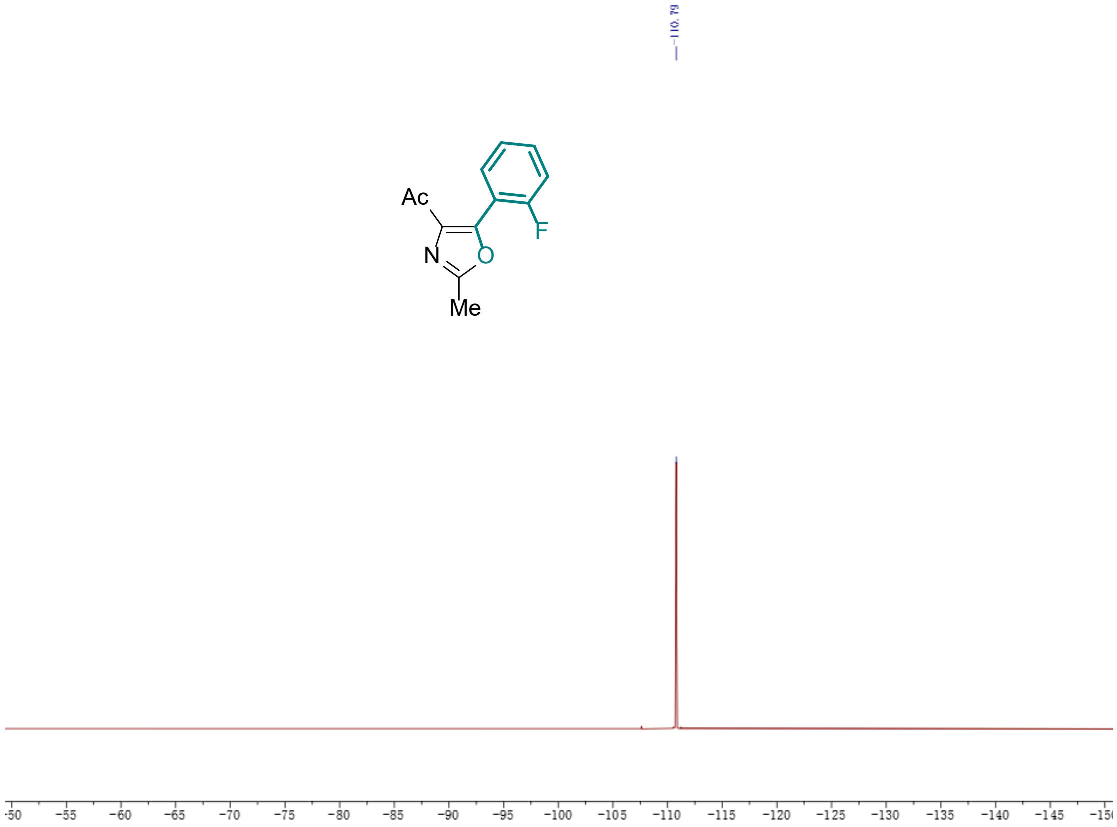

^{19}F NMR of the **3r** (376 MHz, 25 °C in CDCl3)

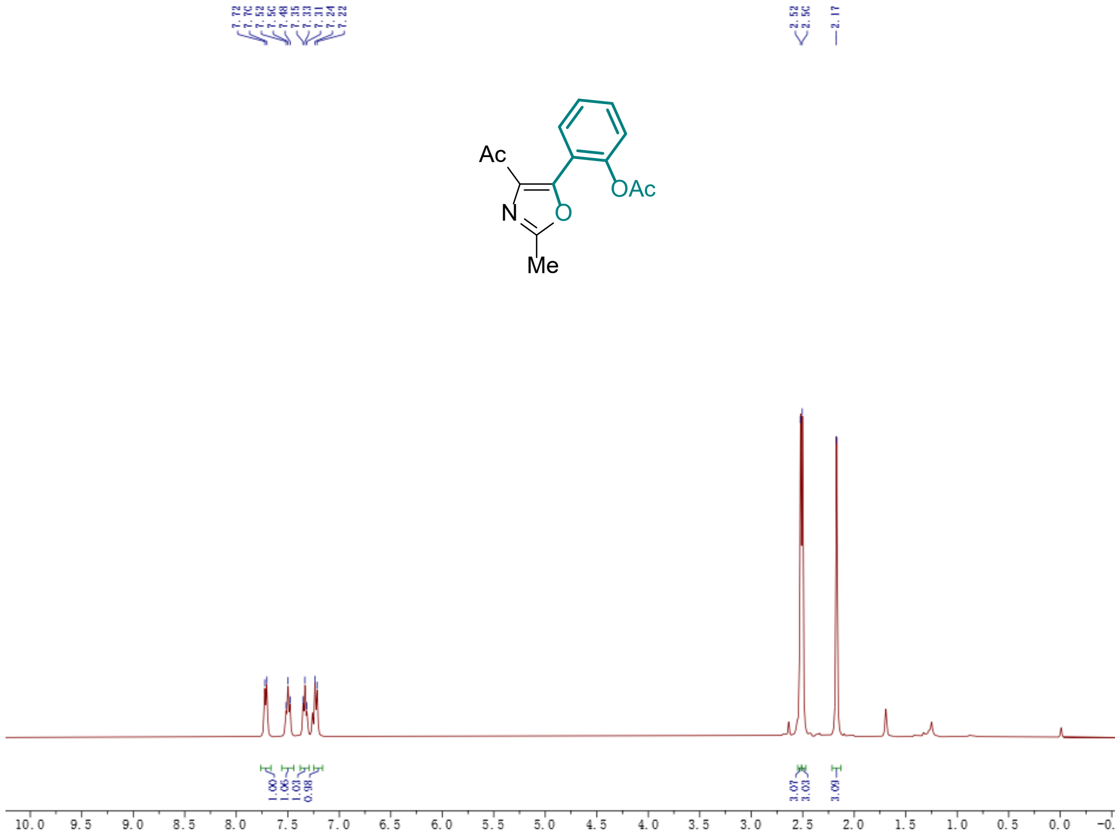

^{1}H NMR of the **3s** (400 MHz, 25 °C in CDCl3)

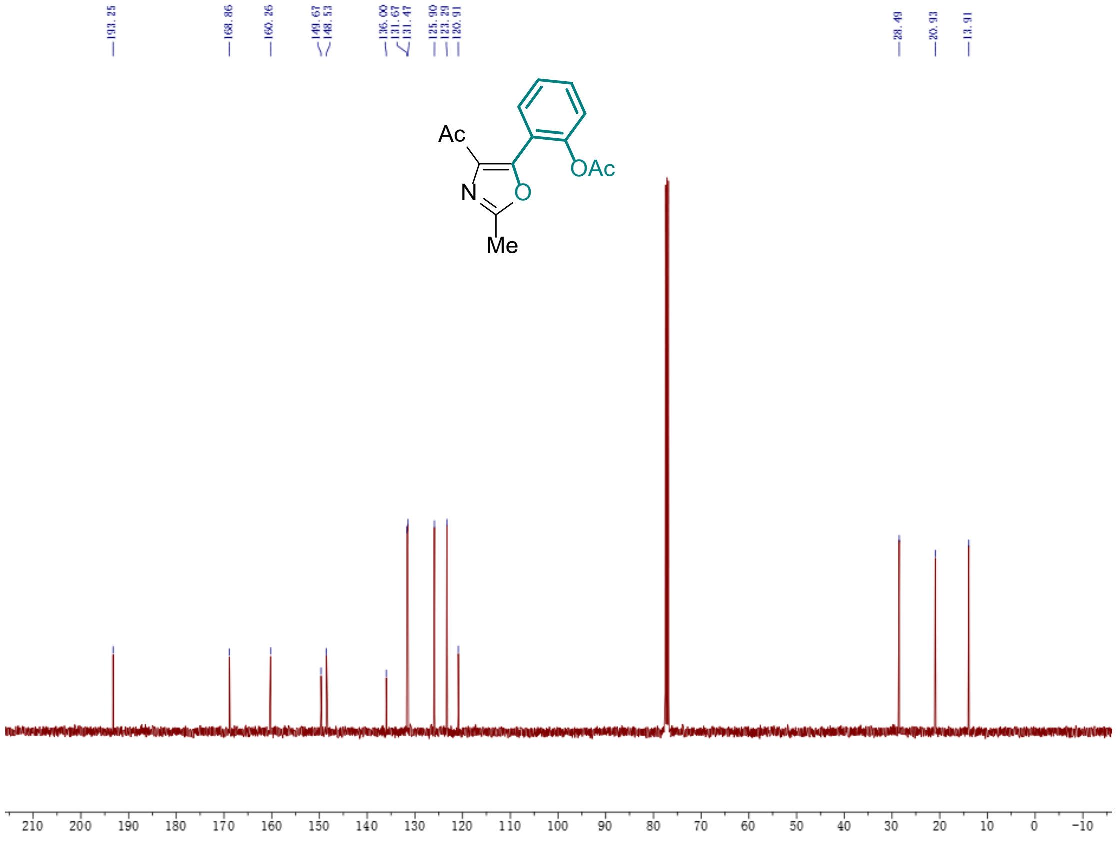

^{13}C NMR of the **3s** (101 MHz, 25 °C in $CDCl_3$)

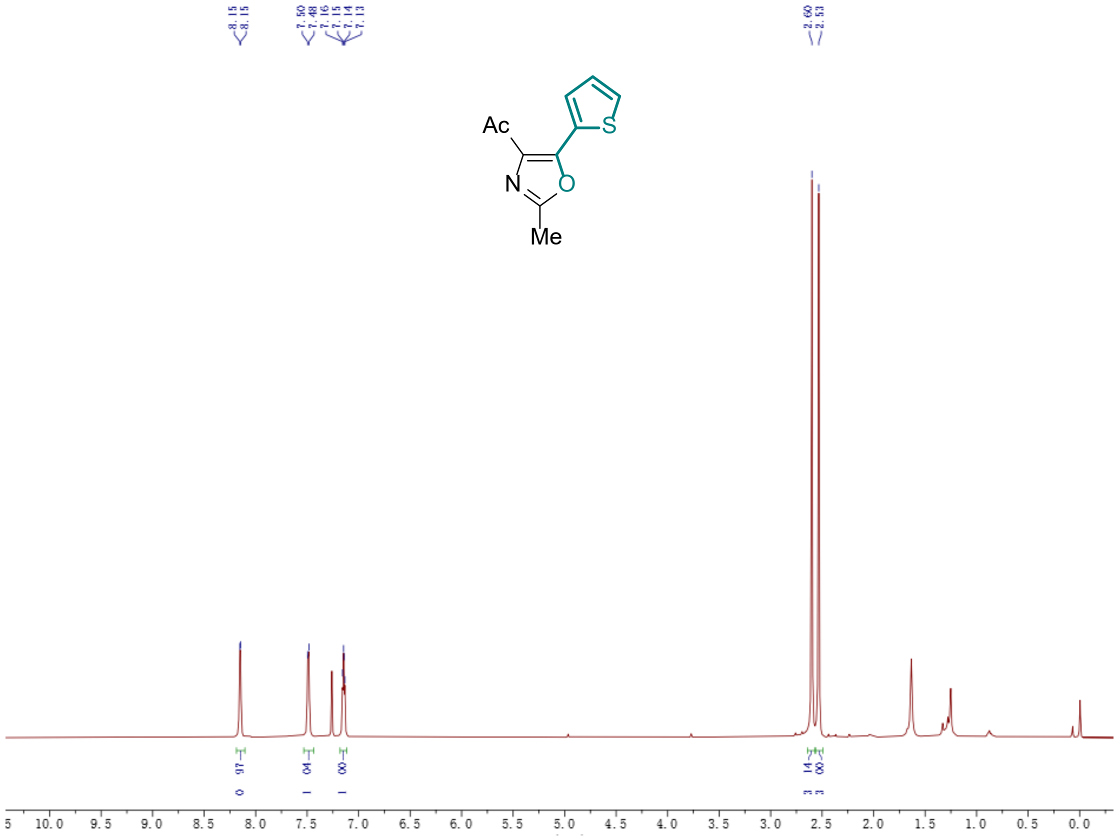

^{1}H NMR of the **3t** (400 MHz, 25 °C in $CDCl_3$)

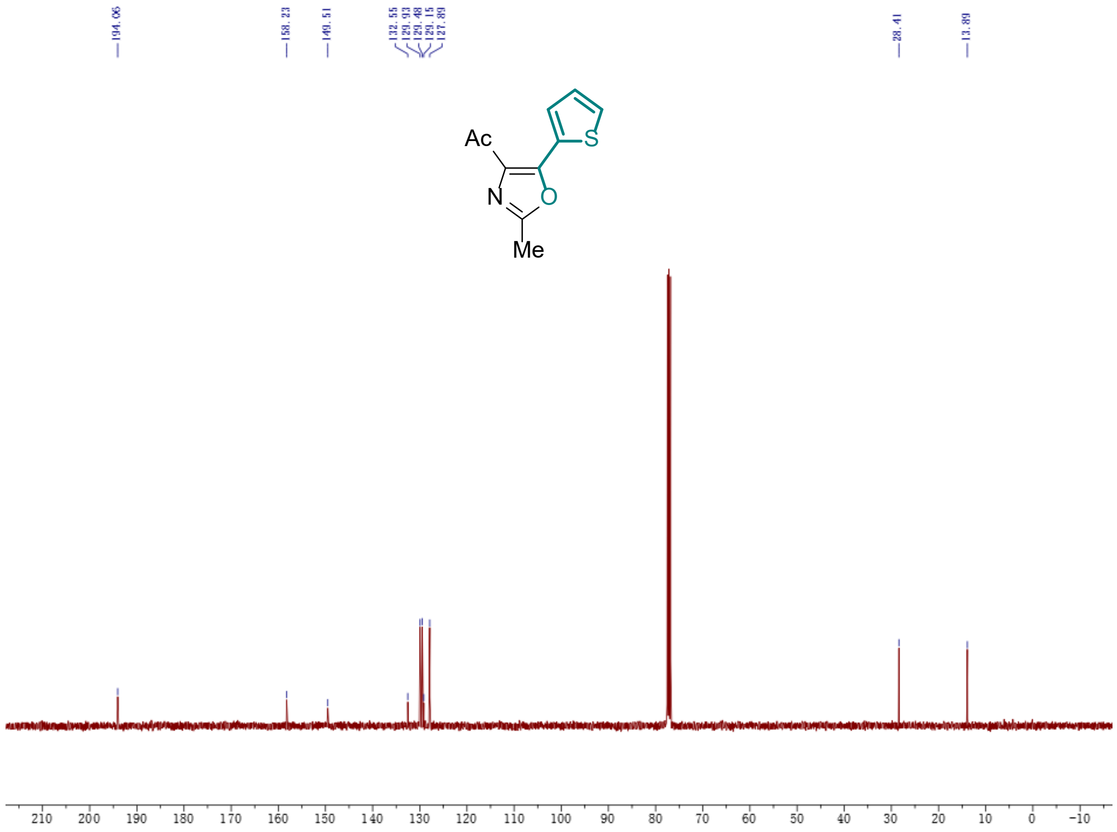

^{13}C NMR of the 3t (101 MHz, 25 °C in $CDCl_3$)

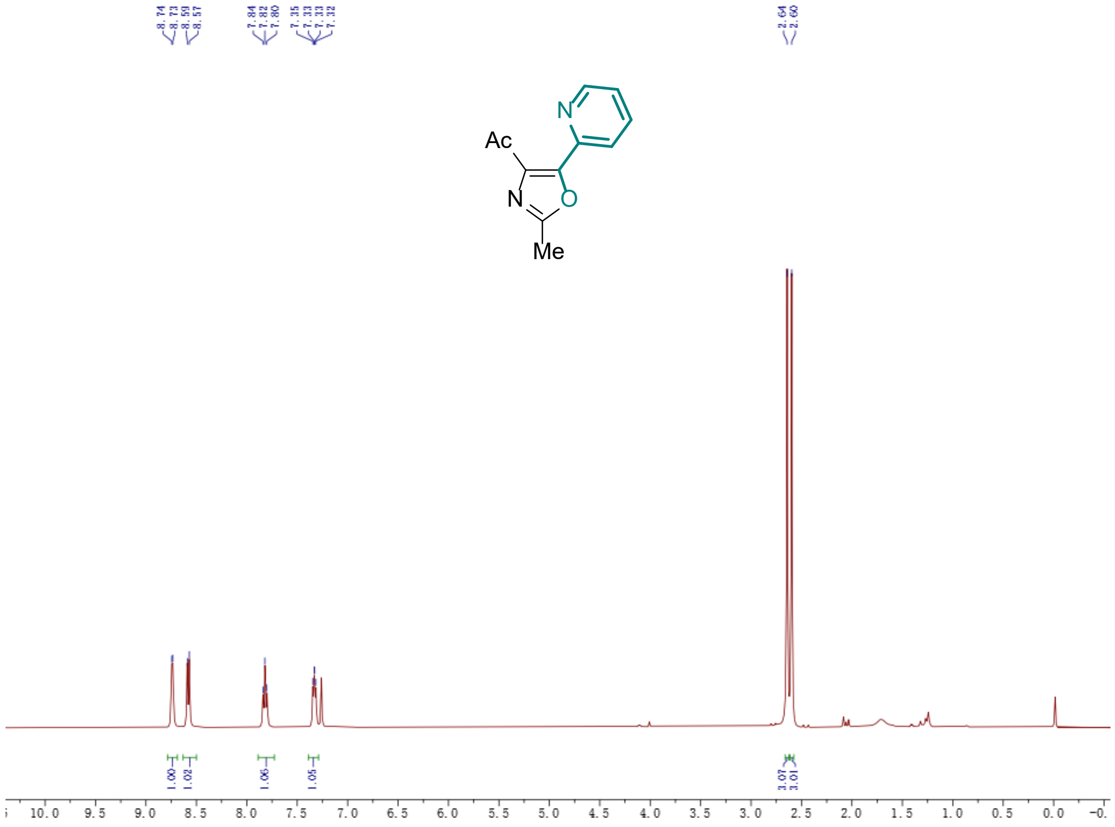

^{1}H NMR of the 3u (400 MHz, 25 °C in $CDCl_3$)

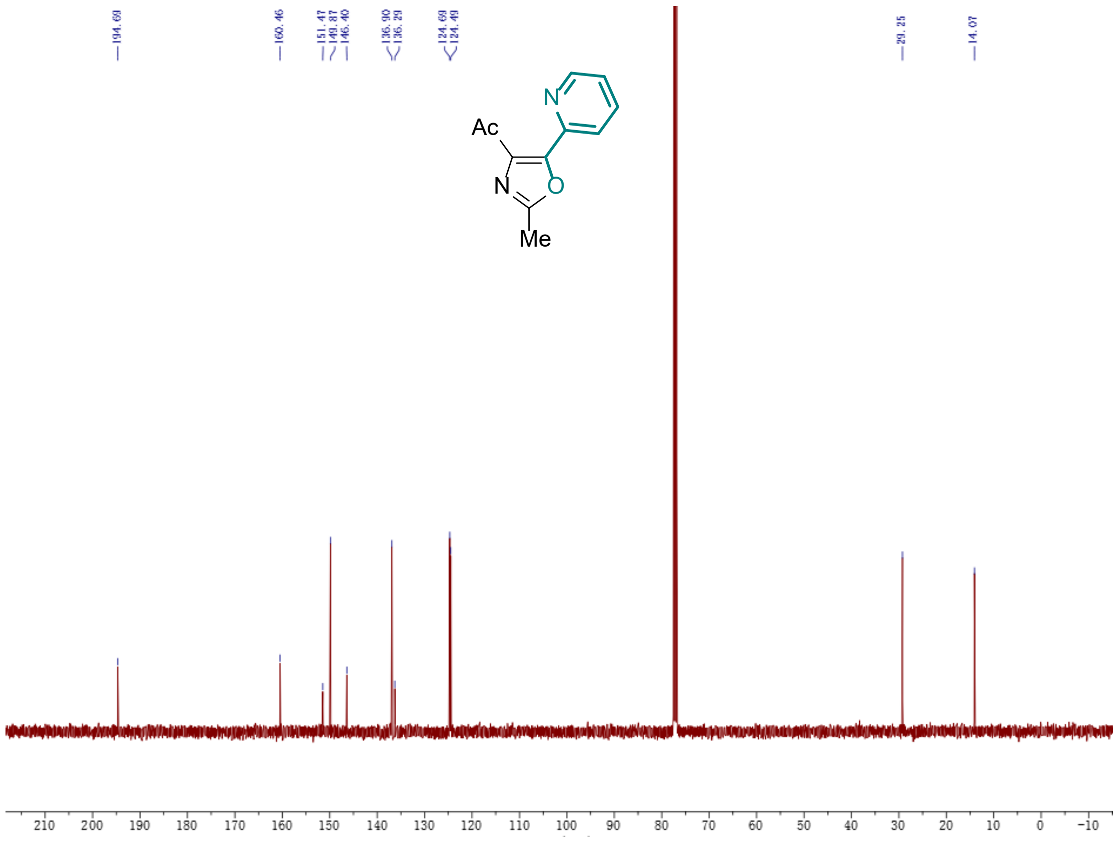

^{13}**C NMR** of the **3u** (101 MHz, 25 °C in $CDCl_3$)

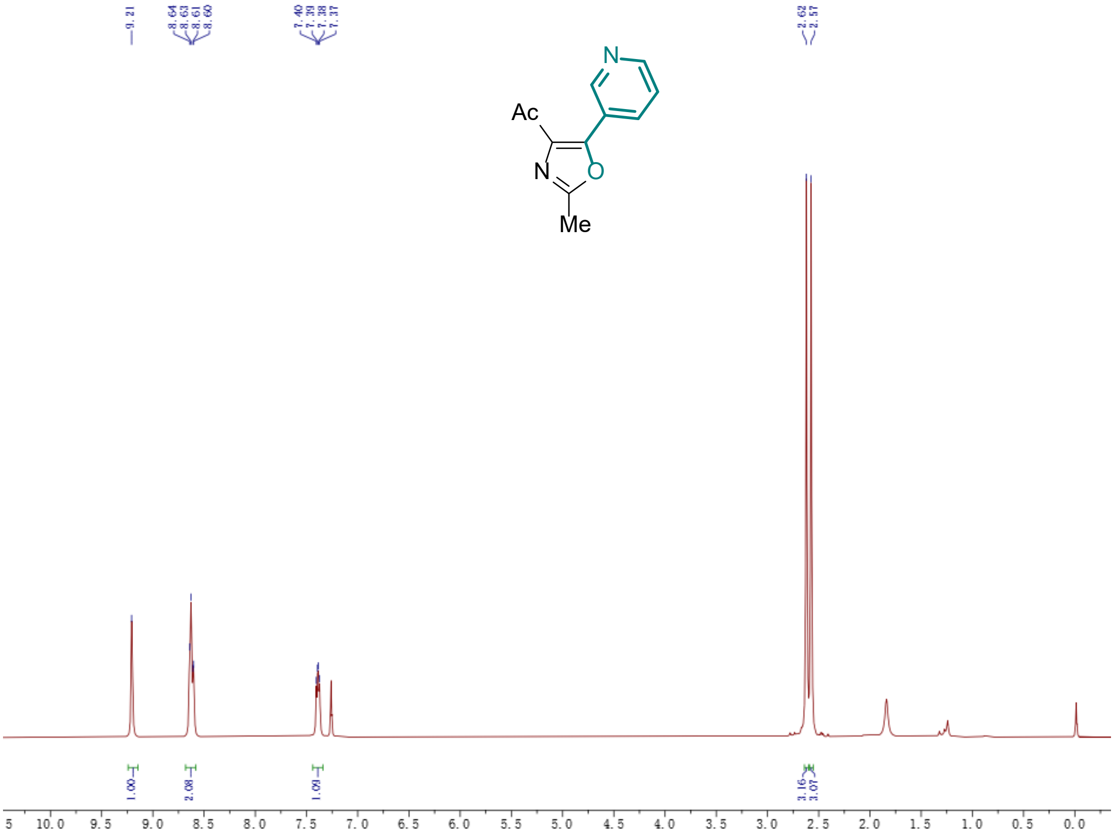

^{1}**H NMR** of the **3v** (400 MHz, 25 °C in $CDCl_3$)

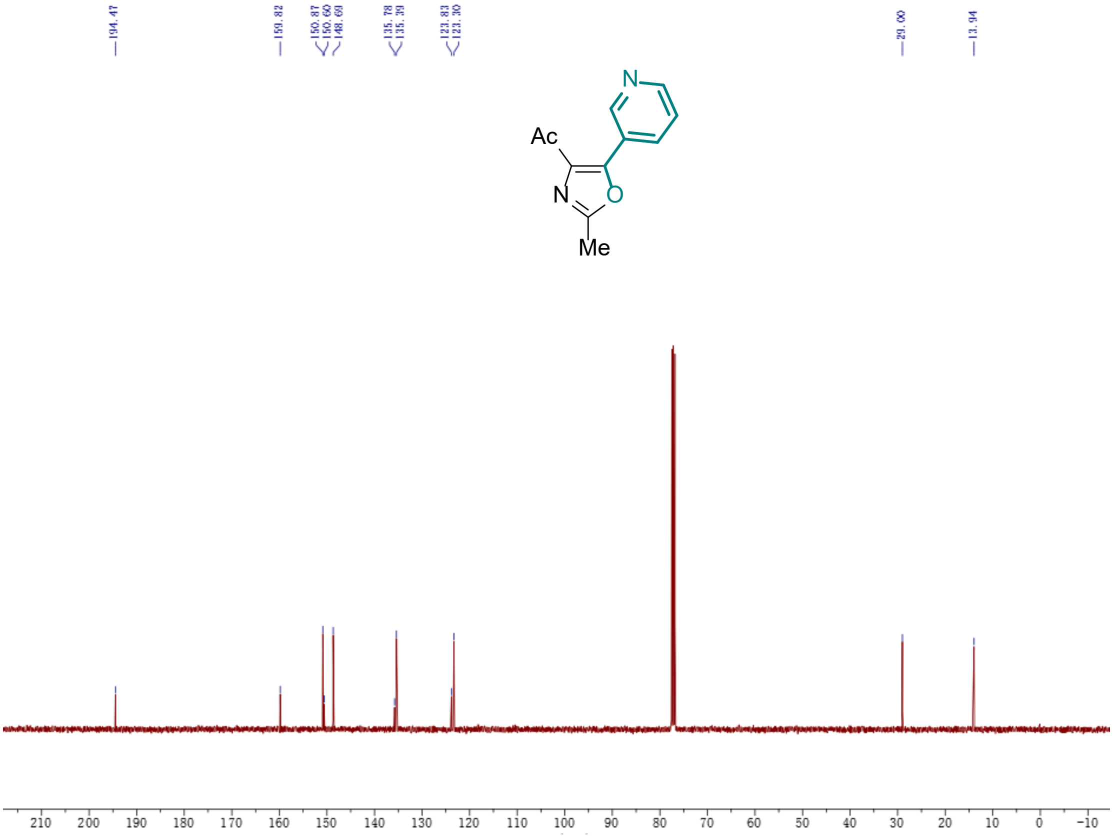

^{13}C NMR of the 3v (101 MHz, 25 °C in $CDCl_3$)

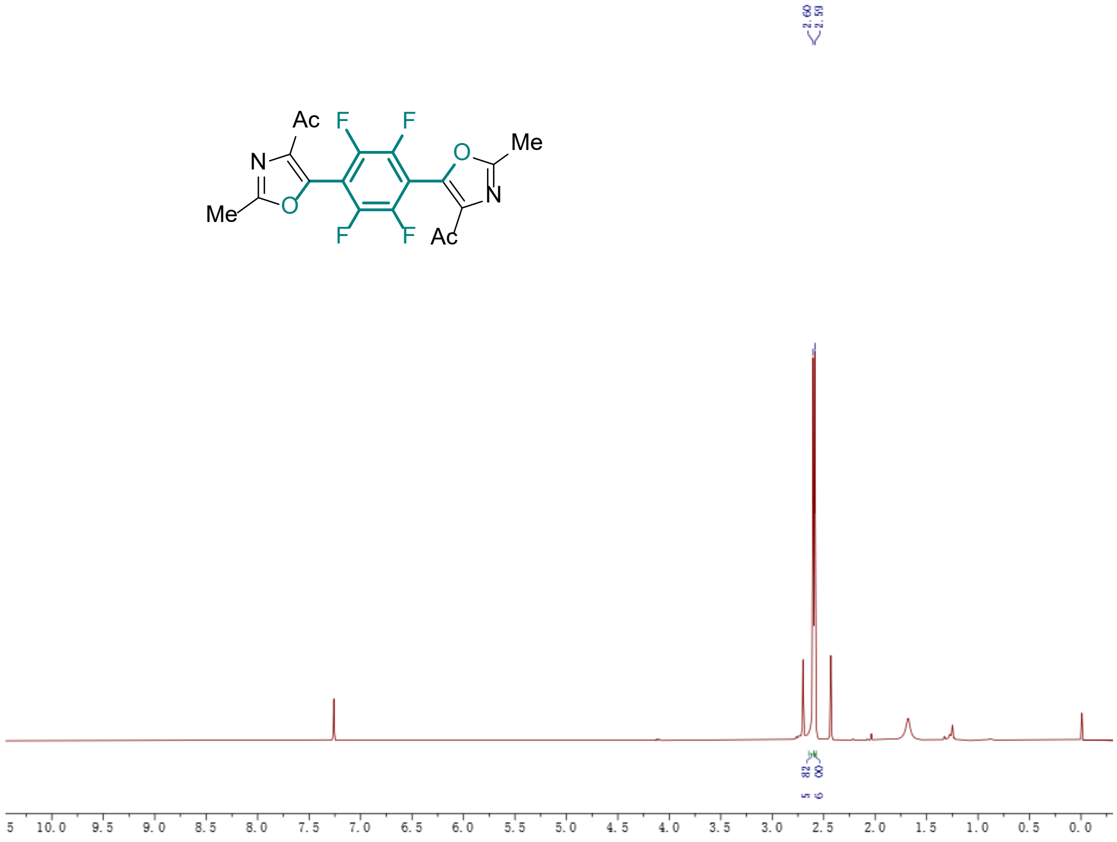

^{1}H NMR of the 3w (400 MHz, 25 °C in $CDCl_3$)

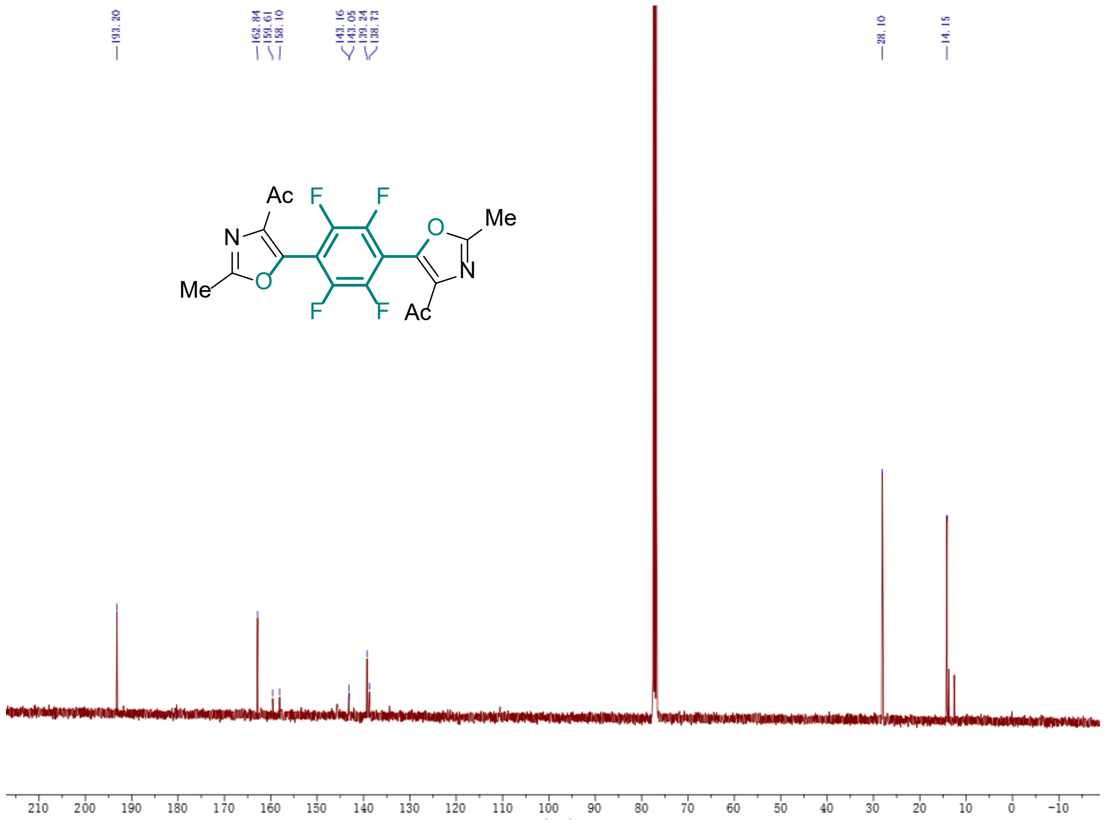

^{13}C NMR of the **3w** (101 MHz, 25 °C in CDCl3)

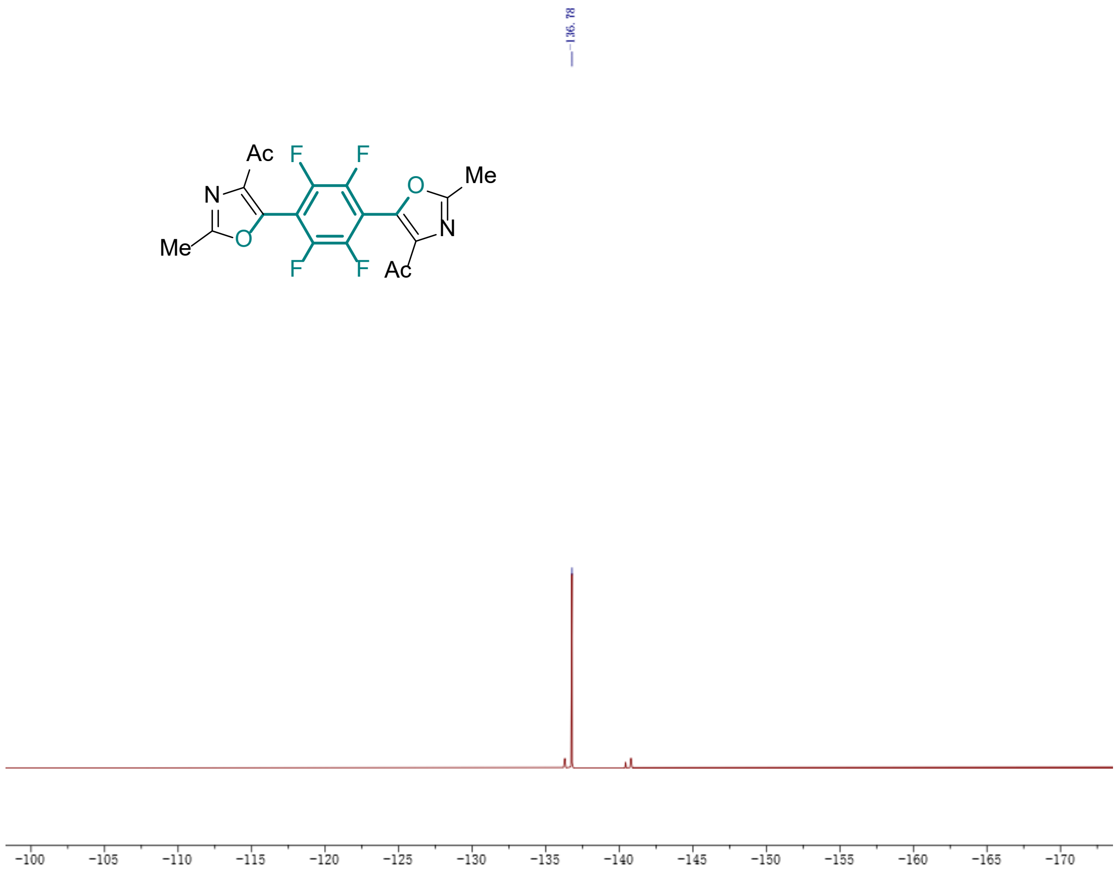

^{19}F NMR of the **3w** (376 MHz, 25 °C in CDCl3)

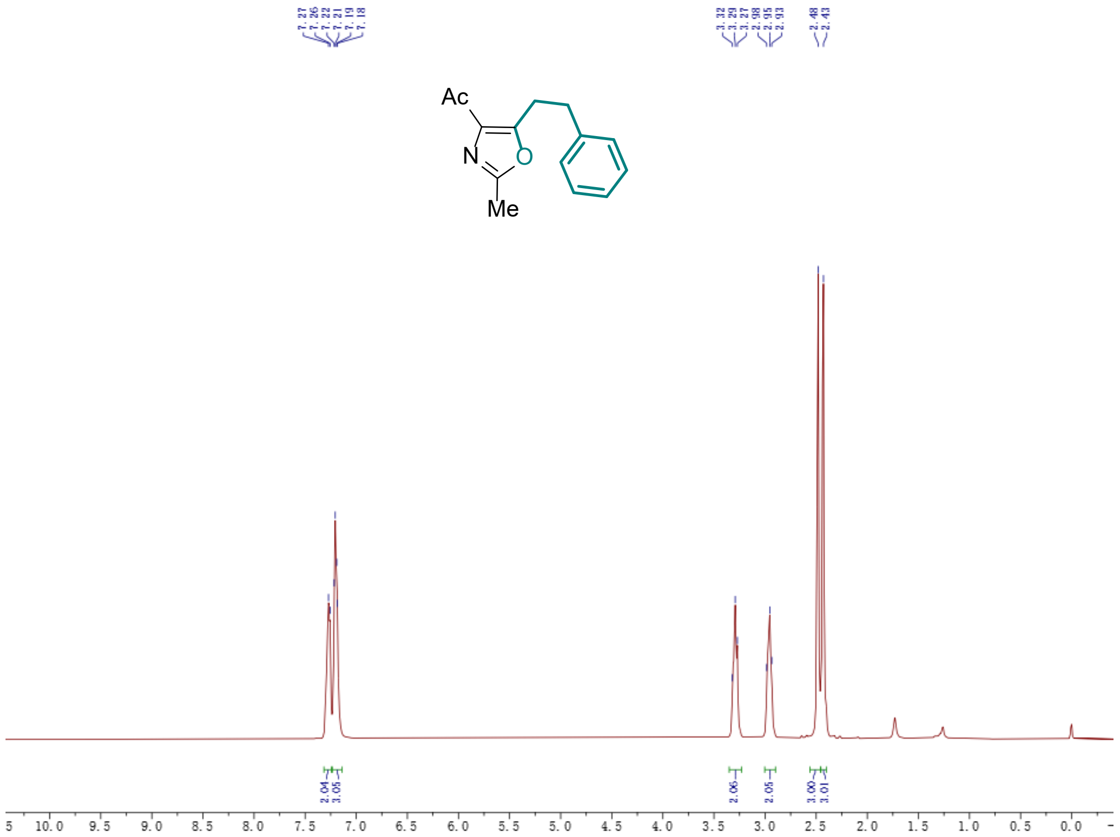

^{1}H NMR of the 3x (400 MHz, 25 °C in CDCl3)

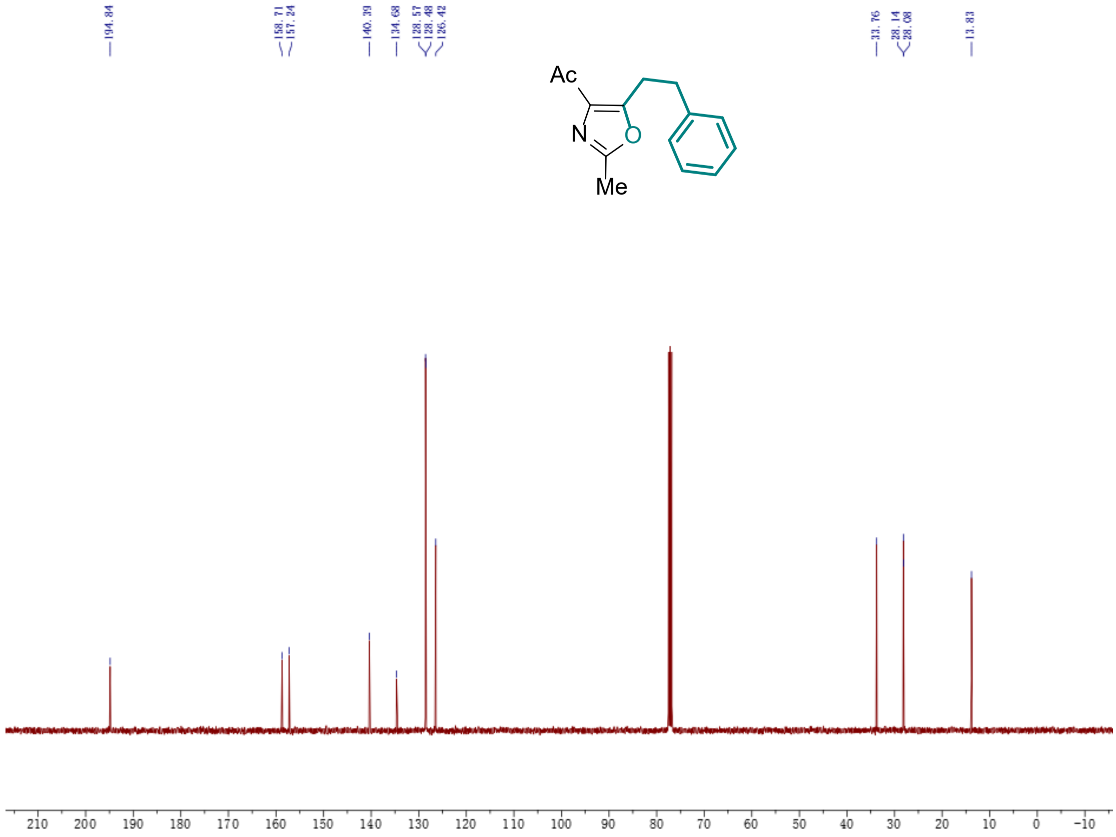

^{13}C NMR of the 3x (101 MHz, 25 °C in CDCl3)

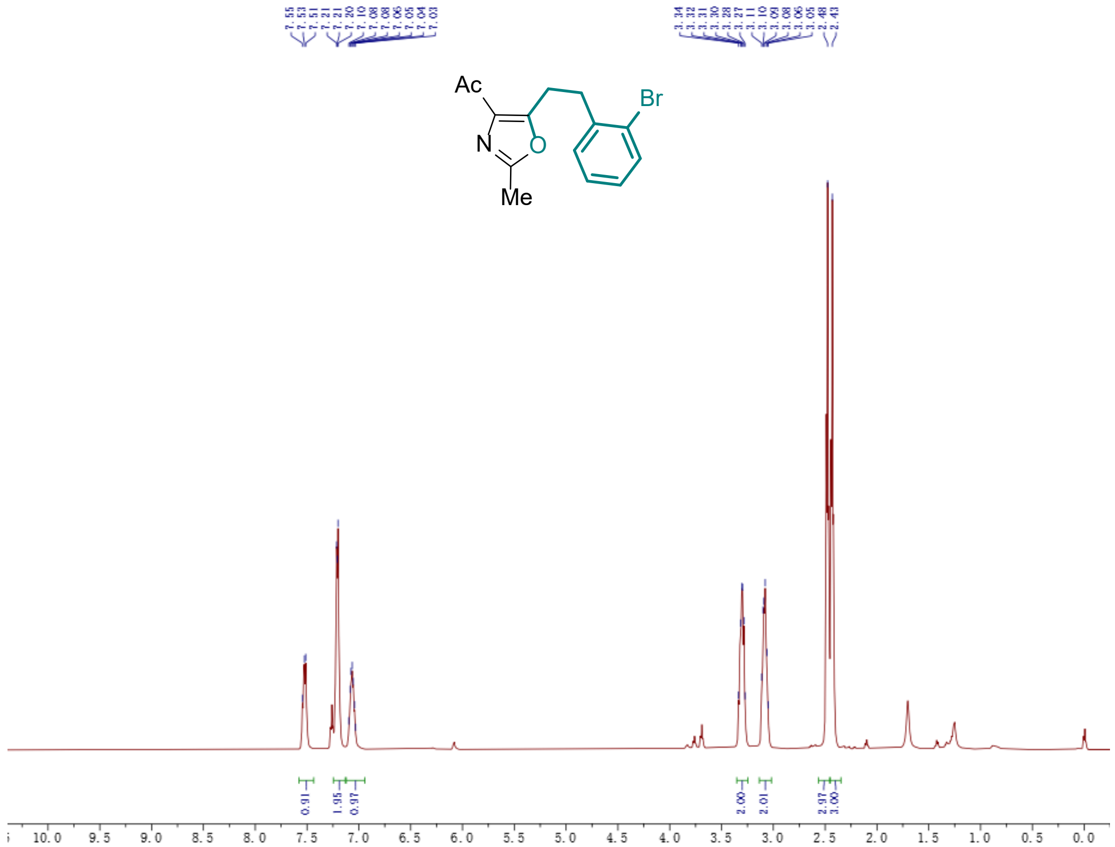

^{1}H NMR of the **3y** (400 MHz, 25 °C in CDCl3)

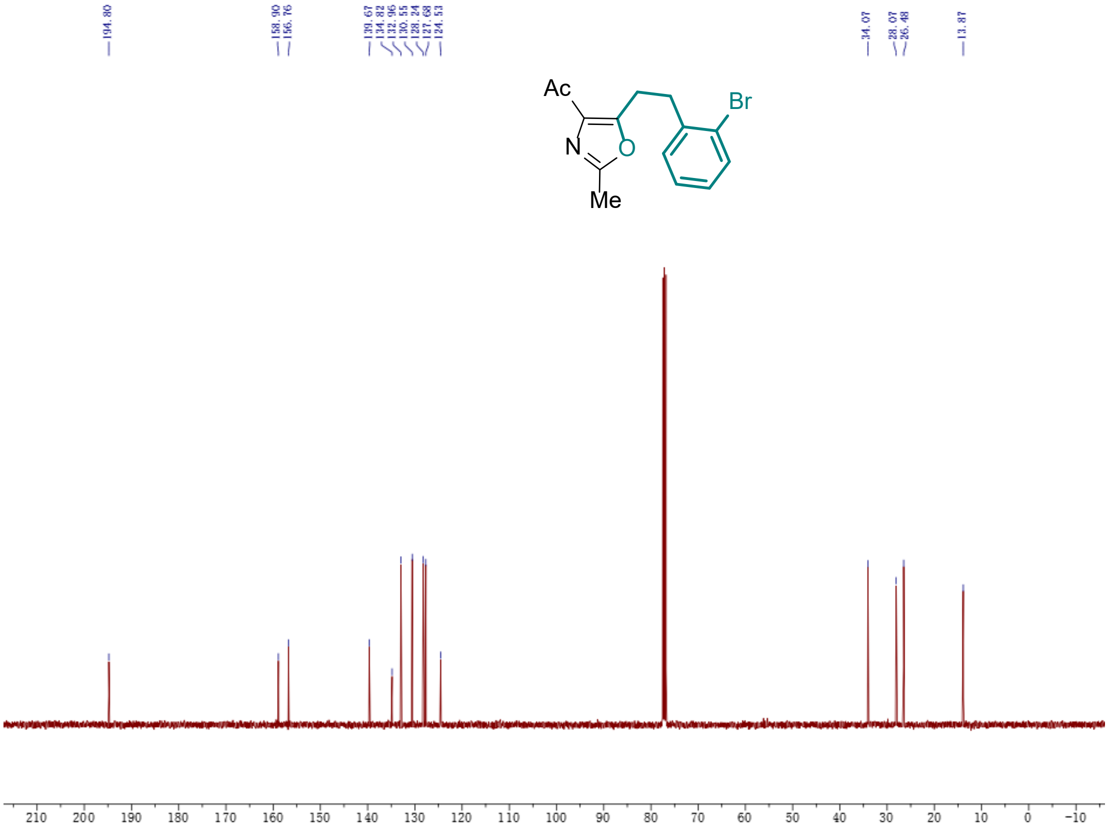

^{13}C NMR of the **3y** (101 MHz, 25 °C in CDCl3)

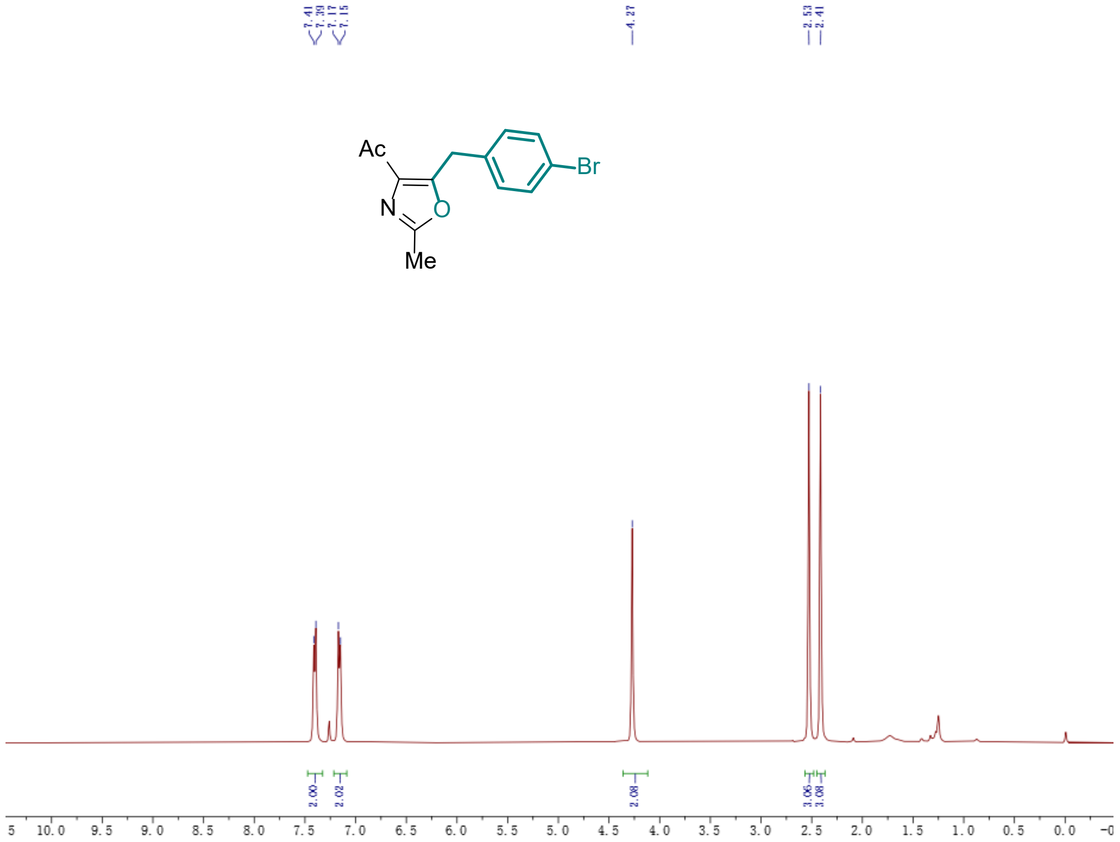

^{1}H NMR of the **3z** (400 MHz, 25 °C in $CDCl_3$)

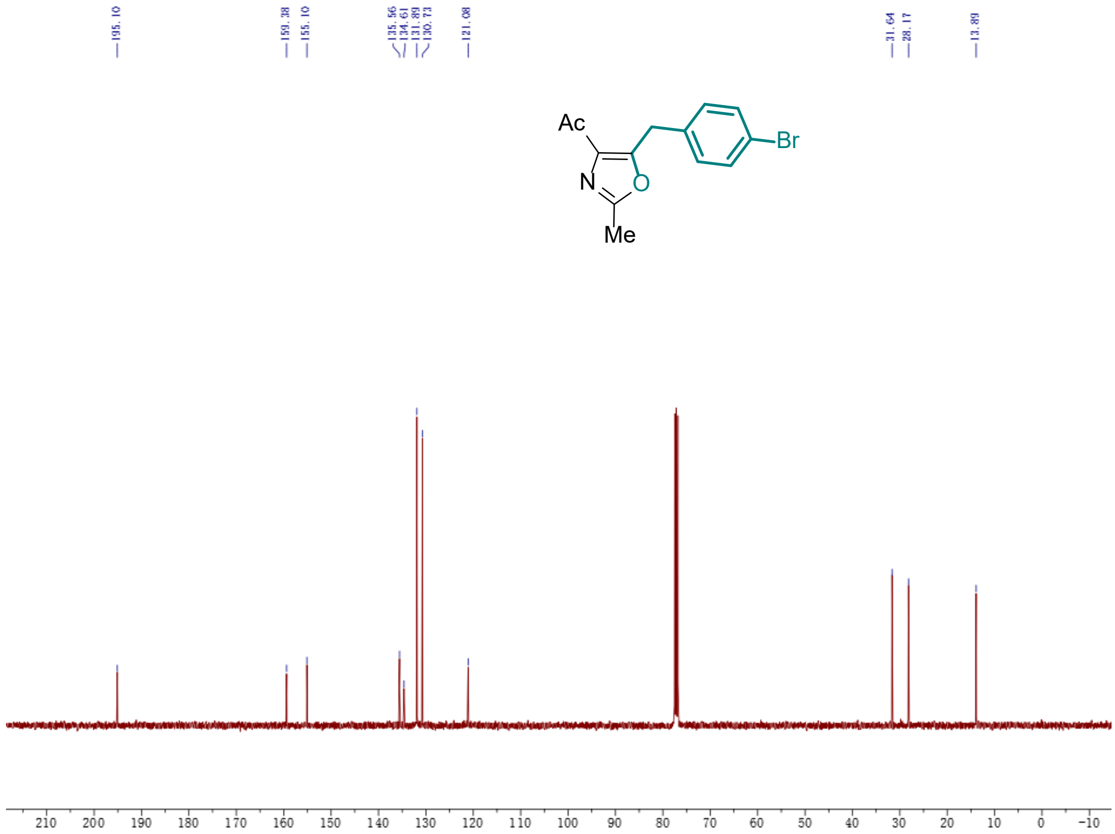

^{13}C NMR of the **3z** (101 MHz, 25 °C in $CDCl_3$)

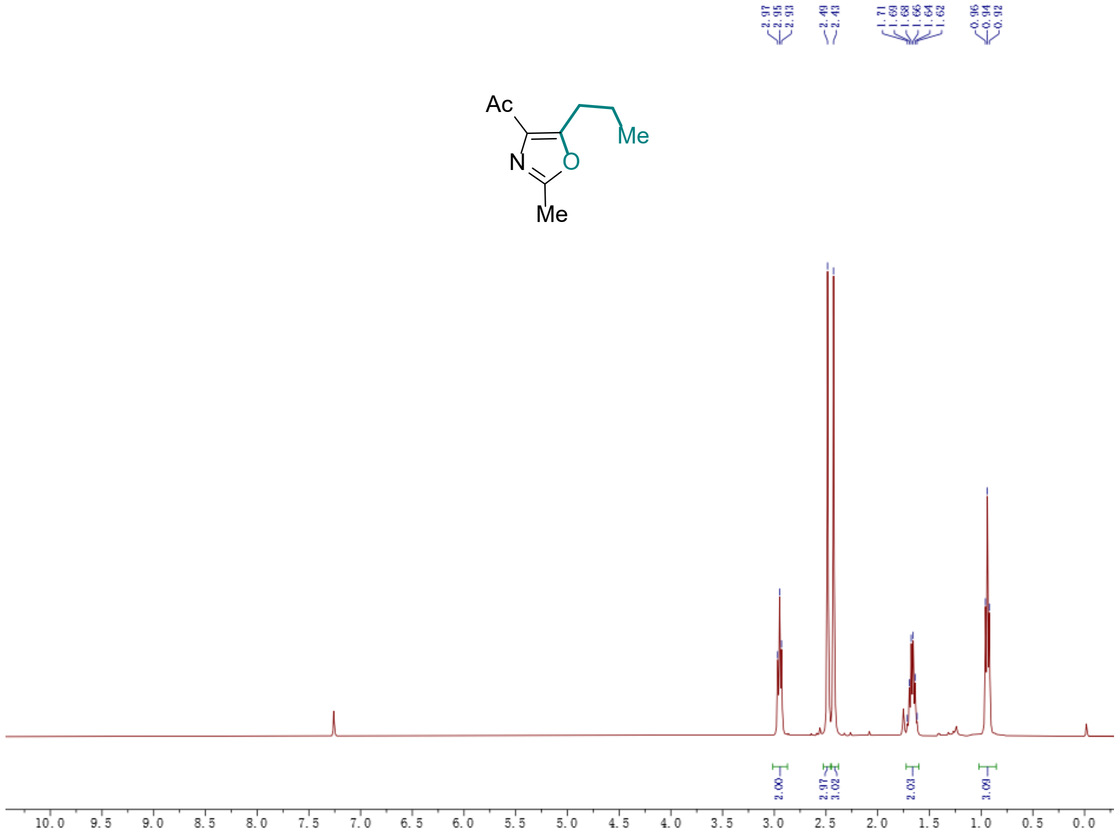

1H NMR of the **3aa** (400 MHz, 25 °C in CDCl3)

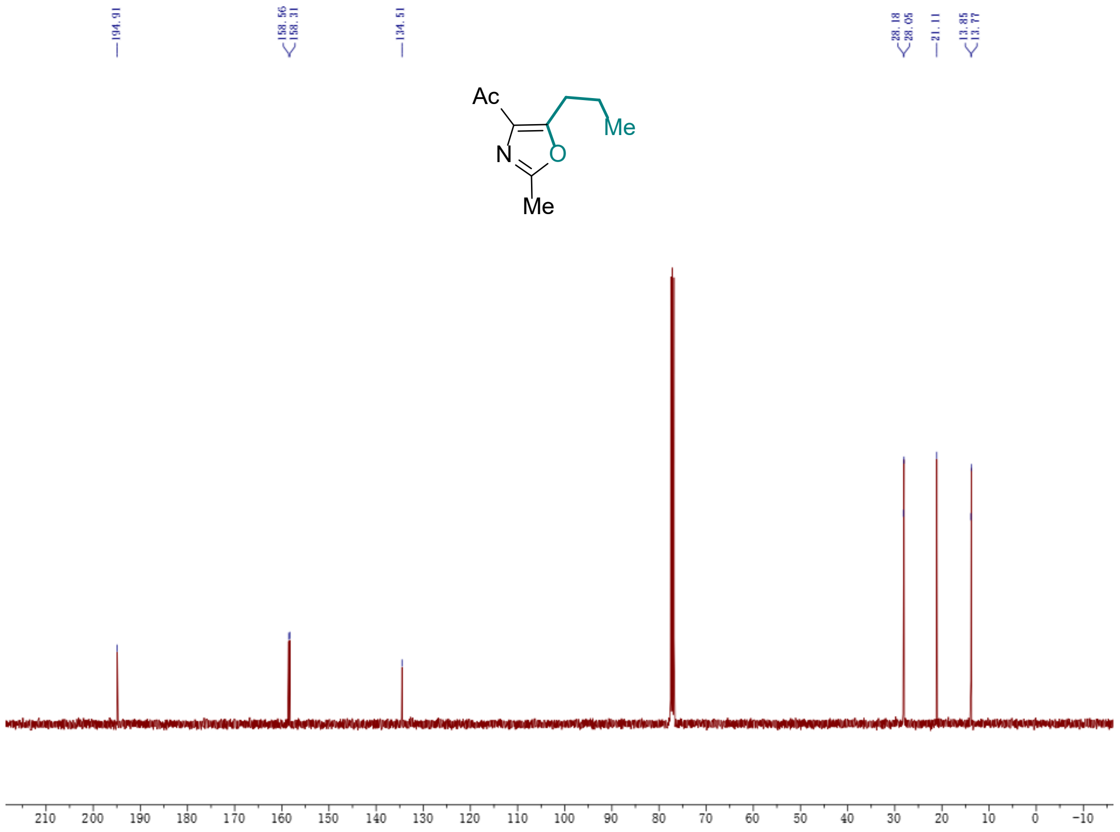

13C NMR of the **3aa** (101 MHz, 25 °C in CDCl3)

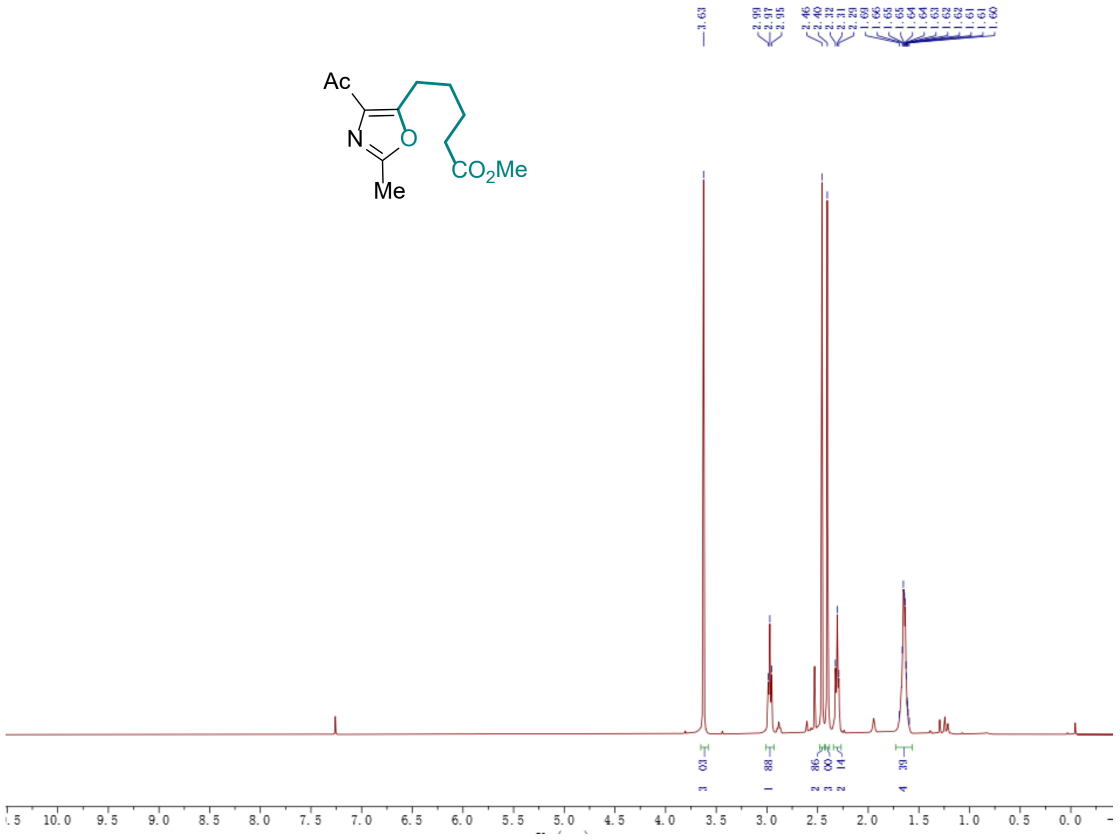

1H NMR of the **3ab** (400 MHz, 25 °C in CDCl3)

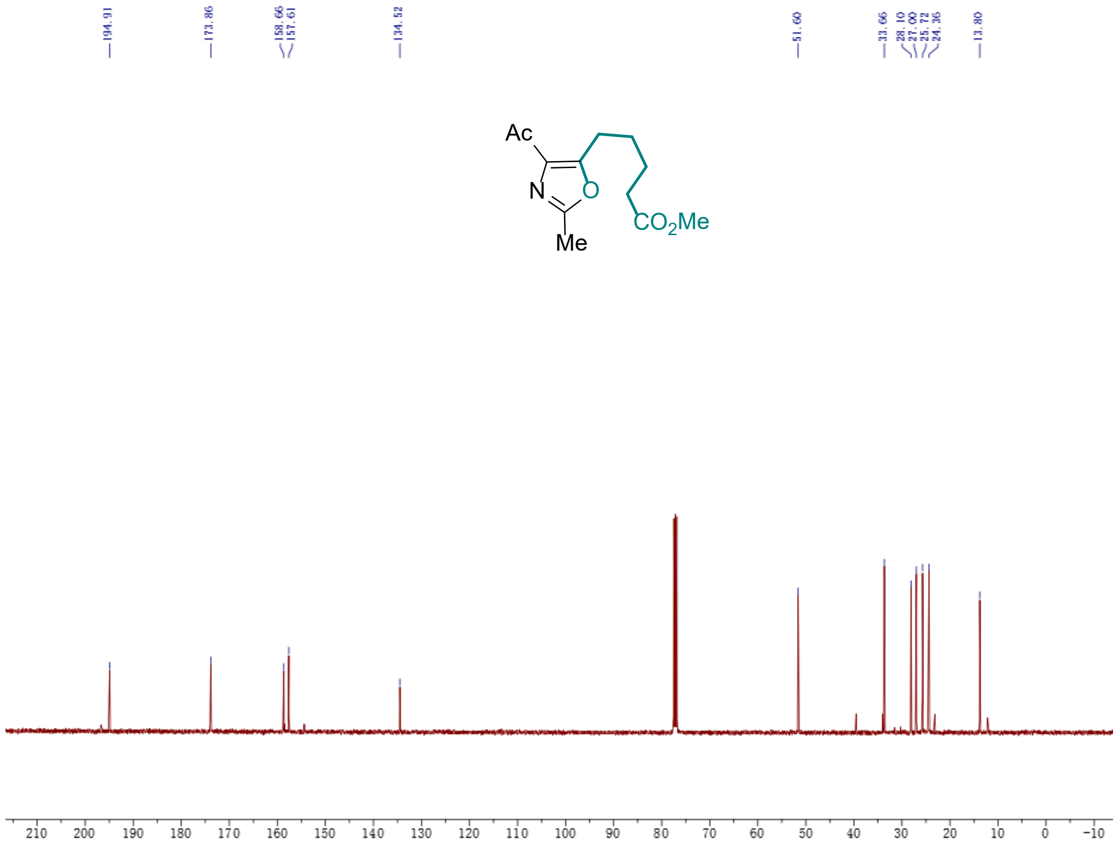

^{13}C NMR of the **3ab** (101 MHz, 25 °C in CDCl3)

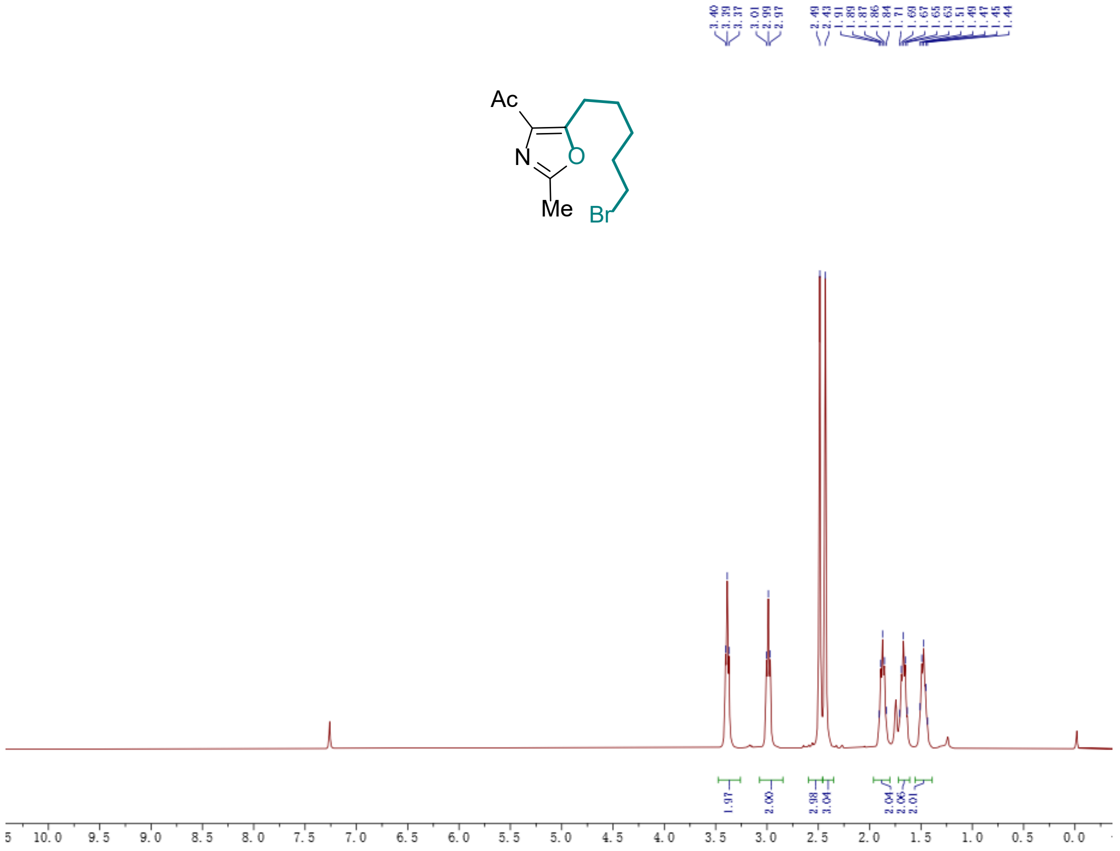

^{1}H NMR of the **3ac** (400 MHz, 25 °C in CDCl3)

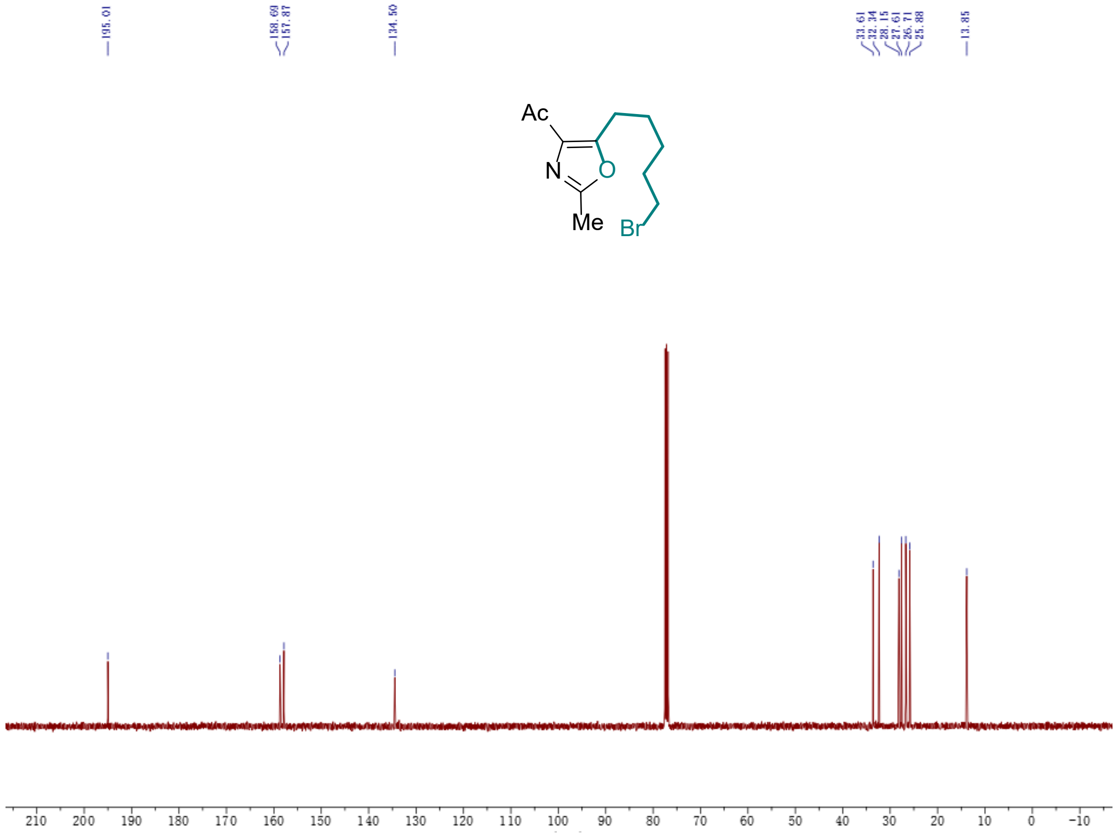

^{13}**C NMR** of the **3ac** (101 MHz, 25 °C in $CDCl_3$)

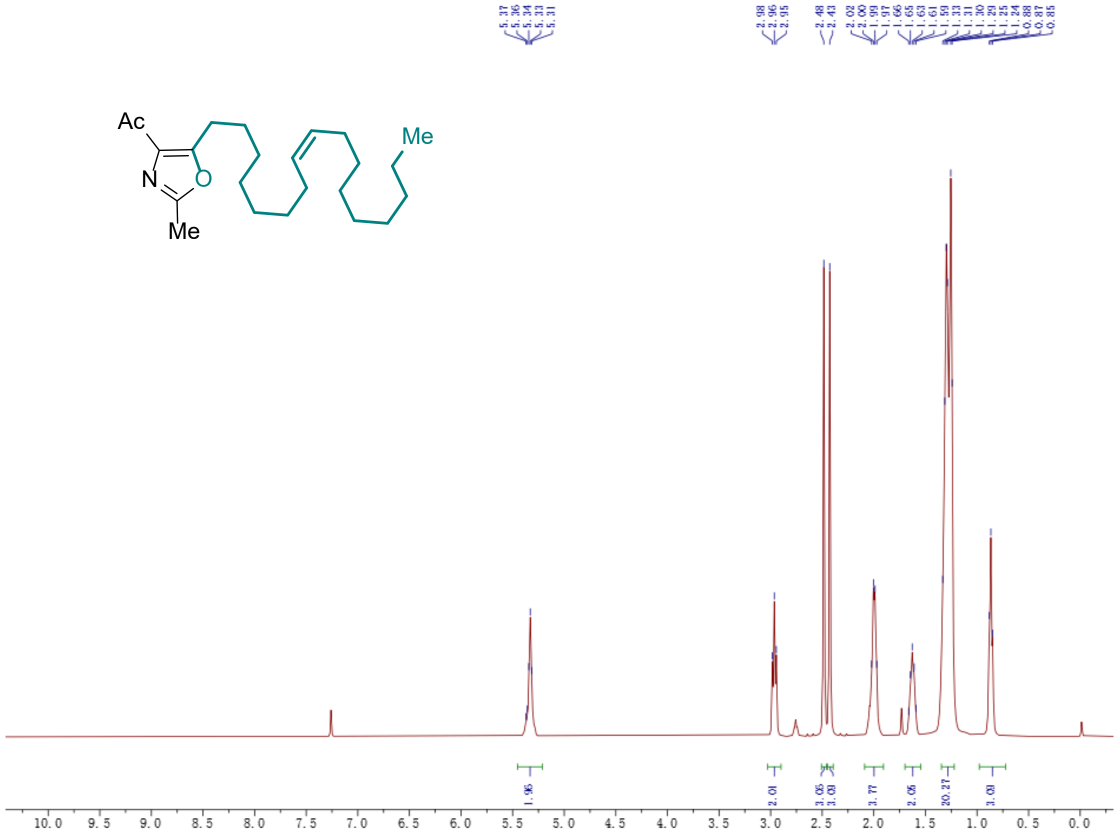

^{1}**H NMR** of the **3ad** (400 MHz, 25 °C in $CDCl_3$)

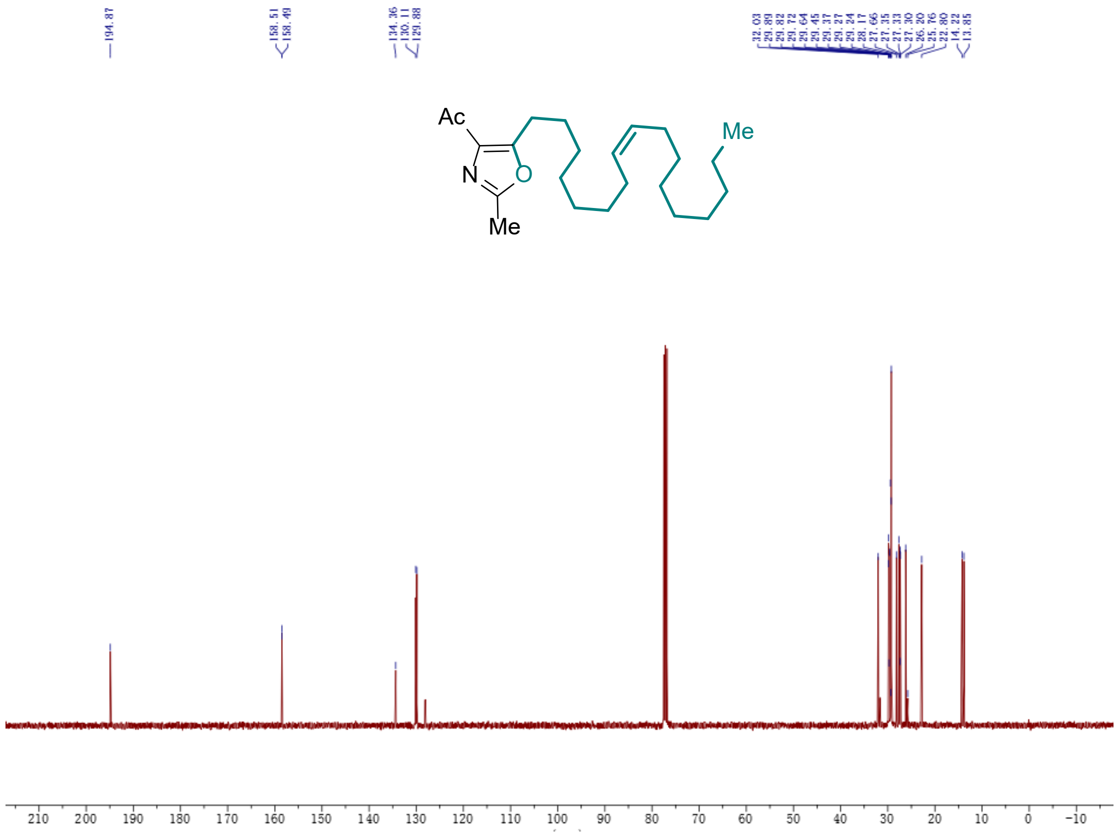

^{13}C NMR of the **3ad** (101 MHz, 25 °C in $CDCl_3$)

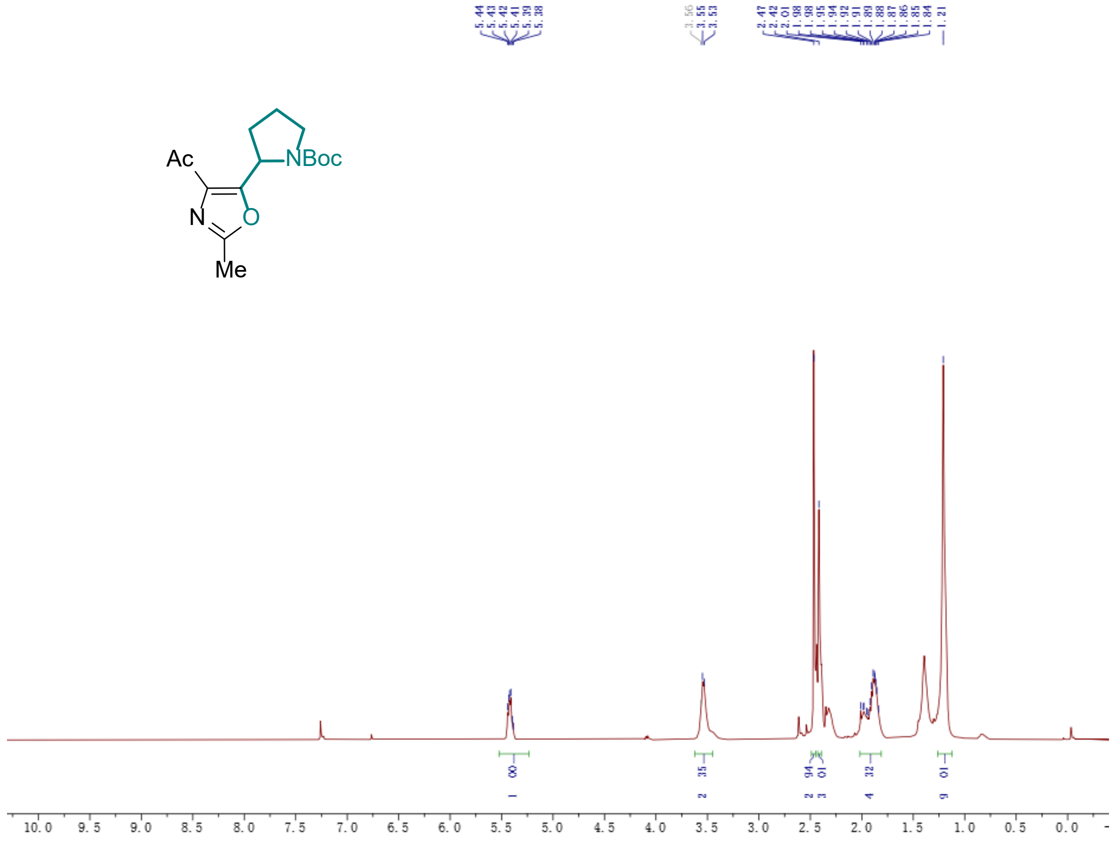

^{1}H NMR of the **3ae** (400 MHz, 25 °C in $CDCl_3$)

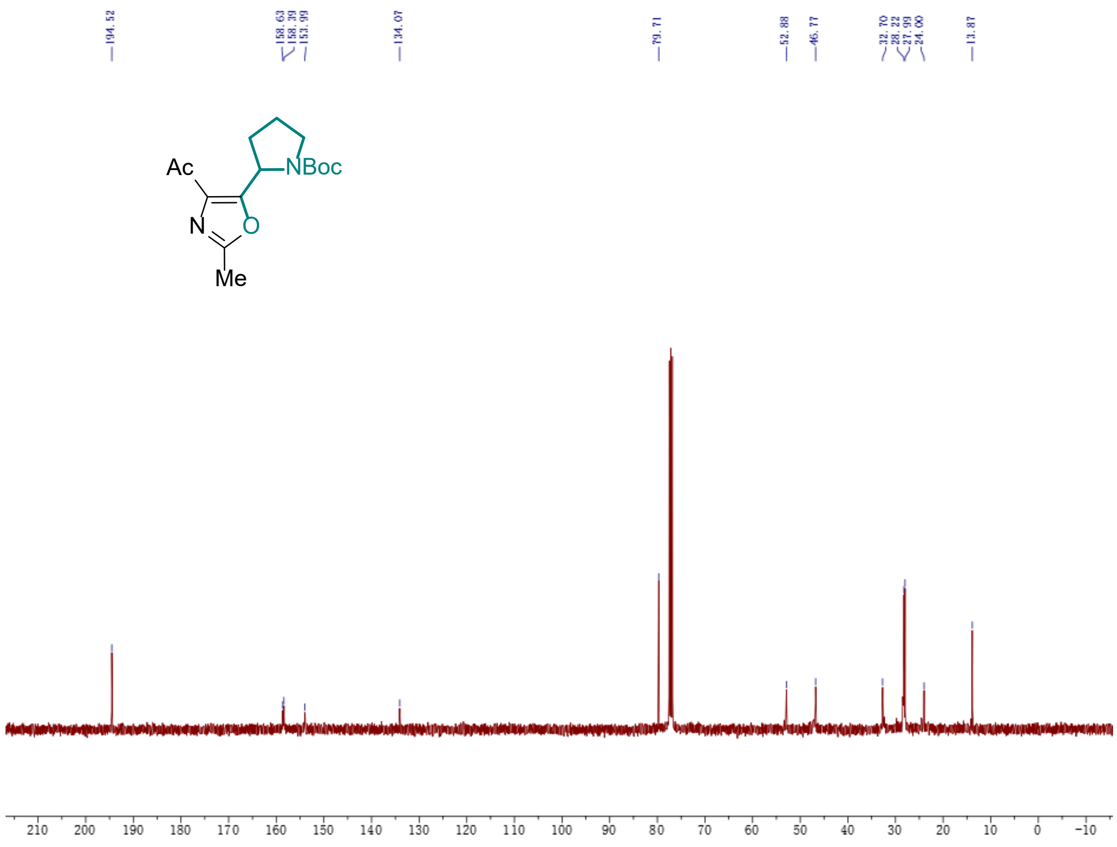

^{13}**C NMR** of the **3ae** (101 MHz, 25 °C in $CDCl_3$)

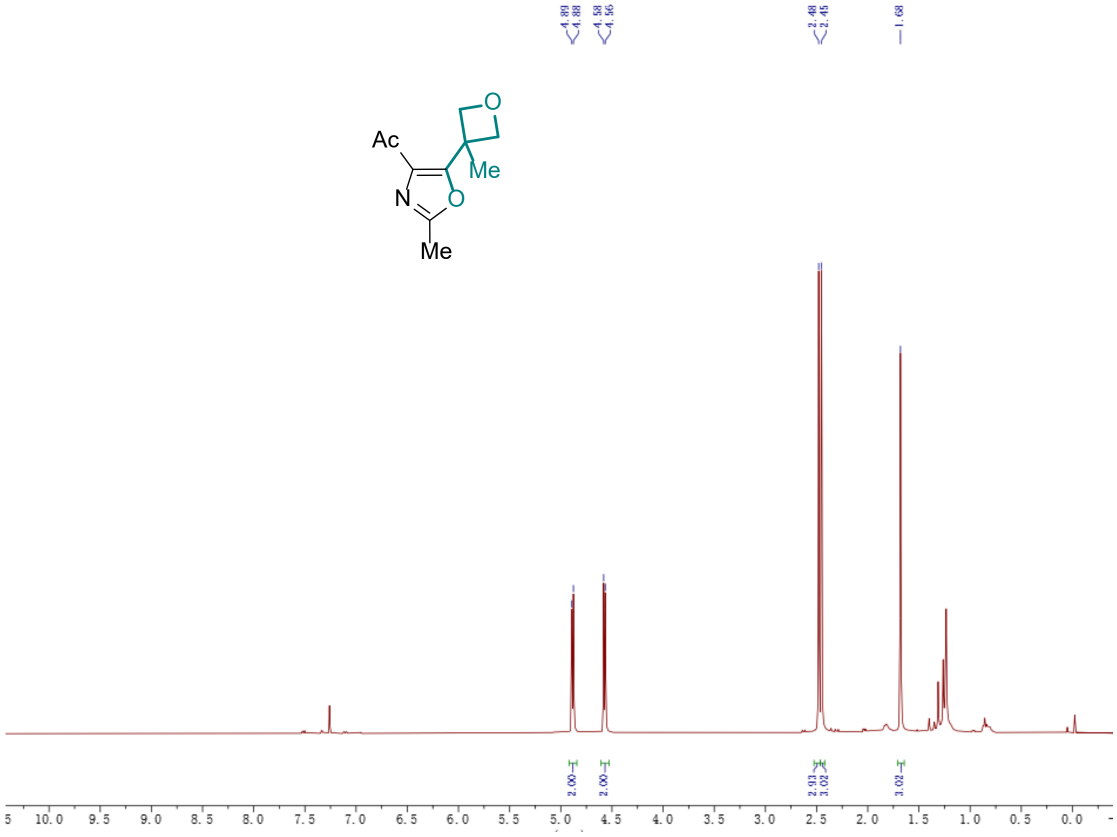

^{1}**H NMR** of the **3af** (400 MHz, 25 °C in $CDCl_3$)

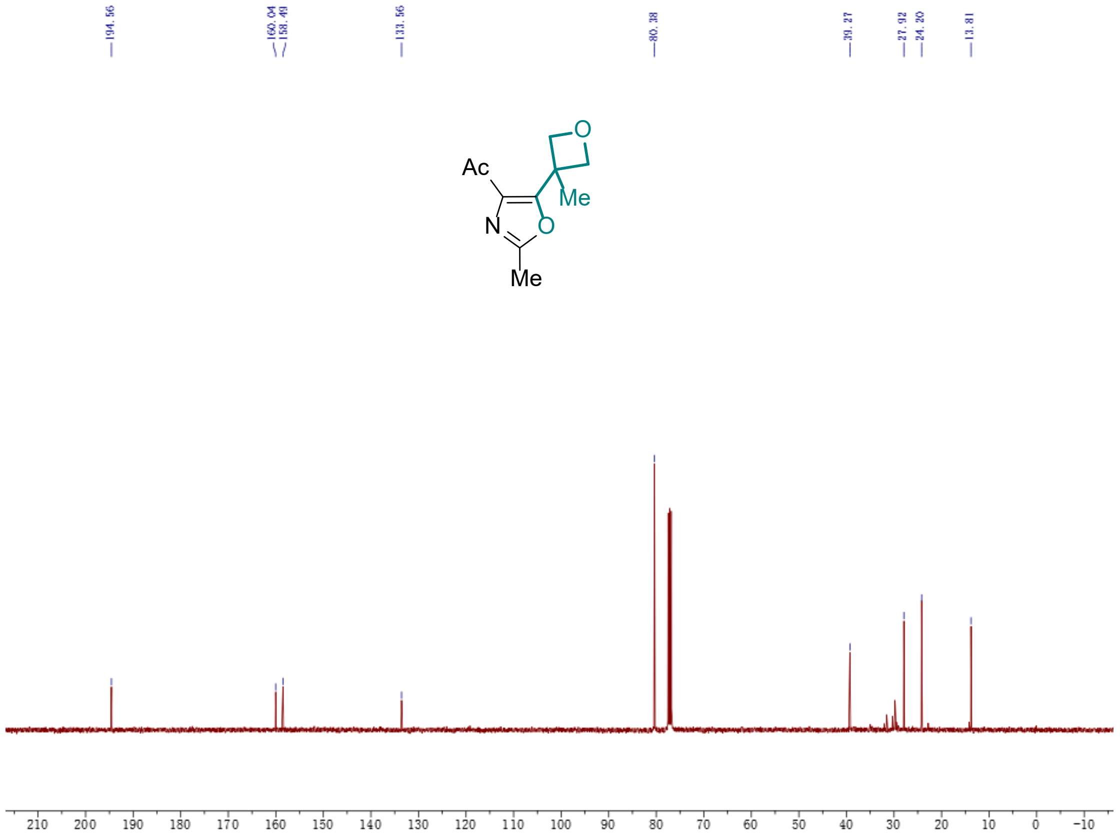

^{13}C NMR of the **3af** (101 MHz, 25 °C in $CDCl_3$)

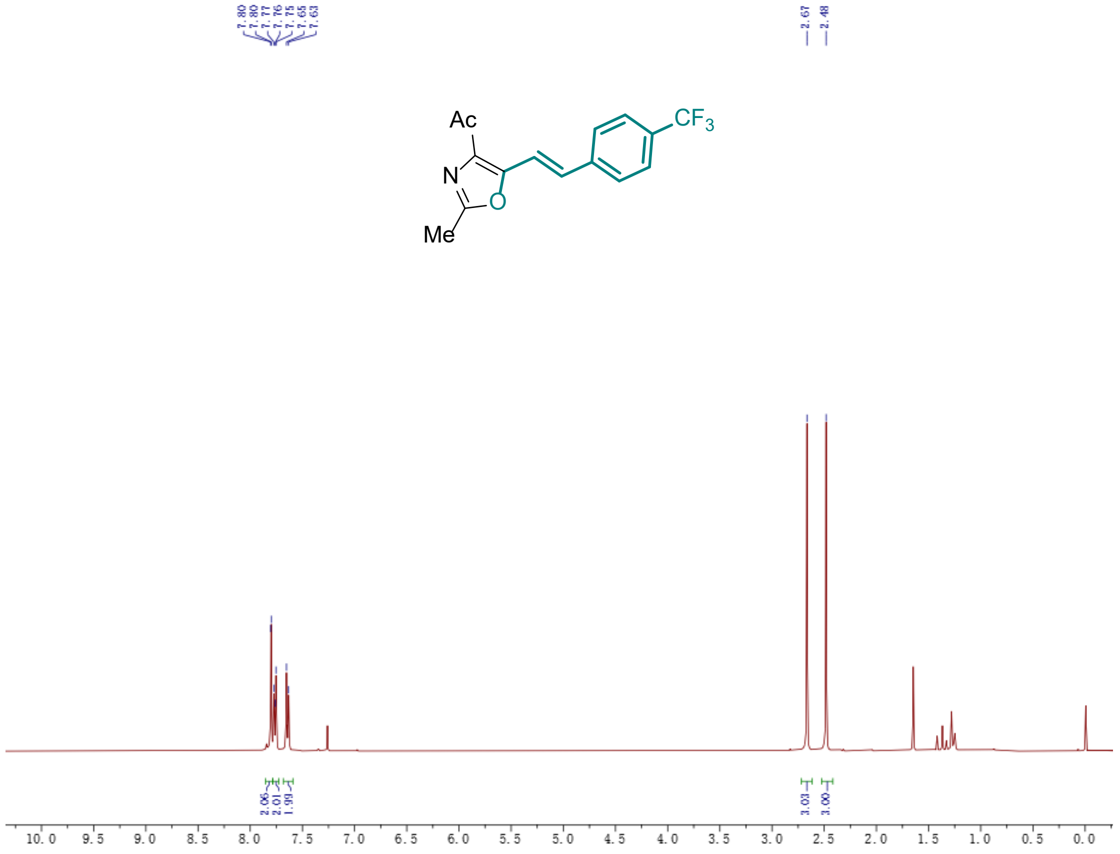

^{1}H NMR of the **3ag** (400 MHz, 25 °C in $CDCl_3$)

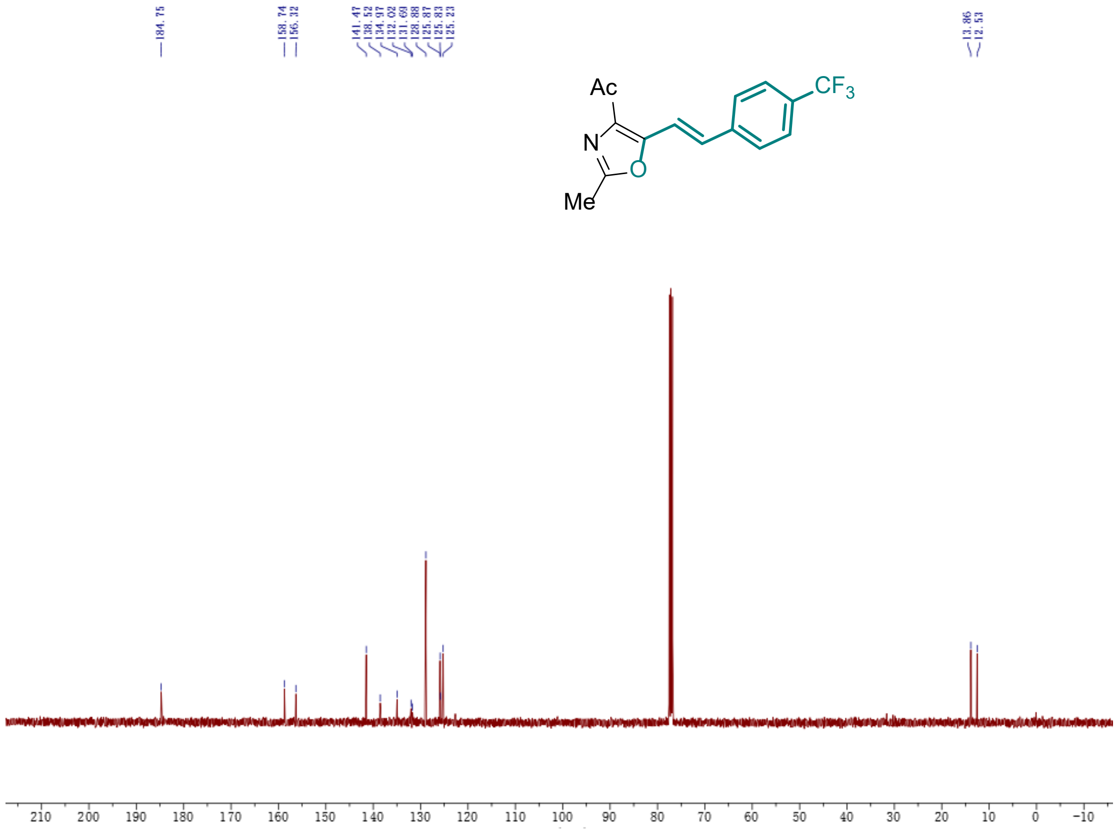

^{13}C NMR of the **3ag** (101 MHz, 25 °C in $CDCl_3$)

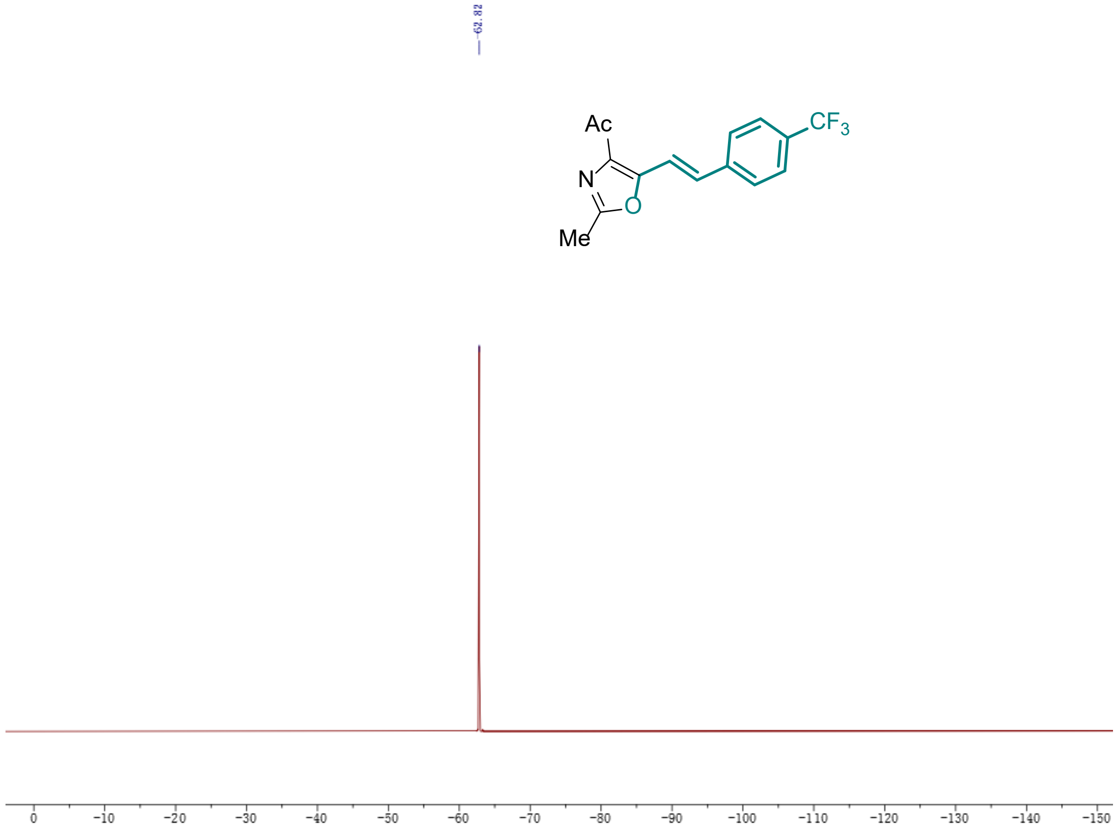

^{19}F NMR of the **3ag** (376 MHz, 25 °C in $CDCl_3$)

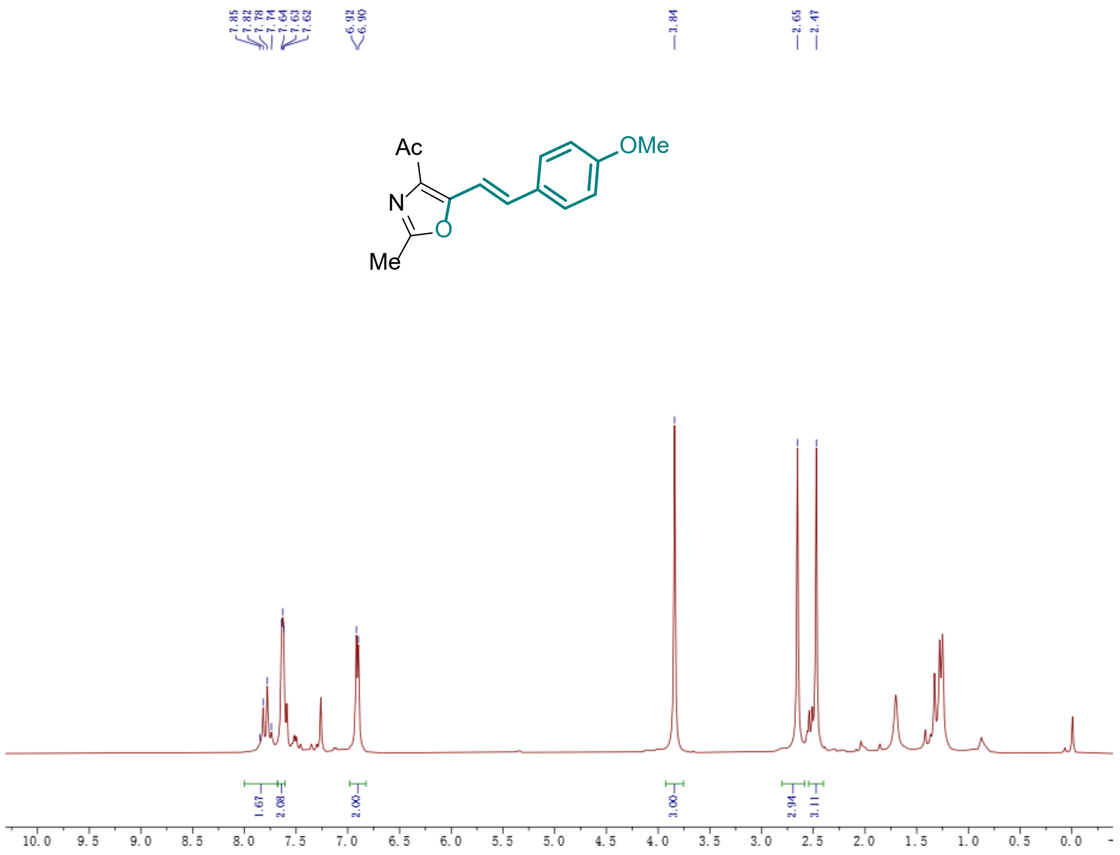

^{1}H NMR of the **3ah** (400 MHz, 25 °C in $CDCl_3$)

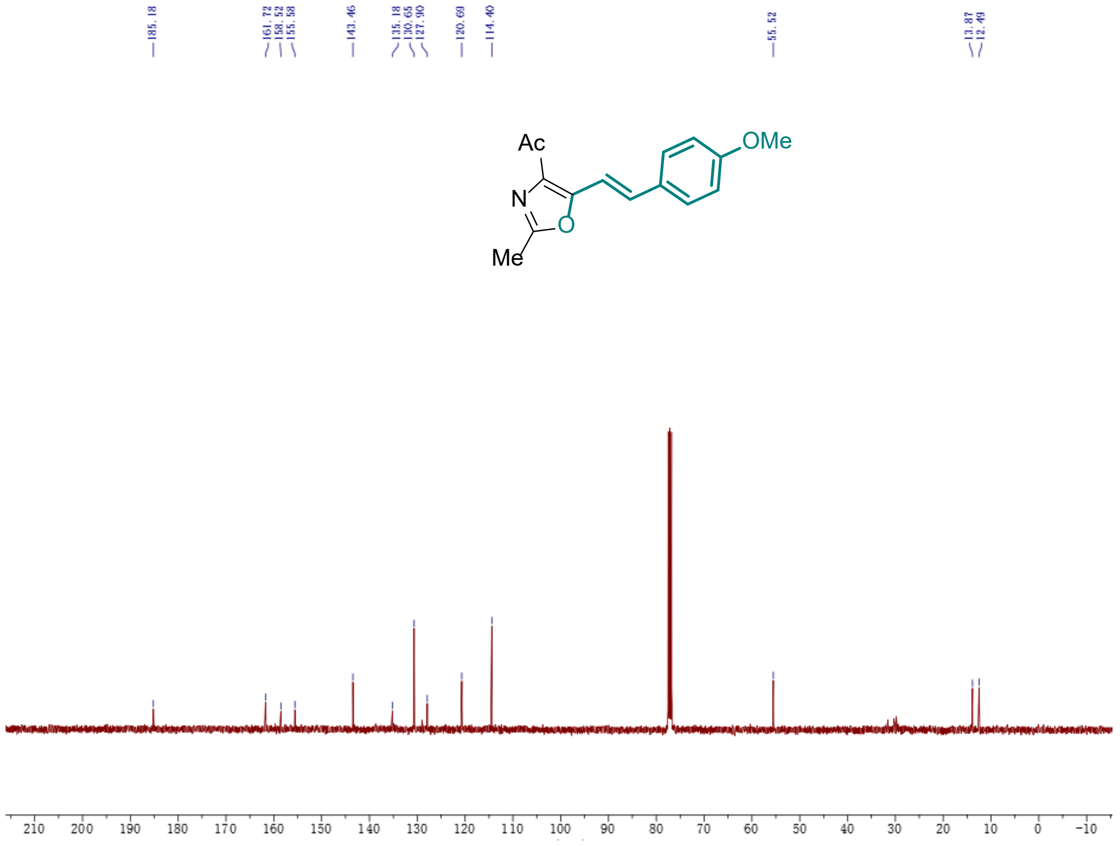

^{13}C NMR of the **3ah** (101 MHz, 25 °C in $CDCl_3$)

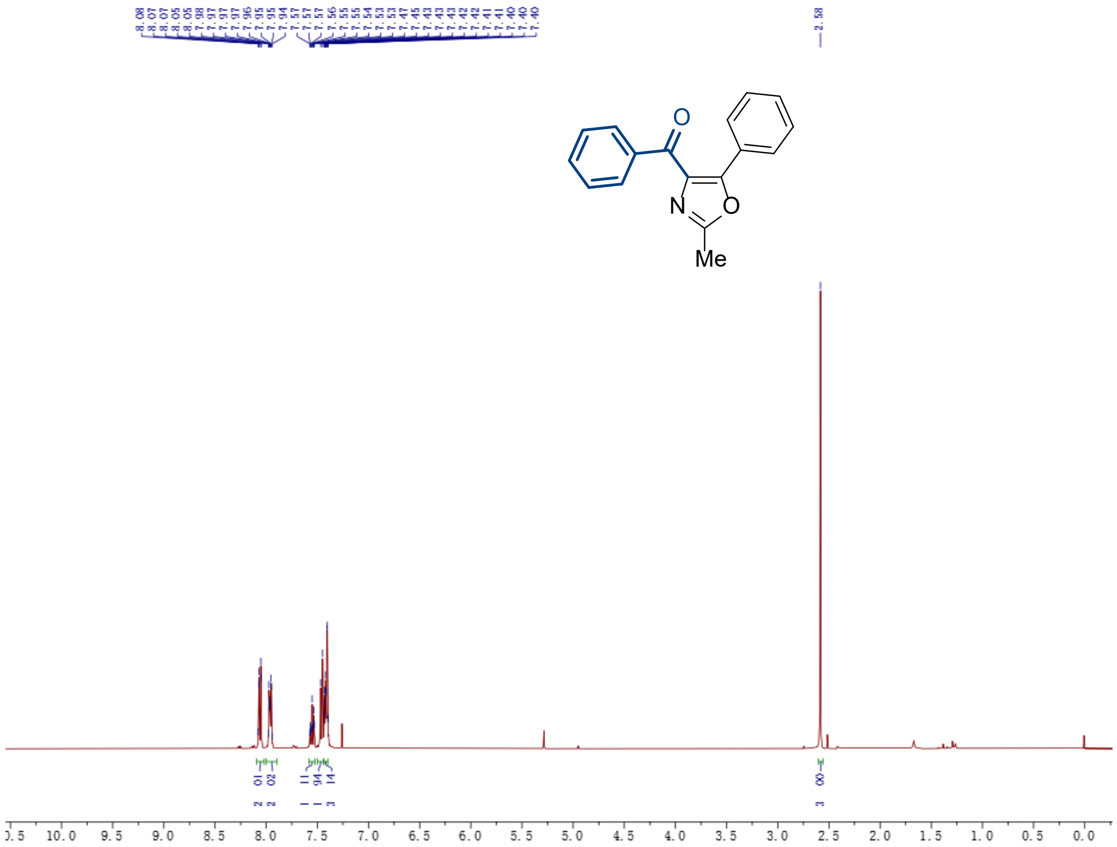

^{1}H **NMR** of the **3ai** (400 MHz, 25 °C in $CDCl_3$)

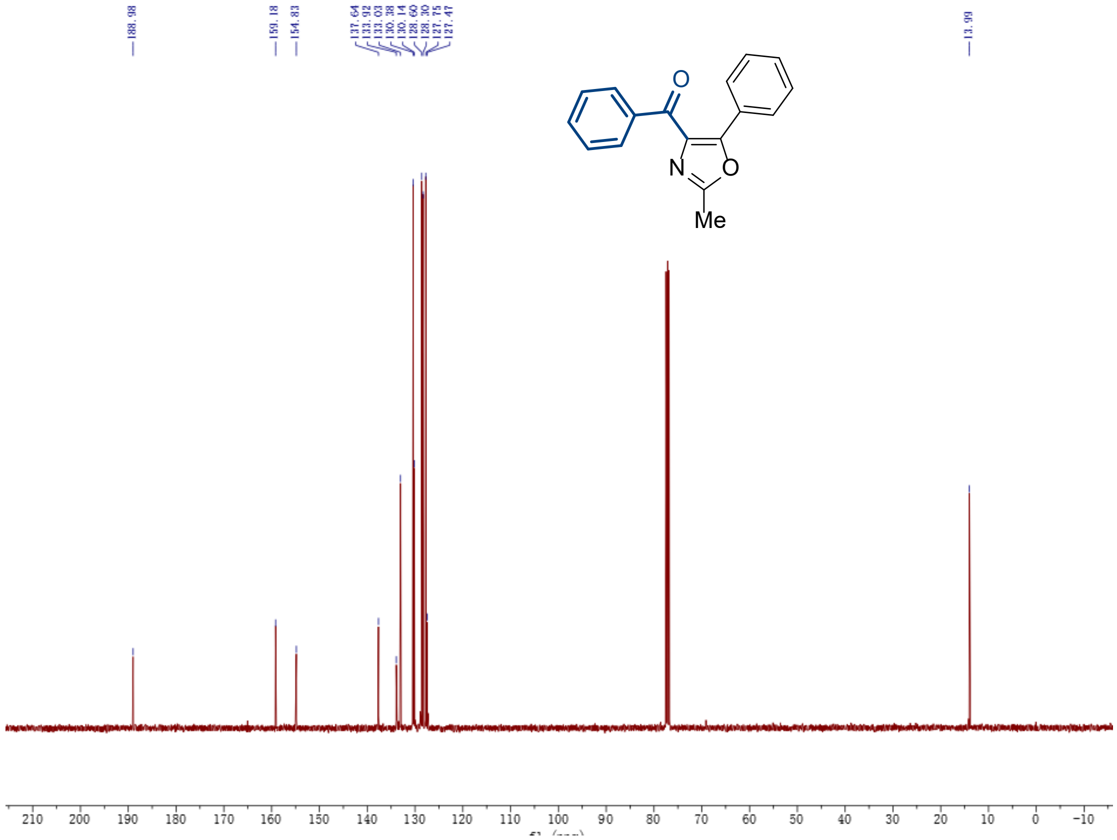

^{13}C **NMR** of the **3ai** (101 MHz, 25 °C in $CDCl_3$)

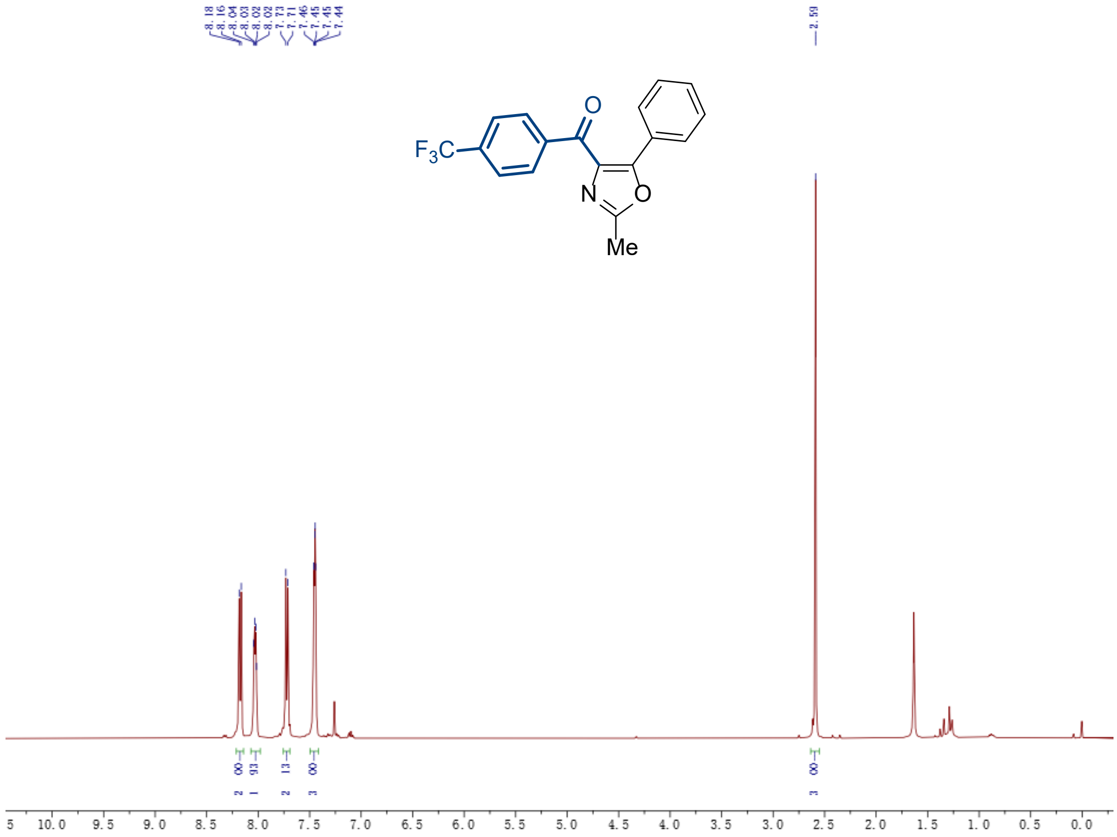

^{1}**H NMR** of the **3aj** (400 MHz, 25 °C in $CDCl_3$)

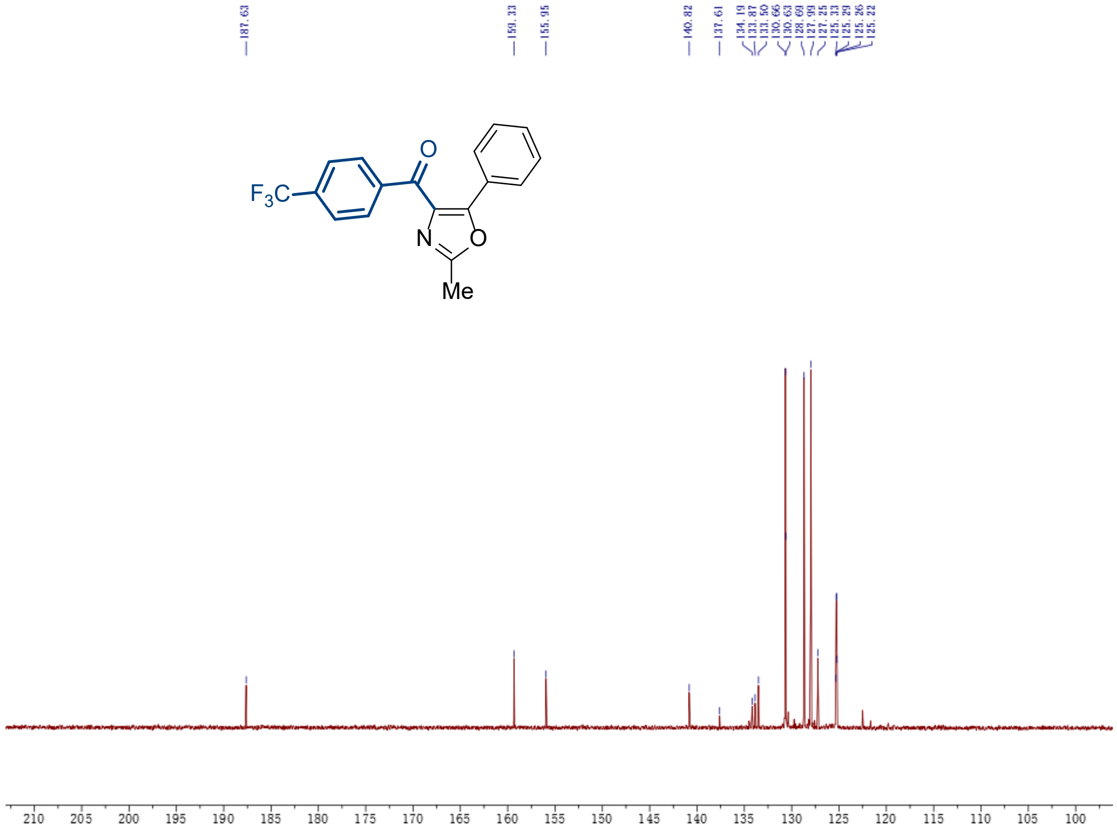

^{13}**C NMR** of the **3aj** (101 MHz, 25 °C in $CDCl_3$)

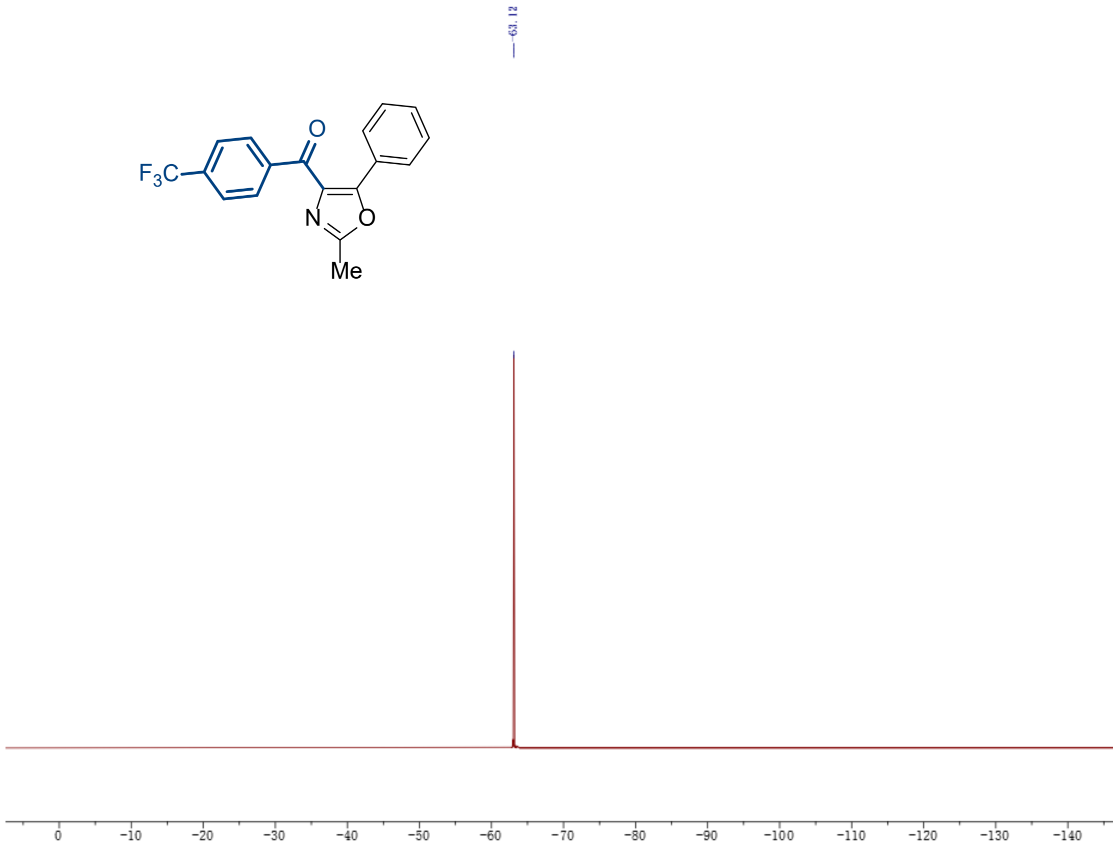

^{19}F NMR of the **3aj** (376 MHz, 25 °C in $CDCl_3$)

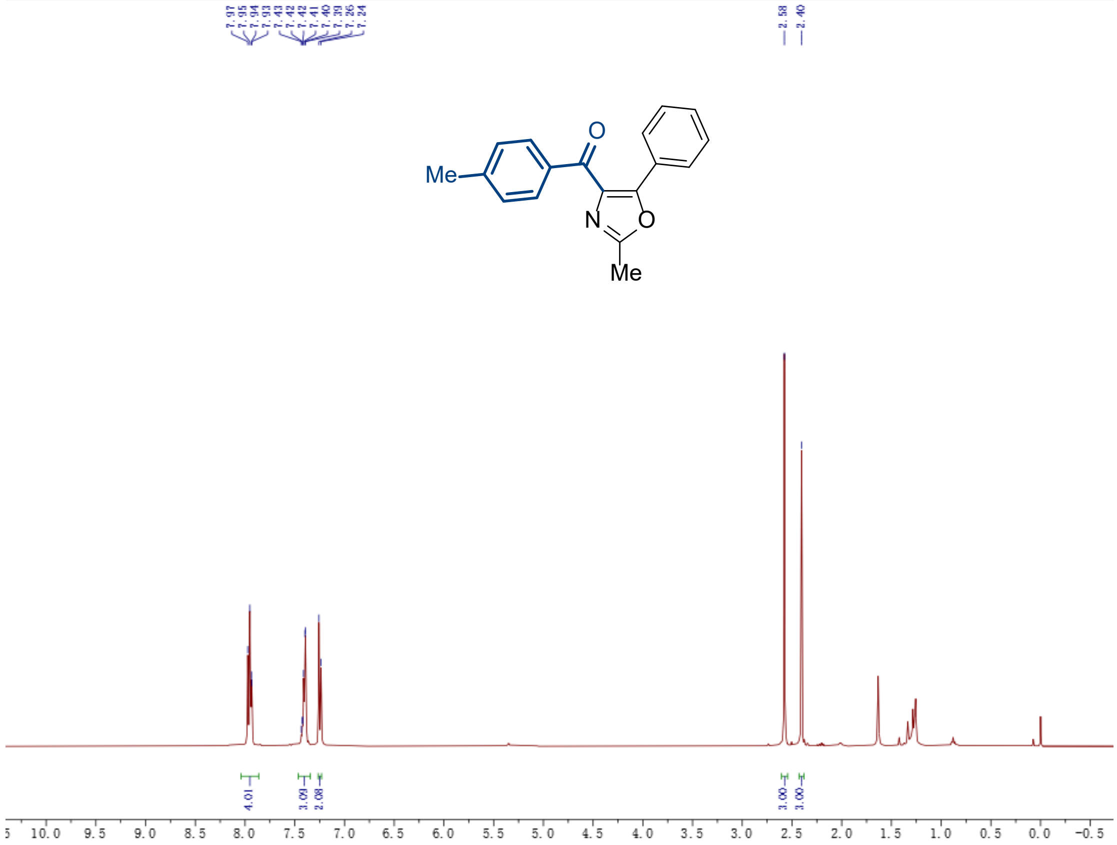

^{1}H NMR of the **3ak** (400 MHz, 25 °C in $CDCl_3$)

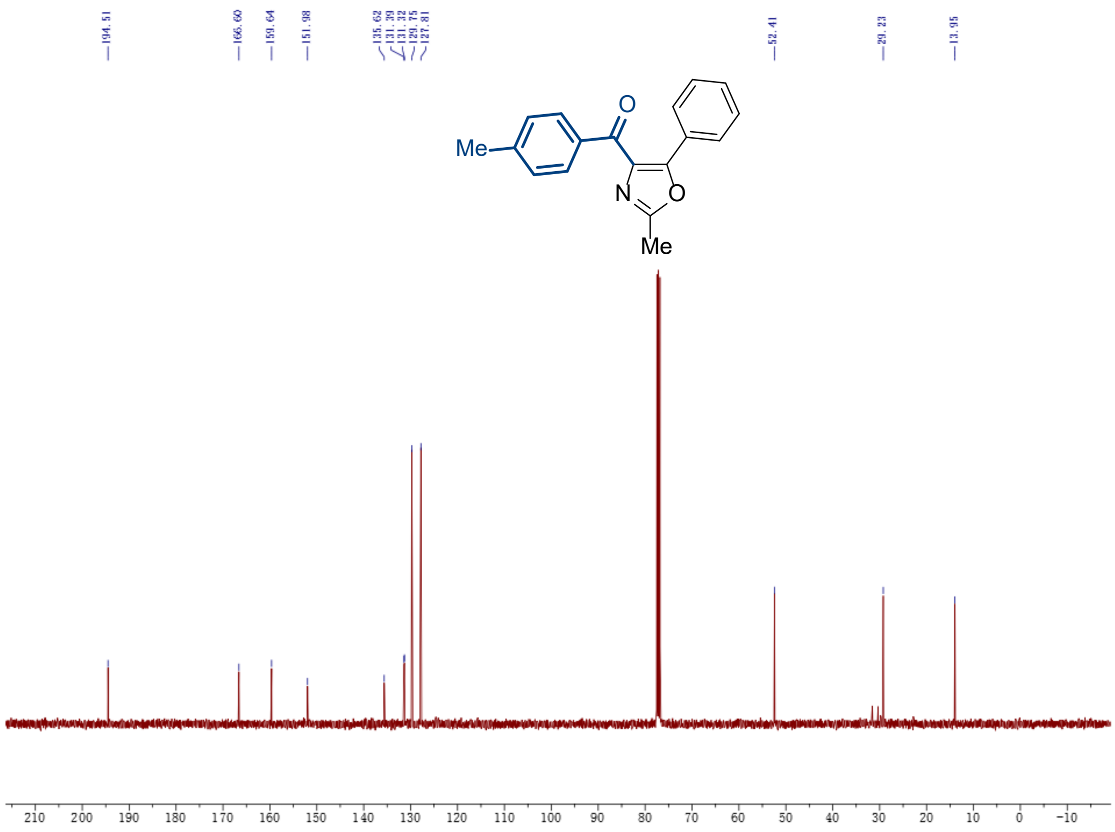

^{13}C NMR of the **3ak** (101 MHz, 25 °C in $CDCl_3$)

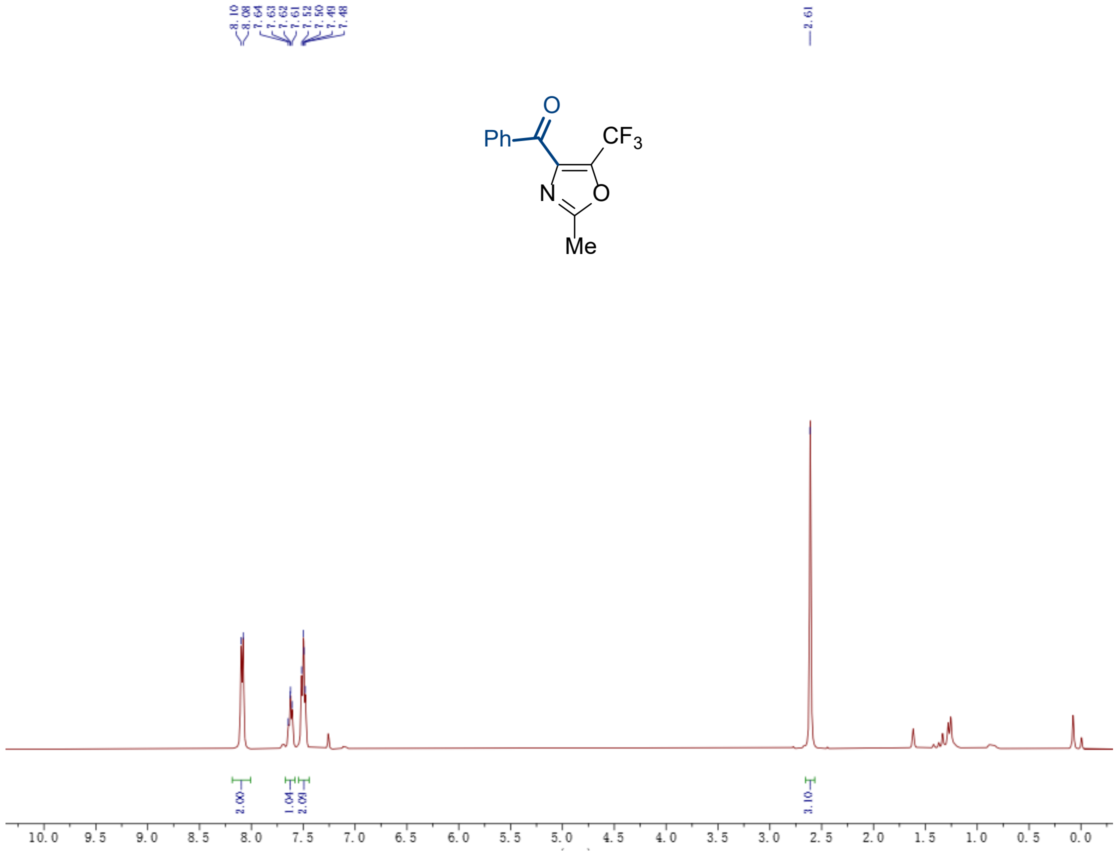

^{1}H NMR of the **3al** (400 MHz, 25 °C in $CDCl_3$)

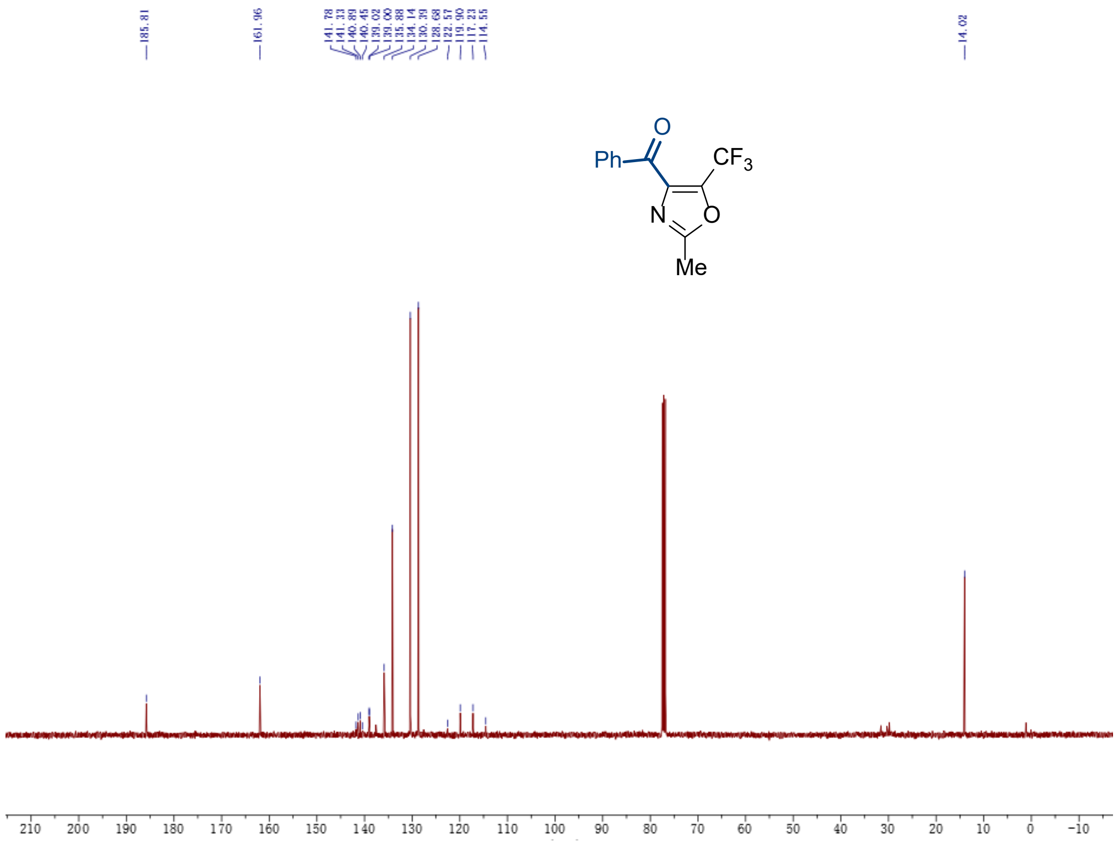

^{13}**C NMR** of the **3al** (101 MHz, 25 °C in $CDCl_3$)

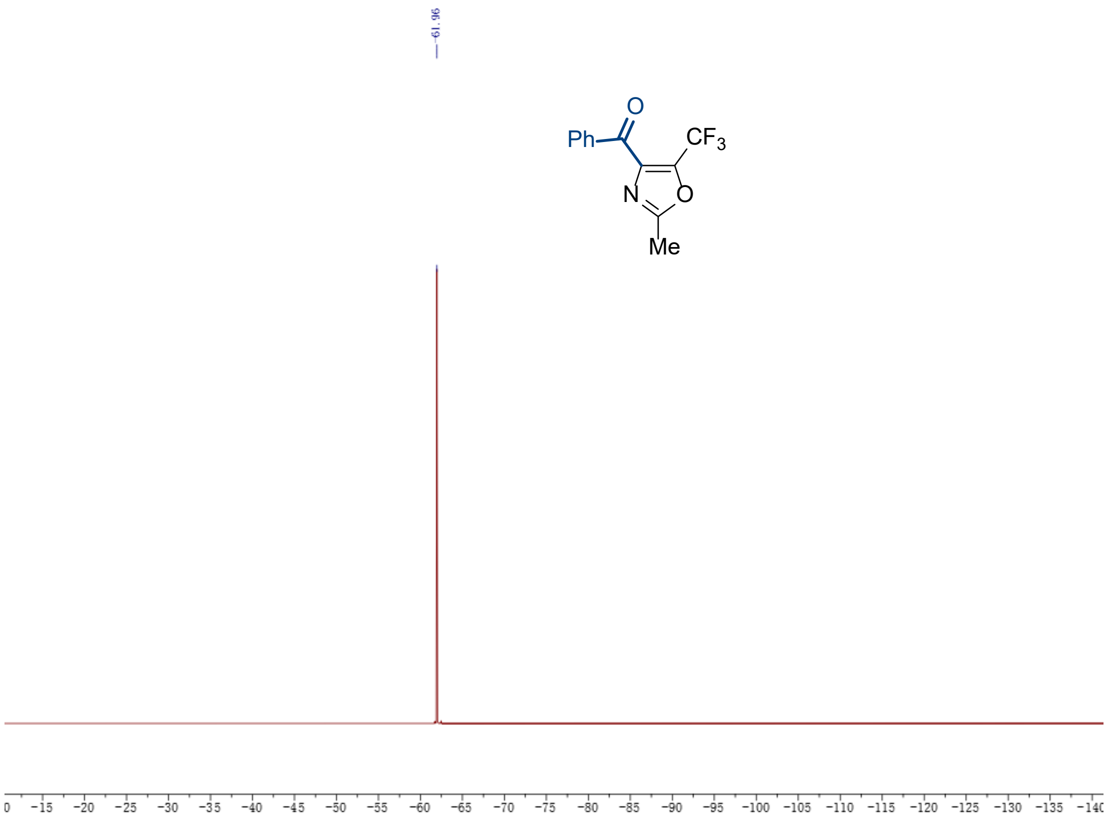

^{19}**F NMR** of the **3al** (376 MHz, 25 °C in $CDCl_3$)

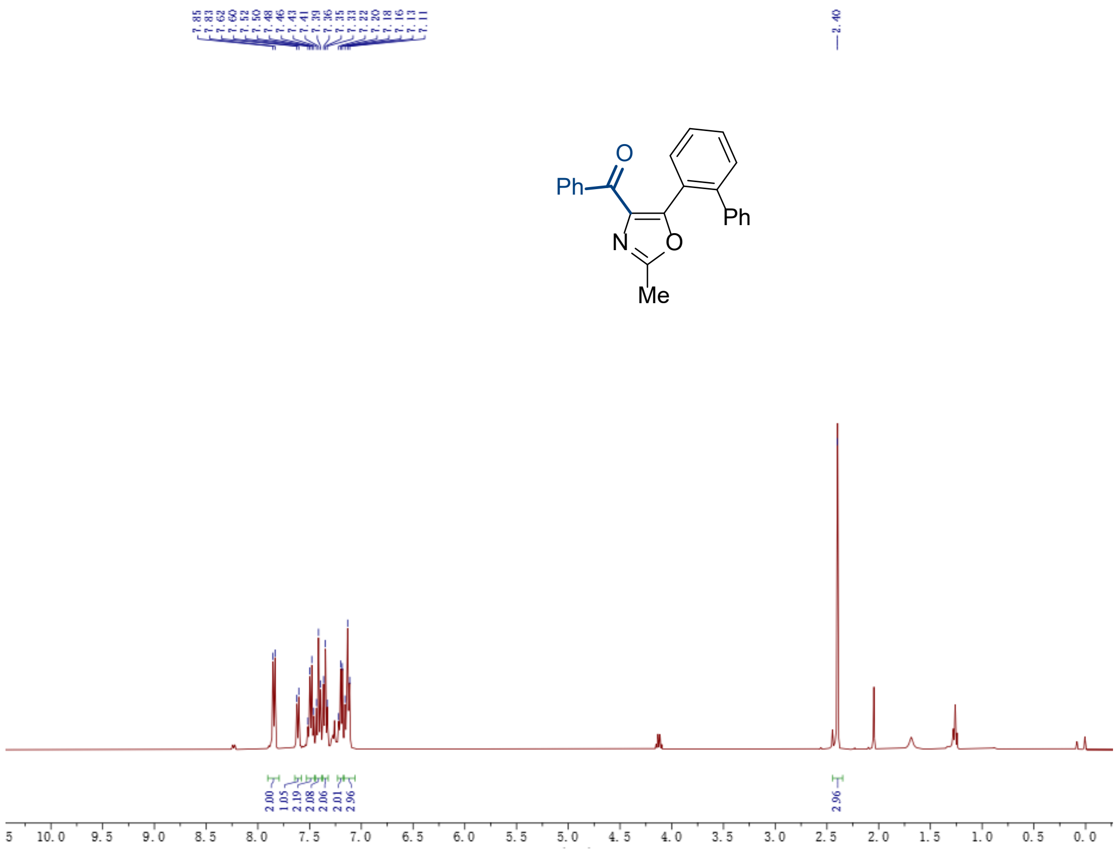

^{1}**H NMR** of the **3am** (400 MHz, 25 °C in $CDCl_3$)

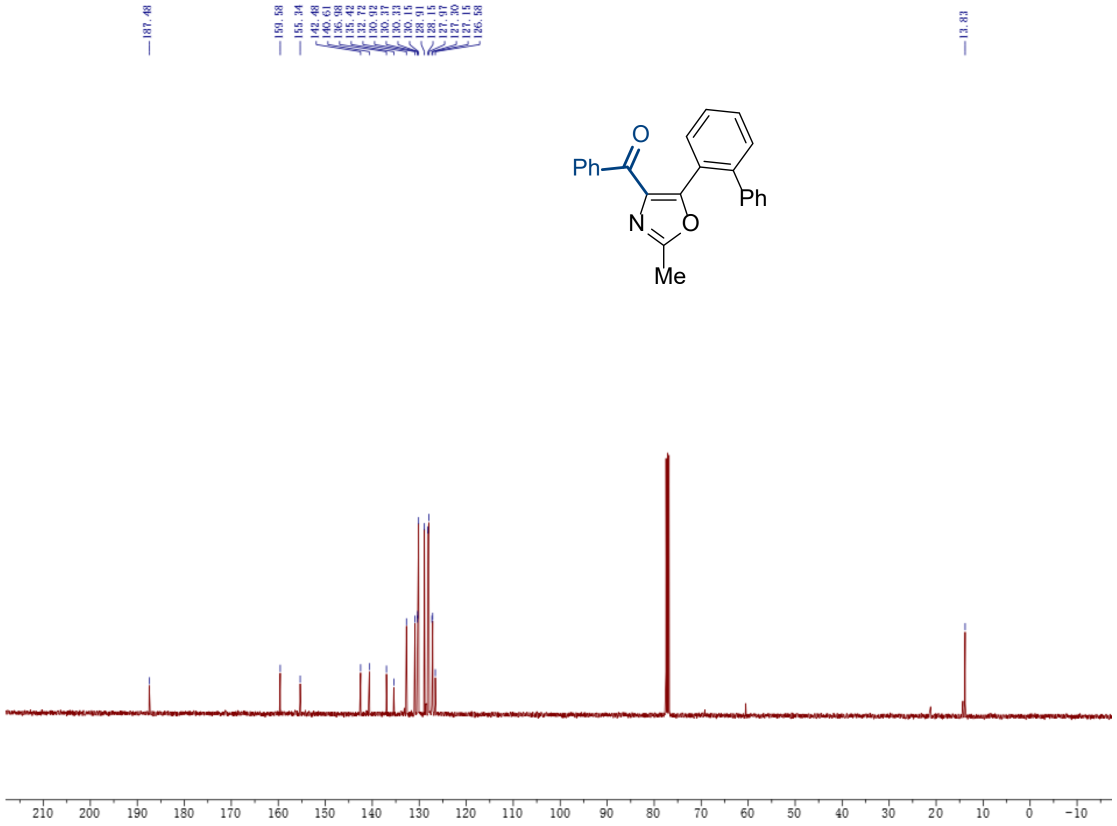

^{13}**C NMR** of the **3am** (101 MHz, 25 °C in $CDCl_3$)

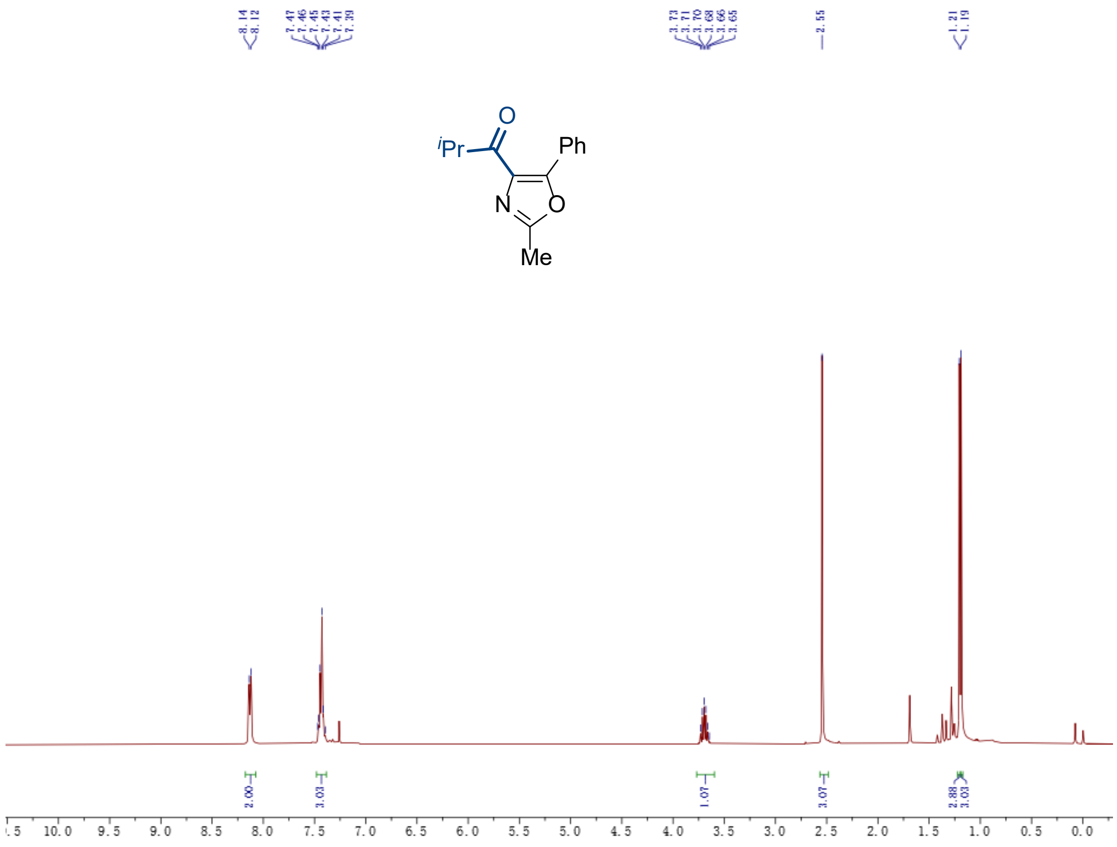

^{1}H NMR of the **3an** (400 MHz, 25 °C in $CDCl_3$)

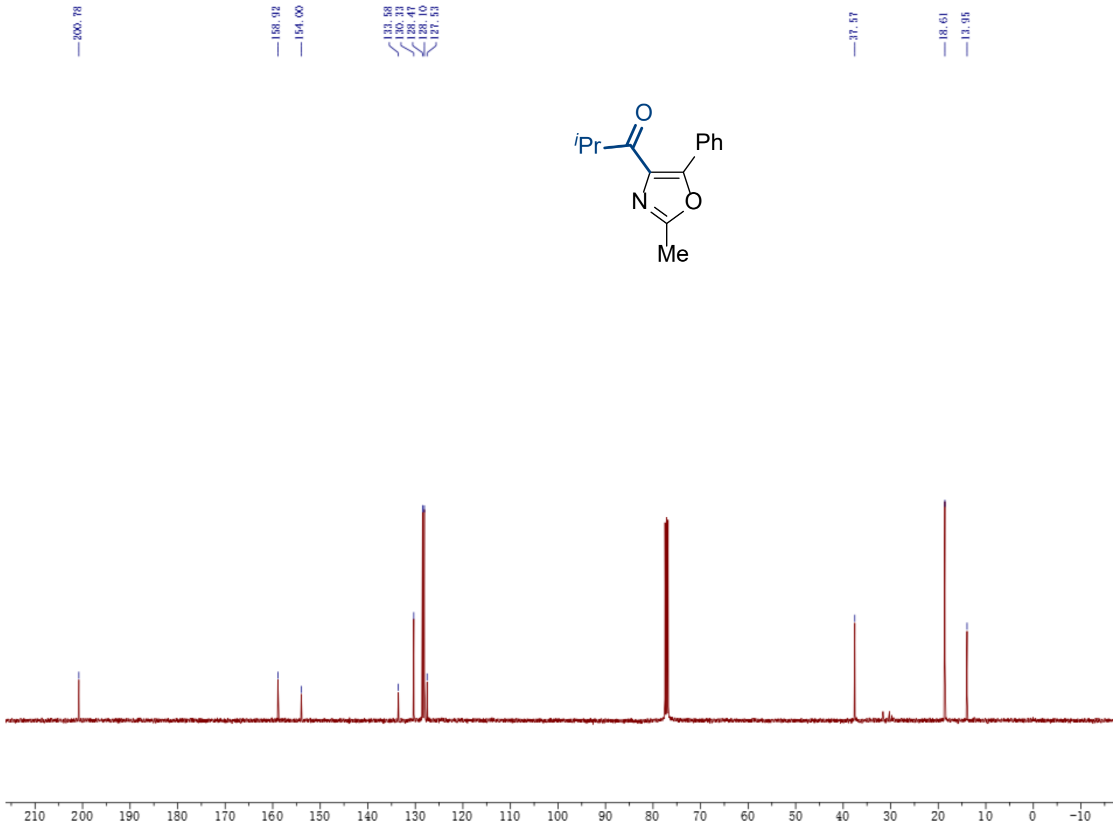

^{13}C NMR of the **3an** (101 MHz, 25 °C in $CDCl_3$)

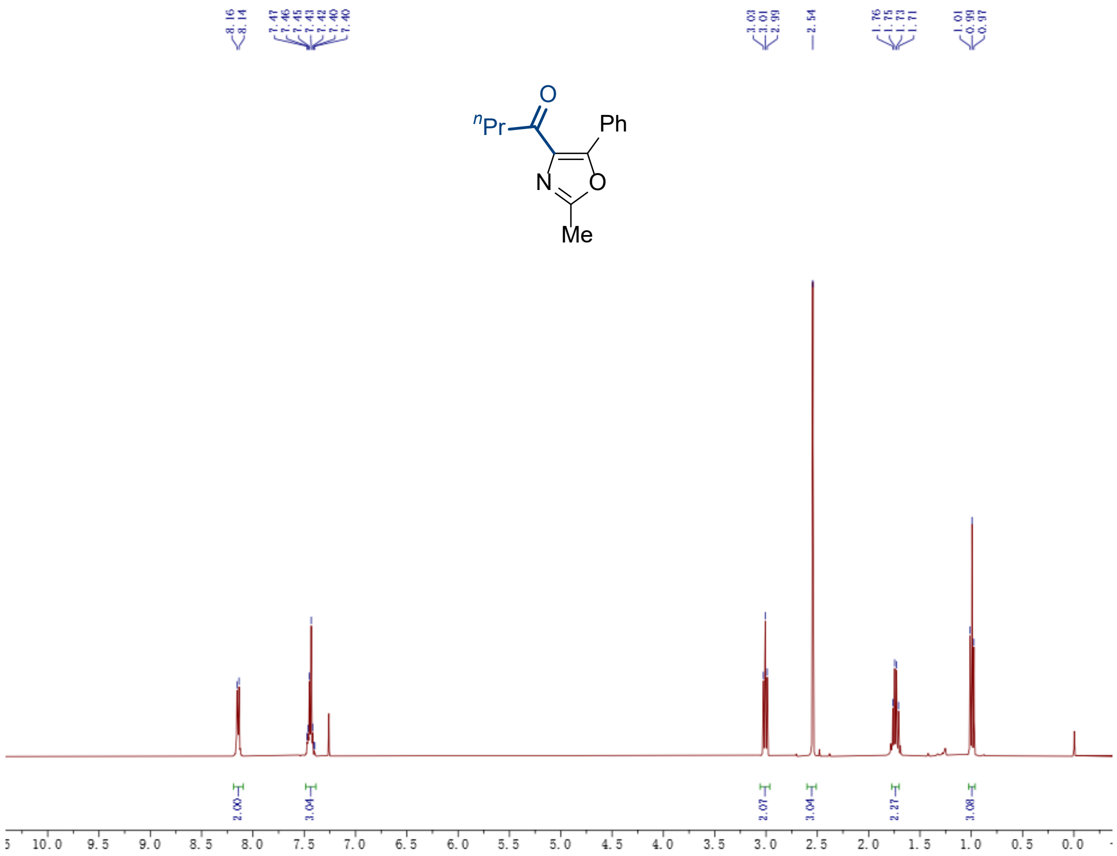

^{1}H NMR of the **3ao** (400 MHz, 25 °C in $CDCl_3$)

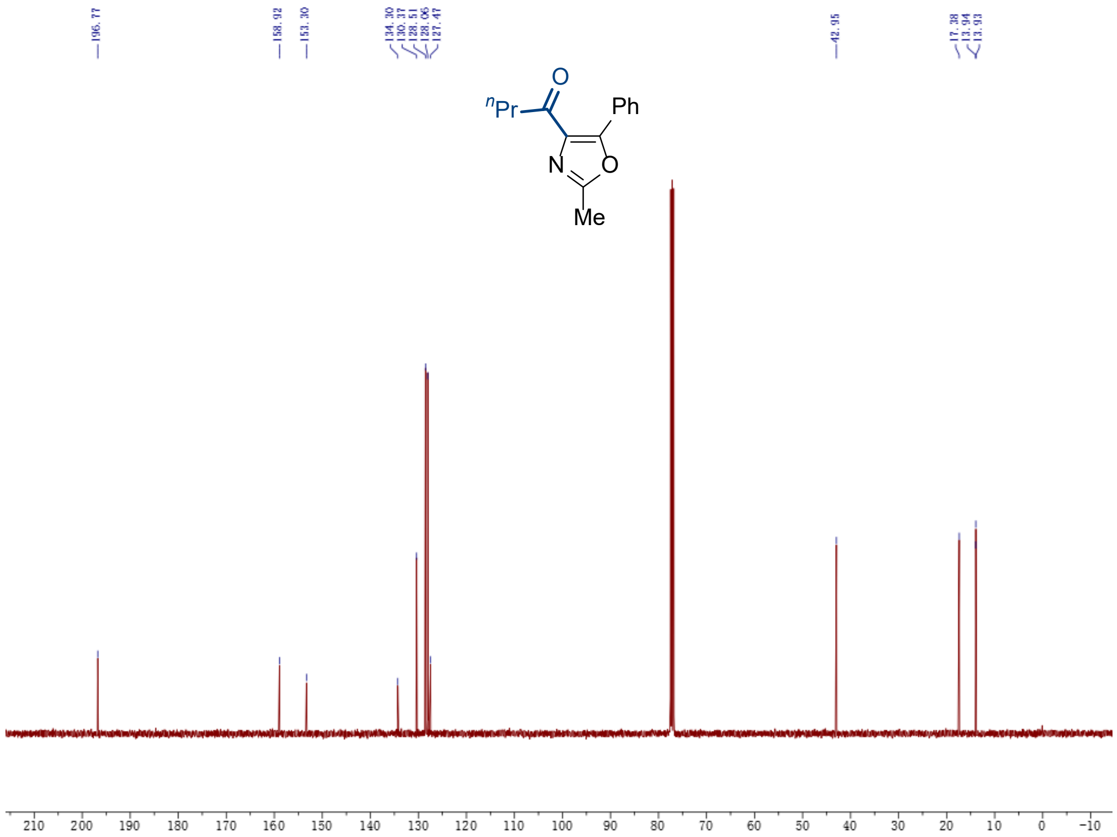

^{13}C NMR of the **3ao** (101 MHz, 25 °C in $CDCl_3$)

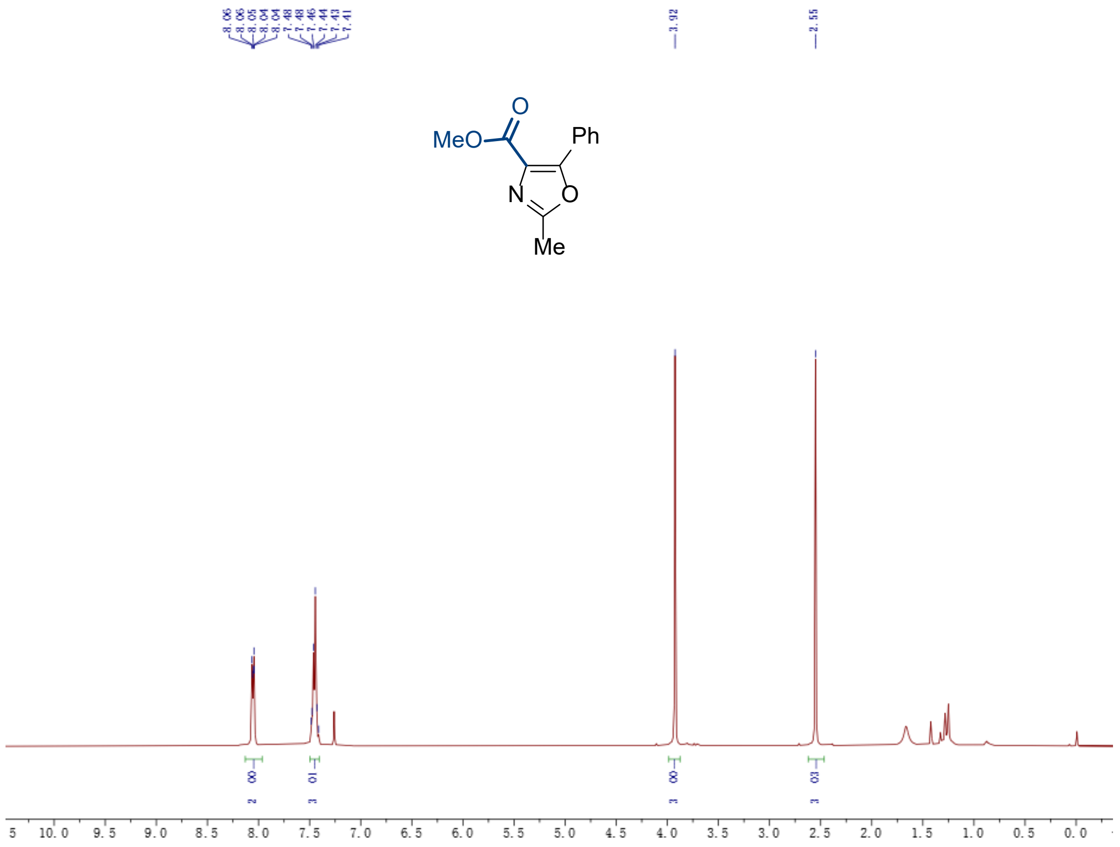

^{1}H NMR of the **3ap** (400 MHz, 25 °C in $CDCl_3$)

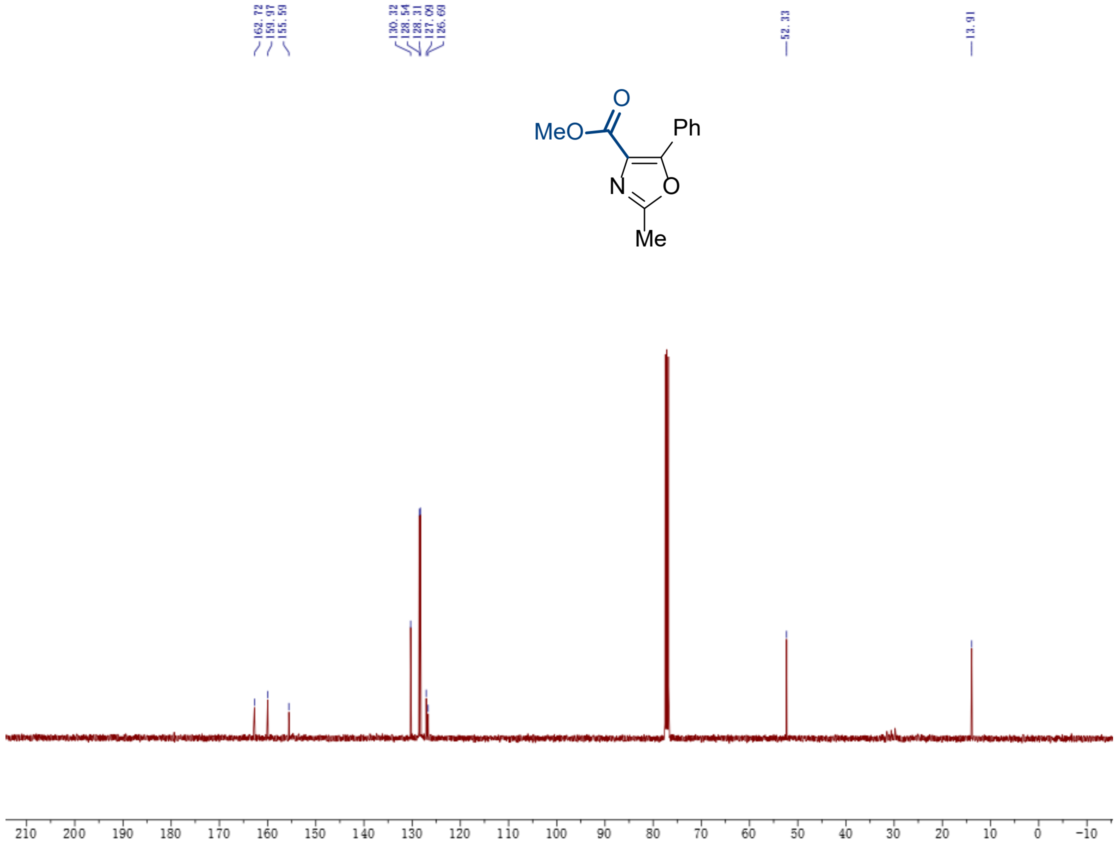

^{13}C NMR of the **3ap** (101 MHz, 25 °C in $CDCl_3$)

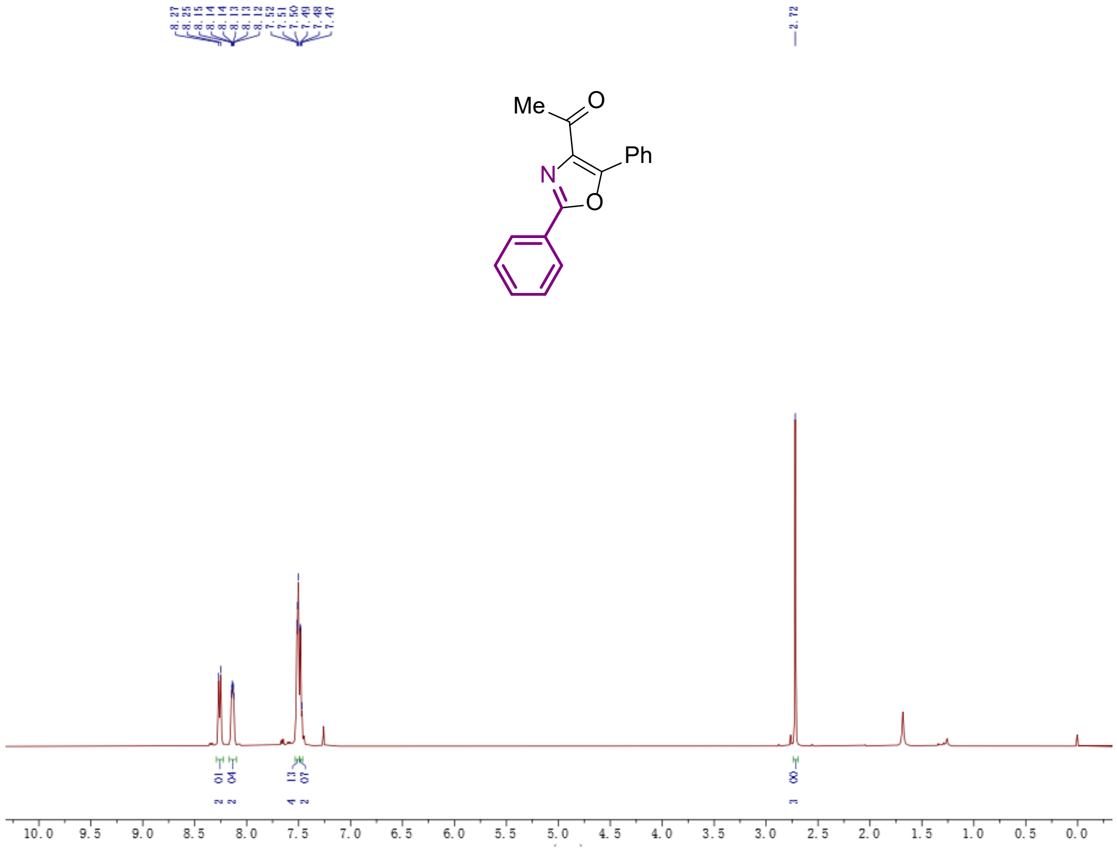

^{1}H NMR of the **3aq** (400 MHz, 25 °C in $CDCl_3$)

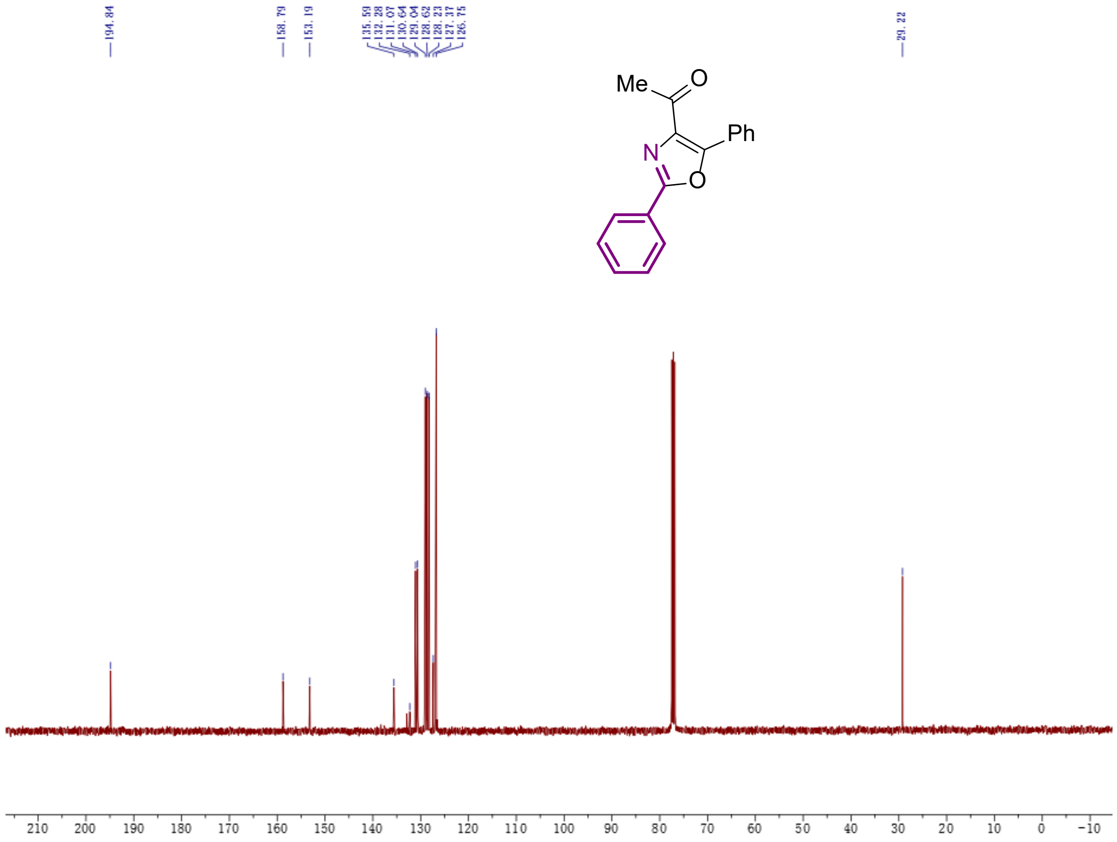

^{13}C NMR of the **3aq** (101 MHz, 25 °C in $CDCl_3$)

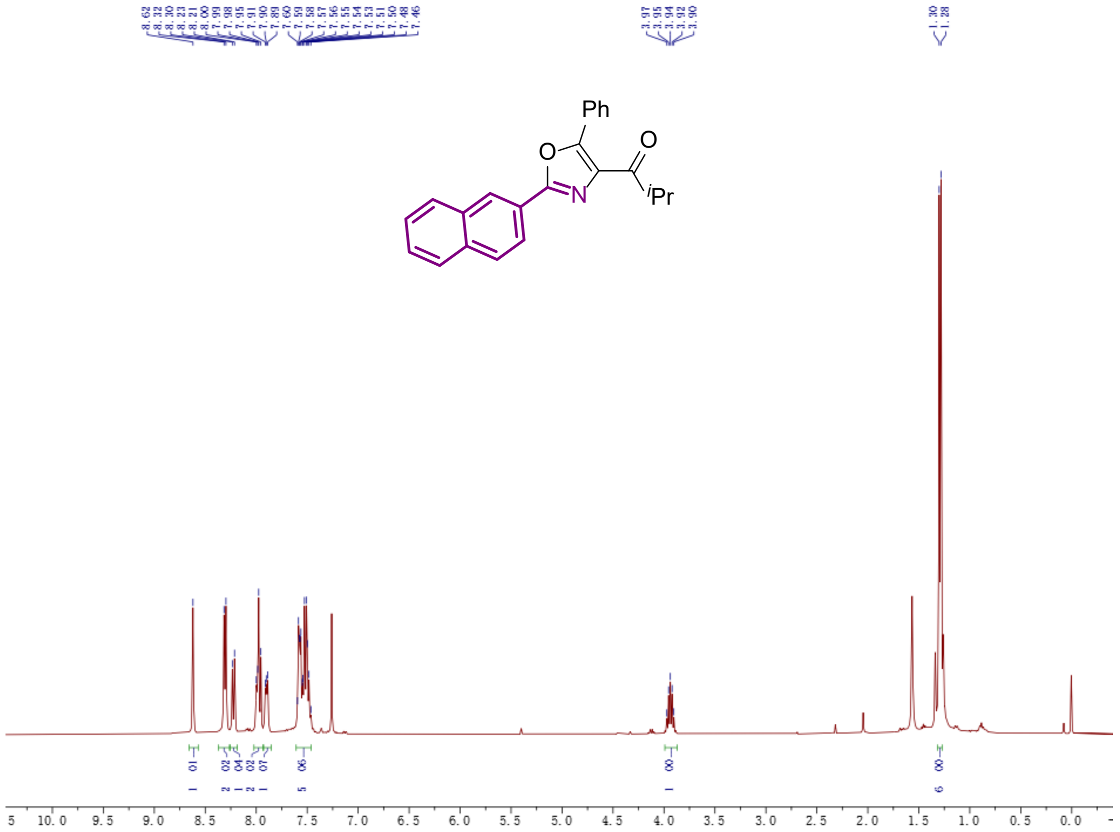

^{1}H NMR of the **3ar** (400 MHz, 25 °C in $CDCl_3$)

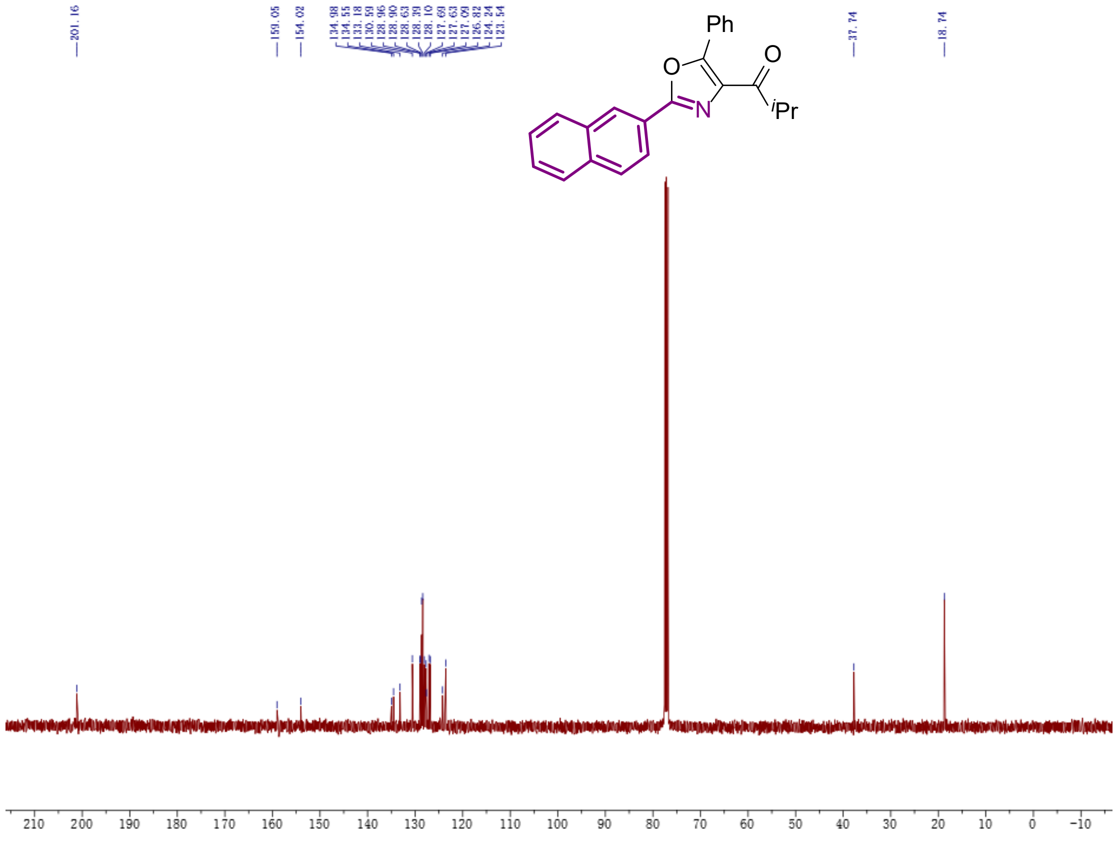

^{13}C NMR of the **3ar** (101 MHz, 25 °C in $CDCl_3$)

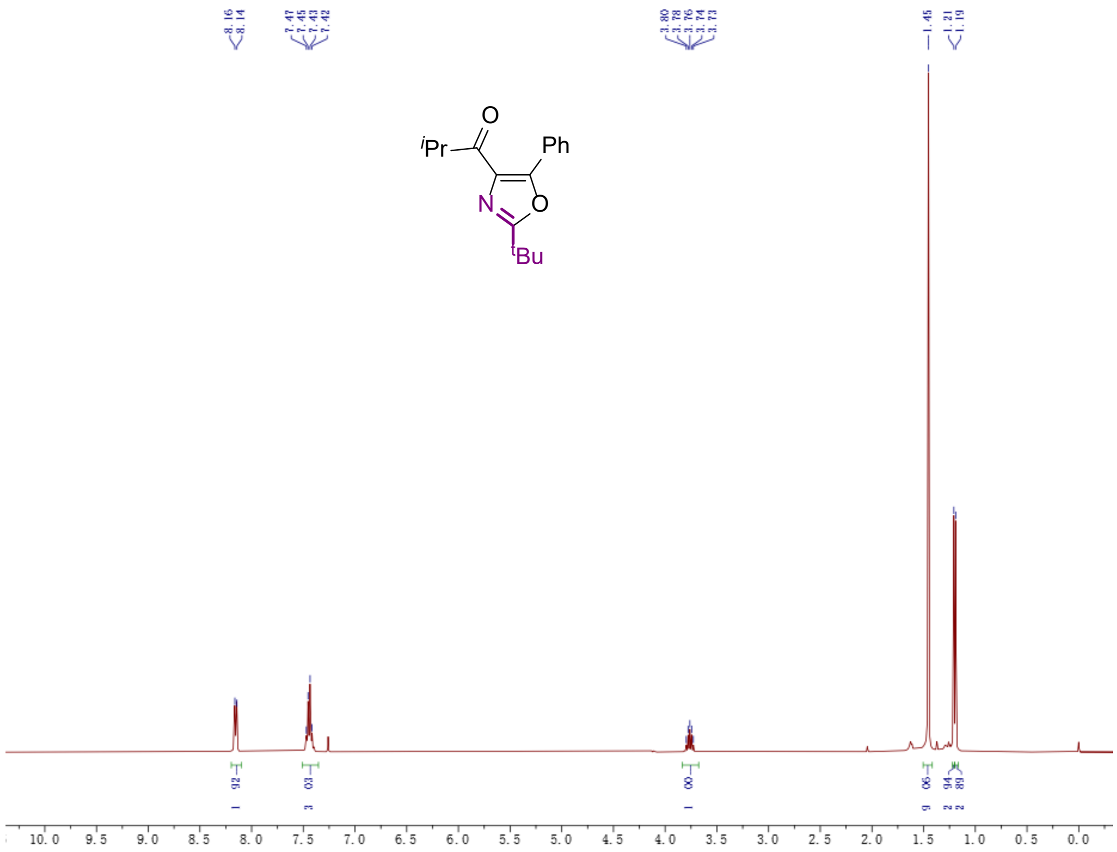

^{1}H NMR of the **3as** (400 MHz, 25 °C in $CDCl_3$)

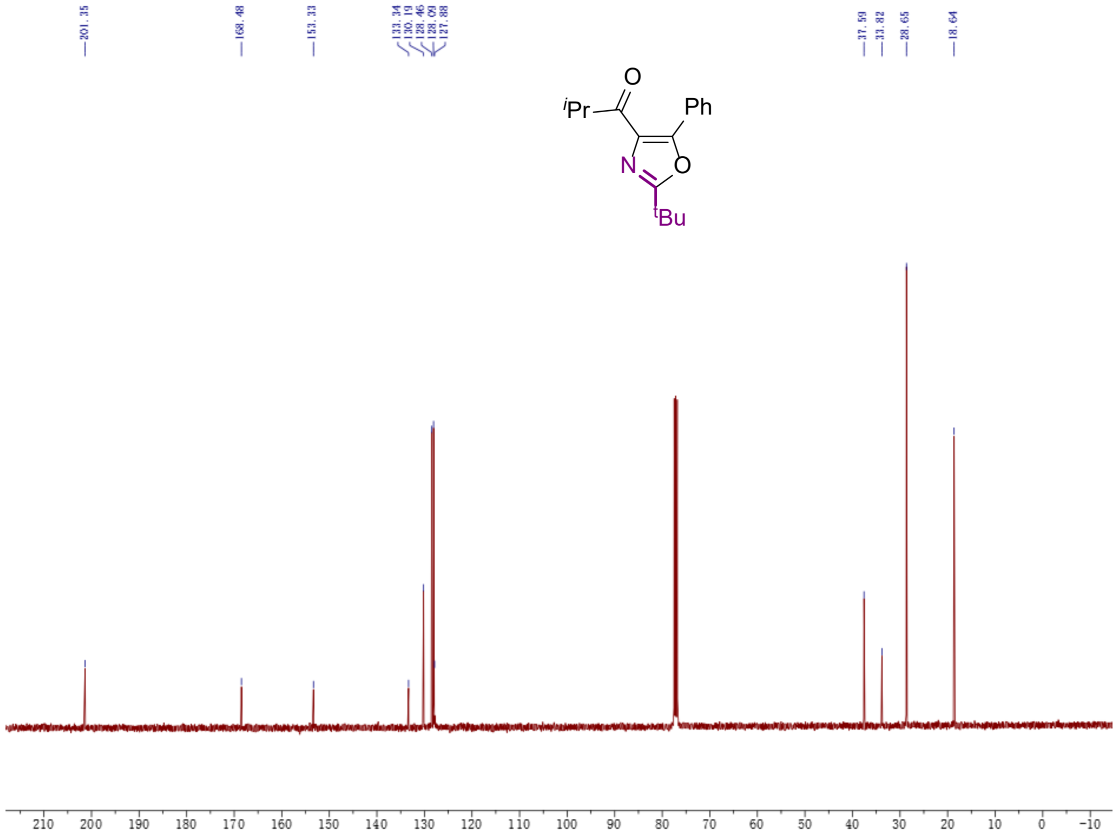

^{13}C NMR of the **3as** (101 MHz, 25 °C in $CDCl_3$)

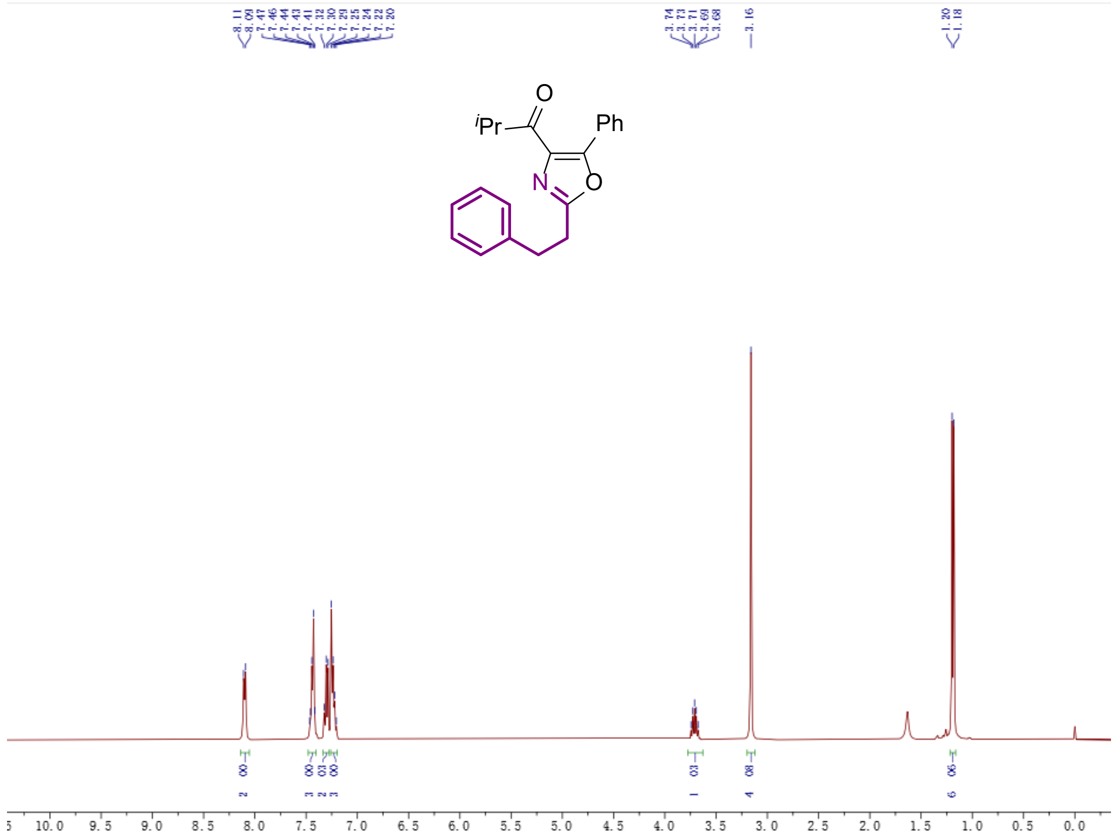

^{1}H NMR of the **3at** (400 MHz, 25 °C in $CDCl_3$)

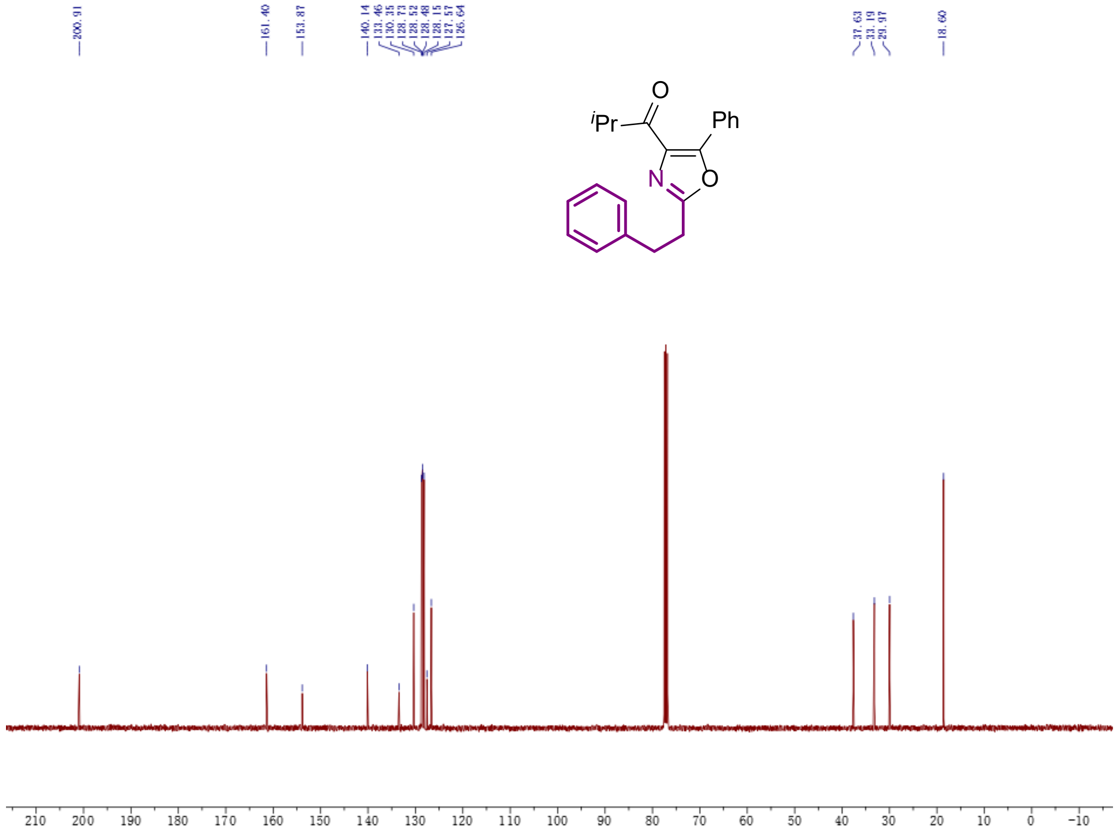

^{13}C NMR of the **3at** (101 MHz, 25 °C in $CDCl_3$)

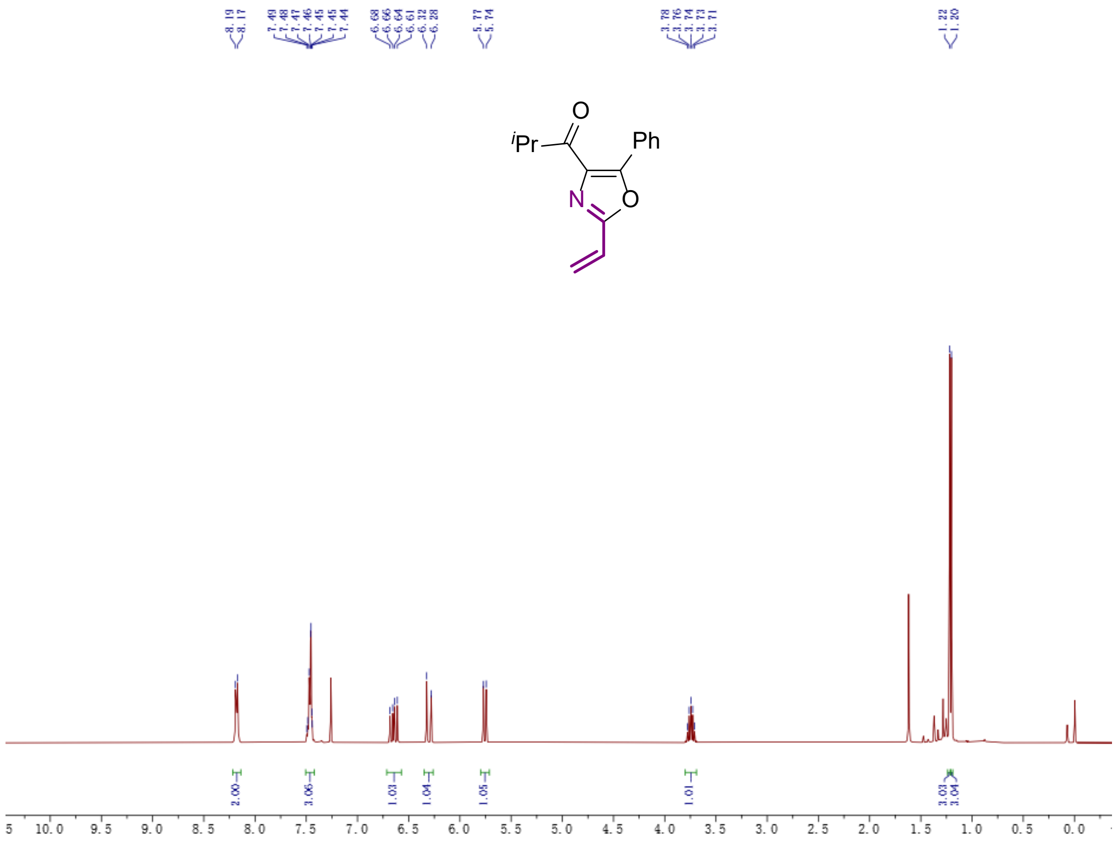

^{1}H NMR of the **3au** (400 MHz, 25 °C in $CDCl_3$)

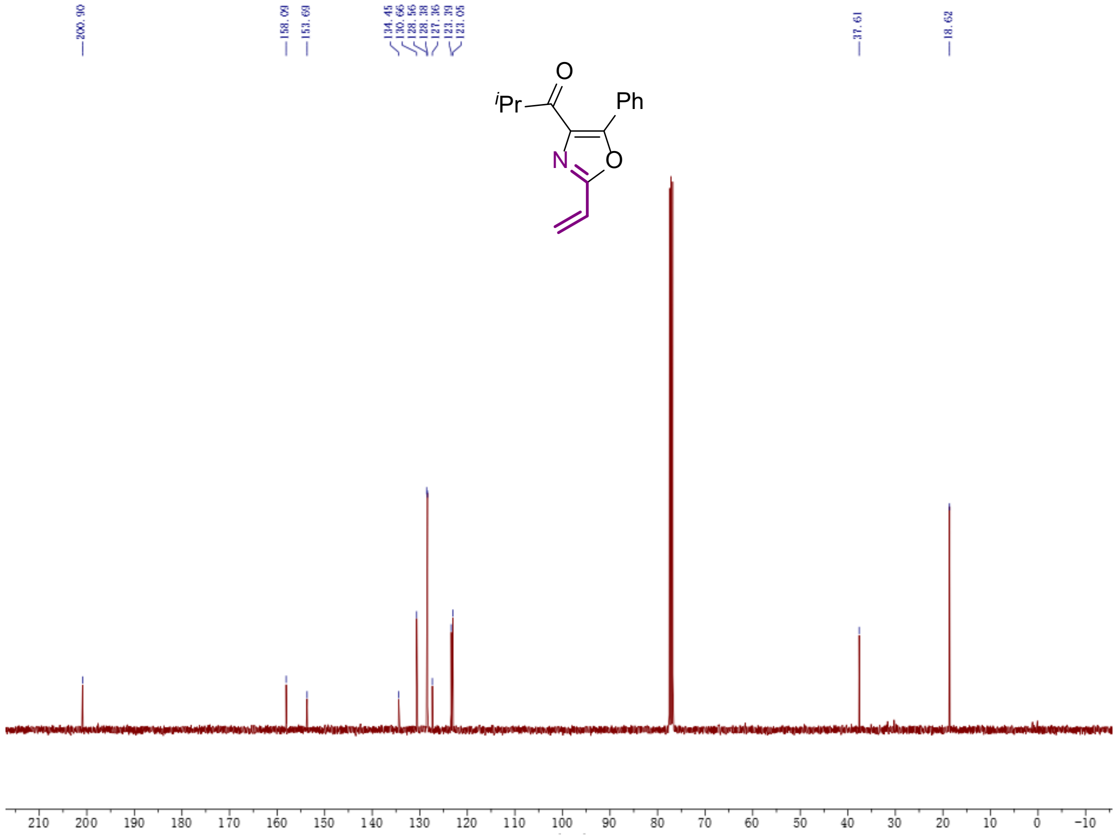

^{13}C NMR of the **3au** (101 MHz, 25 °C in $CDCl_3$)

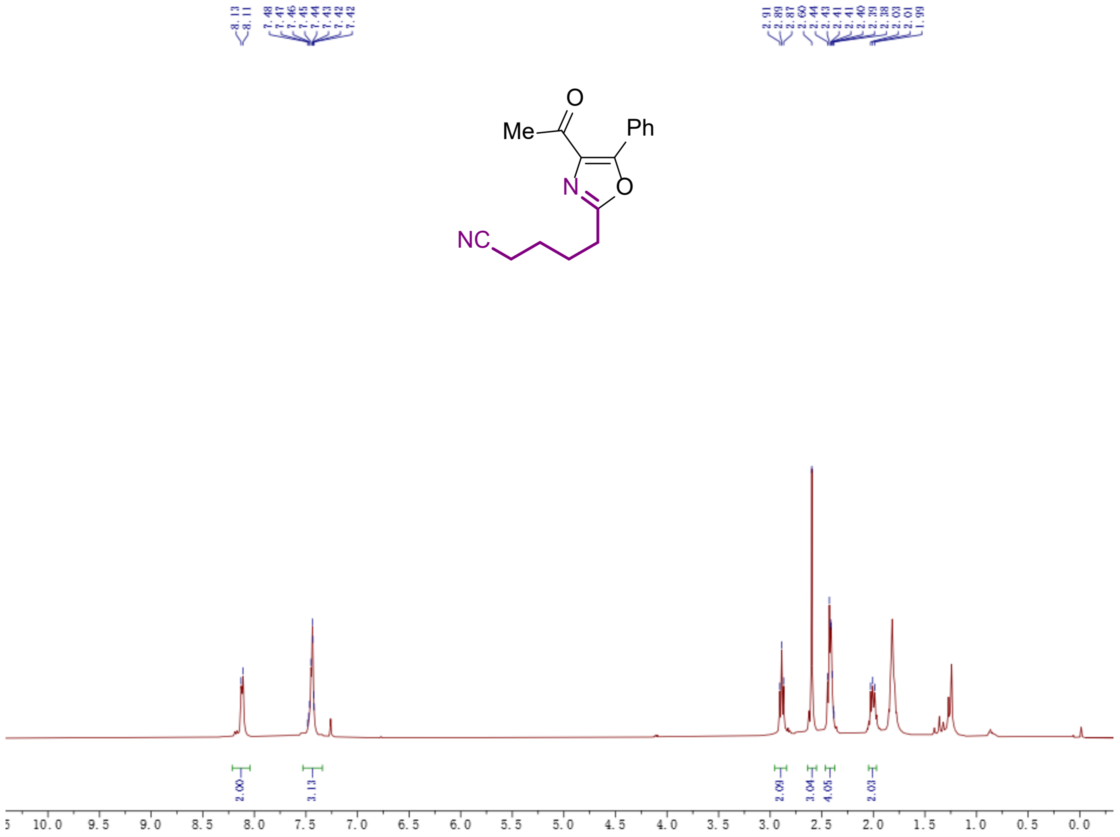

^{1}H NMR of the **3av** (400 MHz, 25 °C in $CDCl_3$)

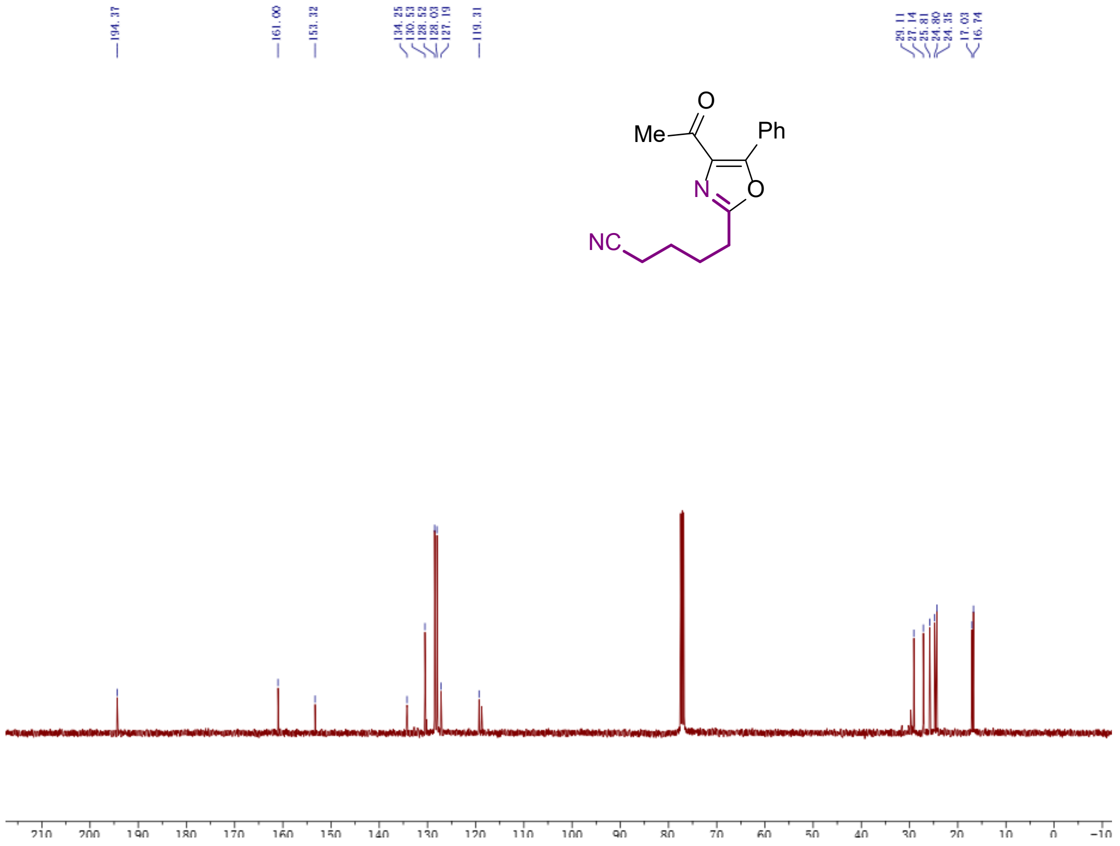

^{13}C NMR of the **3av** (101 MHz, 25 °C in $CDCl_3$)

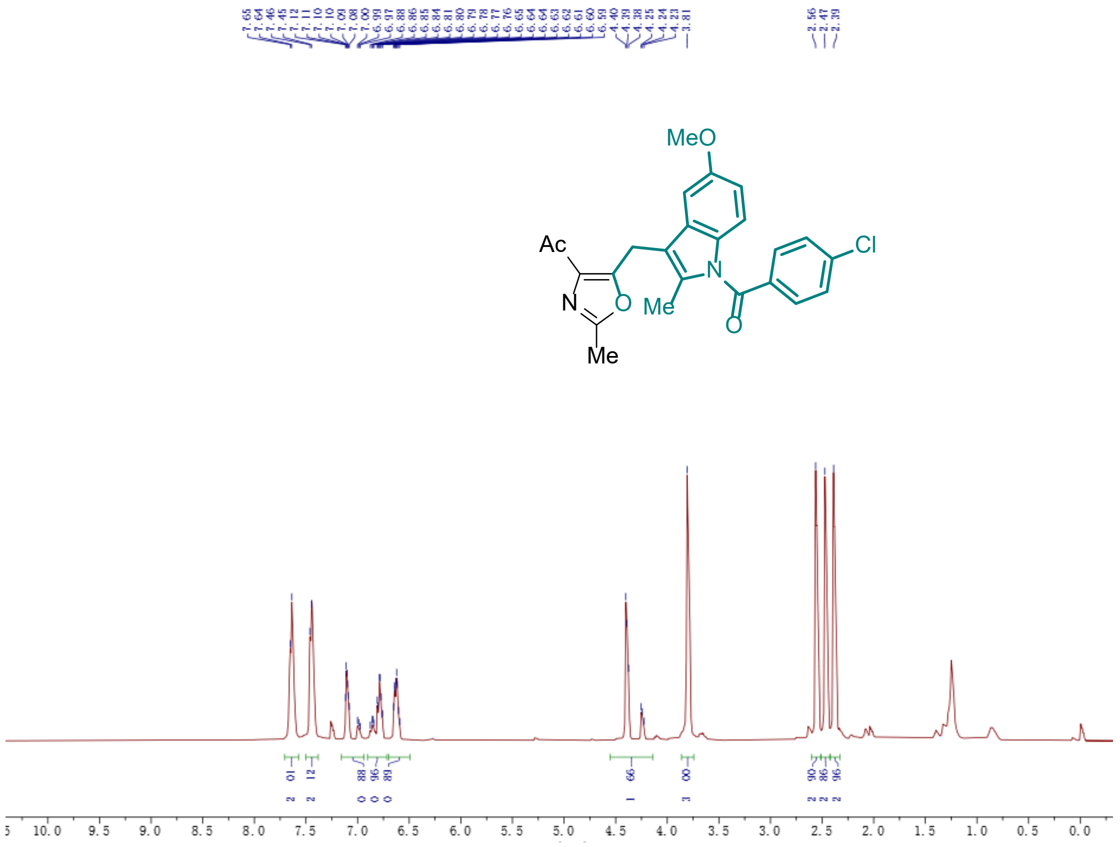

^{1}H NMR of the **4a** (400 MHz, 25 °C in $CDCl_3$)

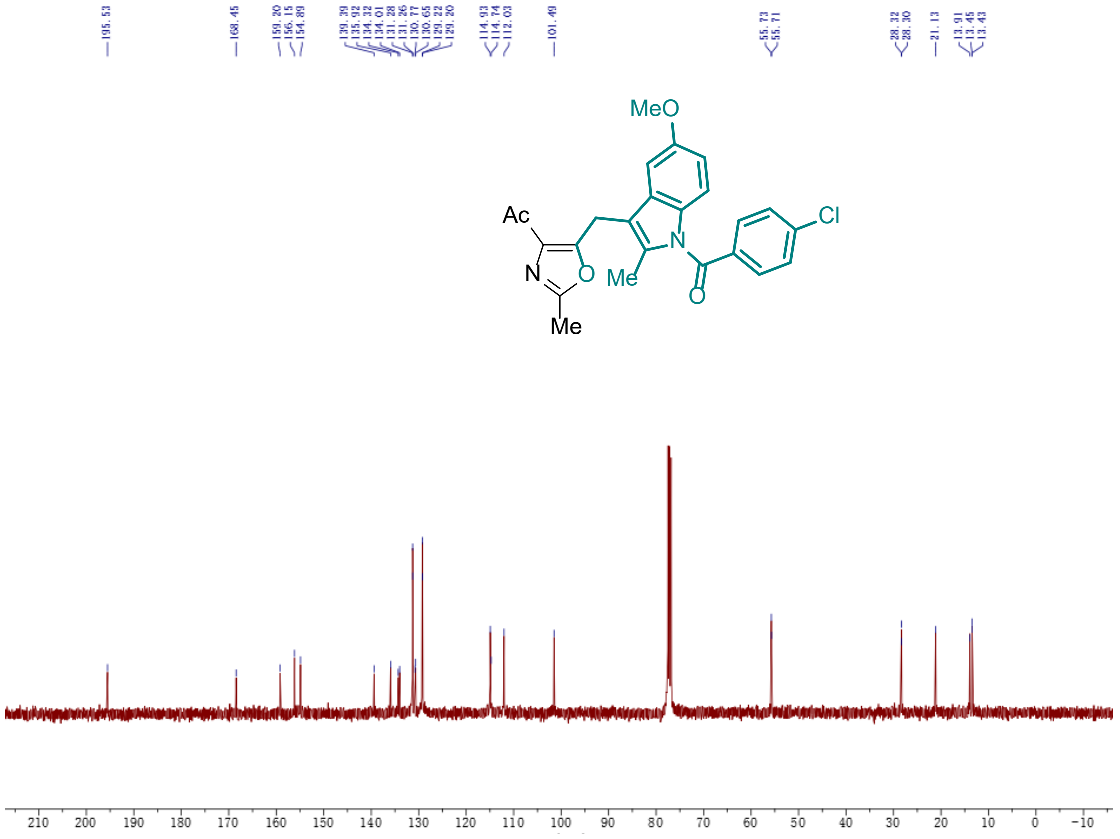

^{13}C NMR of the **4a** (101 MHz, 25 °C in $CDCl_3$)

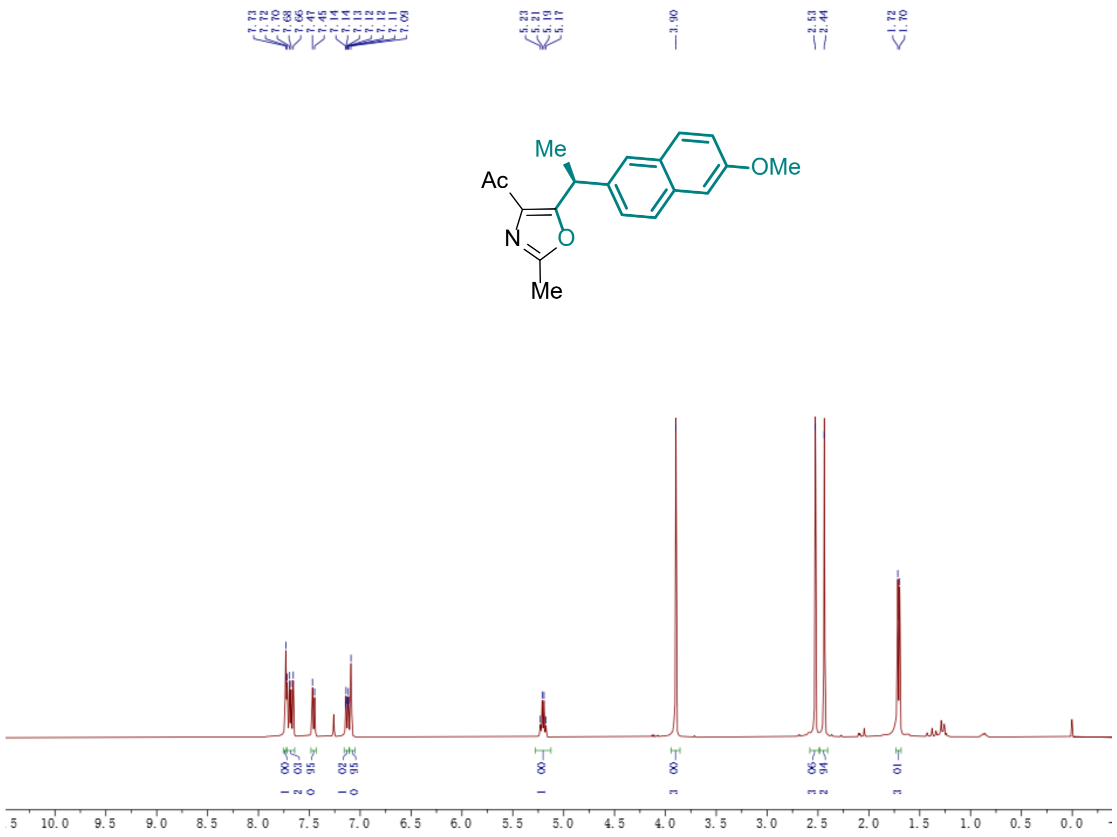

^{1}**H NMR** of the **4b** (400 MHz, 25 °C in $CDCl_3$)

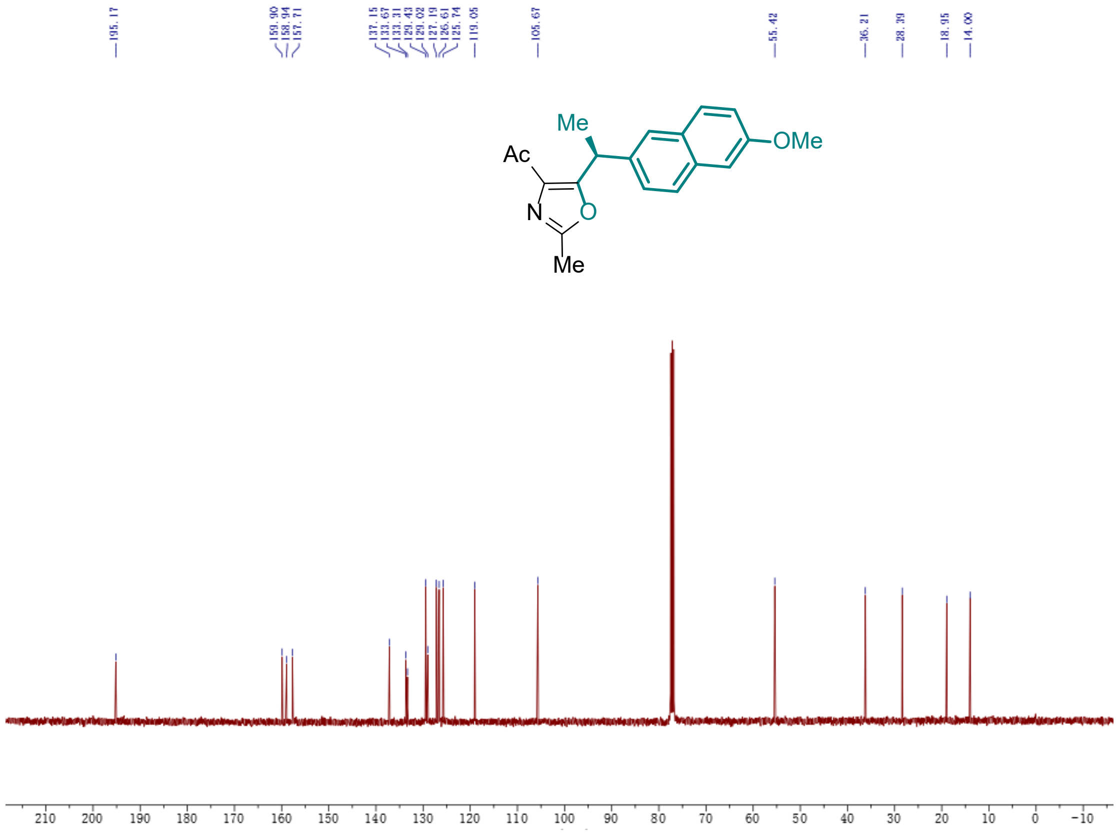

^{13}**C NMR** of the **4b** (101 MHz, 25 °C in $CDCl_3$)

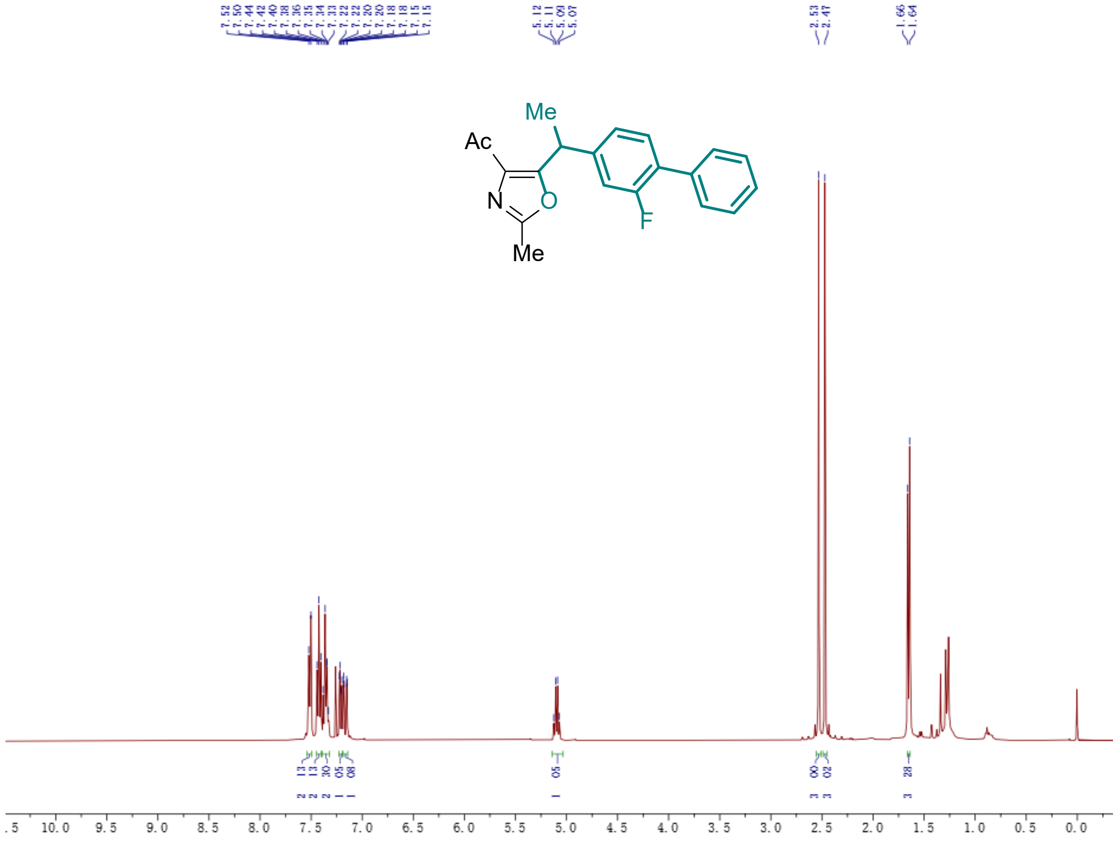

^{1}**H NMR** of the **4c** (400 MHz, 25 °C in $CDCl_3$)

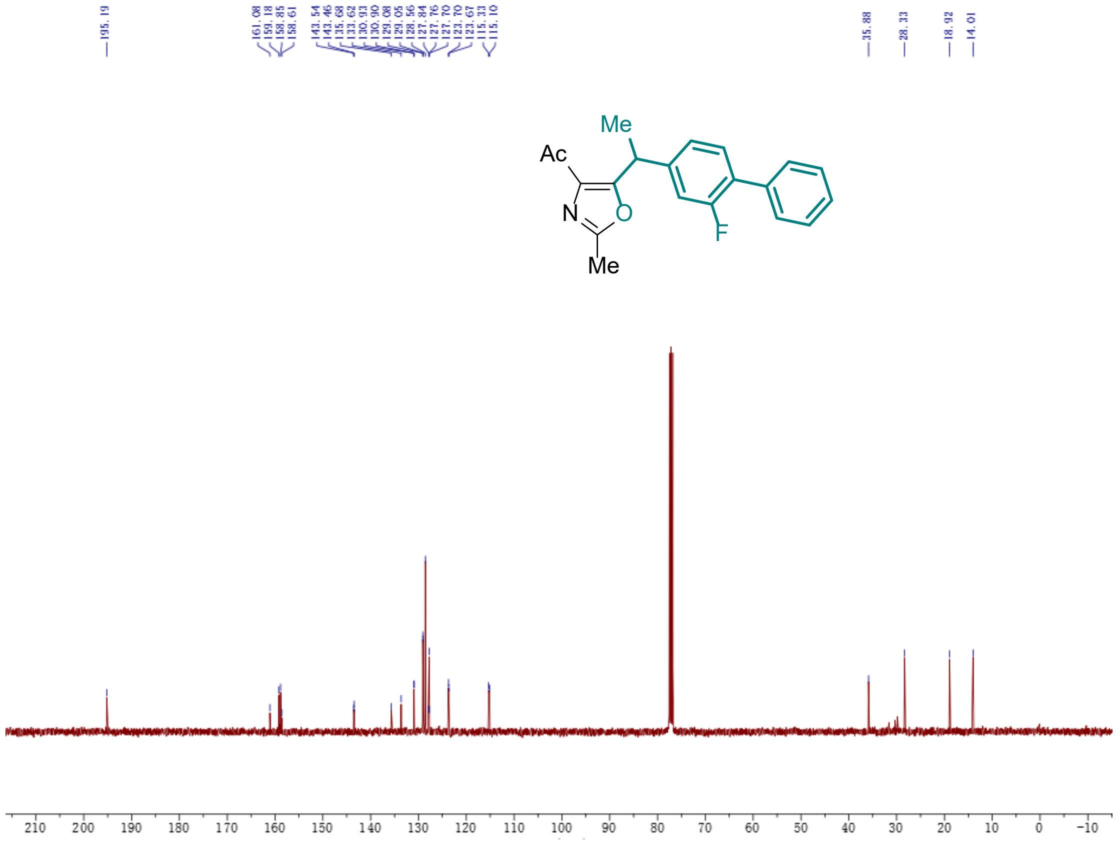

^{13}**C NMR** of the **4c** (101 MHz, 25 °C in $CDCl_3$)

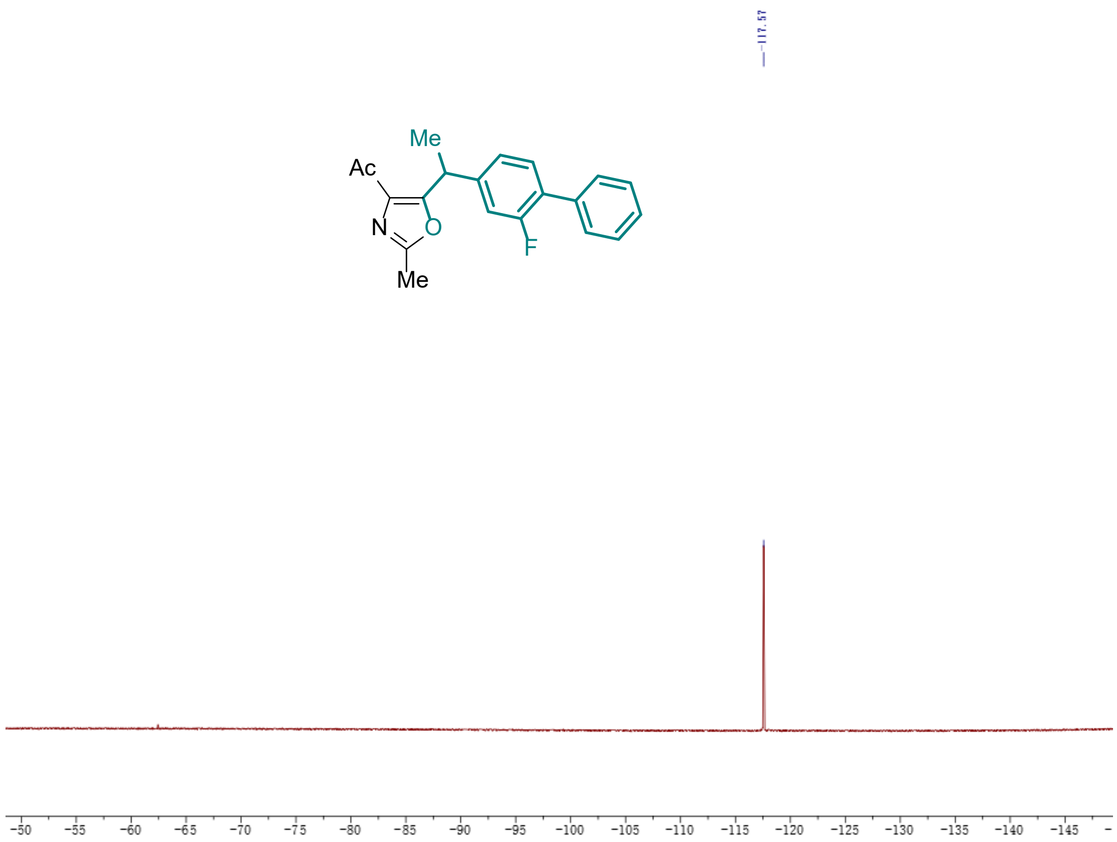

^{19}**F NMR** of the **4c** (376 MHz, 25 °C in $CDCl_3$)

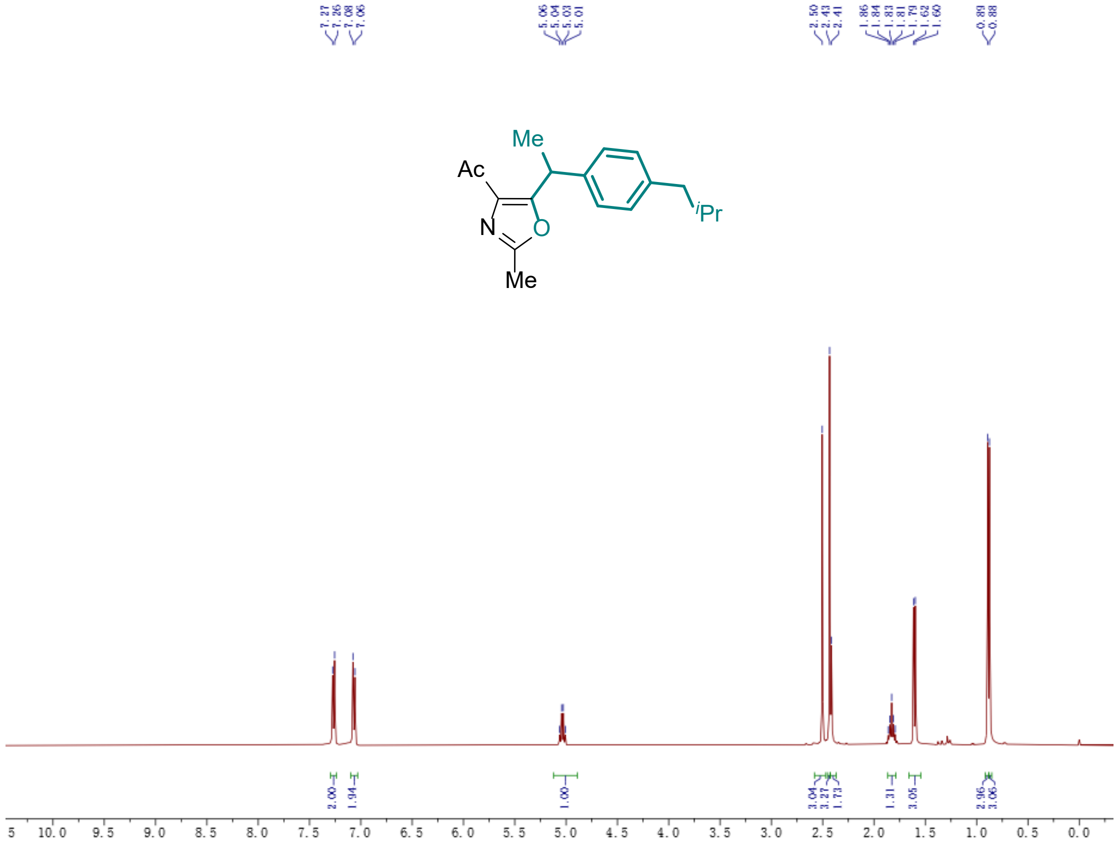

^{1}**H NMR** of the **4d** (400 MHz, 25 °C in $CDCl_3$)

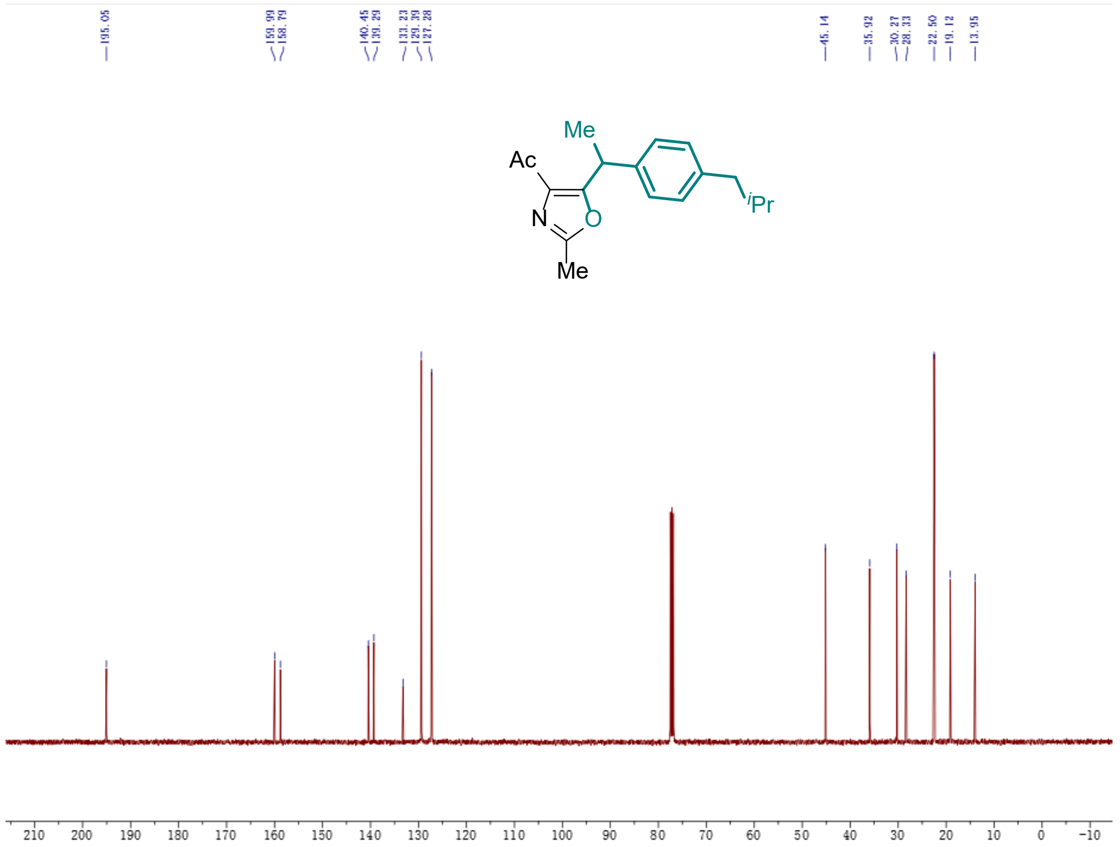

¹³C NMR of the **4d** (101 MHz, 25 °C in $CDCl_3$)

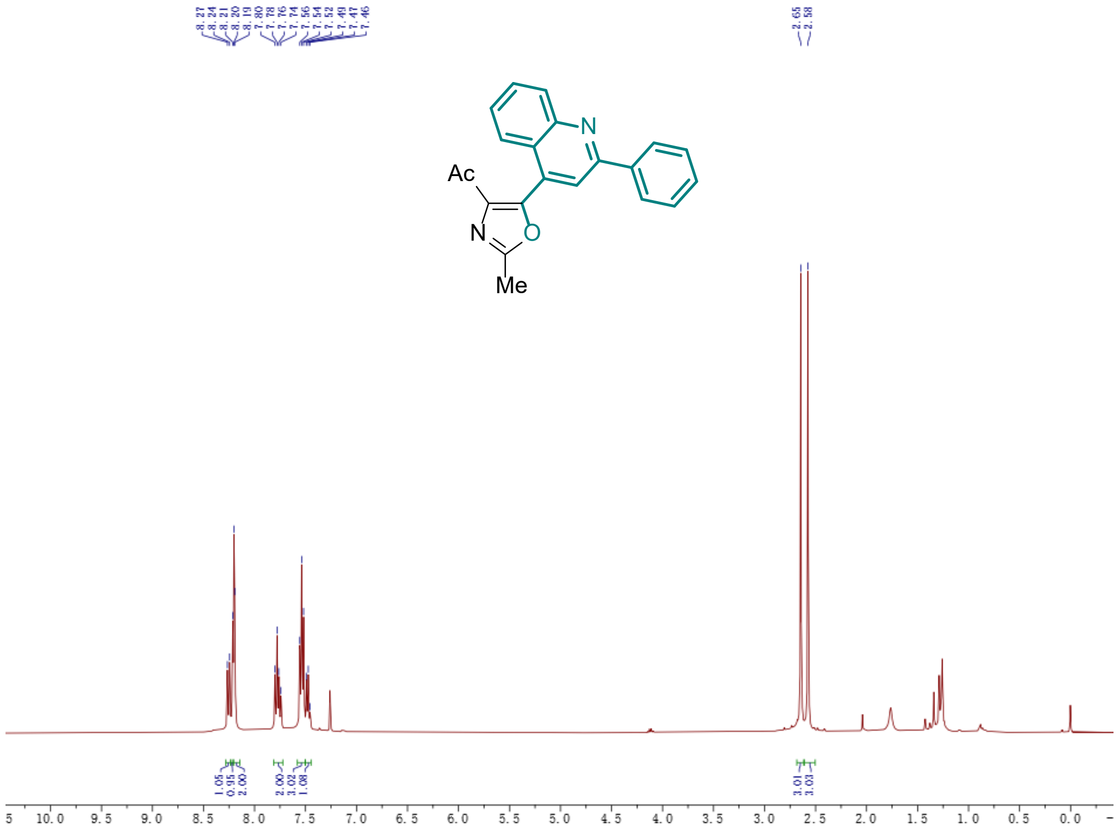

¹H NMR of the **4e** (400 MHz, 25 °C in $CDCl_3$)

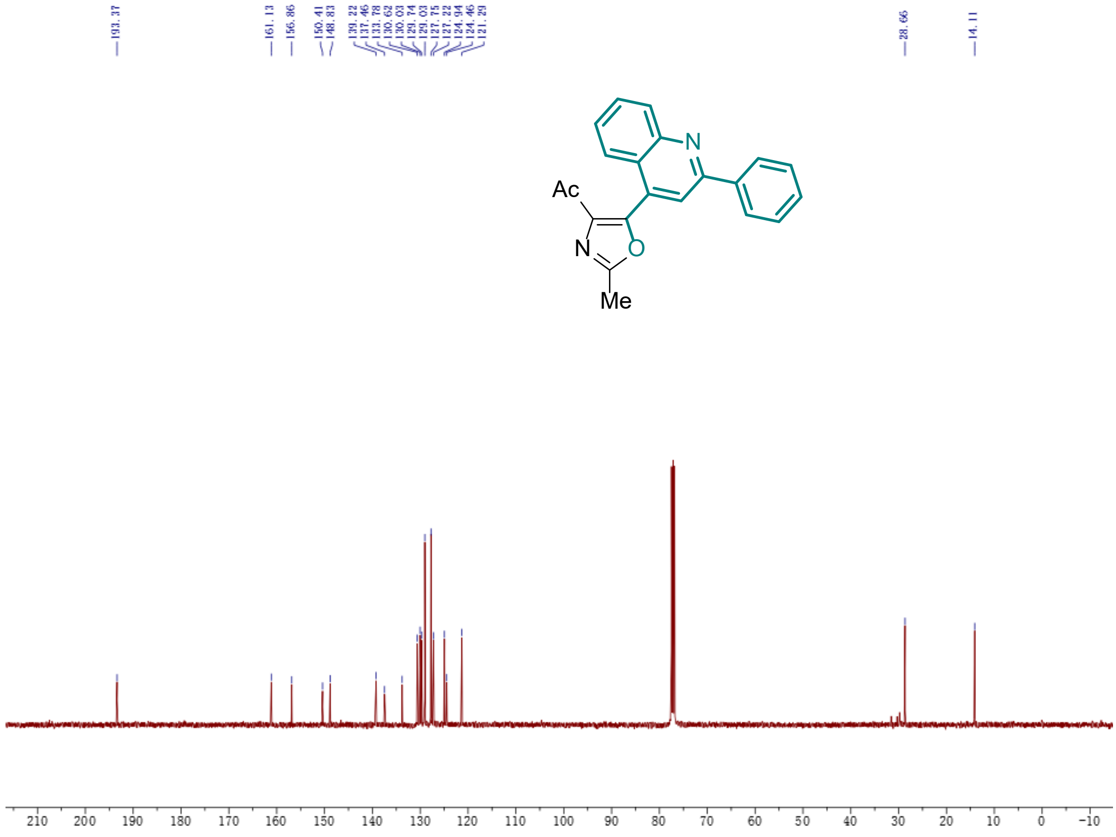

^{13}C NMR of the **4e** (101 MHz, 25 °C in $CDCl_3$)

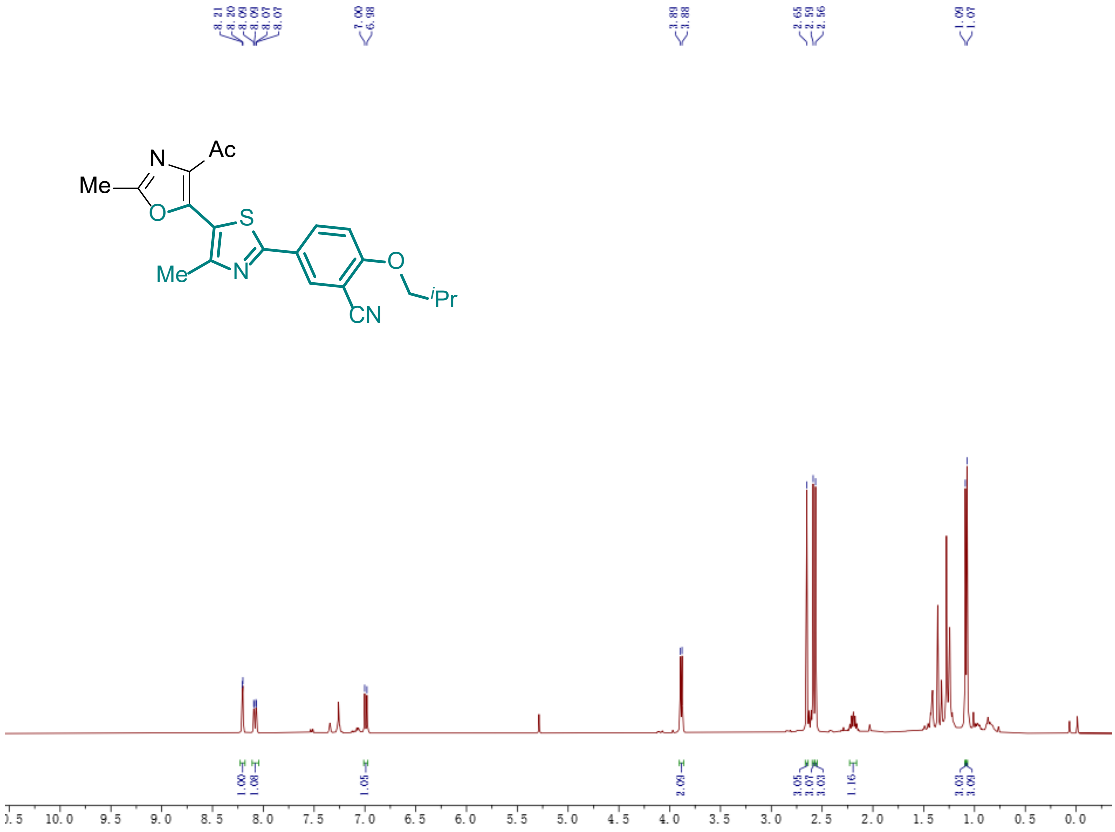

^{1}H NMR of the **4f** (400 MHz, 25 °C in $CDCl_3$)

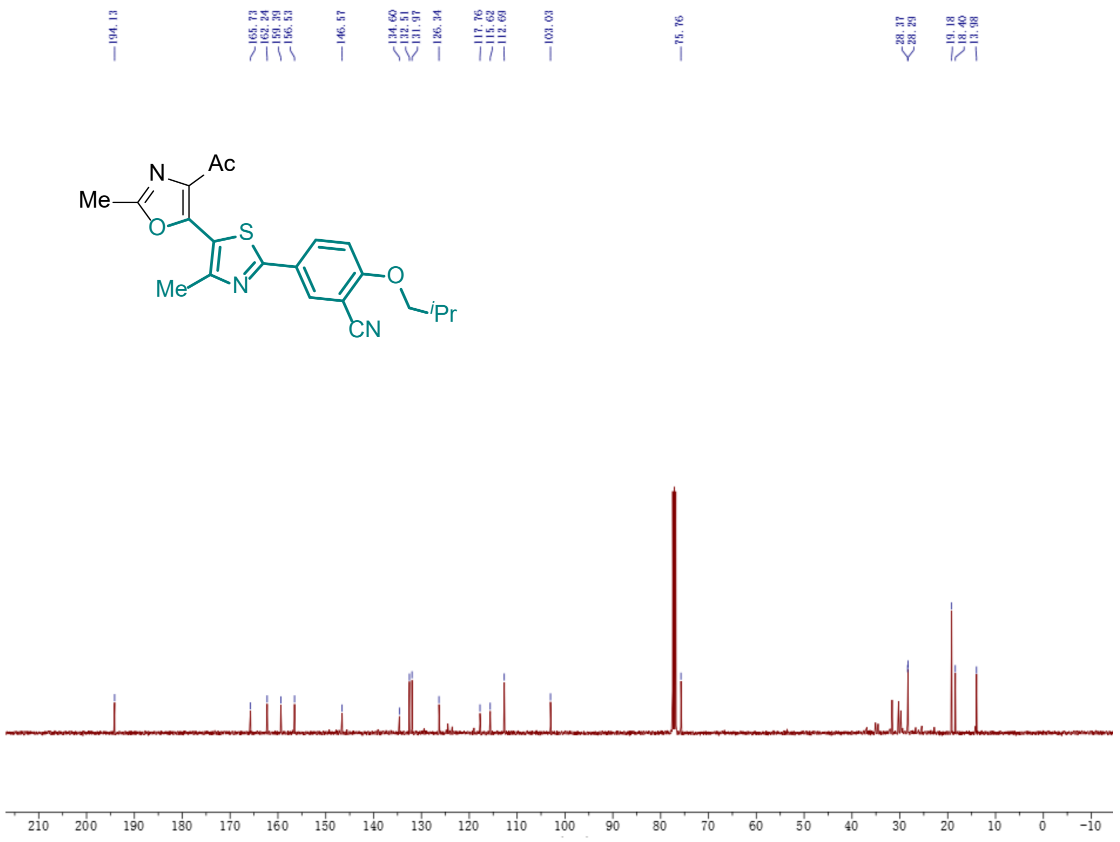

^{13}C NMR of the **4f** (101 MHz, 25 °C in CDCl3)

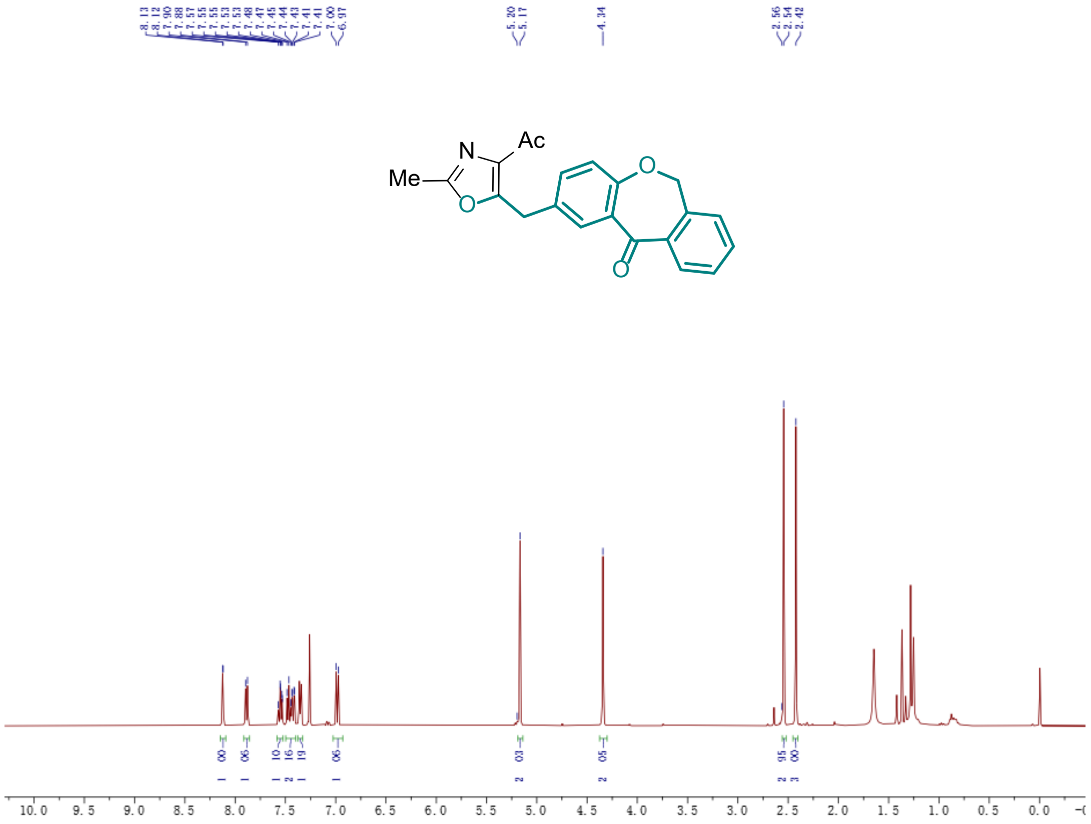

^{1}H NMR of the **4g** (400 MHz, 25 °C in CDCl3)

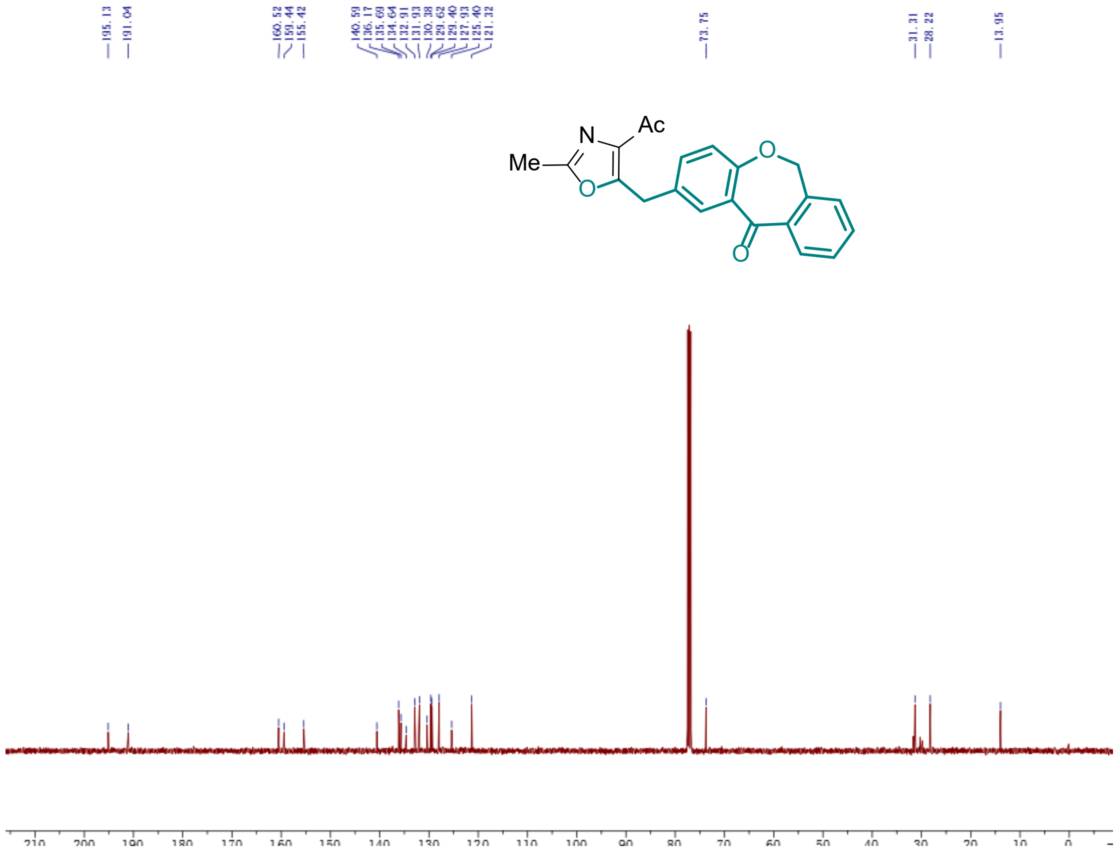

^{13}C NMR of the **4g** (101 MHz, 25 °C in $CDCl_3$)

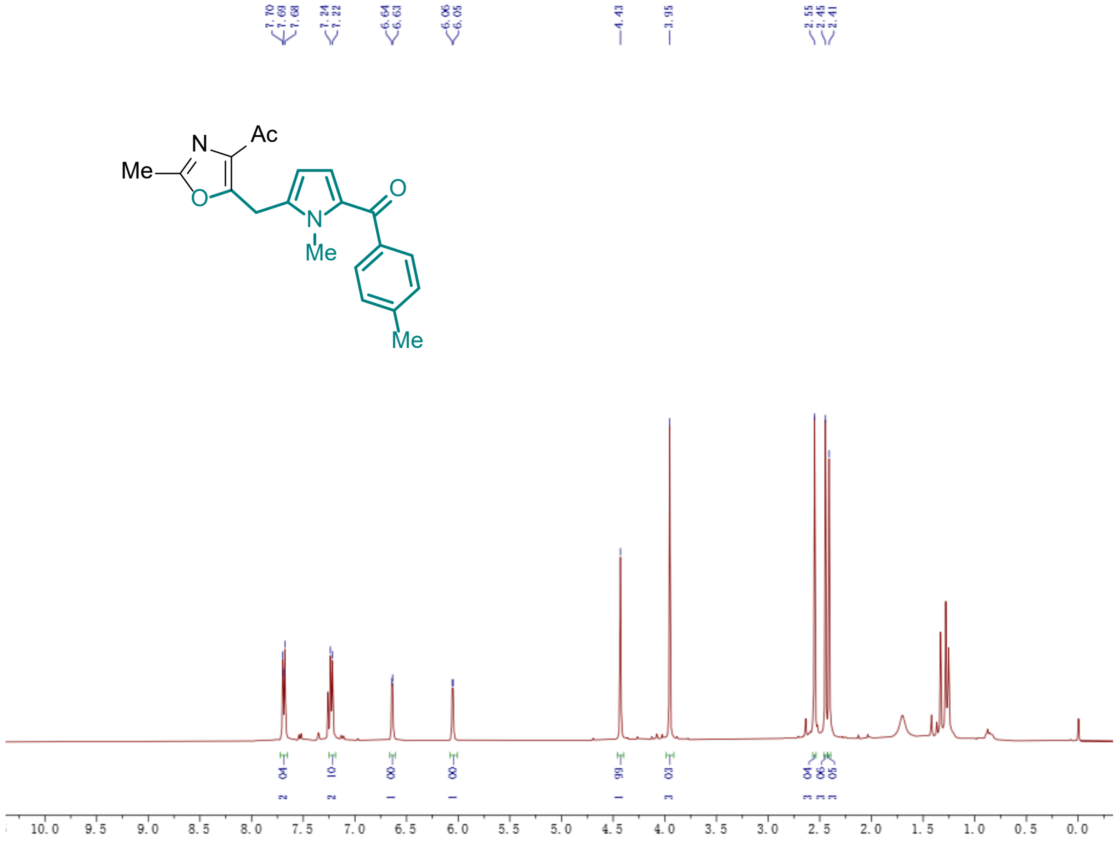

^{1}H NMR of the **4h** (400 MHz, 25 °C in $CDCl_3$)

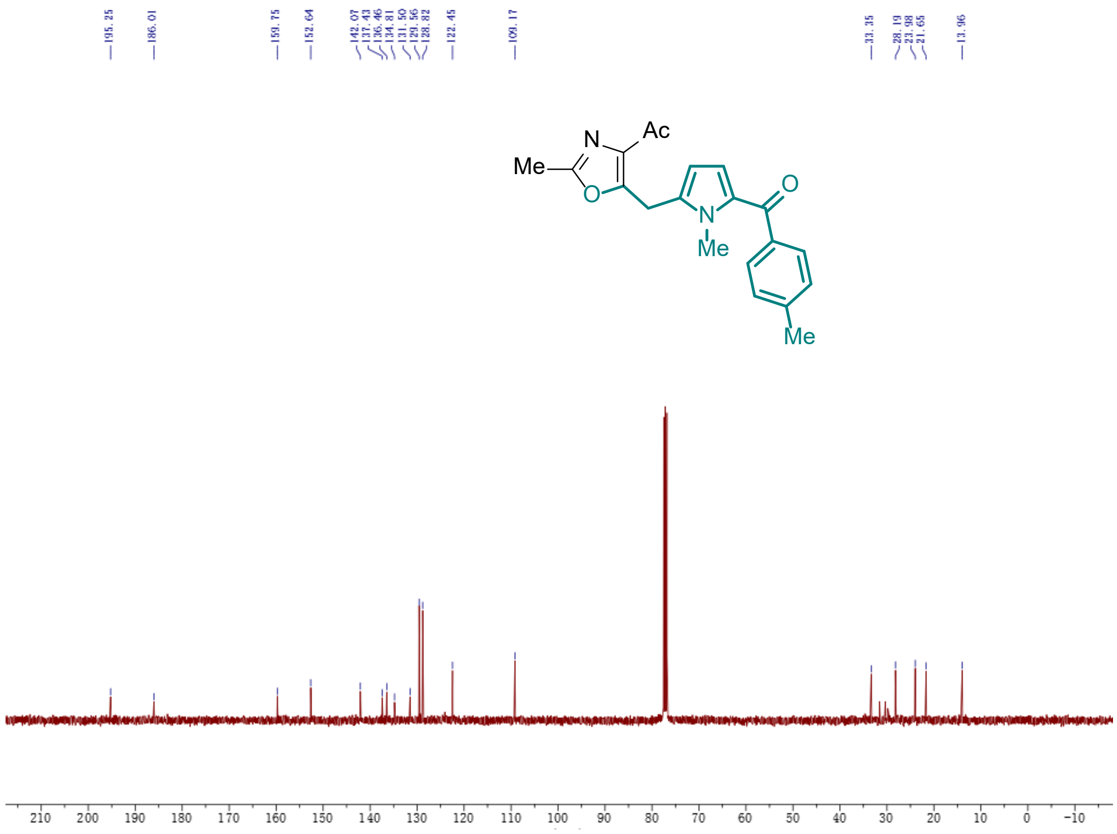

^{13}C NMR of the **4h** (101 MHz, 25 °C in $CDCl_3$)

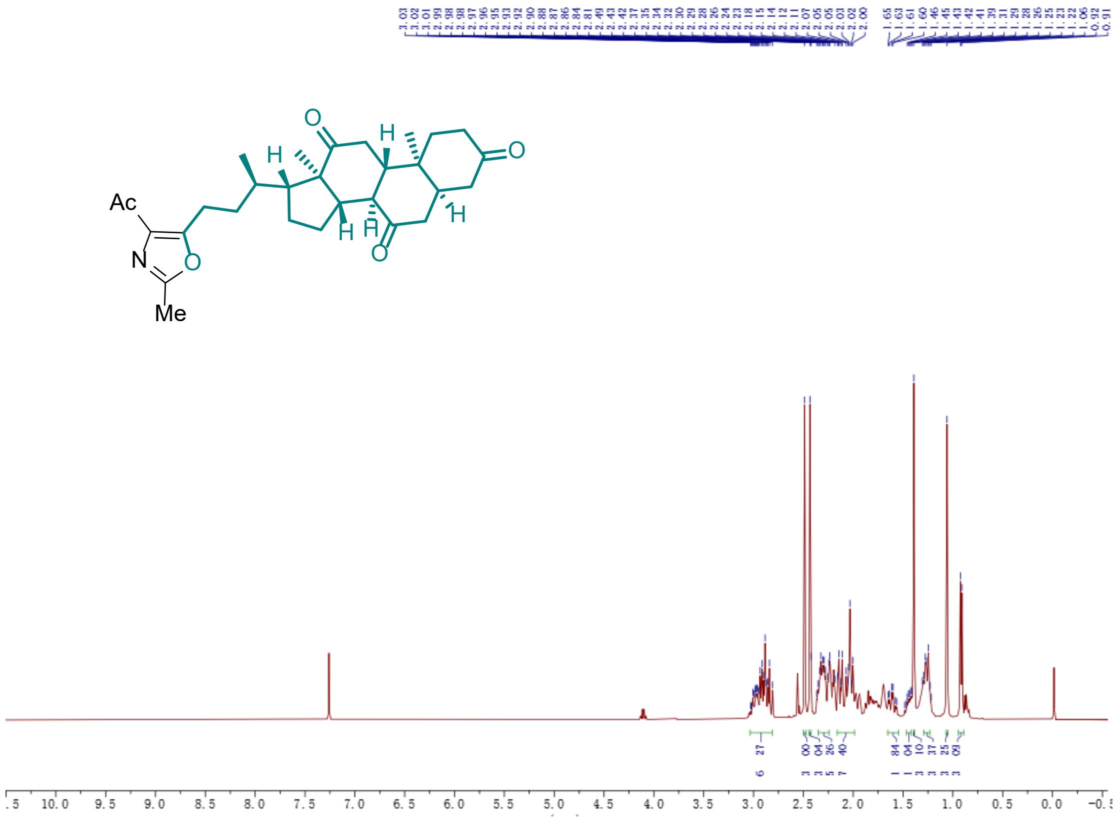

^{1}H NMR of the **4i** (400 MHz, 25 °C in $CDCl_3$)

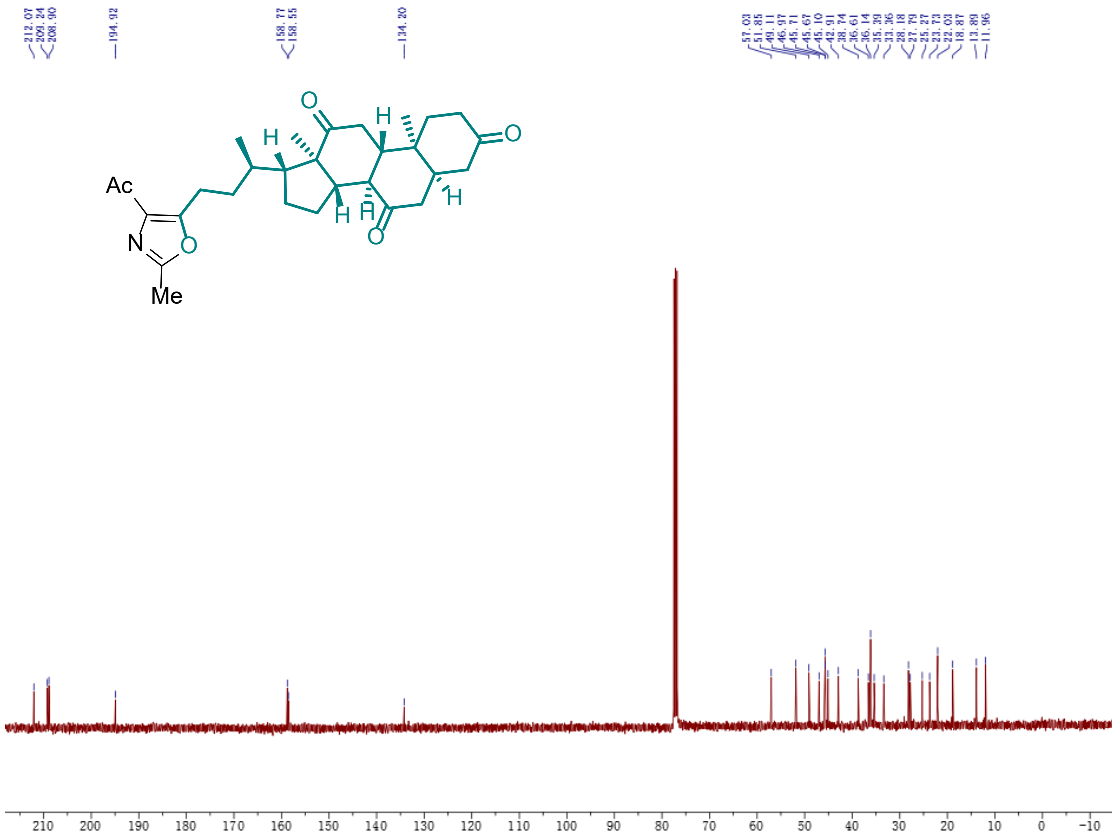

^{13}C NMR of the **4i** (101 MHz, 25 °C in $CDCl_3$)

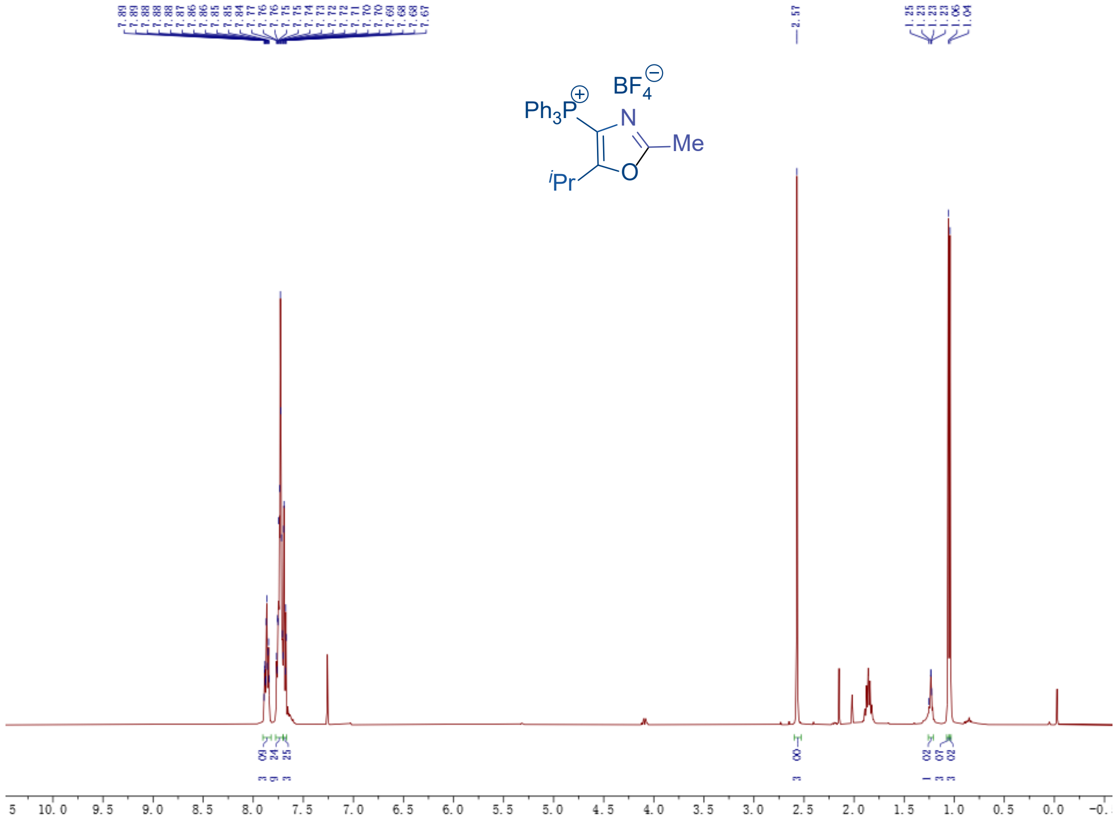

^{1}H NMR of the **5** (400 MHz, 25 °C in $CDCl_3$)

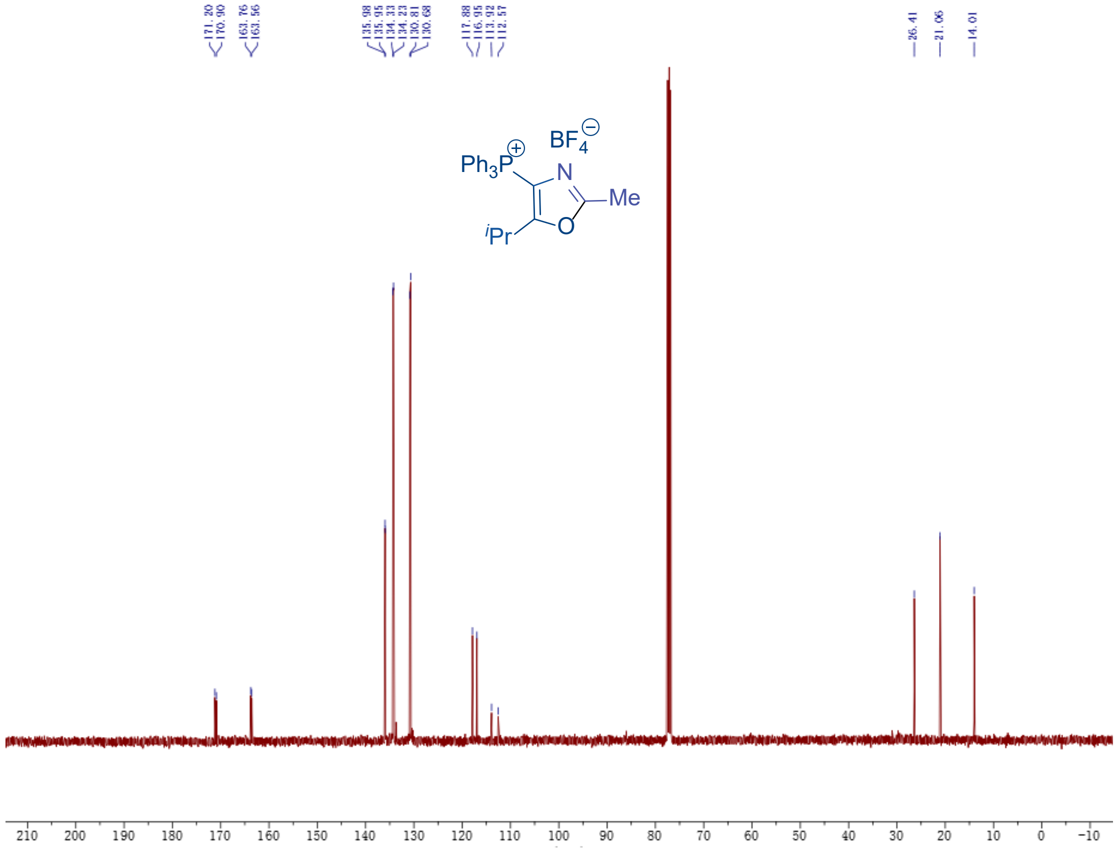

^{13}**C NMR** of the **5** (101 MHz, 25 °C in CDCl$_3$)

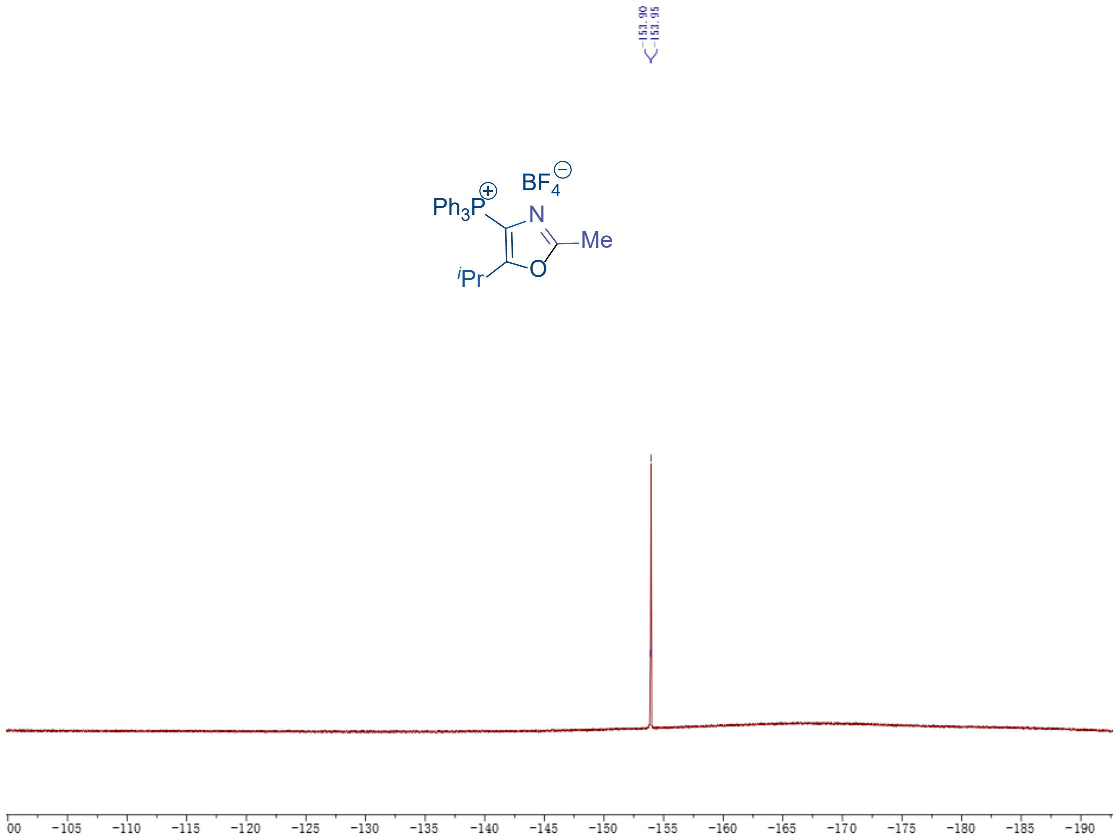

^{19}**F NMR** of the **5** (376 MHz, 25 °C in CDCl$_3$)

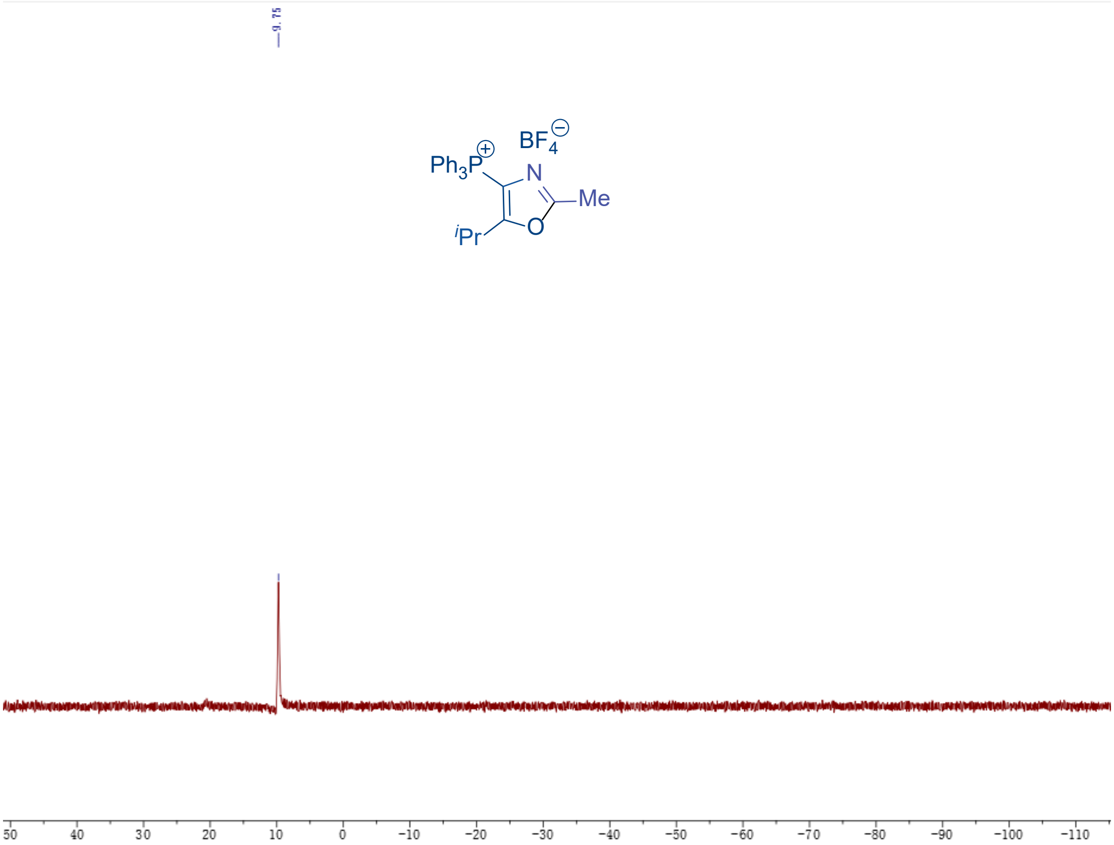

^{31}P NMR of the **5** (162 MHz, 25 °C in $CDCl_3$)

6. References

[1]. Huang, Z.; Yu, X.; Huang, X. *J. Org. Chem.* **2002**, *67*, 8261−8264.

[2]. Yang, X.-Y.; Tay, W. S.; Li, Y.; Pullarkat, S. A.; Leung, P.-H. *Organometallics* **2015**, *34*, 5196.

[3]. Zhdankin, V. V.; Maydanovych, O.; Herschbach, J.; Bruno, J.; Matveeva, E. D.; Zefirov, N. S. *J. Org. Chem.* **2003**, *68*, 1018−1023.

[4]. Zörb, A.; Brückner, R. *Eur. J. Org. Chem.* **2010**, 4785−4801.
